# Continuous Photoflow Synthesis of Heterohelicenes

**DOI:** 10.1021/acs.orglett.6c00559

**Published:** 2026-05-05

**Authors:** Katherine Lyon, Chenyu Pan, Shainthavaan Sathiyalingam, Yang Wu, Jayden Matthews, Jochen R. Brandt

**Affiliations:** † School of Physical and Chemical Sciences, 4617 Queen Mary University of London, 327 Mile End Road, London E1 4NS, U.K.; ‡ Department of Chemistry and Molecular Sciences Research Hub, 4615 Imperial College London, White City Campus, 82 Wood Lane, London W12 0BZ, U.K.

## Abstract

Heterohelicenes continue to attract interest for their potential applications as organic chiral materials. To keep up with the demand for novel heterohelicene structures, expedient and scaleable synthetic procedures are required. We report a “Mallory” photocyclization methodology in continuous flow for the synthesis of thia-, oxa-, and azahelicenes. This procedure has been successfully scaled up to 9.8 mmol and can be applied to an iterative modular synthesis.

## Graphical abstract

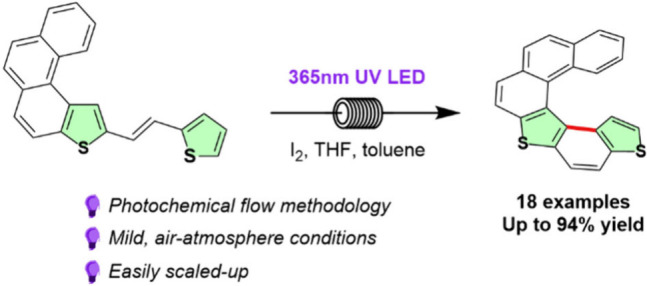

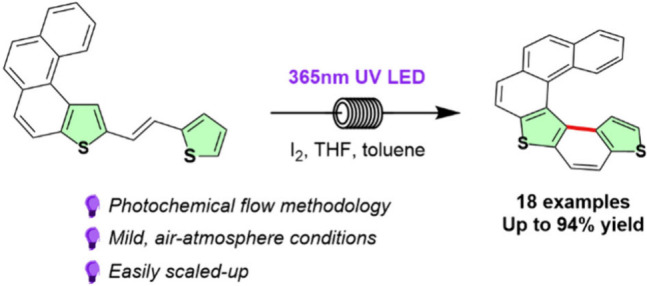

Helicenes are polyaromatic compounds, composed of *ortho*-fused aromatic rings, that are known for their unusual, screw-like chiral structure.^1^ This combination of helical chirality and polyaromatic structure results in both highly dissymmetric interaction with circularly polarized (CP) light and high charge transport.^2–5^ Thus, helicenes have found applications as semiconductors in OFETs or for the emission or detection of CP light.^6,7^ Perhaps the most intriguing application is the potential use of helicenes as spin-filters. Helicene enantiomers may be able to achieve spin-selective electron transport,^8–12^ also known as the chiral induced spin selectivity (CISS) effect,^13^ opening up the potential for helicenes to be used in a variety of “spintronics” devices.^14,15^

A common feature in many helicene designs is the inclusion of a heteroatom into the helical backbone.^16^ This is often highlighted by studies as being crucial in influencing the physical and optoelectronic properties of a helicene.^17–20^ However, relatively few studies have been dedicated to investigating synthetic strategies toward these heterohelicenes. Existing examples include photochemical methodologies as applied to the synthesis of azahelicenes by the Caronna group^21,22^ and a variety of thiahelicenes synthesized by the Wynberg group.^23–25^ Mallory photocyclizations^26^ are one of the most common strategies to access heterohelicenes, but nonphotochemical methodologies such as [2+2+2] cycloisomerizations^27–29^ are also often employed.

Photoflow methodologies are an often overlooked synthetic strategy for accessing heterohelicenes. Photoflow chemistry has practical advantages over batch chemistry, including straightforward scalability, shorter reaction times, and a safer reaction setup, which are desirable traits for industrial synthesis.^30,31^ A handful of examples of helicenes and heterohelicenes synthesized in flow have been reported, but very few of these studies have explored the preparation of heterohelicenes by photocyclization.^32–35^

In this work, we report the Mallory photocyclization of a series of heterohelicenes using a photochemical flow reactor. Most substrates were synthesized with a 100 s residence time (corresponding to a 6 mL/min flow rate when using a 10 mL tubing coil), enabling throughputs of up to 195 mg of stilbene precursor/h. A further elaboration of the products to longer helicenes afforded nonsymmetric [6]­di­(thieno)­helicene **4b** in 53% yield over three steps from stilbene **1b** (Figure 1).

**Figure 1:**
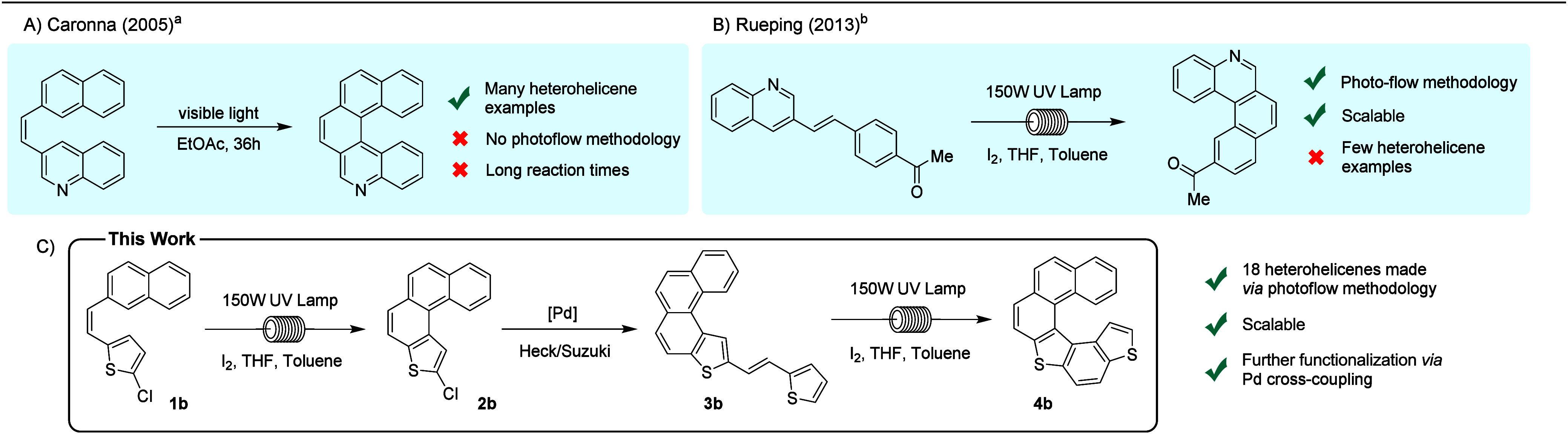
(A and B) Previous syntheses of heterohelicenes by Mallory photocyclization. (C) This work: photoflow synthesis of heterohelicenes. ^a^From ref 21. ^b^From ref 35.

The preparation of the heterostilbenes was achieved via a Wittig reaction between literature-known Wittig salts^36,37^ and commercially available heteroaromatic aldehydes (Table S1). The resulting products are *E*/*Z* mixtures of stilbenes, but due to rapid photoisomerization, both isomers can be used in the cyclization.^38^ We used a Vapourtec UV-150 photochemical reactor and a 150 W (365 nm) UV lamp, a lab-scale setup that provides yields comparable to those of commercial pilot-scale systems while slightly underperforming commercial or 3D-printed lab-scale systems for photon-intensive applications.^30,39^ Optimization of the cyclization of heterostilbene **1a** (Table S2) to [4]­thienohelicene **2a** was achieved in 94% yield (conditions A, Table 1). The Mallory reaction proceeds through an initial 6π photocyclization step, after which an oxidant such as iodine or oxygen is required to complete the reaction via rearomatization.^2^

**Table 1:**
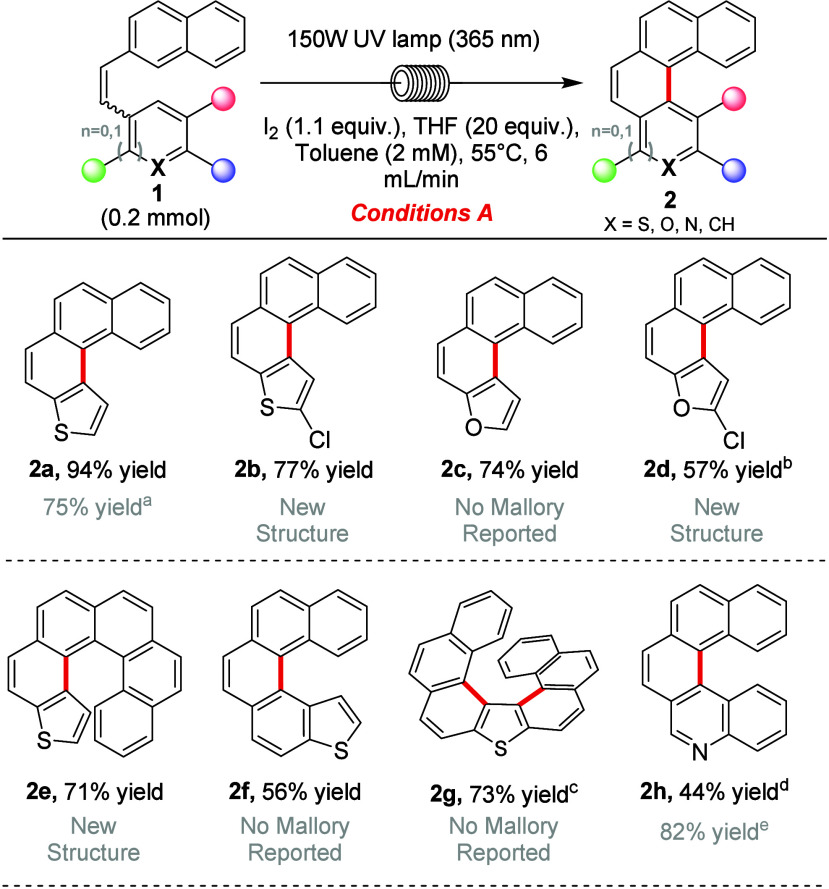
Scope of the Mallory Photocyclization under Conditions A (stoichiometric iodine)

a From ref 40.

b With a 60 W lamp at 40 °C. When using a 150 W lamp at 55 °C and a 2 mL/min flow rate, the yield was 48%.

c At a concentration of 0.5 mM.

d With a 60 W lamp in a 1:1 toluene:acetonitrile mixture at 40 °C.

e From ref 21.

To illustrate the scope, we targeted heterohelicenes that were previously unknown or for which no Mallory reaction had been reported (cf. gray text in Table 1). 2-Chloro-substituted [4]­thienohelicene **2b** was obtained in 77% yield on a 0.2 mmol scale and was scaled up to a multigram scale (9.8 mmol) to afford **2b** in 79% yield (see the Supporting Information (SI)). Furan-based helicenes **2c** and **2d** were obtained in lower yields of 74% and 57% respectively, compared to the corresponding [4]­thienohelicenes **2a** and **2b**. A less powerful 60 W lamp was found to be more optimal for the synthesis of **2d**; the use of a 150 W lamp resulted in a lower yield of 48%. [6]­Thienohelicene **2e** and [5]­thienohelicene **2f** were obtained in 71% and 56% yields, respectively. A longer [7]­thienohelicene **2g** was synthesized in a single step in 73% yield through a double-Mallory reaction, though the reaction mixture was diluted to 0.5 mM to accommodate the low solubility of heterostilbene precursor **1g** in toluene. The mild conditions of the Mallory reaction provide an alternative synthesis of **2g**, which was previously made via a thermal Newman–Kwart rearrangement.^41^ For quinolinyl substrate **2h**, we found that the use of the 150 W lamp resulted in very low yields, but upon switching to a 60 W lamp, our yield of product **2h** improved to 44%. **2h** was previously prepared in batch by Caronna and co-workers with a yield of 82% over 36 h.^21^ We propose that a weaker lamp may reduce the conversion of desired product **2h** into doubly cyclized perylene-like structure **S2h′**, which has been reported to be a typical side product of [5]­helicene synthesis via a Mallory photocyclization.^26^ Additionally, due to solubility issues of product **2h** in toluene, we used a reaction mixture of equal parts toluene and acetonitrile.

For some substrates, the best conditions used catalytic quantities of iodine (0.1 equiv) and an increased volume of THF (10 vol %) (conditions B, Table 2). These conditions are likely to reduce the level of formation of the highly acidic hydrogen iodide side product, which may protonate *N*-heterocyclic products and cause the formation of a dark blue-black precipitate.^38,42^ Under these conditions, the cyclization of thiazole-containing stilbene **1i** led to a combined 61% yield of ring-closure products. However, desired [4]­thiazolohelicene **2i** was obtained in only 35% yield, comparable to that of a similar thiazole-based helicene.^43^ The remaining mass balance of ring-closure products was side product **S2i′** (see the SI), obtained in 26% yield after the ring opening of product **2i**. No comparable ring opening was observed for [5]­oxazolohelicene **2k**, which was synthesized in 40% yield. Chloro-substituted pyridohelicenes **2m** and **2m′** were afforded in a 7:3 ratio (51% and 19% yields, respectively) in favor of more sterically hindered product **2m**, which is the expected regioselectivity for Mallory cyclizations involving pyridine rings and has been explained through molecular orbital calculations.^26^ Similarly, [5]­thiadiazolohelicene **2n** was obtained in 54% yield alongside 17% of linear side product **2n′**, improving on the literature precedent where the ratio of the desired helicene to the linear product was 1:1.^44^ The synthesis of chloro-substituted pyrido[4]­helicene **2o** was achieved in 78% yield. This higher yield compared to that of **2m** is likely due to the chlorine atom blocking the alternative cyclization location, removing the possibility of a regioisomer.

**Table 2:**
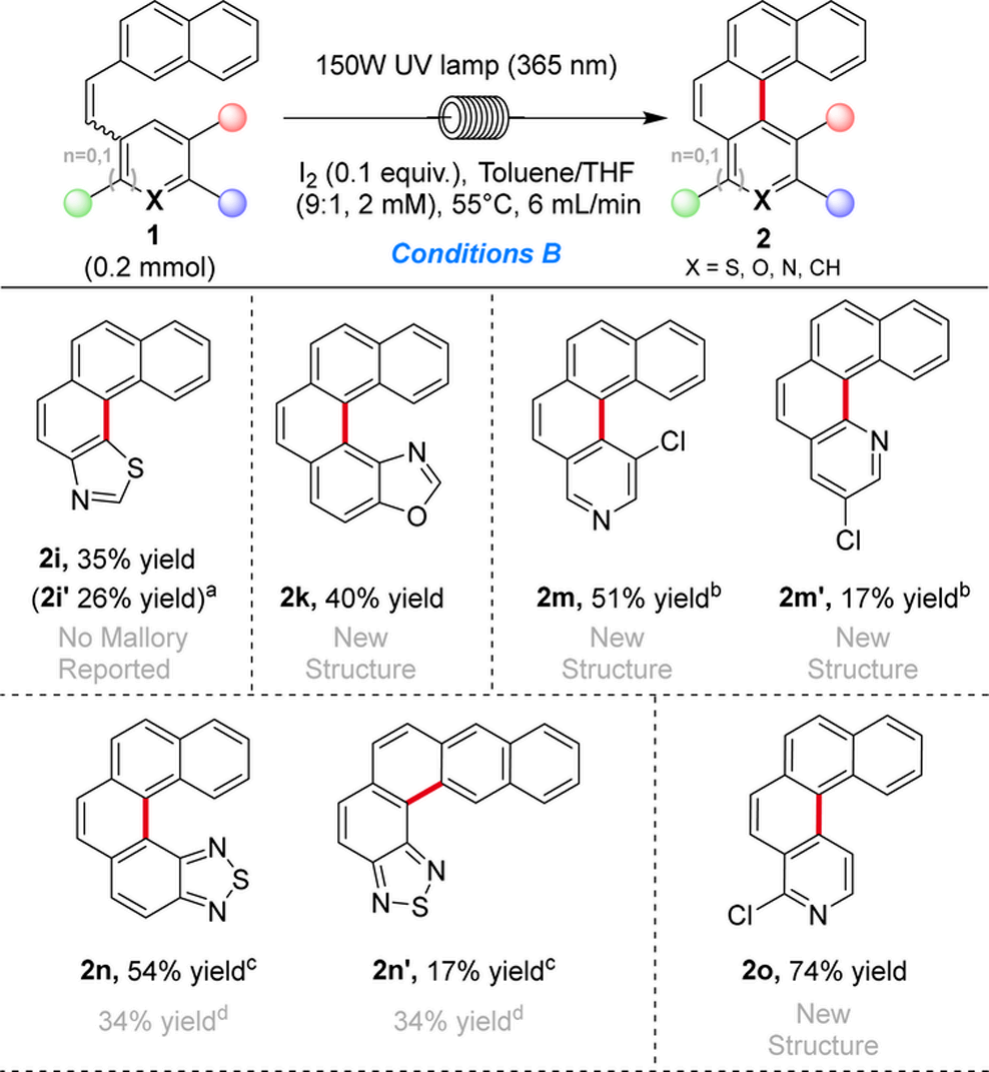
Scope of the Mallory Photocyclization under Conditions B (catalytic iodine)

a Yield of ring-opened side product **S2i′**. See the SI for structure and characterization. Reaction performed on a 0.8 mmol scale.

b Reaction performed on a 0.8 mmol scale.

c With 0.19 equiv of I_2_, 59 equiv of THF, and 1 mM toluene at 65 °C and a flow rate of 3.3 mL/min. Reaction performed on a 0.4 mmol scale.

d From ref 44.

The cyclization of benzothiophene stilbene **1p** afforded [7]­thienohelicene **2p** in 56% yield alongside S-shaped side product **S2p′** (see the SI) in 18% yield (Scheme 1). The double cyclization of carbazole-based **1q** afforded dibromo [7]­pyrrolohelicene **2q** in 50% yield on a 0.32 mmol scale. The reaction was scaled to 1.0 mmol to afford **2q** in 50% yield.

**Scheme 1:**
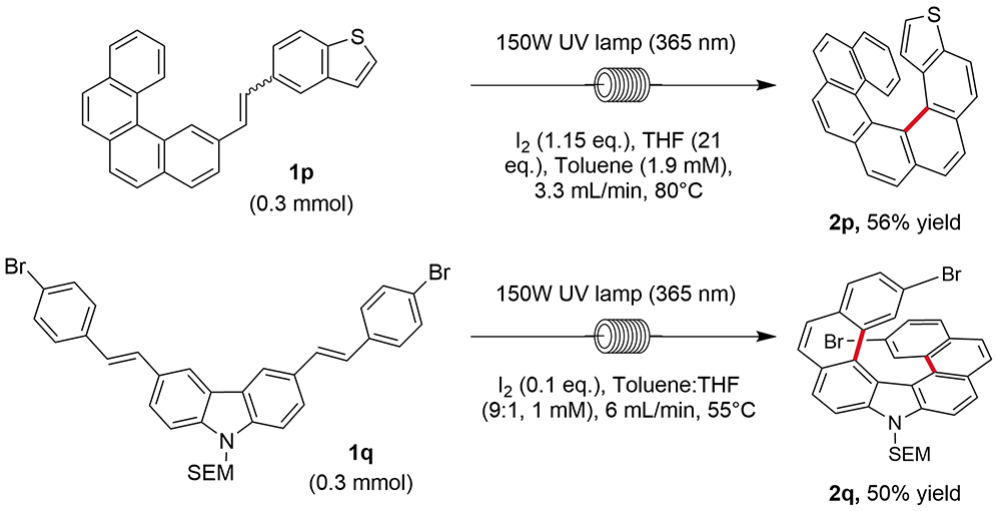
Synthesis of [7]­Heterohelicenes **2p** and **2q**

a For the structure of side product **S2p′**, see the SI.

For the sake of synthetic simplicity, most stilbene substrates were synthesized from naphthyl phosphonium salt **S5a** and a mono- or bicyclic aldehyde, affording [4]- or [5]­helicenes, respectively. While simple to synthesize, such short helicenes enantiomerize at room temperature unless bulky substituents are included. Longer helicenes can avoid this process. The enantiomerization half-life of [6]­helicene is 8 orders of magnitude longer than that of [5]­helicene.^45^ Thus, for chiral materials applications, longer helicenes, which can be resolved into their *P-* and *M-*enantiomers, are desirable.

As the synthesis of polyaromatic Wittig salts such as **S5b** (see the SI) often involves multiple steps, we considered other approaches to elongate a heterohelicene. First, the use of a bicyclic aldehyde in the formation of the stilbene precursor, such as in the case of **1f**, **1p**, and **1h**, adds an extra ring compared to the corresponding stilbene containing monocyclic heteroaromatics at the cost of a reduced yield from alternative cyclization pathways (e.g., **S2p′** (see the SI)). Second, double-Mallory cyclizations, such as those affording **2g** and **2q**, can quickly assemble large structures from simple starting materials. While stilbene **1g** displayed a very low solubility in toluene, a similar problem was avoided for **1q** by using the solubilizing 2-(trimethylsilyl)­ethoxymethyl (SEM) protecting group.

Finally, we decided to investigate a modular stepwise synthesis of longer helicenes. We performed further functionalizations on chloro-substituted [4]­thienohelicene **2b** via Heck^46^ or Suzuki^47^ reactions, creating longer heterostilbene substrates (Scheme 2). Using conditions A (Table 1), we were pleased to synthesize [6]­thienohelicenes **4a** and **4b** in 80% and 87% yields, respectively, some our highest yields so far for a heterohelicene substrate.

**Scheme 2:**
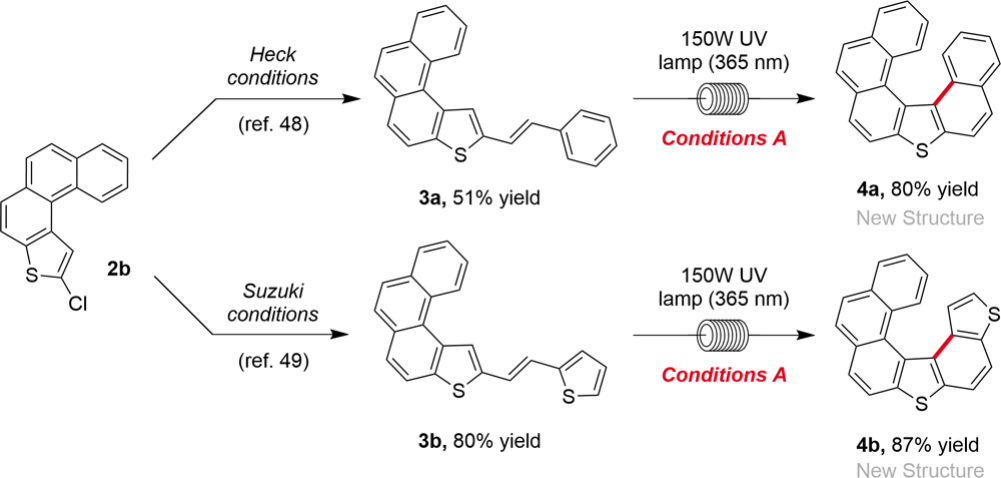
Synthesis of [6]­Thienohelicenes **4a** and **4b** in Two Steps from **2b** (Table 1)

We have developed a robust Mallory photocyclization methodology in flow that is suitable for a wide range of heterohelicenes, as demonstrated by the synthesis of previously unreported thiahelicenes, azahelicenes, and oxahelicenes. Aryl halides such as [4]­thienohelicene **2b** are tolerated and can be used to elongate the helicenes to longer, nonsymmetrical structures through a three-step Mallory–cross-coupling–Mallory sequence. We envision this strategy to also be applicable to aza- and oxahelicenes. While traditional Mallory reactions in batch can be difficult to scale up, our continuous flow methodology enables facile multigram syntheses with throughputs of up to 195 mg/h.

## Supplementary Material



## Data Availability

The data underlying this study are available in the published article, in its Supporting Information, and openly available in nmrXiv at 10.57992/nmrxiv.p146.

## Notes

Experimental procedures, photoflow reactor specifications, structures not pictured in the manuscript, characterization data, and NMR spectra (PDF)

## Notes

A previous version of this manuscript was published as a preprint on *ChemRxiv*.^48^
