# Supplementary material for: Continuous Photoflow Synthesis of Heterohelicenes
Source: Org Lett. 2026 May 5;28(19):5918–22. doi: 10.1021/acs.orglett.6c00559 (PMC13185090; doi:10.1021/acs.orglett.6c00559)

Supporting Information

Continuous photo-flow synthesis of heterohelicenes

Katherine Lyon,[a] Chenyu Pan,[b] Shainthavaan Sathiyalingam,[a,b] Yang Wu,[b] Jayden Matthews,[a] and Jochen R. Brandt[a]*

[a] School of Physical and Chemical Sciences, Queen Mary University of London, 327 Mile End Road, London, E1 4NS, UK.

[b] Department of Chemistry and Molecular Sciences Research Hub, Imperial College London, White City Campus, 82 Wood Lane, London W12 0BZ, UK.

Materials and Methods 7
Figure S1: Diagram of the photochemical flow reactor 7
Note on maintenance for fouled tubing 8
Experimental Data 9
Table S1: Structures not pictured in the main text 9
Table S2: Optimisation of [4]thienohelicene **2a** synthesis 10
Optimisation of the Mallory photocyclisation 10
General Procedures 11
General procedure A: Synthesis of hetero-stilbenes *via* a Wittig reaction 11
General Procedure B: Synthesis of heterohelicenes *via* a Mallory photocyclisation 11
General Procedure C: Synthesis of heterohelicenes *via* a Mallory photocyclisation (catalytic iodine) 11
Synthetic Data 11
Bromo(naphthalen-2-ylmethyl)triphenyl-λ^5-phosphane (**S5a**)[2] 11
(Benzo[*c*]phenanthren-2-ylmethyl)bromotriphenyl- λ^5-phosphane (**S5b**)[2-4] 12
2-(2-(naphthalen-2-yl)vinyl)thiophene (**1a**)[5] 13
2-chloro-5-(2-(naphthalen-2-yl)vinyl)thiophene (**1b**) 13
2-(2-(naphthalen-2-yl)vinyl)furan (**1c**)[5,6] 13
2-chloro-5-(2-(naphthalen-2-yl)vinyl)furan (**1d**) 14
2-(2-(benzo[*c*]phenanthren-2-yl)vinyl)thiophene (**1e**) 14
5-(2-(naphthalen-2-yl)vinyl)benzo[*b*]thiophene (**1f**) 15
2,5-bis(2-(naphthalen-2-yl)vinyl)thiophene (**1g**)[7] 15
3-(2-(naphthalen-2-yl)vinyl)quinoline (**1h**)[8] 15
4-(2-(naphthalen-2-yl)vinyl)thiazole (**1i**) 16
5-(2-(naphthalen-2-yl)vinyl)benzo[*d*]oxazole (**1k**) 16
3-chloro-5-(2-(naphthalen-2-yl)vinyl)pyridine (**1m**) 17
5-(2-(naphthalen-2-yl)vinyl)benzo[*c*][1,2,5]thiadiazole (**1n**)[9] 17
2-chloro-3-(2-(naphthalen-2-yl)vinyl)pyridine (**1o**) 18
5-(2-(benzo[*c*]phenanthren-2-yl)vinyl)benzo[*b*]thiophene (**1p**) 18
3,6-dibromo-9-((2-(trimethylsilyl)ethoxy)methyl)-9*H*-carbazole (**1qa**)[10] 19
9-((2-(trimethylsilyl)ethoxy)methyl)-9*H*-carbazole-3,6-dicarbaldehyde (**1qb**)[11] 19
3,6-bis((*E*)-4-bromostyryl)-9-((2-(trimethylsilyl)ethoxy)methyl)-9*H*-carbazole (**1q**)[12] 20
[4]Thienohelicene (**2a**)[13] 20
2-Chloro[4]thienohelicene (**2b**) 21
[4]Furohelicene (**2c**)[14] 21
2-chloro[4]furohelicene (**2d**) 21
[6]Thienohelicene (**2e**) 21
[5]Thienohelicene (**2f**)[15] 22

[7]Thienohelicene (**2g**)[16] 22
[5]Pyridohelicene (**2h**)[8] 22
[4]Thiazolohelicene (**2i**)[17] and 3-isothiocyanatophenanthrene (**S2i'**) 23
[5]Oxazolohelicene (**2k**) 24
1-chloro[4]pyridohelicene (**2m**) and 3-chloro[4]pyridohelicene (**2m'**) 24
[5]Thiadiazolohelicene (**2n**) and tetrapheno[2,3-*c*][1,2,5]thiadiazole (**2n'**)[9] 24
4-chloro[4]pyridohelicene (**2o**) 25
[7]Thienohelicene (**2p**) and naphtho[1',2':8,9]tetrapheno[2,1-*b*]thiophene (**S2p'**) 25
Dibromo[7]pyrrolohelicene (**2q**) 26
(*E*)-2-styrylphenanthro[3,4-*b*]thiophene (**3a**)[19] 26
[6]Thienohelicene (**4a**) 27
(*E*)-4,4,5,5-tetramethyl-2-(2-(thiophen-2-yl)vinyl)-1,3,2-dioxaborolane (**S5c**)[20] 27
(*Z*)-2-(2-(thiophen-2-yl)vinyl)phenanthro[3,4-*b*]thiophene (**3b**)[21] 28
[6]Dithienohelicene (**4b**) 28
References 28
NMR spectra 31
^{1}H-NMR of Bromo(naphthalen-2-ylmethyl)triphenyl-λ^{5}-phosphane (400 MHz, $CDCl_3$) (**S5a**) 31
^{13}C-NMR of Bromo(naphthalen-2-ylmethyl)triphenyl-λ^{5}-phosphane (101 MHz, $CDCl_3$) (**S5a**) 31
^{31}P-NMR of Bromo(naphthalen-2-ylmethyl)triphenyl-λ^{5}-phosphane (162 MHz, $CDCl_3$) (**S5a**) 32
^{1}H-NMR of (Benzo[c]phenanthren-2-ylmethyl)bromotriphenyl- λ^{5}-phosphane (400 MHz, $CDCl_3$) (**S5b**) 33
^{13}C-NMR of (Benzo[c]phenanthren-2-ylmethyl)bromotriphenyl- λ^{5}-phosphane (101 MHz, $CDCl_3$) (**S5b**) 33
^{31}P-NMR of (Benzo[c]phenanthren-2-ylmethyl)bromotriphenyl- λ^{5}-phosphane (162 MHz, $CDCl_3$) (**S5b**) 34
^{1}H-NMR of (E/Z)-2-(2-(naphthalen-2-yl)vinyl)thiophene (400 MHz, $CDCl_3$) (**1a**) 35
^{13}C-NMR of (E/Z)-2-(2-(naphthalen-2-yl)vinyl)thiophene (101 MHz, $CDCl_3$) (**1a**) 35
^{1}H-NMR of (E)-2-chloro-5-(2-(naphthalen-2-yl)vinyl)thiophene (400 MHz, $CDCl_3$) (**1b**) 36
^{13}C-NMR of (E)-2-chloro-5-(2-(naphthalen-2-yl)vinyl)thiophene (101 MHz, $CDCl_3$) (**1b**) 36
^{1}H-NMR of (E/Z)-2-chloro-5-(2-(naphthalen-2-yl)vinyl)thiophene (400 MHz, $CDCl_3$) (**1b**) 37
^{13}C-NMR of (E/Z)-2-chloro-5-(2-(naphthalen-2-yl)vinyl)thiophene (101 MHz, $CDCl_3$) (**1b**) 37
^{1}H-NMR of (E)-2-(2-(naphthalen-2-yl)vinyl)furan (400 MHz, $CDCl_3$) (**1c**) 38
^{13}C-NMR of (E)-2-(2-(naphthalen-2-yl)vinyl)furan (101 MHz, $CDCl_3$) (**1c**) 38

^{1}H-NMR of (Z)-2-(2-(naphthalen-2-yl)vinyl)furan (400 MHz, $CDCl_3$) (**1c**)....................39
^{13}C-NMR of (Z)-2-(2-(naphthalen-2-yl)vinyl)furan (101 MHz, $CDCl_3$) (**1c**)...................39
^{1}H-NMR of (Z)-2-chloro-5-(2-(naphthalen-2-yl)vinyl)furan (400 MHz, $CDCl_3$) (**1d**).....40
^{13}C-NMR of (Z)-2-chloro-5-(2-(naphthalen-2-yl)vinyl)furan (101 MHz, $CDCl_3$) (**1d**)....40
^{1}H-NMR of (E/Z)-2-chloro-5-(2-(naphthalen-2-yl)vinyl)furan (400 MHz, $CDCl_3$) (**1d**)..41
^{13}C-NMR of (E/Z)-2-chloro-5-(2-(naphthalen-2-yl)vinyl)furan (101 MHz, $CDCl_3$) (**1d**) 41
^{1}H-NMR of (E)-2-(2-(benzo[c]phenanthren-2-yl)vinyl)thiophene (400 MHz, $CDCl_3$) (**1e**) 42
^{13}C-NMR of (E)-2-(2-(benzo[c]phenanthren-2-yl)vinyl)thiophene (101 MHz, $CDCl_3$) (**1e**)....................42
^{1}H-NMR of (E/Z)-2-(2-(benzo[c]phenanthren-2-yl)vinyl)thiophene (400 MHz, $CDCl_3$) (**1e**)....................43
^{13}C-NMR of (E/Z)-2-(2-(benzo[c]phenanthren-2-yl)vinyl)thiophene (101 MHz, $CDCl_3$) (**1e**)....................43
^{1}H-NMR of (Z)-5-(2-(naphthalen-2-yl)vinyl)benzo[b]thiophene (400 MHz, $CDCl_3$) (**1f**) 43
^{13}C-NMR of (Z)-5-(2-(naphthalen-2-yl)vinyl)benzo[b]thiophene (101 MHz, $CDCl_3$) (**1f**) 44
^{1}H-NMR of (E)-5-(2-(naphthalen-2-yl)vinyl)benzo[b]thiophene (400 MHz, THF-d8) (**1f**) 45
^{1}H-NMR of 2,5-bis(2-(naphthalen-2-yl)vinyl)thiophene (400 MHz, THF-d8) (**1g**)........46
^{1}H-NMR of (E)-3-(2-(naphthalen-2-yl)vinyl)quinoline (400 MHz, $CDCl_3$) (**1h**).............47
^{13}C-NMR of (E)-3-(2-(naphthalen-2-yl)vinyl)quinoline (101 MHz, $CDCl_3$) (**1h**)............47
^{1}H-NMR of (Z)-3-(2-(naphthalen-2-yl)vinyl)quinoline (400 MHz, $CDCl_3$) (**1h**) 48
^{13}C-NMR of (Z)-3-(2-(naphthalen-2-yl)vinyl)quinoline (101 MHz, $CDCl_3$) (**1h**)............48
^{1}H-NMR of (E/Z)-4-(2-(naphthalen-2-yl)vinyl)thiazole (400 MHz, $CDCl_3$) (**1i**).............49
^{13}C-NMR of (E/Z)-4-(2-(naphthalen-2-yl)vinyl)thiazole (101 MHz, $CDCl_3$) (**1i**)............49
^{1}H-NMR of (E)-5-(2-(naphthalen-2-yl)vinyl)benzo[d]oxazole (400 MHz, $CDCl_3$) (**1k**) .50
^{13}C-NMR of (E)-5-(2-(naphthalen-2-yl)vinyl)benzo[d]oxazole (101 MHz, $CDCl_3$) (**1k**) 50
^{1}H-NMR of (Z)-5-(2-(naphthalen-2-yl)vinyl)benzo[d]oxazole (400 MHz, $CDCl_3$) (**1k**)..51
^{13}C-NMR of (Z)-5-(2-(naphthalen-2-yl)vinyl)benzo[d]oxazole (101 MHz, $CDCl_3$) (**1k**) 51
^{1}H-NMR of (E)-3-chloro-5-(2-(naphthalen-2-yl)vinyl)pyridine (400 MHz, $CDCl_3$) (**1m**)52
^{13}C-NMR of (E)-3-chloro-5-(2-(naphthalen-2-yl)vinyl)pyridine (101 MHz, $CDCl_3$) (**1m**) 52
^{1}H-NMR of (Z)-3-chloro-5-(2-(naphthalen-2-yl)vinyl)pyridine (400 MHz, $CDCl_3$) (**1m**) 53
^{13}C-NMR of (Z)-3-chloro-5-(2-(naphthalen-2-yl)vinyl)pyridine (101 MHz, $CDCl_3$) (**1m**) 53
^{1}H-NMR of (E)-5-(2-(naphthalen-2-yl)vinyl)benzo[c][1,2,5]thiadiazole (400 MHz, $CDCl_3$) (**1n**)....................54

^{13}C-NMR of (E)-5-(2-(naphthalen-2-yl)vinyl)benzo[c][1,2,5]thiadiazole (101 MHz, $CDCl_3$) (**1n**)....54

^{1}H-NMR of (E/Z)-5-(2-(naphthalen-2-yl)vinyl)benzo[c][1,2,5]thiadiazole (400 MHz, $CDCl_3$) (**1n**)....55

^{13}C-NMR of (E/Z)-5-(2-(naphthalen-2-yl)vinyl)benzo[c][1,2,5]thiadiazole (101 MHz, $CDCl_3$) (**1n**)....56

^{1}H-NMR of (Z)-2-chloro-3-(2-(naphthalen-2-yl)vinyl)pyridine (400 MHz, $CDCl_3$) (**1o**).57

^{13}C-NMR of (Z)-2-chloro-3-(2-(naphthalen-2-yl)vinyl)pyridine (101 MHz, $CDCl_3$) (**1o**)57

^{1}H-NMR of (E)-2-chloro-3-(2-(naphthalen-2-yl)vinyl)pyridine (400 MHz, $CDCl_3$) (**1o**).58

^{13}C-NMR of (E)-2-chloro-3-(2-(naphthalen-2-yl)vinyl)pyridine (101 MHz, $CDCl_3$) (**1o**)58

^{1}H-NMR of 5-(2-(benzo[c]phenanthren-2-yl)vinyl)benzo[b]thiophene (400 MHz, $CDCl_3$) (**1p**)....59

^{13}C-NMR of 5-(2-(benzo[c]phenanthren-2-yl)vinyl)benzo[b]thiophene (101 MHz, $CDCl_3$) (**1p**)....59

^{1}H-NMR of 3,6-dibromo-9-((2-(trimethylsilyl)ethoxy)methyl)-9H-carbazole (400 MHz, $CDCl_3$) (**1qa**)....60

^{13}C-NMR of 3,6-dibromo-9-((2-(trimethylsilyl)ethoxy)methyl)-9H-carbazole (101 MHz, $CDCl_3$) (**1qa**)....60

^{1}H-NMR of 9-((2-(trimethylsilyl)ethoxy)methyl)-9H-carbazole-3,6-dicarbaldehyde (400 MHz, $CDCl_3$) (**1qb**)....61

^{13}C-NMR of 9-((2-(trimethylsilyl)ethoxy)methyl)-9H-carbazole-3,6-dicarbaldehyde (101 MHz, $CDCl_3$) (**1qb**)....61

^{1}H-NMR of 3,6-bis((E)-4-bromostyryl)-9-((2-(trimethylsilyl)ethoxy)methyl)-9H-carbazole (400 MHz, $CDCl_3$) (**1q**)....62

^{13}C-NMR of 3,6-bis((E)-4-bromostyryl)-9-((2-(trimethylsilyl)ethoxy)methyl)-9H-carbazole (101 MHz, $CDCl_3$) (**1q**)....62

^{1}H-NMR of [4]thienohelicene (400 MHz, $CDCl_3$) (**2a**)....63

^{13}C-NMR of [4]thienohelicene (101 MHz, $CDCl_3$) (**2a**)....63

^{1}H-NMR of 2-chloro[4]thienohelicene (400 MHz, $CDCl_3$) (**2b**)....64

^{13}C-NMR of 2-chloro[4]thienohelicene (101 MHz, $CDCl_3$) (**2b**)....64

^{1}H-NMR of [4]furohelicene (400 MHz, $CDCl_3$) (**2c**)....65

^{13}C-NMR of [4]furohelicene (101 MHz, $CDCl_3$) (**2c**)....65

^{1}H-NMR of 2-chloro[4]furohelicene (400 MHz, $CDCl_3$) (**2d**)....66

^{13}C-NMR of 2-chloro[4]furohelicene (101 MHz, $CDCl_3$) (**2d**)....66

^{1}H-NMR of [6]thienohelicene (400 MHz, $CDCl_3$) (**2e**)....67

^{13}C-NMR of [6]thienohelicene (101 MHz, $CDCl_3$) (**2e**)....67

^{1}H-NMR of [5]thienohelicene (400 MHz, $CDCl_3$) (**2f**)....68

^{13}C-NMR of [5]thienohelicene (101 MHz, $CDCl_3$) (**2f**)....68

^{1}H-NMR of [7]thienohelicene (400 MHz, $CDCl_3$) (**2g**)....69

^{13}C-NMR of [7]thienohelicene (101 MHz, $CDCl_3$) (**2g**)....69

^{1}H-NMR of [5]pyridohelicene (400 MHz, $CDCl_3$) (**2h**)....70
^{13}C-NMR of [5]pyridohelicene (101 MHz, $CDCl_3$) (**2h**)....70
^{1}H-NMR of [4]thiazolohelicene (400 MHz, $CDCl_3$) (**2i**)....71
^{13}C-NMR of [4]thiazolohelicene (101 MHz, $CDCl_3$) (**2i**)....71
^{1}H-NMR of 3-isothiocyanatophenanthrene (400 MHz, $CDCl_3$) (**S2i'**)....72
^{13}C-NMR of 3-isothiocyanatophenanthrene (101 MHz, $CDCl_3$) (**S2i'**)....72
^{1}H-NMR of [5]oxazolohelicene (400 MHz, $CDCl_3$) (**2k**)....73
^{13}C-NMR of [5]oxazolohelicene (101 MHz, $CDCl_3$) (**2k**)....73
^{1}H-NMR of 1-chloro[4]pyridohelicene (400 MHz, $CDCl_3$) (**2m**)....74
^{13}C-NMR of 1-chloro[4]pyridohelicene (101 MHz, $CDCl_3$) (**2m**)....74
^{1}H-NMR of 3-chloro[4]pyridohelicene (400 MHz, $CDCl_3$) (**2m'**)....75
^{13}C-NMR of 3-chloro[4]pyridohelicene (101 MHz, $CDCl_3$) (**2m'**)....75
^{1}H-NMR of [5]thiadiazolohelicene (400 MHz, $CDCl_3$) (**2n**)....76
^{13}C-NMR of [5]thiadiazolohelicene (101 MHz, $CDCl_3$) (**2n**)....76
^{1}H-NMR of tetrapheno[2,3-c][1,2,5]thiadiazole (400 MHz, $CDCl_3$) (**2n'**)....77
^{13}C-NMR of tetrapheno[2,3-c][1,2,5]thiadiazole (101 MHz, $CDCl_3$) (**2n'**)....77
^{1}H-NMR of 4-chloro[4]pyridohelicene (400 MHz, $CDCl_3$) (**2o**)....78
^{13}C-NMR of 4-chloro[4]pyridohelicene (400 MHz, $CDCl_3$) (**2o**)....78
^{1}H-NMR of [7]thienohelicene (400 MHz, $CDCl_3$) (**2p**)....79
^{13}C-NMR of [7]thienohelicene (101 MHz, $CDCl_3$) (**2p**)....79
^{1}H-NMR of naphtho[1',2':8,9]tetrapheno[2,1-b]thiophene (400 MHz, $CDCl_3$) (**S2p'**)...80
^{13}C-NMR of naphtho[1',2':8,9]tetrapheno[2,1-b]thiophene (101 MHz, $CDCl_3$) (**S2p'**).80
^{1}H-NMR of dibromo[7]pyrrolohelicene (400 MHz, $CDCl_3$) (**2q**)....81
^{13}C-NMR of dibromo[7]pyrrolohelicene (101 MHz, $CDCl_3$) (**2q**)....81
^{1}H-NMR of (E)-2-styrylphenanthro[3,4-b]thiophene (400 MHz, $CDCl_3$) (**3a**)....82
^{13}C-NMR of (E)-2-styrylphenanthro[3,4-b]thiophene (101 MHz, $CDCl_3$) (**3a**)....82
^{1}H-NMR of [6]thienohelicene (400 MHz, $CDCl_3$) (**4a**)....83
^{13}C-NMR of [6]thienohelicene (101 MHz, $CDCl_3$) (**4a**)....83
^{1}H-NMR of (E)-4,4,5,5-tetramethyl-2-(2-(thiophen-2-yl)vinyl)-1,3,2-dioxaborolane (400 MHz, $CDCl_3$) (**S5c**)....84
^{13}C-NMR of (E)-4,4,5,5-tetramethyl-2-(2-(thiophen-2-yl)vinyl)-1,3,2-dioxaborolane (101 MHz, $CDCl_3$) (**S5c**)....84
^{1}H-NMR of (E)-2-(2-(thiophen-2-yl)vinyl)phenanthro[3,4-b]thiophene (400 MHz, $CDCl_3$) (**3b**)....85
^{13}C-NMR of (E)-2-(2-(thiophen-2-yl)vinyl)phenanthro[3,4-b]thiophene (101 MHz, $CDCl_3$) (**3b**)....85
^{1}H-NMR of [6]Dithienohelicene (400 MHz, $CDCl_3$) (**4b**)....86
^{13}C-NMR of [6]Dithienohelicene (400 MHz, $CDCl_3$) (**4b**)....86

Materials and Methods

Reagents were purchased and used without further purification (unless otherwise stated) from Sigma-Aldrich, Fluorochem, Apollo Scientific, Thermo-Fisher or TCI. Copper powder (625 mesh, 0.50-1.5 micron, 99% (metals basis)) was purchased from Thermo-Fisher (product number 041205.30). 'Dry' solvents were purchased from Thermo-Fisher in AcroSeal™ packaging. Flash chromatographic purification of products was carried out using Fluorochem silica gel (60 Å, 40-63 µm) using a forced flow of eluent at 0.3–0.5 bar pressure. [1] Further purifications were performed on a JAI LaboACE LC-5060 Plus II recycling HPLC with JAIGEL-HR Plus 2 + 2.5HR size exclusion (gel permeation chromatography, GPC) columns in sequence with chloroform as an eluent. Thin layer chromatography was performed using Merck pre-coated silica gel GF254 and was visualised under UV light (254 nm and 365 nm). The ^{1}H NMR, ^{13}C NMR and ^{31}P NMR spectra were recorded in deuterated chloroform (purchased from Cambridge Isotope Laboratories) or deuterated THF (purchased from Fluorochem) on Bruker AVANCE III HD at 400 MHz, 101 MHz and 162 MHz respectively. Chemical shifts (δ) were given in parts per million (ppm) and coupling constants (J) were given in Hertz (Hz). TMS is used as reference for the NMR peaks. Concentration under reduced pressure was performed by rotary evaporation at 40-50 °C at the appropriate pressure. Purified compounds were further dried under high vacuum (0.1–0.01 mbar). HRMS was performed on a Waters Synapt G2Si HDMS with a TOF mass analyser or in collaboration with NMSF at Swansea University. Melting ranges were recorded on a Cole-Parmer® MP-800 Series with the ramping set to 5 °C/min. Photo-flow reactions were performed in air on a Vapourtec E-Series system with a UV-150 photochemical reactor (7cm coil diameter, fluoropolymer tubing, tubing ID 1.3 mm) and high-power 150 W LED lamp (365 nm) or a 60 W LED lamp (365 nm) that is placed inside the tubing coil (lamp to wall distance 22 mm). Without external cooling, the temperature inside the photoreactor reaches approximately 55 and 40 °C, using the 150 and 60 W lamps, respectively.

Figure S1: Diagram of the photochemical flow reactor

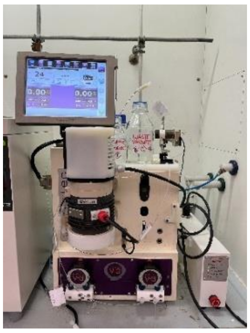

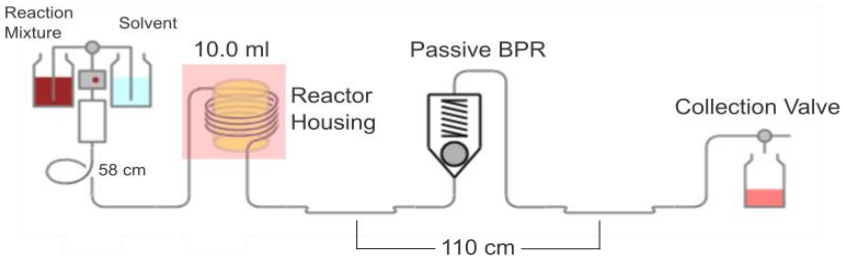

Note on maintenance for fouled tubing

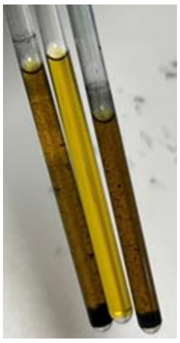

After gram-scale reactions or reactions at temperatures above 55°C, we began to see yellow/brown sediment build up on the inside of the tubing. To maintain consistent light penetration inside the reactor, it is important to clean any significant build-up inside the tubing. We accomplished this by flushing the tubing with toluene, THF and aqueous 15% sodium thiosulfate solution alternately until the tubing becomes clear. Users should ensure the compatibility of their flow reactor with the reaction and cleaning conditions before repeating the work described here.

Figure: NMR samples of the crude reaction mixture of **2o** performed under Conditions A (left and right tubes) and Conditions B (centre tube). The left and right tubes show a blue-black precipitate which can occur when nitrogen-containing substrates are used with Conditions A.

Experimental Data

Table S1: Structures not pictured in the main text

Heterostilbenes and precursors

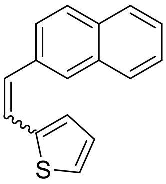

1a

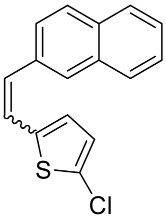

1b

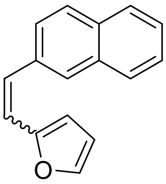

1c

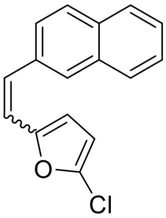

1d

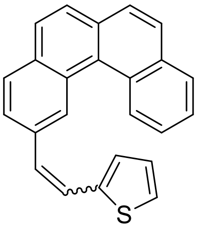

1e

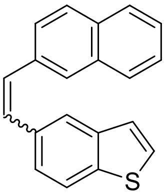

1f

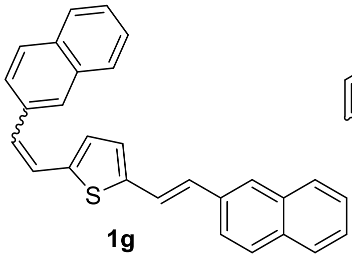

1g

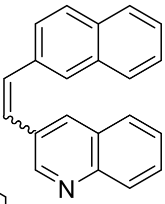

1h

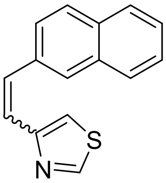

1i

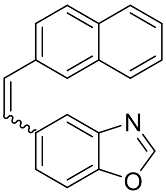

1k

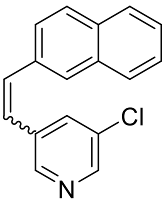

1m

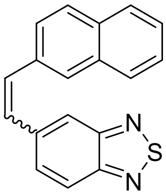

1n

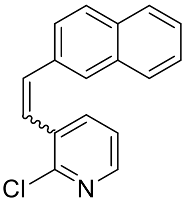

1o

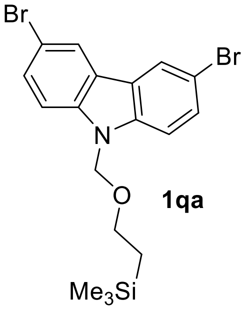

1qa

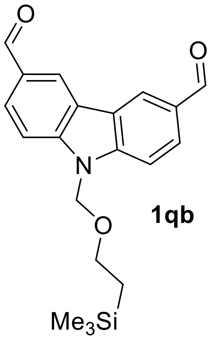

1qb

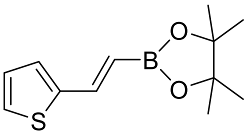

S5c

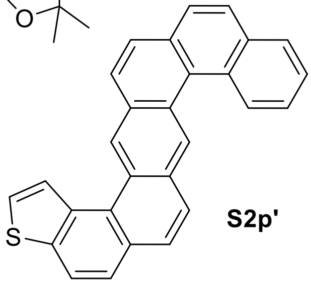

S2p'

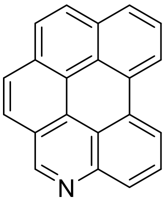

S2h'

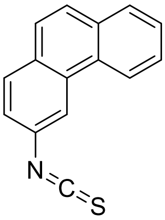

S2i'

Wittig Salts

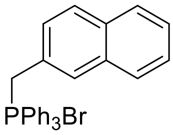

S5a

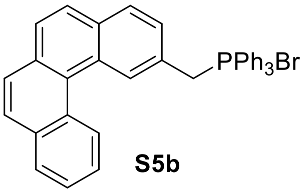

S5b

NHC-Pd(II)-Im

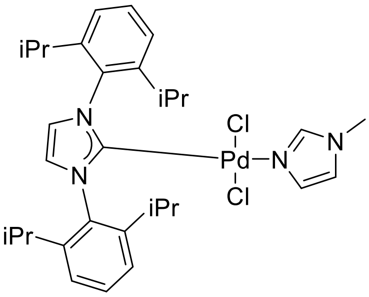

Table S2: Optimisation of [4]thienohelicene **2a** synthesis

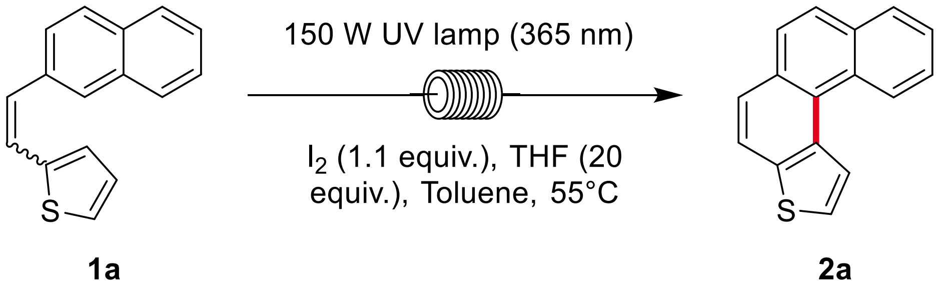

Entry	Concentration (mM)	Flow rate (mL/min)	Residence time (s)	NMR yield (%)
1	2	8	75	99
**2**	**2**	**6**	**100**	**100**
3	2	5	120	97
4	2	4	150	>100
5	2	3	200	95
6	2	2	300	91
7	4	6	100	96
8	4	2	300	87
**9**	2	6	100	0[a]

The estimated error of the NMR yields is 2-3%. [a]Experiment was run with the lamp turned off.

Optimisation of the Mallory photocyclisation

A stock solution of stilbene **1a** (1.00 equiv.), iodine (1.10 equiv.) and THF (20.0 equiv.) was prepared in toluene to a concentration of 2.0 or 4.0 mM. For each test run, 20 mL of stock solution (corresponding to 0.04 mmol or 0.08 mmol of **1a** for 2.0 mM and 4.0 mM concentrations respectively) was run through a Vapourtec E-series flow reactor fitted with a 150 W 365 nm LED lamp at 55 °C with a reactor coil volume of 10 mL. The collected crude mixture was washed with 2.0 mL each of $Na_2S_2O_3$ (15% in water) and brine. The crude product was dried with $MgSO_4$ and concentrated *in vacuo*. Trimethoxybenzene (1.00 equiv.) was added to the crude mixture as an internal standard. The proton NMR spectrum was used to determine an approximate NMR yield for the product **2a** by comparing the integration of the product peaks to the integration of the internal standard. Errors from overlapping peaks and weighing the internal standard may result in yields over 100% when the 'actual' yield is close to complete conversion.

General Procedures

General procedure A: Synthesis of hetero-stilbenes *via* a Wittig reaction

Wittig salt **S5a** or **S5b** (1.00 equiv.) and a (hetero)aryl aldehyde (1.00 equiv.) were dissolved in dry MeOH (0.1 M), and the reaction solution was sparged by bubbling nitrogen through the reaction mixture for 10 min. KO*t*-Bu (3.00 equiv.) was added and the reaction mixture was refluxed for 2 h or until completion (determined by TLC). The mixture was left to cool to room temperature and then was quenched with distilled water (10 mL). The crude product was extracted with DCM (3 x 10 mL) and the combined organic layers were washed with brine, dried under $MgSO_4$ (Na_2SO_4 for nitrogen containing compounds) and concentrated *in vacuo*. The crude product was purified by column chromatography.

General Procedure B: Synthesis of heterohelicenes *via* a Mallory photocyclisation

Stilbene derivative (1.00 equiv.), iodine (1.10 equiv.) and THF (20.0 equiv.) were dissolved in toluene or toluene:acetonitrile (1:1) to a concentration of 2.0 mM. The reaction was run through a Vapourtec E-series flow reactor fitted with a 150 W 365 nm LED lamp at 55 °C and 6 mL/min with a reactor coil volume of 10 mL. The collected reaction mixture was washed with 10 mL each of NaOH (1.0 M) (only for nitrogen-containing compounds), $Na_2S_2O_3$ solution (15% in water) and brine. The crude product was dried with $MgSO_4$ or Na_2SO_4 and concentrated *in vacuo* and purified by column chromatography.

General Procedure C: Synthesis of heterohelicenes *via* a Mallory photocyclisation (catalytic iodine)

Stilbene derivative (1.00 equiv.) and iodine (0.10 equiv.) were dissolved in toluene:THF (9:1) to a concentration of 2.0 mM. The reaction was run through a Vapourtec E-series flow reactor fitted with a 150 W 365 nm LED lamp at 55 °C and 6 mL/min with a reactor coil length of 10 mL. The collected reaction mixture was washed with 10 mL each of NaOH (1.0 M) (only for nitrogen-containing compounds), $Na_2S_2O_3$ solution (15% in water) and brine. The crude product was dried with $MgSO_4$ or Na_2SO_4 and concentrated *in vacuo* and purified by column chromatography.

Synthetic Data

Bromo(naphthalen-2-ylmethyl)triphenyl-λ^5-phosphane (**S5a**)[2]

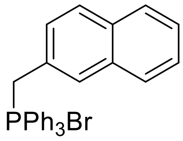

2-(Bromomethyl)naphthalene (4.86 g, 22.0 mmol, 1.00 equiv.) and triphenylphosphine (8.66 g, 33.0 mmol, 1.50 equiv.) were dissolved in dry toluene (66 mL). The reaction mixture was sparged with nitrogen, then refluxed for 18 h. The reaction mixture was left to cool to room temperature, and the precipitate was collected by vacuum filtration and washed with toluene to give the product **S5a** as an off-white solid in 90% yield (9.52 g, 19.7 mmol).

^{1}H NMR (400 MHz, $CDCl_3$) δ 7.71 – 7.60 (m, 10H), 7.56 – 7.49 (m, 6H), 7.48 – 7.42 (m, 3H), 7.38 – 7.32 (m, 1H), 7.32 – 7.27 (m, 1H), 7.11 – 7.06 (m, 1H), 5.43 (d, J = 14.5 Hz, 2H). **^{13}C NMR (101 MHz, CDCl3)** δ 134.9 (d, J = 3.0 Hz), 134.3 (d, J = 9.8 Hz), 132.8 (d, J = 3.4 Hz), 132.5 (d, J = 2.8 Hz), 131.1 (d, J = 7.3 Hz), 130.0 (d, J = 12.5 Hz), 128.4 (d, J = 4.2 Hz), 128.3 (d, J = 2.6 Hz), 127.7 (d, J = 1.3 Hz), 127.4 (d, J = 1.7 Hz), 126.5 (d, J = 1.5 Hz), 126.3 (d, J = 1.0 Hz), 124.4 (d, J = 9.0 Hz), 117.6 (d, J = 85.7 Hz), 30.9 (d, J = 46.8 Hz). **^{31}P NMR (162 MHz, $CDCl_3$)** δ 22.81.

HRMS (ASAP, QTOF) m/z: $[M+H]^+$ calcd. for $C_{29}H_{24}BrP$ 483.0877; Found 483.0868.

The procedure was adapted from a previous report.

(Benzo[*c*]phenanthren-2-ylmethyl)bromotriphenyl- λ^5-phosphane (**S5b**)[2-4]

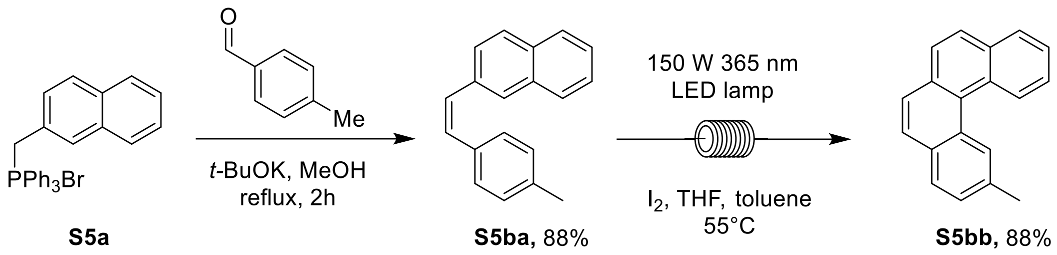

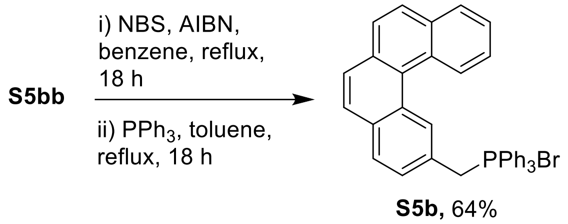

4-methylbenzaldehyde was subjected to general procedure A on a scale of 10.0 mmol and purified by column chromatography (pentane) to give stilbene **S5ba** as a white solid in 88% yield (2.15 g, 8.76 mmol). **S5ba** was carried forward and subjected to general procedure B and purified by column chromatography (2.5:97.5 EtOAc:pentane) to give **S5bb** as a white solid in 88% yield (1.77 g, 7.30 mmol).

[4]-Methylhelicene **S5bb** (1.77 g, 7.30 mmol, 1 equiv.), AIBN (120 mg, 0.73 mmol, 0.1 equiv.) and NBS (1.37 g, 7.67 mmol, 1.05 equiv.) were dissolved in benzene (70 mL) and refluxed under nitrogen for 18 h. The reaction mixture was filtered to remove solids, washed with $Na_2S_2O_3$ solution (15% in water) and water, dried with Na_2SO_4 and concentrated *in vacuo*. The unpurified crude was dissolved in dry toluene (20 mL) alongside triphenylphosphine (2.87 g, 11.0 mmol, 1.5 equiv.), sparged with nitrogen and refluxed for 18 h. The reaction mixture was left to cool to room temperature and the precipitate was collected by vacuum filtration and washed with toluene to give product **S5b** as a light brown solid in 64% yield (2.73 g, 4.68 mmol).

^{1}H NMR (400 MHz, $CDCl_3$) δ 8.42 (s, 1H), 8.12 (d, J = 8.4 Hz, 1H), 7.84 (dd, J = 7.9, 1.2 Hz, 1H), 7.76 (d, J = 8.5 Hz, 1H), 7.68 – 7.56 (m, 11H), 7.55 – 7.43 (m, 9H), 7.42 – 7.37 (m, 1H), 7.05 (d, J = 8.3 Hz, 1H), 5.35 (d, J = 14.4 Hz, 2H). **^{13}C NMR (101 MHz, $CDCl_3$)** δ 134.9 (d, J = 2.9 Hz), 134.2 (d, J = 9.8 Hz), 133.1 (s), 132.5 (d, J = 3.2 Hz), 131.0 (d, J = 1.0 Hz), 130.1 (s, J = 3.7 Hz), 130.1 (d, J = 12.5 Hz), 129.6 (s), 129.6 (s), 128.9 (d, J = 2.8 Hz), 128.2 (s), 128.0 (d, J = 4.4 Hz), 127.6 (s), 127.4 (s), 126.9 (s), 126.9 (d, J = 1.7 Hz), 126.6 (s), 126.4 (d, J = 0.9 Hz), 125.9 (s), 124.9 (d, J = 9.0 Hz), 118.1 (s), 117.2 (s), 30.9 (d, J = 46.4 Hz). **^{31}P NMR (162 MHz, $CDCl_3$)** δ 23.10.

HRMS (ASAP, QTOF) m/z: $[M-Br]^+$ calcd. for $C_{37}H_{28}P$ 503.1929; Found 503.1927.

Melting point: 296-305 °C.

The procedure was adapted from a previous report.[2] Proton NMR spectra of compounds **S5ba**,[3] and **S5bb**[4] matched those reported in literature.

2-(2-(naphthalen-2-yl)vinyl)thiophene (1a)[5]

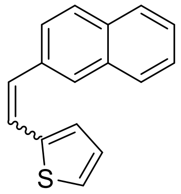

Thiophene-2-carbaldehyde was subjected to general procedure A on a scale of 5.00 mmol and purified by column chromatography (pentane) to give product **1a** as a light brown solid in 42% yield (498 mg, 2.11 mmol).

Mixture (1:1.6 *E:Z*): **^{1}H NMR (400 MHz, $CDCl_3$)** δ 7.90 (s, 1.6H), 7.88 – 7.79 (m, 9.0H), 7.70 (dd, J = 8.6, 1.6 Hz, 1.0H), 7.53 – 7.44 (m, 7.0H), 7.39 (d, J = 16.1 Hz, 1.0H), 7.24 (d, J = 5.1 Hz, 1.0H), 7.16 – 7.10 (m, 2.0H), 7.09 (dd, J = 5.1, 1.2 Hz, 1.6H), 7.05 (dd, J = 5.1, 3.6 Hz, 1.0H), 7.03 (d, J = 3.6 Hz, 1.5H), 6.91 (dd, J = 5.1, 3.6 Hz, 1.6H), 6.81 (d, J = 12.0 Hz, 1.6H), 6.74 (d, J = 12.0 Hz, 1.6H). **^{13}C NMR (101 MHz, $CDCl_3$)** δ 143.1, 140.0, 135.0, 134.6, 133.9, 133.7, 133.2, 133.0, 131.8, 128.9, 128.6, 128.5, 128.2, 128.1, 128.1, 128.1, 127.9, 127.9, 127.8, 127.0, 126.6, 126.6, 126.5, 126.3, 126.2, 126.2, 126.1, 125.7, 124.6, 123.8, 123.4, 122.3.

HRMS (ASAP, Orbitrap) m/z: $[M+H]^+$ calcd. for $C_{16}H_{13}S$ 237.0732; Found 237.0739.

The ^{1}H NMR peaks of ***(E)*-1a** matched those of the pure (*E*)-isomer reported in the literature.

2-chloro-5-(2-(naphthalen-2-yl)vinyl)thiophene (1b)

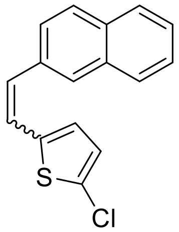

5-Chloro-2-thiophenecarboxaldehyde was subjected to general procedure A on a scale of 10.0 mmol and purified by column chromatography (hexane) to give product **1b** as a light yellow (*E*) or oily brown (*E/Z* mix) solid in 64% yield (1.73 g, 6.39 mmol).

E isomer: **^{1}H NMR (400 MHz, $CDCl_3$)** δ 7.85 – 7.78 (m, 4H), 7.64 (dd, J = 8.5, 1.8 Hz, 1H), 7.52 – 7.42 (m, 2H), 7.20 (d, J = 16.1 Hz, 1H), 6.96 (d, J = 16.1 Hz, 1H), 6.86 (d, J = 3.9 Hz, 1H), 6.84 (d, J = 3.8 Hz, 1H). **^{13}C NMR (101 MHz, $CDCl_3$)** δ 141.8, 134.1, 133.7, 133.1, 128.8, 128.6, 128.4, 128.0, 127.7, 126.8, 126.6, 126.5, 126.1, 125.5, 123.1, 121.7.

Mixture (1:1.6 *E:Z*): **^{1}H NMR (400 MHz, $CDCl_3$** δ 7.90 – 7.79 (m, 10.4H), 7.66 (dd, J = 8.5, 1.8 Hz, 1.0H), 7.55 – 7.43 (m, 6.8H), 7.22 (d, J = 16.1 Hz, 1.0H), 6.97 (d, J = 16.1 Hz, 1.0H), 6.86 (q, J = 3.9 Hz, 2.0H), 6.79 (d, J = 3.9 Hz, 1.6H), 6.72 (dd, J = 7.9, 4.0 Hz, 3.1H), 6.65 (d, J = 11.9 Hz, 1.6H). **^{13}C NMR (101 MHz, CDCl3)** δ 141.9, 138.7, 134.5, 134.2, 133.8, 133.6, 133.2, 133.0, 130.01, 129.1, 128.9, 128.7, 128.6, 128.5, 128.2, 128.1, 128.1, 128.0, 127.9, 127.9, 126.9, 126.8, 126.7, 126.6, 126.3, 126.2, 125.6, 125.5, 123.6, 123.3, 121.8.

HRMS (ASAP, QTOF) m/z: $[M]^{+\cdot}$ calcd. for $C_{16}H_{11}ClS$ 270.0270; Found 270.0262.

Melting range: 131-134 °C (*E*-isomer).

2-(2-(naphthalen-2-yl)vinyl)furan (1c)[5,6]

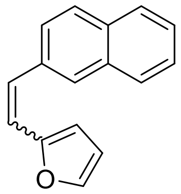

Furfural was subjected to general procedure A on a scale of 3.00 mmol and purified by column chromatography (pentane) to give product **1c** as an *E/Z* mixture in 83% yield (550mg, 2.50 mmol). In a separate experiment, *E* and *Z* isomers (both pale, beige solids) were separated to provide pure analytical data.

E isomer: **^{1}H NMR (400 MHz, $CDCl_3$)** δ 7.86 – 7.76 (m, 4H), 7.67 (dd, J = 8.6, 1.5 Hz, 1H), 7.50 – 7.41 (m, 3H), 7.21 (d, J = 16.2 Hz, 1H), 7.03 (d, J = 16.2 Hz, 1H), 6.45 (dd, J = 3.2, 1.8 Hz, 1H), 6.40 (d, J = 3.2 Hz, 1H). **^{13}C NMR (101 MHz, $CDCl_3$)** δ 153.5, 142.4, 134.7, 133.9, 133.2, 128.5, 128.1, 127.8, 127.4, 126.6, 126.5, 126.0, 123.4, 117.0, 111.9, 108.9.

Z isomer: **^{1}H NMR (400 MHz, $CDCl_3$)** δ 7.91 (s, 1H), 7.85 – 7.77 (m, 3H), 7.58 (dd, J = 8.5, 1.4 Hz, 1H), 7.51 – 7.43 (m, 2H), 7.31 (d, J = 1.2 Hz, 1H), 6.64 (d, J = 12.6 Hz, 1H), 6.46 (d, J = 12.6 Hz, 1H), 6.35 –

6.25 (m, 2H). **^{13}C NMR (101 MHz, $CDCl_3$)** δ 152.3, 141.8, 135.1, 133.5, 132.9, 128.2, 128.1, 127.9, 127.8, 127.6, 127.0, 126.2, 126.1, 118.5, 111.4, 110.3.

HRMS (ASAP, QTOF) m/z: $[M+H]^+$ calcd. for $C_{16}H_{13}O$ 221.0966; Found 221.0964.

NMR spectra of **(*E*)-1c**[5] and **(*Z*)-1c**[6] matched those reported in literature.

2-chloro-5-(2-(naphthalen-2-yl)vinyl)furan (1d)

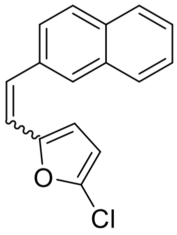

5-Chloro-2-furaldehyde was subjected to general procedure A on a scale of 4.00 mmol and purified by column chromatography (5:95 EtOAc:pentane) to product **1d** as a beige solid in 69% yield (705 mg, 2.77 mmol).

Mixture (1:1.3 *E:Z*): **^{1}H NMR (400 MHz, $CDCl_3$)** δ 7.91 (s, 1.3H), 7.86 – 7.77 (m, 8.0H), 7.65 (dd, J = 8.6, 1.7 Hz, 1.0H), 7.57 (dd, J = 8.6, 1.7 Hz, 1.3H), 7.51 – 7.42 (m, 4.6H), 7.18 (d, J = 16.2 Hz, 1.0H), 6.90 (d, J = 16.2 Hz, 1.0H), 6.63 (d, J = 12.6 Hz, 1.3H), 6.39 – 6.34 (m, 2.3H), 6.25 – 6.21 (m, 2.3H), 6.08 (d, J = 3.4 Hz, 1.3H). **^{13}C NMR (101 MHz, $CDCl_3$)** δ 153.1, 151.9, 136.3, 135.6, 134.6, 134.3, 133.8, 133.5, 133.2, 132.9, 128.6, 128.5, 128.2, 127.8, 127.8, 127.8, 127.7, 126.9, 126.7, 126.6, 126.3, 126.2, 126.2, 123.3, 117.6, 115.9, 112.0, 110.6, 108.5, 108.1.

Z isomer: **^{1}H NMR (400 MHz, $CDCl_3$)** δ 7.91 (s, 1H), 7.85 – 7.78 (m, 3H), 7.57 (dd, *J* = 8.5, 1.5 Hz, 1H), 7.51 – 7.45 (m, 2H), 6.63 (d, *J* = 12.6 Hz, 1H), 6.40 – 6.33 (m, 1H), 6.23 (d, *J* = 3.4 Hz, 1H), 6.08 (d, *J* = 3.4 Hz, 1H). **^{13}C NMR (101 MHz, $CDCl_3$)** δ 151.9, 135.6, 134.6, 133.5, 133.0, 128.6, 128.2, 127.8, 127.8, 127.8, 126.7, 126.3, 126.2, 117.7, 112.0, 108.1.

HRMS (ASAP, QTOF) m/z: $[M]^{+\cdot}$ calcd. for $C_{16}H_{11}ClO$ 254.0498; Found 254.0495.

2-(2-(benzo[*c*]phenanthren-2-yl)vinyl)thiophene (1e)

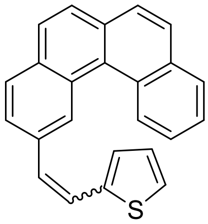

Thiophene-2-carbaldehyde was subjected to general procedure A (Wittig salt **S5b** was used instead of **S5a**) on a scale of 2.00 mmol and purified by column chromatography (1:99 EtOAc:hexane) to give product **1e** as a yellow solid in 78% yield (526 mg, 1.56 mmol).

E isomer: **^{1}H NMR (400 MHz, $CDCl_3$)** δ 9.15 (d, J = 8.5 Hz, 1H), 9.11 (s, 1H), 8.04 (d, J = 7.9 Hz, 1H), 7.99 (d, J = 8.4 Hz, 1H), 7.91 (d, J = 8.5 Hz, 1H), 7.87 (d, J = 8.5 Hz, 1H), 7.81 (t, J = 8.5 Hz, 3H), 7.75 (t, J = 7.1 Hz, 1H), 7.66 (t, J = 7.4 Hz, 1H), 7.43 (d, J = 16.0 Hz, 1H), 7.25 – 7.19 (m, 2H), 7.16 (d, J = 3.4 Hz, 1H), 7.05 (dd, J = 5.0, 3.6 Hz, 1H). **^{13}C NMR (101 MHz, $CDCl_3$)** δ 143.1, 135.0, 133.7, 133.2, 131.5, 130.8, 130.5, 129.1, 129.1, 128.8, 128.0, 127.9, 127.8, 127.5, 127.3, 127.0, 127.0, 127.0, 126.5, 126.4, 126.1, 124.7, 123.2, 122.4.

Mixture (1:1.1 *E:Z*): **1H NMR (400 MHz, $CDCl_3$)** δ 9.17 (d, J = 8.5 Hz, 1H), 9.13 (s, 1.1H), 9.12 (s, 1H), 8.89 (d, J = 8.4 Hz, 1.1H), 8.06 (dd, J = 7.9, 1.1 Hz, 1H), 8.03 – 7.99 (m, 2.2H), 7.98 (d, J = 8.4 Hz, 1H), 7.94 – 7.88 (m, 3H), 7.87 (d, J = 8.5 Hz, 1.1H), 7.85 – 7.75 (m, 6H), 7.71 – 7.65 (m, 2.1H), 7.63 – 7.58 (m, 1.1H), 7.55 (ddd, J = 8.3, 7.0, 1.5 Hz, 1.1H), 7.45 (d, J = 16.0 Hz, 1H), 7.29 – 7.21 (m, 1H), 7.17 (d, J = 3.6 Hz, 1.0H), 7.15 (d, J = 5.1 Hz, 1.1H), 7.12 (dd, J = 3.6, 0.9 Hz, 1.1H), 7.08 (dd, J = 5.0, 3.6 Hz, 1H), 6.96 (dd, J = 5.0, 3.6 Hz, 1.1H), 6.87 (s, 2.2H). **^{13}C NMR (101 MHz, $CDCl_3$)** δ 143.1, 140.0, 135.4, 134.9, 133.6, 133.5, 133.1, 132.9, 131.5, 131.3, 130.7, 130.6, 130.4, 130.4, 130.0, 129.1, 129.0, 128.8, 128.7, 128.5, 128.4, 128.2, 127.9, 127.8, 127.7, 127.7, 127.5, 127.4, 127.3, 127.2, 127.1, 127.0, 127.0, 127.0, 126.9, 126.8, 126.7, 126.4, 126.4, 126.3, 126.0, 125.9, 125.7, 124.6, 123.8, 123.1, 122.3.

HRMS (ASAP, QTOF) m/z: $[M+H]^+$ calcd. for $C_{24}H_{16}S$ 337.1051; Found 337.1043.

Melting point: 127-133 °C (*E*-isomer).

5-(2-(naphthalen-2-yl)vinyl)benzo[b]thiophene (1f)

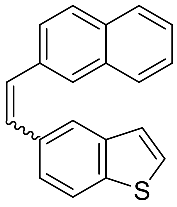

Benzo[*b*]thiophene-5-carbaldehyde was subjected to general procedure A on a scale of 6.00 mmol and purified by column chromatography (2.5:97.5 EtOAc:hexane) to give the *E*-isomer as a white, powdery solid in 34% yield (578 mg, 2.04 mmol), the *Z*-isomer as a white crystalline solid in 3% yield (57.3 mg, 0.180 mmol) and an E/Z mixture in 13% yield (221 mg, 0.780 mmol). The total combined yield of **1f** is 50% (856 mg, 2.99 mmol).

E isomer: **^{1}H NMR (400 MHz, THF-*d8*)** δ 8.02 (d, J = 1.5 Hz, 1H), 7.92 (s, 1H), 7.89 (d, J = 8.4 Hz, 1H), 7.85 – 7.79 (m, 4H), 7.66 (dd, J = 8.5, 1.6 Hz, 1H), 7.57 (d, J = 5.4 Hz, 1H), 7.46 – 7.40 (m, 4H), 7.38 (dd, J = 5.5, 0.6 Hz, 1H). Carbon NMR unable to be obtained due to the insolubility of the sample in all common NMR solvents.

Z isomer: **^{1}H NMR (400 MHz, $CDCl_3$)** δ 7.82 – 7.77 (m, 3H), 7.76 – 7.69 (m, 2H), 7.65 (d, J = 8.5 Hz, 1H), 7.46 (dd, J = 6.2, 3.3 Hz, 2H), 7.43 – 7.38 (m, 2H), 7.30 (dd, J = 8.4, 1.3 Hz, 1H), 7.23 (d, J = 5.4 Hz, 1H), 6.83 (s, 2H). **^{13}C NMR (101 MHz, $CDCl_3$)** δ 139.9, 138.7, 135.1, 133.6, 133.6, 132.7, 130.7, 130.1, 128.2, 128.1, 127.8, 127.6, 127.1, 126.7, 126.2, 126.0, 125.5, 124.1, 122.2.

HRMS (ASAP, QTOF) m/z: $[M]^{+\cdot}$ calcd. for $C_{20}H_{14}S$ 286.0816; Found 286.0808.

Melting point: 238-242 °C (*E*-isomer), 79-84 °C (*Z*-isomer).

2,5-bis(2-(naphthalen-2-yl)vinyl)thiophene (1g)[7]

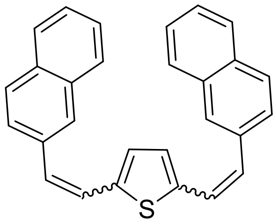

Thiophene-2,5-dicarboxaldehyde was subjected to general procedure A on a scale of 3.00 mmol (Wittig salt **S5a** in excess by 2.50 equiv.). Due to the low solubility of the product, after the quench with distilled water, the crude was washed with a small quantity of cold methanol and dried under vacuum filtration to give product **1g** as a bright yellow solid in 46% yield (538 mg, 1.38 mmol).

EZ:EE Mixture (2:18): **^{1}H NMR (400 MHz, THF-*d8*)** δ 7.92 (s, 1.1H), 7.89 (s, 9.4H), 7.87 – 7.72 (m, 45.4H), 7.70 – 7.64 (m, 1.5H), 7.53 (dd, J = 8.5, 1.7 Hz, 1.2H), 7.49 (s, 4.3H), 7.48 – 7.36 (m, 28.9H), 7.32 (d, J = 16.1 Hz, 1H), 7.13 (d, J = 16.1 Hz, 9.5H), 7.08 (s, 9.2H), 6.97 (s, 2H), 6.90 (d, J = 16.1 Hz, 1H), 6.76 (d, J = 12.0 Hz, 1H), 6.70 (d, J = 11.9 Hz, 1H). Due to the low solubility of **1g**, no suitable ^{13}C-NMR data was obtained.

HRMS (ASAP, QTOF) m/z: $[M+H]^+$ calcd. for $C_{28}H_{21}S$ 389.1364; Found 389.1357.

The ^{1}H NMR peaks of **1g** in THF-*d8* did not match those reported in the literature in $CDCl_3$.

3-(2-(naphthalen-2-yl)vinyl)quinoline (1h)[8]

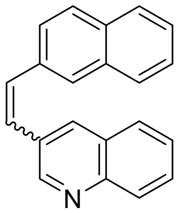

3-Quinoline carboxaldehyde was subjected to general procedure A on a scale of 1.50 mmol and purified by column chromatography (1:9 EtOAc:pentane) to give the *E*-isomer as a pale yellow solid in 29% yield (124 mg, 0.425 mmol), the *Z*-isomer as a pale yellow solid in 43% yield (183 mg, 0.645 mmol) and an E/Z mixture in 7% yield (27.9 mg, 0.105 mmol). The total combined yield of **1h** is 79% (335 mg, 1.19 mmol).

E isomer: **^{1}H NMR (400 MHz, $CDCl_3$)** δ 9.18 (d, *J* = 1.9 Hz, 1H), 8.21 (s, 1H), 8.11 (d, *J* = 8.4 Hz, 1H), 7.93 (s, 1H), 7.90 – 7.76 (m, 5H), 7.69 (t, *J* = 7.6 Hz, 1H), 7.59 – 7.45 (m, 4H), 7.36 (d, *J* = 16.4 Hz, 1H). **^{13}C NMR (101 MHz, $CDCl_3$)** δ 150.4, 148.3, 134.7, 134.1, 133.7, 133.6, 133.5, 132.4, 128.7, 128.3, 127.9, 127.8, 126.7, 126.6, 123.6, 123.6, 122.9.

Z isomer: ^{1}**H NMR (400 MHz, CDCl$_3$)** δ 8.79 (d, *J* = 1.8 Hz, 1H), 8.07 – 8.01 (m, 2H), 7.79 – 7.75 (m, 2H), 7.73 – 7.69 (m, 1H), 7.69 – 7.62 (m, 3H), 7.51 – 7.41 (m, 3H), 7.34 (d, *J* = 8.5 Hz, 1H), 6.99 (d, *J* = 12.2 Hz, 1H), 6.79 (d, *J* = 12.1 Hz, 1H).). ^{13}**C NMR (101 MHz, CDCl$_3$)** δ 151.0, 148.5, 139.5, 133.5, 133.5, 133.5, 132.9, 132.6, 128.6, 128.2, 128.1, 127.8, 126.6, 126.5, 126.5, 125.7, 122.2.

HRMS (ASAP, QTOF) m/z: [M+H]$^+$ calcd. for $C_{21}H_{16}N$ 282.1283; Found 282.1276.

Melting point: 167-169 °C (*E*-isomer), 88-92 °C (*Z*-isomer).

NMR spectra of **(*E*)-1h** matched those reported in literature.

4-(2-(naphthalen-2-yl)vinyl)thiazole (**1i**)

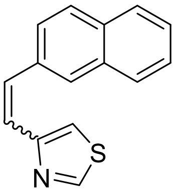

Thiazole-4-carbaldehyde was subjected to general procedure A on a scale of 4.00 mmol and purified by column chromatography (1:9 EtOAc:hexane) to give product **1i** as a yellow waxy solid (*E*/*Z* mixture) in 84% yield (796 mg, 3.36 mmol).

E:Z mixture (1:1.7): ^{1}**H NMR (400 MHz, CDCl$_3$)** δ 8.84 (dd, J = 2.0, 0.5 Hz, 1.0H), 8.73 (d, J = 2.0 Hz, 1.7H), 7.91 (s, 1.0H), 7.87 – 7.69 (m, 11.6H), 7.52 – 7.44 (m, 6.8H), 7.30 (d, J = 16.0 Hz, 1.0H), 7.22 (d, J = 2.0 Hz, 1.0H), 7.02 (dd, J = 2.0, 0.4 Hz, 1.7H), 6.92 (d, J = 12.4 Hz, 1.7H), 6.85 (d, J = 12.4 Hz, 1.7H). ^{13}**C NMR (101 MHz, CDCl$_3$)** δ 155.1, 153.2, 153.1, 151.6, 134.8, 134.5, 133.7, 133.5, 133.3, 132.8, 132.0, 131.8, 128.4, 128.2, 128.1, 127.9, 127.9, 127.8, 127.7, 127.2, 126.8, 126.4, 126.2, 126.2, 126.1, 123.9, 123.6, 121.4, 116.6, 114.9.

HRMS (ASAP, QTOF) m/z: [M+H]$^+$ calcd. for $C_{15}H_{11}NS$ 238.0690; Found 238.0693.

5-(2-(naphthalen-2-yl)vinyl)benzo[*d*]oxazole (**1k**)

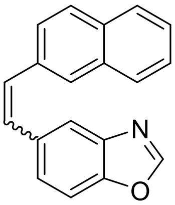

Benzo[*d*]oxazole-5-carbaldehyde was subjected to general procedure A on a scale of 6.80 mmol and purified by column chromatography (2:8 EtOAc:hexane) to give the *E*-isomer as a pink solid in 33% yield (608 mg, 2.24 mmol), the *Z*-isomer as a pink oily solid in 16% yield (295 mg, 1.09 mmol) and an E/Z mixture in 6% yield (120 mg, 0.408 mmol). The total combined yield of **1k** is 55% (1.02 g, 3.77 mmol).

E isomer: ^{1}**H NMR (400 MHz, CDCl$_3$)** δ 8.11 (s, 1H), 7.97 (d, J = 1.5 Hz, 1H), 7.89 (s, 1H), 7.83 (dd, J = 12.2, 4.8 Hz, 3H), 7.77 (dd, J = 8.6, 1.7 Hz, 1H), 7.63 (dd, J = 8.5, 1.7 Hz, 1H), 7.58 (dd, J = 8.5, 0.5 Hz, 1H), 7.52 – 7.43 (m, 2H), 7.35 (d, J = 16.3 Hz, 1H), 7.29 (d, J = 16.3 Hz, 1H). ^{13}**C NMR (101 MHz, CDCl$_3$)** δ 153.2, 149.8, 140.9, 134.8, 134.8, 133.9, 133.2, 129.2, 128.6, 128.5, 128.2, 127.9, 126.8, 126.5, 126.1, 124.7, 123.6, 118.4, 111.2.

Z isomer: ^{1}**H NMR (400 MHz, CDCl$_3$)** δ 8.05 (s, 1H), 7.79 – 7.68 (m, 4H), 7.64 (d, J = 8.5 Hz, 1H), 7.46 – 7.41 (m, 2H), 7.40 (d, J = 8.6 Hz, 1H), 7.31 (ddd, J = 8.5, 2.9, 1.7 Hz, 2H), 6.81 (dd, 2H). ^{13}**C NMR (101 MHz, CDCl$_3$)** δ 153.0, 149.3, 140.4, 134.6, 134.3, 133.6, 132.7, 130.6, 130.1, 128.2, 128.0, 127.8, 127.8, 127.1, 126.9, 126.2, 126.1, 121.0, 110.7.

HRMS (ASAP, QTOF) m/z: [M+H]$^+$ calcd. for $C_{19}H_{14}NO$ 272.1075; Found 272.1067.

Melting point: 183-185 °C (*E*-isomer), 61-65 °C (*Z*-isomer).

3-chloro-5-(2-(naphthalen-2-yl)vinyl)pyridine (**1m**)

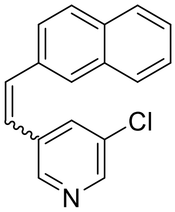

5-Chloronicotinaldehyde was subjected to general procedure A on a scale of 2.00 mmol and purified by column chromatography (1:9 EtOAc:pentane) to give the *E*-isomer as a white solid in 8% yield (66.6 mg, 0.160 mmol), the *Z*-isomer as a beige solid in 41% yield (330 mg, 0.820 mmol) and an E/Z mixture in 42% yield (337 mg, 0.840 mmol). The total combined yield of **1m** is 88% (467 mg, 1.76 mmol).

E isomer: **^{1}H NMR (400 MHz, $CDCl_3$)** δ 8.63 (d, J = 1.9 Hz, 1H), 8.46 (d, J = 2.3 Hz, 1H), 7.90 – 7.81 (m, 5H), 7.73 (dd, J = 8.6, 1.7 Hz, 1H), 7.54 – 7.46 (m, 2H), 7.34 (d, J = 16.4 Hz, 1H), 7.14 (d, J = 16.4 Hz, 1H). **^{13}C NMR (101 MHz, $CDCl_3$)** δ 147.4, 146.4, 134.5, 133.8, 133.7, 133.6, 132.6, 132.4, 132.3, 128.8, 128.3, 127.9, 127.7, 126.8, 126.7, 123.7, 123.4.

Z isomer: **^{1}H NMR (400 MHz, $CDCl_3$)** δ 8.39 (dd, J = 10.5, 2.0 Hz, 2H), 7.82 – 7.77 (m, 1H), 7.76 – 7.69 (m, 3H), 7.57 – 7.54 (m, 1H), 7.50 – 7.44 (m, 2H), 7.28 (dd, J = 8.6, 1.6 Hz, 1H), 6.95 (d, J = 12.2 Hz, 1H), 6.56 (d, J = 12.2 Hz, 1H). **^{13}C NMR (101 MHz, $CDCl_3$)** δ 148.1, 147.1, 135.6, 134.4, 134.1, 133.6, 133.6, 133.0, 131.7, 128.4, 128.3, 128.2, 127.9, 126.6, 126.5, 126.3, 125.2.

HRMS (ES, TOF) m/z: $[M+H]^+$ calcd. for $C_{17}H_{13}ClN$ 266.0736; Found 266.0749.

Melting point: 139-141 °C (E-isomer), 42-43 °C (Z-isomer).

5-(2-(naphthalen-2-yl)vinyl)benzo[*c*][1,2,5]thiadiazole (**1n**)[9]

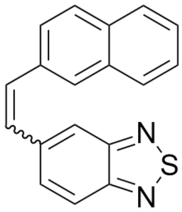

The procedure to prepare **1n** was borrowed from the literature. To a solution of **S5a** (2.94 g, 6.08 mmol, 1.00 equiv.) in dry THF (100 mL), was added *n*-BuLi (1.6 M in hexanes, 4.0 mL, 6.40 mmol, 1.05 equiv.) at -78 °C under nitrogen. The reaction mixture was stirred for 10 min at -78 °C, warmed to room temperature over 30 min, and cooled again to -78 °C. To the reaction mixture was added 2,1,3-benzothiadiazole-5-carboxaldehyde (1.00 g, 6.09 mmol, 1.00 equiv.) in THF (10 ml) and stirred at -78 °C for 10 min, followed by stirring at room temperature overnight. The reaction mixture was quenched with distilled water and the crude product was extracted with EtOAc. The combined organic layers were washed with brine, dried under Na_2SO_4 and concentrated *in vacuo*. The crude product was purified by column chromatography *via* 3:17 to 100:0 DCM:pentane elution gradient to afford the *E* isomer as a yellow solid in 52% yield (913 mg, 3.17 mmol) and a *E/Z*-isomeric mixture (1:7) as a yellow solid in 29% yield (504 mg, 1.75 mmol).

E isomer: **^{1}H NMR (400 MHz, $CDCl_3$)** δ 8.03 – 7.92 (m, 4H), 7.90 – 7.77 (m, 4H), 7.54 – 7.43 (m, 3H), 7.38 (d, *J* = 16.3 Hz, 1H). **^{13}C NMR (101 MHz, $CDCl_3$)** δ 155.8, 154.8, 138.9, 134.2, 133.8, 133.5, 132.0, 128.7, 128.3, 128.1, 127.9, 127.8, 127.6, 126.7, 126.5, 123.5, 121.6, 119.1.

HRMS (ESI^+) calculated for $C_{18}H_{13}N_2S$ $[M+H]^+$ 289.0799, found 289.0794.

Melting point: 169-171 °C

Mixture (1:7 *E:Z*)**: ^{1}H NMR (400 MHz, $CDCl_3$)** δ 8.03 – 7.93 (m, 0.58H), 7.92 (s, 1H), 7.89 – 7.83 (m, 0.42H), 7.82 – 7.65 (m, 5.07H), 7.52 – 7.40 (m, 3.42H), 7.33 (dd, *J* = 8.5, 1.4 Hz, 1H), 6.98 (d, *J* = 12.2 Hz, 1H), 6.83 (d, *J* = 12.2 Hz, 1H). **^{13}C NMR (101 MHz, $CDCl_3$)** δ 155.4, 154.3, 139.2, 134.2, 133.5, 133.0, 133.0, 131.5, 129.2, 128.5, 128.1, 128.1, 127.8, 126.9, 126.5, 126.4, 121.1, 120.7.

HRMS (ESI^+) calculated for $C_{18}H_{13}N_2S$ $[M+H]^+$ 289.0799, found 289.0780.

The procedure was adapted from a procedure by Hauser, Caricato, Avarvari, and co-workers. The NMR spectra of **1n** matched those reported in the same article.

2-chloro-3-(2-(naphthalen-2-yl)vinyl)pyridine (1o)

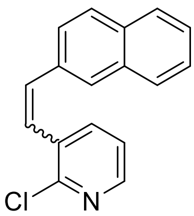

2-Chloronicotinaldehyde was subjected to general procedure A on a scale of 2.00 mmol and purified by column chromatography (1:9 EtOAc:pentane) to give the *E*-isomer as a yellow-green solid in 24% yield (129 mg, 0.480 mmol) and the *Z*-isomer as a pale, yellow solid in 61% yield (323 mg, 1.22 mmol). The total combined yield of **1o** is 85% (452 mg, 1.70 mmol).

E isomer: **^{1}H NMR (400 MHz, $CDCl_3$)** δ 8.31 (dd, *J* = 4.6, 1.5 Hz, 1H), 8.03 (dd, *J* = 7.7, 1.3 Hz, 1H), 7.91 – 7.80 (m, 4H), 7.78 (d, *J* = 8.6 Hz, 1H), 7.55 (s, 1H), 7.53 – 7.45 (m, 3H), 7.27 (m, 1H). **^{13}C NMR (101 MHz, $CDCl_3$)** δ 150.4, 148.3, 134.7, 134.1, 133.7, 133.6, 133.5, 132.4, 128.7, 128.3, 127.9, 127.8, 126.7, 126.6, 123.6, 123.6, 122.9.

Z isomer: **^{1}H NMR (400 MHz, $CDCl_3$)** δ 8.27 (dd, *J* = 4.6, 1.6 Hz, 1H), 7.79 – 7.74 (m, 1H), 7.73 – 7.68 (m, 1H), 7.65 (d, *J* = 8.3 Hz, 2H), 7.51 – 7.41 (m, 3H), 7.21 (d, *J* = 9.6 Hz, 1H), 6.98 (dd, *J* = 12.1, 6.8 Hz, 2H), 6.71 (d, *J* = 12.1 Hz, 1H). **^{13}C NMR (101 MHz, $CDCl_3$)** δ 151.0, 148.5, 139.5, 133.5, 133.5, 133.5, 132.9, 132.6, 128.6, 128.2, 128.1, 127.8, 126.6, 126.5, 126.5, 125.7, 122.2.

HRMS (ES, TOF) m/z: $[M+H]^+$ calcd. for $C_{17}H_{13}ClN$ 266.0736; Found 266.0749.

Melting Point: 139-141 °C (*E*-isomer), 80-84 °C (*Z*-isomer).

5-(2-(benzo[*c*]phenanthren-2-yl)vinyl)benzo[*b*]thiophene (1p)

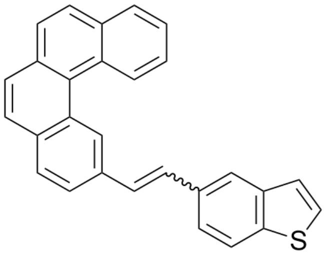

Benzo[*b*]thiophene-5-carbaldehyde was subjected to general procedure A (Wittig salt **S5b** was used instead of **S5a**) on a scale of 0.20 mmol and purified by column chromatography *via* 0:100 to 1:19 EtOAc:hexane elution gradient, followed by purification by recycling GPC in chloroform to afford **1p** as a pale yellow solid in 73% yield (56.5 mg, 0.146 mmol).

^{1}H NMR (400 MHz, $CDCl_3$) δ 9.23 – 9.17 (m, 1H), 8.96 (s, 1H), 8.21 (d, *J* = 8.6 Hz, 1H), 8.07 – 8.00 (m, 4.35H), 7.96 – 7.87 (m, 8.95H), 7.87 – 7.73 (m, 9.65H), 7.70 – 7.64 (m, 2.91H), 7.57 (dd, *J* = 8.3, 1.6 Hz, 1H), 7.51 – 7.37 (m, 9.03H), 7.29 (dd, *J* = 5.4, 0.6 Hz, 1H), 6.91 (s, 2H), 6.73 (ddd, *J* = 8.5, 6.9, 1.4 Hz, 1H). **^{13}C NMR (101 MHz, $CDCl_3$)** δ 192.2, 140.4, 140.2, 139.2, 138.8, 135.5, 135.4, 134.0, 133.9, 133.7, 133.5, 133.2, 132.6, 131.5, 131.3, 131.0, 130.8, 130.5, 130.4, 130.2, 129.5, 129.2, 129.1, 129.1, 128.8, 128.7, 128.5, 128.4, 128.0, 127.7, 127.7, 127.6, 127.5, 127.4, 127.3, 127.3, 127.2, 127.0, 127.0, 127.0, 127.0, 126.8, 126.4, 126.0, 126.0, 125.9, 125.9, 124.2, 124.2, 124.2, 123.4, 122.8, 122.8, 122.6, 122.2.

HRMS (APCI$^+$, Orbitrap) calculated for $C_{28}H_{19}S$ $[M+H]^+$ 387.1207, found 387.1192.

3,6-dibromo-9-((2-(trimethylsilyl)ethoxy)methyl)-9*H*-carbazole (**1qa**)[10]

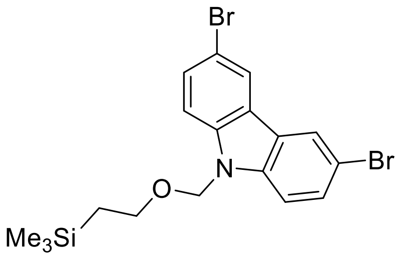

To a solution of 3,6-dibromocarbazole (3.20 g, 9.85 mmol, 1.00 equiv.) in dry DMF (10 mL), was added sodium hydride (60 wt.% in mineral oil) (1.20 g, 30.0 mmol, 3.00 equiv.) under inert, anhydrous conditions and stirred at room temperature for 20 min. SEM-Cl (3.50 mL, 19.8 mmol, 2.00 equiv.) was added dropwise and stirred overnight at room temperature. The reaction mixture was quenched with distilled water and the crude product was extracted with DCM. The combined organic layers were washed with brine, dried under Na_2SO_4 and concentrated *in vacuo*. The crude product was purified by column chromatography *via* 0:100 to 1:20 EtOAc:hexane elution gradient to afford **1qa** as a white solid (2.75 g) containing 30 mol% SEM-Cl. The corrected quantity of product is 2.48 g (5.45 mmol, 55%).

^{1}H NMR (400 MHz, $CDCl_3$) δ 8.11 (dd, *J* = 1.9, 0.4 Hz, 2H), 7.56 (dd, *J* = 8.7, 1.9 Hz, 2H), 7.39 (d, *J* = 8.7 Hz, 2H), 5.62 (s, 2H), 3.52 – 3.46 (m, 2H), 0.91 – 0.84 (m, 2H), -0.10 (s, 9H). **^{13}C NMR (101 MHz, $CDCl_3$)** δ 139.7, 129.5, 124.1, 123.4, 113.2, 111.1, 72.7, 66.4, 17.9, -1.3.

HRMS (APCI$^+$, Orbitrap) calculated for C18H21Br2NOSi $[M]^+$ 452.9759, found 452.9740.

The procedure was adapted from a previous report.

9-((2-(trimethylsilyl)ethoxy)methyl)-9*H*-carbazole-3,6-dicarbaldehyde (**1qb**)[11]

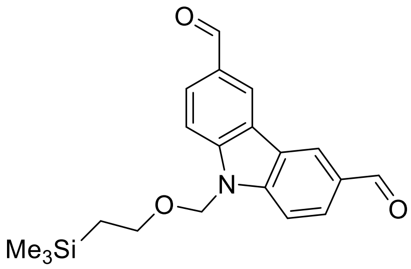

To a solution of **1qa** (4.00 g, 8.79 mmol, 1.00 equiv.) in dry THF (50 mL), was added *n*-BuLi (1.6 M in hexanes, 13.8 mL, 22.1 mmol, 2.50 equiv.) dropwise at -78 °C under inert, anhydrous conditions and stirred for 1 h. Dry DMF (4.0 mL) was added dropwise to the reaction mixture at -78 °C and stirred for 1 h at -78 °C, followed by 2 h at room temperature. The reaction mixture was quenched with distilled water and the crude product was extracted with DCM. The combined organic layers were washed with brine and dried under Na_2SO_4 and concentrated *in vacuo*. The crude product was purified by column chromatography *via* 2:8 to 3:7 EtOAc:hexane elution gradient to afford **1qb** as a white solid in 84% yield (2.61 g, 7.38 mmol).

^{1}H NMR (400 MHz, $CDCl_3$) δ 10.14 (s, 2H), 8.64 (d, *J* = 1.2 Hz, 2H), 8.08 (dd, *J* = 8.5, 1.6 Hz, 2H), 7.69 (d, *J* = 8.5 Hz, 2H), 5.77 (s, 2H), 3.61 – 3.55 (m, 2H), 0.94 – 0.88 (m, 2H), -0.10 (s, 9H). **^{13}C NMR (101 MHz, $CDCl_3$)** δ 191.6, 145.0, 130.5, 128.3, 124.1, 123.7, 110.5, 72.9, 66.9, 17.9, -1.4.

HRMS (ES, TOF) calculated for $C_{20}H_{24}NO_3Si$ $[M+H]^+$ 354.1525, found 354.1522.

The procedure was adapted from a previous report.

3,6-bis((*E*)-4-bromostyryl)-9-((2-(trimethylsilyl)ethoxy)methyl)-9*H*-carbazole (**1q**)[12]

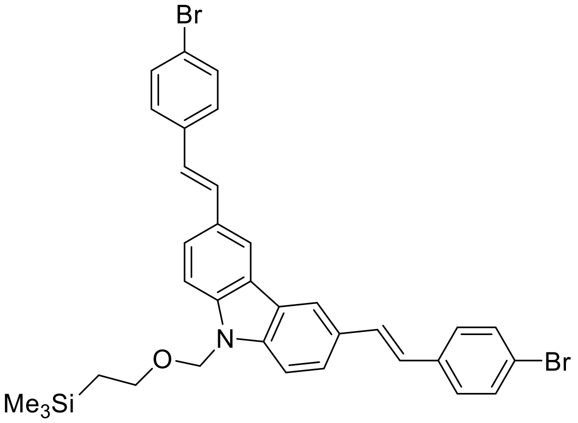

To a solution of diethyl (4-bromobenzyl)phosphonate (2.5 mL, 11.0 mmol, 3.00 equiv.) in THF (30 mL), was added sodium hydride (60 wt.% in mineral oil) (500 mg, 12.5 mmol, 3.30 equiv.) under inert, anhydrous conditions and stirred for 40 min at 45 °C. To the reaction mixture was added **1qb** (1.33 g, 3.76 mmol, 1.00 equiv.) in THF (20 mL) at room temperature. The reaction mixture was then refluxed overnight. The reaction mixture was quenched with distilled water and the crude product was extracted with DCM. The combined organic layers were washed with brine, dried under Na_2SO_4 and concentrated *in vacuo*. The crude product was purified by column chromatography *via* 2:23 to 7:43 EtOAc:hexane elution gradient to afford **1q** as a white solid in 83% yield (2.05 g, 3.12 mmol).

^{1}H NMR (400 MHz, CDCl$_3$) δ 8.20 (d, *J* = 1.5 Hz, 2H), 7.65 (dd, *J* = 8.6, 1.6 Hz, 2H), 7.53 – 7.46 (m, 6H), 7.45 – 7.38 (m, 4H), 7.28 (d, *J* = 15.7 Hz, 2H), 7.09 (d, *J* = 16.3 Hz, 2H), 5.67 (s, 2H), 3.59 – 3.53 (m, 2H), 0.94 – 0.88 (m, 2H), -0.08 (s, 9H). **^{13}C NMR (101 MHz, CDCl$_3$)** δ 141.1, 136.9, 131.9, 130.2, 129.6, 127.9, 125.5, 125.2, 123.9, 120.9, 118.8, 109.9, 72.7, 66.4, 18.0, -1.3.

HRMS (ES, TOF) calculated for $C_{34}H_{33}Br_2NOSi$ $[M]^+$ 657.0698, found 657.0698.

The procedure was adapted from a previous report.

[4]Thienohelicene (**2a**)[13]

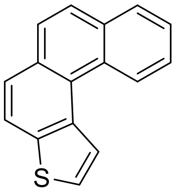

1a was subjected to general procedure B on a scale of 0.200 mmol and purified by column chromatography (pentane) to give product **2a** as a white solid in 94% yield (44.1 mg, 0.188 mmol).

^{1}H NMR (400 MHz, CDCl$_3$) δ 9.16 (d, *J* = 8.5 Hz, 1H), 8.65 (d, *J* = 5.6 Hz, 1H), 8.09 (d, *J* = 8.4 Hz, 1H), 8.00 (d, *J* = 7.9 Hz, 1H), 7.91 – 7.80 (m, 3H), 7.77 – 7.70 (m, 2H), 7.65 (t, *J* = 7.2 Hz, 1H). **^{13}C NMR (101 MHz, CDCl$_3$)** δ 140.3, 135.1, 133.2, 130.6, 130.6, 128.9, 127.6, 126.8, 126.6, 126.5, 126.5, 126.5, 126.3, 125.8, 1\ 25.7, 121.6.

HRMS (ASAP, QTOF) m/z: $[M]^{+\cdot}$ calcd. for $C_{16}H_{10}S$ 234.0503; Found 234.0497.

NMR spectra of **2a** matched those reported in literature.

2-Chloro[4]thienohelicene (**2b**)

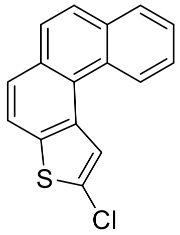

1b was subjected to general procedure B on a scale of 0.200 mmol and purified by column chromatography (hexane) to give product **2b** as a white solid in 77% yield (41.3 mg, 0.154 mmol). When scaled up to 9.80 mmol, product **2b** was achieved in 79% yield (2.06 g, 7.66 mmol).

^{1}H NMR (400 MHz, $CDCl_3$) δ 8.95 (d, *J* = 8.4 Hz, 1H), 8.44 (s, 1H), 7.97 (d, *J* = 7.8 Hz, 1H), 7.86 (d, *J* = 8.5 Hz, 1H), 7.82 – 7.76 (m, 3H), 7.72 (t, *J* = 7.5 Hz, 1H), 7.65 (t, *J* = 7.3 Hz, 1H). **^{13}C NMR (101 MHz, $CDCl_3$)** δ 139.0, 133.9, 133.0, 131.9, 130.6, 130.2, 128.8, 127.4, 126.8, 126.6, 126.4, 126.0, 125.9, 125.8, 124.9, 120.5.

HRMS (ASAP, QTOF) m/z: $[M]^{+\cdot}$ calcd. for $C_{16}H_9ClS$ 268.0113; Found 268.0105.

[4]Furohelicene (**2c**)[14]

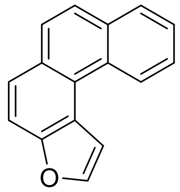

1c was subjected to general procedure B on a scale of 0.200 mmol and purified by column chromatography (2.5:97.5 EtOAc:hexane) to give product **2c** as a white solid in 74% yield (32.2 mg, 0.147 mmol).

^{1}H NMR (400 MHz, $CDCl_3$) δ 9.03 (d, J = 8.4 Hz, 1H), 8.00 (dd, J = 7.9, 1.4 Hz, 1H), 7.91 (d, J = 2.3 Hz, 1H), 7.89 (d, J = 8.7 Hz, 1H), 7.87 – 7.82 (m, 3H), 7.80 (d, J = 8.7 Hz, 1H), 7.75 (ddd, J = 8.4, 7.0, 1.5 Hz, 1H), 7.66 (ddd, J = 8.0, 7.0, 1.2 Hz, 1H). **^{13}C NMR (101 MHz, $CDCl_3$)** δ 154.4, 144.9, 132.9, 130.9, 129.4, 128.9, 127.9, 126.5, 126.3, 126.2, 125.7, 125.5, 125.2, 122.2, 112.4, 108.6.

HRMS (ASAP, QTOF) m/z: $[M+H]^+$ calcd. for $C_{16}H_{11}O$ 219.0810; Found 219.0814.

Previous literature reports did not provide NMR spectra.

2-chloro[4]furohelicene (**2d**)

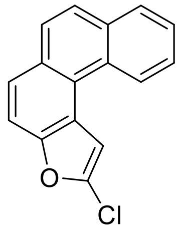

1d was subjected to general procedure B on a scale of 0.200 mmol and purified by column chromatography (hexane) to give product **2d** as a white solid in 48% yield (24.2 mg, 0.096 mmol). When using a 60 W 365 nm lamp at 40°C, the yield of **2d** was increased to 57% yield (28.8 mg, 0.114 mmol).

^{1}H NMR (400 MHz, $CDCl_3$) δ 8.83 (d, J = 8.3 Hz, 1H), 8.00 – 7.94 (m, 1H), 7.83 (d, J = 8.8 Hz, 1H), 7.79 (t, J = 9.2 Hz, 2H), 7.71 (ddd, J = 8.4, 6.0, 2.1 Hz, 2H), 7.68 – 7.61 (m, 2H). **^{13}C NMR (101 MHz, $CDCl_3$)** δ 153.4, 141.2, 132.9, 130.6, 129.6, 128.9, 127.8, 126.7, 126.5, 126.2, 126.0, 125.2, 124.3, 123.2, 111.6, 105.4.

HRMS (ASAP, QTOF) m/z: $[M]^{+\cdot}$ calcd. for $C_{16}H_9ClO$ 252.0342; Found 252.0337.

[6]Thienohelicene (**2e**)

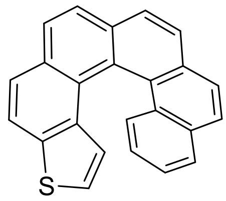

1e was subjected to general procedure B on a scale of 0.200 mmol and purified by column chromatography (1:99 EtOAc:hexane) to give product **2e** as a pale yellow solid in 71% yield (47.2 mg, 0.141 mmol).

^{1}H NMR (400 MHz, $CDCl_3$) δ 8.05 (t, J = 8.7 Hz, 2H), 7.98 – 7.90 (m, 7H), 7.87 (d, J = 8.6 Hz, 1H), 7.38 – 7.32 (m, 1H), 6.90 (ddd, J = 8.4, 6.9, 1.3 Hz, 1H), 6.86 (d, J = 5.6 Hz, 1H), 6.63 (dd, J = 5.6, 0.6 Hz, 1H). **^{13}C NMR (101 MHz, $CDCl_3$)** δ 138.1, 135.8, 132.8, 131.8, 131.8, 130.8, 130.6, 127.8, 127.8, 127.8, 127.6, 127.4, 127.4, 127.4, 126.9, 126.7, 126.3, 125.9, 125.6, 125.2, 124.7, 124.7, 123.6, 121.8.

HRMS (ES, TOF) m/z: $[M+H]^+$ calcd. for $C_{24}H_{15}S$ 335.0894; Found 335.0893.

Melting point: 186-189 °C.

[5]Thienohelicene (**2f**)[15]

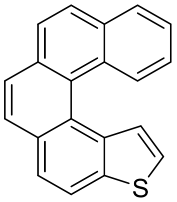

1f was subjected to general procedure B on a scale of 0.200 mmol and purified by column chromatography (2.5:97.5 EtOAc:hexane) to give product **2f** as a white solid in 56% yield (31.7 mg, 0.111 mmol).

^{1}H NMR (400 MHz, CDCl$_3$) δ 8.64 (d, J = 8.2 Hz, 1H), 8.09 (dd, J = 8.5, 0.7 Hz, 1H), 8.00 (dd, J = 8.0, 1.2 Hz, 1H), 7.96 (dd, J = 8.4, 3.1 Hz, 2H), 7.91 (d, J = 8.5 Hz, 1H), 7.87 (d, J = 8.5 Hz, 1H), 7.82 (d, J = 8.3 Hz, 1H), 7.79 (d, J = 5.6 Hz, 1H), 7.58 (ddd, J = 8.0, 6.9, 1.2 Hz, 1H), 7.44 (ddd, J = 8.4, 6.9, 1.4 Hz, 1H), 7.39 (d, J = 5.5 Hz, 1H). **^{13}C NMR (101 MHz, CDCl$_3$)** δ 139.3, 136.3, 133.0, 131.9, 131.6, 130.3, 129.1, 127.9, 127.8, 127.7, 127.4, 127.0, 126.5, 126.4, 126.0, 126.0, 125.1, 124.6, 123.6, 121.6.

HRMS (ASAP, QTOF) m/z: $[M+H]^+$ calcd. for $C_{20}H_{12}S$ 285.0738; Found 285.0737.

Melting point: 108-113 °C.

This compound was previously reported in the literature, but no synthetic or characterisation data was provided.

[7]Thienohelicene (**2g**)[16]

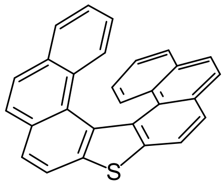

1g was subjected to general procedure B (at a concentration of 0.50 mM) on a scale of 0.200 mmol and purified by column chromatography (1:19 EtOAc:hexane) to give product **2g** as a yellow solid in 73% yield (55.9 mg, 0.145 mmol).

^{1}H NMR (400 MHz, CDCl$_3$) δ 8.15 (d, *J* = 8.2 Hz, 2H), 8.03 (d, *J* = 8.2 Hz, 2H), 7.93 (d, *J* = 8.6 Hz, 2H), 7.80 (d, *J* = 8.6 Hz, 2H), 7.63 (d, *J* = 7.9 Hz, 2H), 7.50 (d, *J* = 8.4 Hz, 2H), 7.07 (t, *J* = 7.4 Hz, 2H), 6.32 (t, *J* = 7.7 Hz, 2H). **^{13}C NMR (101 MHz, CDCl$_3$)** δ 139.6, 131.4, 130.9, 130.2, 129.9, 128.7, 127.6, 126.8, 126.5, 126.5, 126.4, 126.3, 123.7, 121.2.

HRMS (ASAP, QTOF) m/z: $[M+H]^+$ calcd. for $C_{28}H_{17}S$ 385.1051; Found 385.1046.

Melting point: 241-243 °C.

NMR spectra of **2g** matched those reported in literature.

[5]Pyridohelicene (**2h**)[8]

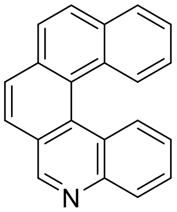

1h subjected to general procedure B (using 60 W 365 nm LED lamp at 40 °C) on a scale of 0.200 mmol and purified by column chromatography (1:29:70 Et_3N:EtOAc:hexane) to give product **2h** as a white solid in 44% yield (24.5 mg, 0.088 mmol).

^{1}H NMR (400 MHz, CDCl$_3$) δ 9.40 (s, 1H), 8.61 (d, J = 8.5 Hz, 1H), 8.57 – 8.49 (m, 1H), 8.26 (dd, J = 8.3, 1.1 Hz, 1H), 8.01 (d, J = 8.6 Hz, 1H), 7.99 – 7.94 (m, 3H), 7.89 – 7.85 (m, 1H), 7.69 (ddd, J = 8.3, 6.9, 1.4 Hz, 1H), 7.58 (ddd, J = 8.0, 6.9, 1.1 Hz, 1H), 7.34 (dddd, J = 8.4, 6.9, 4.3, 1.4 Hz, 2H). **^{13}C NMR (101 MHz, CDCl$_3$)** δ 152.7, 145.8, 134.8, 132.9, 130.6, 130.6, 129.6, 129.6, 128.7, 128.7, 128.5, 128.1, 127.8, 127.1, 126.4, 126.3, 126.1, 125.9, 125.0, 125.0, 124.9.

HRMS (ASAP, QTOF) m/z: $[M+H]^+$ calcd. for $C_{21}H_{14}N$ 280.1126; Found 280.1122.

Melting point: 201-203 °C.

NMR spectra of **2h** matched those reported in literature.

[4]Thiazolohelicene (**2i**)[17] and 3-isothiocyanatophenanthrene (**S2i’**)

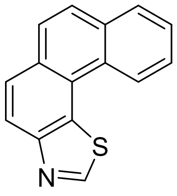

2i

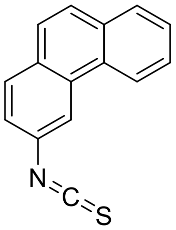

S2i'

1i was subjected to general procedure C on a scale of 0.800 mmol and purified by column chromatography (1:99 EtOAc:hexane) to give the desired product **2i** as a white solid in 35% yield (66.1 mg, 0.281 mmol) and ring-opened side product **S2i’** as a white solid in 26% yield (49.4 mg, 0.210 mmol). **2i** was triturated in Et_2O to give cleaner NMR spectra.

2i: ^{1}H NMR (400 MHz, $CDCl_3$) δ 9.21 (s, 1H), 8.90 (dd, J = 8.4, 0.6 Hz, 1H), 8.37 (d, J = 8.5 Hz, 1H), 8.05 – 8.00 (m, 2H), 7.90 (q, J = 8.8 Hz, 2H), 7.83 (ddd, J = 8.5, 7.0, 1.5 Hz, 1H), 7.70 (ddd, J = 8.0, 7.0, 1.0 Hz, 1H). **^{13}C NMR (101 MHz, $CDCl_3$)** δ 153.5, 153.1, 132.9, 131.2, 129.4, 129.1, 128.5, 128.3, 127.6, 127.3, 126.7, 126.0, 125.3, 122.9.

HRMS (ASAP, QTOF) m/z: $[M]^{+\cdot}$ calcd. for $C_{15}H_9NS$ 235.0456; Found 235.0450.

Melting point: 127-131 °C.

Previous literature reports did not provide NMR spectra of **2i**.

S2i’: **^{1}H NMR (400 MHz, $CDCl_3$)** δ 8.53 (d, J = 8.0 Hz, 1H), 8.44 (d, J = 1.7 Hz, 1H), 7.92 – 7.87 (m, 1H), 7.83 (d, J = 8.5 Hz, 1H), 7.75 (d, J = 8.8 Hz, 1H), 7.71 – 7.61 (m, 3H), 7.41 (dd, J = 8.4, 2.0 Hz, 1H). **^{13}C NMR (101 MHz, $CDCl_3$)** δ 135.6, 132.3, 130.9, 130.7, 130.0, 129.4, 129.3, 128.8, 128.0, 127.5, 127.1, 126.2, 124.1, 122.7, 119.7.

HRMS (ASAP, QTOF) m/z: $[M]^{+\cdot}$ calcd. for $C_{15}H_9NS$ 235.0456; Found 235.0452.

Melting point: 105-106 °C.

IR (ν_{max}/cm^{-1}): 2922, 2855, 2173, 2087.

The structure of **S2i’** was identified by the characteristic strong, broad band at 2087 cm^{-1} and smaller shoulder at 2173 cm^{-1}, which is characteristic of the isothiocyanate group.[18]

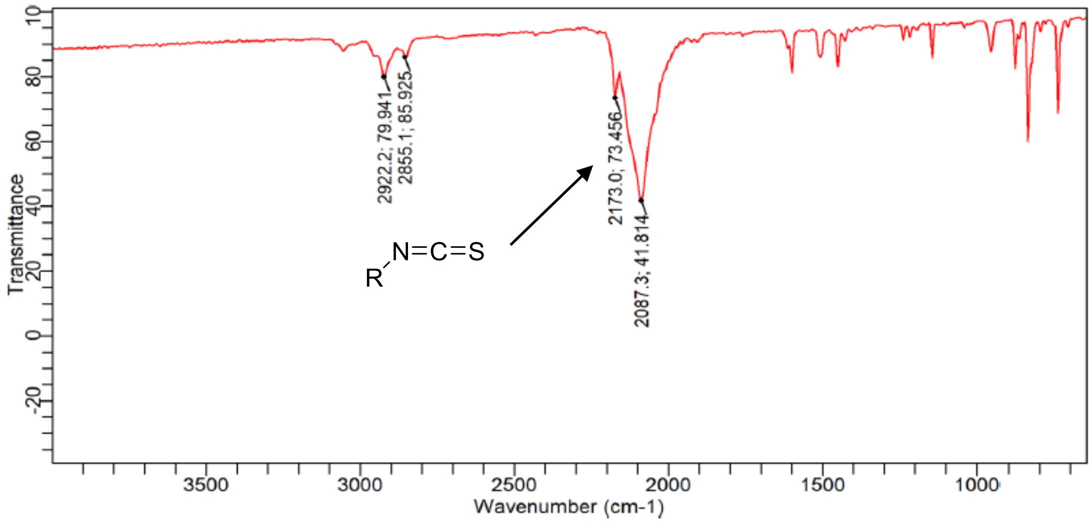

[5]Oxazolohelicene (**2k**)

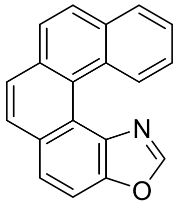

1k was subjected to general procedure C on a scale of 0.200 mmol and purified by column chromatography (1:9 EtOAc:hexane) to give product **2k** as a beige solid in 40% yield (21.3 mg, 0.079 mmol).

^{1}H NMR (400 MHz, $CDCl_3$) δ 8.95 (d, J = 8.4 Hz, 1H), 8.22 (s, 1H), 8.07 – 7.97 (m, 4H), 7.90 (d, J = 8.7 Hz, 1H), 7.86 (d, J = 8.4 Hz, 2H), 7.69 – 7.63 (m, 1H), 7.59 (ddd, J = 8.3, 6.9, 1.5 Hz, 1H). **^{13}C NMR (101 MHz, $CDCl_3$)** δ 149.6, 148.6, 136.8, 133.2, 132.3, 131.8, 130.5, 130.1, 128.9, 127.8, 127.5, 127.4, 126.8, 126.4, 126.3, 126.2, 124.2, 123.7, 111.1.

HRMS (ASAP, QTOF) m/z: $[M+H]^+$ calcd. for $C_{19}H_{12}NO$ 270.0919; Found 270.0922.

Melting point: 147-151 °C

1-chloro[4]pyridohelicene (**2m**) and 3-chloro[4]pyridohelicene (**2m'**)

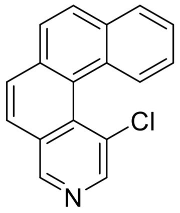

2m

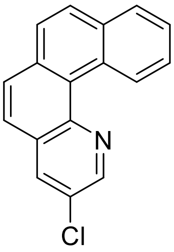

2m'

1m was subjected to general procedure C on a scale of 0.800 mmol and purified by column chromatography *via* 2.5:97.5 to 2:8 EtOAc:hexane elution gradient to give major product **2m** as a pale yellow solid in 51% yield (107.7 mg, 0.408 mmol) and minor product **2m'** as a pale yellow solid in 17% yield (36.8 mg, 0.140 mmol)

2m: **^{1}H NMR (400 MHz, $CDCl_3$)** δ 9.26 (s, 1H), 8.75 (s, 1H), 8.17 – 8.13 (m, 1H), 8.10 (d, J = 8.5 Hz, 1H), 8.05 – 8.00 (m, 1H), 7.95 (s, 2H), 7.89 – 7.84 (m, 1H), 7.69 – 7.63 (m, 2H). **^{13}C NMR (101 MHz, $CDCl_3$)** δ 149.9, 145.6, 134.7, 132.7, 131.4, 131.3, 130.8, 130.0, 128.9, 128.8, 128.7, 127.6, 126.8, 125.6, 125.2, 125.1, 124.4.

HRMS (ASAP, QTOF) m/z: $[M+H]^+$ calcd. for $C_{17}H_{11}ClN$ 264.0580; Found 264.0574.

Melting point: 165-171 °C.

2m': **^{1}H NMR (400 MHz, $CDCl_3$)** δ 11.03 (dd, J = 8.8, 0.5 Hz, 1H), 9.11 (d, J = 2.6 Hz, 1H), 8.26 (d, J = 2.6 Hz, 1H), 8.02 (dd, J = 11.8, 5.1 Hz, 2H), 7.98 (d, J = 8.6 Hz, 1H), 7.88 (d, J = 8.6 Hz, 1H), 7.83 (ddd, J = 8.7, 6.9, 1.6 Hz, 1H), 7.79 (d, J = 8.6 Hz, 1H), 7.69 (ddd, J = 8.0, 6.9, 1.2 Hz, 1H). **^{13}C NMR (101 MHz, $CDCl_3$)** δ 147.3, 146.4, 134.7, 134.1, 133.9, 131.5, 130.6, 130.3, 130.2, 128.7, 128.7, 128.4, 127.6, 126.9, 126.7, 126.5, 125.7.

HRMS (ASAP, QTOF) m/z: $[M+H]^+$ calcd. for $C_{17}H_{11}ClN$ 264.0580; Found 264.0575.

Melting point: 186-189 °C.

[5]Thiadiazolohelicene (**2n**) and tetrapheno[2,3-*c*][1,2,5]thiadiazole (**2n'**)[9]

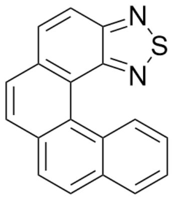

2n

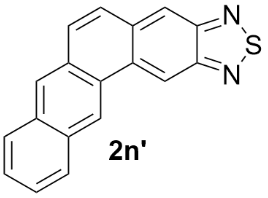

2n'

A solution of **1n** (108 mg, 0.375 mmol, 1.00 equiv.), iodine (18.0 mg, 0.071 mmol, 0.190 equiv.), THF (1.8 mL, 22.0 mmol, 59.0 equiv.) in toluene (360 mL, 1.0 mM) was run through a Vapourtec E-series flow reactor fitted with a 150 W 365 nm LED lamp at 65 °C and 3.3 mL/min with a reactor coil volume of 10 mL. The product mixture was concentrated *in vacuo* and the crude product was purified by column chromatography *via* 1:9 to 1:2 DCM:pentane elution gradient to afford the desired helicene **2n** as a yellow solid in 54% yield (58.0 mg, 0.200 mmol) and linear product **2n'** as a yellow solid in 17% yield (18.0 mg, 0.063 mmol).

2n: ^{1}**H NMR (400 MHz, CDCl$_3$)** δ 8.81 (d, *J* = 8.5 Hz, 1H), 8.09 – 8.01 (m, 3H), 8.00 – 7.93 (m, 3H), 7.83 (d, *J* = 8.6 Hz, 1H), 7.66 (t, *J* = 7.5 Hz, 1H), 7.47 (t, *J* = 7.2 Hz, 1H). ^{13}**C NMR (101 MHz, CDCl$_3$)** δ 155.1, 153.8, 133.9, 133.6, 133.2, 133.0, 129.9, 129.8, 129.8, 129.1, 128.6, 127.7, 127.6, 127.3, 126.1, 124.0, 123.3, 120.0.

Melting point: 142-148 °C

NMR data of **2n** matched those reported in the literature.

2n': ^{1}**H NMR (400 MHz, CDCl$_3$)** δ 11.03 (s, 1H), 8.52 (s, 1H), 8.35 – 8.30 (m, 1H), 8.16 (d, *J* = 8.8 Hz, 1H), 8.11 – 8.07 (m, 2H), 8.01 (d, *J* = 9.0 Hz, 1H), 7.79 (d, *J* = 8.8 Hz, 1H), 7.65 – 7.57 (m, 2H). ^{13}**C NMR (101 MHz, CDCl$_3$)** δ 155.5, 153.8, 133.4, 132.9, 132.9, 131.9, 131.3, 131.1, 129.7, 128.3, 128.2, 127.9, 127.1, 126.5, 126.4, 126.1, 122.6, 120.4.

NMR data of **2n'** matched those reported in the literature.

Melting point: 197-202 °C

4-chloro[4]pyridohelicene (**2o**)

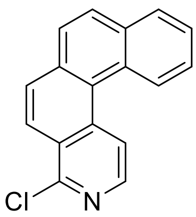

1o was subjected to general procedure C on a scale of 0.200 mmol and purified by column chromatography (1:9 EtOAc:hexane) to give product **2o** as a white solid in 74% yield (39.2 mg, 0.149 mmol).

^{1}**H NMR (400 MHz, CDCl$_3$)** δ 8.99 (d, *J* = 8.4 Hz, 1H), 8.85 (d, *J* = 5.9 Hz, 1H), 8.49 (d, *J* = 5.9 Hz, 1H), 8.44 (d, *J* = 8.8 Hz, 1H), 8.09 – 8.00 (m, 3H), 7.87 (d, *J* = 8.6 Hz, 1H), 7.79 – 7.67 (m, 2H). ^{13}**C NMR (101 MHz, CDCl$_3$)** δ 152.2, 142.7, 136.4, 133.8, 133.6, 130.4, 130.2, 129.8, 129.1, 127.5, 127.3, 126.9, 126.6, 126.5, 125.9, 124.3, 120.8.

HRMS (ASAP, Orbitrap) m/z: $[M+H]^+$ calcd. for $C_{17}H_{11}ClN$ 264.0575; Found 264.0582.

Melting point: 144-146 °C.

[7]Thienohelicene (**2p**) and naphtho[1',2':8,9]tetrapheno[2,1-*b*]thiophene (**S2p'**)

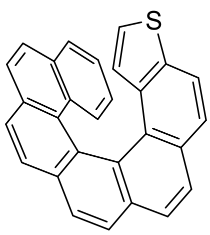

2p

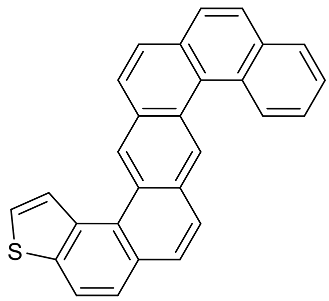

S2p'

A solution of the previously reported mixture of **1p** (122 mg, 95% purity) (corrected to 116 mg, 0.301 mmol, 1.00 equiv.), iodine (88.0 mg, 0.347 mmol, 1.15 equiv.) and THF (510 µL, 6.30 mmol, 21.0 equiv.) in toluene (160 mL, 1.9 mM) was run through a Vapourtec E-series flow reactor fitted with a 150 W 365 nm LED lamp at 80 °C and 3.3 mL/min with a reactor coil volume of 10 mL. The product mixture was concentrated *in vacuo* and the crude product was purified by column chromatography (9:1 hexane:toluene) to afford an unseparated mixture of **2p** and **S2p'**. The mixture was purified by recycling GPC in chloroform to afford **2p** as a yellow solid in 56% yield (65.0 mg, 0.170 mmol) and **S2p'** as a yellow solid in 18% yield (21.0 mg, 0.055 mmol).

2p: ^{1}**H NMR (400 MHz, CDCl$_3$)** δ 8.09 – 7.98 (m, 5H), 7.95 (d, *J* = 8.3 Hz, 1H), 7.87 (d, *J* = 8.5 Hz, 1H), 7.79 (d, *J* = 8.4 Hz, 1H), 7.71 (d, *J* = 8.5 Hz, 1H), 7.71 (dd, *J* = 8.4, 0.7 Hz, 1H), 7.51 (dd, *J* = 8.0, 1.2 Hz,

1H), 7.21 (d, *J* = 8.5 Hz, 1H), 7.02 (ddd, *J* = 8.0, 6.9, 1.1 Hz, 1H), 6.55 (d, *J* = 5.6 Hz, 1H), 6.45 (ddd, *J* = 8.4, 6.9, 1.4 Hz, 1H), 6.23 (dd, *J* = 5.6, 0.7 Hz, 1H). **^{13}C NMR (101 MHz, $CDCl_3$)** δ 138.2, 135.6, 132.6, 132.2, 131.8, 130.8, 130.0, 129.4, 128.7, 128.1, 127.7, 127.6, 127.5, 127.1, 126.9, 126.9, 126.8, 126.1, 125.7, 125.5, 125.5, 124.9, 124.8, 124.4, 123.5, 123.5, 123.2, 121.1.

HRMS (APCI$^+$, Orbitrap) calculated for $C_{28}H_{17}S$ [M+H]$^+$ 385.1051, found 385.1033.

Melting point: 243-248 °C

S2p': **^{1}H NMR (400 MHz, $CDCl_3$)** δ 9.66 (s, 2H), 9.35 (d, *J* = 8.5 Hz, 1H), 8.85 (d, *J* = 5.5 Hz, 1H), 8.10 – 8.13 (m, 2H), 8.10 (dd, *J* = 8.0, 1.3 Hz, 1H), 8.05 – 7.98 (m, 2H), 7.93 – 7.84 (m, 5H), 7.79 (ddd, *J* = 8.5, 6.9, 1.4 Hz, 1H), 7.69 (ddd, *J* = 7.9, 6.9, 1.1 Hz, 1H). **^{13}C NMR (101 MHz, $CDCl_3$)** δ 140.3, 135.6, 133.7, 132.0, 131.4, 131.2, 130.6, 130.4, 129.1, 128.9, 128.8, 128.3, 128.1, 127.9, 127.8, 127.7, 127.3, 127.2, 127.2, 127.2, 127.0, 126.8, 126.6, 126.2, 126.0, 125.7, 125.4, 122.0.

HRMS (APCI$^+$, Orbitrap) calculated for $C_{28}H_{17}S$ [M+H]$^+$ 385.1051, found 385.1036.

Melting point: 197-201 °C

Dibromo[7]pyrrolohelicene (**2q**)

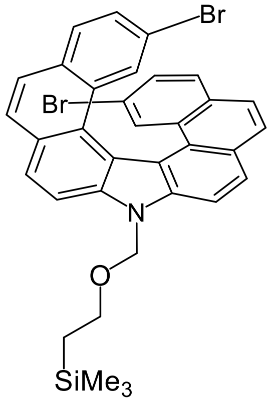

A solution of **1q** (213 mg, 0.323 mmol, 1.00 equiv.) and iodine (8.2 mg, 0.0325 mmol, 0.1 equiv.) in toluene:THF (9:1, 323 mL, 1.0 mM) was run through a Vapourtec E-series flow reactor fitted with a 150 W 365 nm LED lamp at 55 °C and 6 mL/min with a reactor coil volume of 10 mL. The product mixture was concentrated *in vacuo* and purified by column chromatography (1:10 EtOAc:hexane) to afford **2q** as a yellow solid in 50% yield (110 mg, 0.160 mmol). The reaction was repeated on a larger 1.00 mmol scale to afford **2q** in 50% yield (327 mg, 0.500 mmol).

^{1}H NMR (400 MHz, $CDCl_3$) δ 8.17 – 8.05 (m, 6H), 7.91 (d, *J* = 8.5 Hz, 2H), 7.76 (d, *J* = 8.5 Hz, 2H), 7.45 (d, *J* = 1.9 Hz, 2H), 7.33 (dd, *J* = 8.5, 2.0 Hz, 2H), 6.12 (s, 2H), 3.71 (dd, *J* = 8.4, 7.8 Hz, 2H), 0.98 (td, *J* = 7.7, 1.7 Hz, 2H), -0.08 (s, 9H). **^{13}C NMR (101 MHz, $CDCl_3$)** δ 139.8, 130.9, 130.8, 130.2, 129.1, 128.6, 127.7, 127.3, 127.3, 125.6, 124.5, 117.1, 116.9, 110.6, 72.9, 66.7, 18.0, -1.3.

HRMS (ES, TOF) calculated for $C_{34}H_{29}Br_2NOSiNa$ [M+Na]$^+$ 676.0283, found 676.0274.

(*E*)-2-styrylphenanthro[3,4-*b*]thiophene (**3a**)[19]

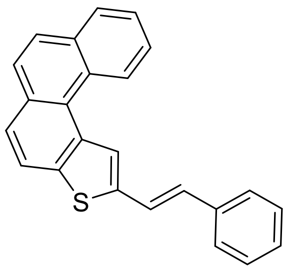

To a Schlenk tube with a stirrer bar under nitrogen was added **2b** (806 mg, 3.00 mmol, 1 equiv.), NHC-Pd(II)-Im[7] (Table S1) (19.4 mg, 0.003 mmol, 1 mol%), Cs_2CO_3 (2.54 g, 7.80 mmol, 2.6 equiv.), TBAB (7.95 g, 24.8 mmol, 8.27 equiv.) and styrene (0.41 mL, 3.60 mmol, 1.2 equiv.). The Schlenk tube was sealed and stirred at 140 °C for 18 h. The resulting mixture was extracted with EtOAc and washed with $HCl_{(aq)}$ and brine, then the organic layers were collected, dried under Na_2SO_4 and concentrated *in vacuo*. The crude was purified by column chromatography (hexane) to give product **3a** as a yellow solid in 51% yield (519 mg, 1.54 mmol).

^{1}H NMR (400 MHz, $CDCl_3$) δ 9.10 (dd, J = 8.5, 0.4 Hz, 1H), 8.51 (s, 1H), 7.99 (dd, J = 7.9, 1.4 Hz, 1H), 7.95 (dd, J = 8.4, 0.7 Hz, 1H), 7.80 (dt, J = 8.5, 6.9 Hz, 3H), 7.74 (ddd, J = 8.5, 6.9, 1.5 Hz, 1H), 7.66 (ddd,

J = 8.0, 7.0, 1.1 Hz, 1H), 7.59 – 7.55 (m, 2H), 7.51 (dd, J = 16.0, 0.5 Hz, 1H), 7.45 – 7.38 (m, 2H), 7.35 – 7.29 (m, 1H), 7.10 (d, J = 16.0 Hz, 1H). **^{13}C NMR (101 MHz, $CDCl_3$)** δ 143.2, 139.5, 136.8, 135.5, 133.2, 130.7, 130.7, 130.6, 128.9, 128.8, 128.1, 127.6, 126.7, 126.6, 126.4, 126.4, 126.4, 126.2, 126.2, 125.4, 122.7, 121.3.

HRMS (ASAP, QTOF) m/z: $[M+H]^+$ calcd. for $C_{24}H_{17}S$ 337.1051; Found 337.1041.

Melting point: 141-144 °C.

The procedure was adapted from a previous report.

[6]Thienohelicene (**4a**)

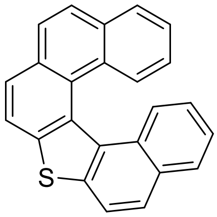

3a was subjected to general procedure B on a scale of 0.200 mmol and purified by column chromatography (1:19 EtOAc:Hexane) to give product **4a** as a yellow solid in 80% yield (46.7 mg, 0.160 mmol).

^{1}H NMR (400 MHz, $CDCl_3$) δ 8.10 – 8.06 (m, 1H), 8.03 (dt, J = 8.6, 2.0 Hz, 2H), 7.98 (dd, J = 8.1, 0.9 Hz, 2H), 7.94 (ddd, J = 14.0, 8.8, 6.4 Hz, 4H), 7.58 (d, J = 8.5 Hz, 1H), 7.50 (ddd, J = 8.0, 7.0, 1.1 Hz, 1H), 7.42 (ddd, J = 8.0, 6.9, 1.1 Hz, 1H), 7.04 (dddd, J = 8.4, 7.0, 5.7, 1.3 Hz, 2H). **^{13}C NMR (101 MHz, $CDCl_3$)** δ 139.8, 138.8, 132.3, 132.0, 131.5, 131.1, 129.7, 129.6, 129.5, 129.2, 128.3, 128.1, 128.1, 127.8, 127.5, 126.8, 126.7, 126.7, 126.5, 125.0, 124.8, 124.8, 121.2, 121.0.

HRMS (ASAP, QTOF) m/z: $[M+H]^+$ calcd. for $C_{24}H_{15}S$ 335.0894; Found 335.0892.

Melting point: 209-217 °C.

(*E*)-4,4,5,5-tetramethyl-2-(2-(thiophen-2-yl)vinyl)-1,3,2-dioxaborolane (**S5c**)[20]

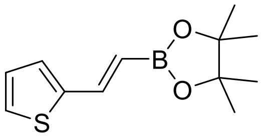

To a Schlenk tube with a stirrer bar was added with B_2Pin_2 (1.27 g, 5.00 mmol, 1.00 equiv.), -625 mesh copper powder (31.8 mg, 0.5 mmol, 10 mol%) and NaOMe (135 mg, 2.50 mmol, 0.500 equiv.). The vessel was evacuated under vacuum and refilled with nitrogen three times. EtOH (20 mL) was sparged with nitrogen and added to the vessel, followed by 2-ethynylthiophene (0.47 mL, 5.00 mmol, 1.00 equiv.). The Schlenk tube was sealed and stirred at 40 °C for 24 h. The reaction was diluted with water (10 mL) and the crude was extracted with DCM (2 x 20 mL). The combined organic layers were dried over $MgSO_4$, concentrated *in vacuo* and purified by column chromatography (Hexane) to give the title compound as a brown solid in 45% yield (531 mg, 2.25 mmol).

1H NMR (400 MHz, $CDCl_3$) δ 7.47 (d, J = 18.1 Hz, 1H), 7.24 (dt, J = 5.0, 0.8 Hz, 1H), 7.09 – 7.07 (m, 1H), 6.99 (dd, J = 5.1, 3.6 Hz, 1H), 5.91 (d, J = 18.1 Hz, 1H), 1.30 (s, 12H). **^{13}C NMR (101 MHz, $CDCl_3$)** δ 144.0, 141.9, 127.8, 127.7, 126.4, 83.4, 24.9.

HRMS (ASAP, QTOF) m/z: $[M]^{+\cdot}$ calcd. for $C_{12}H_{17}BO_2S$ 235.1079; Found 235.1083.

Melting point: 48-54 °C.

The procedure was adapted from a previous report.

(*Z*)-2-(2-(thiophen-2-yl)vinyl)phenanthro[3,4-*b*]thiophene (**3b**)[21]

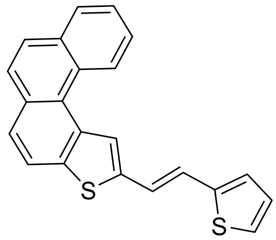

To a Schlenk tube with a stirrer bar was added $Pd(OAc)_2$ (16.8 mg, 0.075 mmol, 5 mol%), XPhos (42.9 mg, 0.09 mmol, 6 mol%), **2b** (403 mg, 1.50 mmol, 1.00 equiv.) and **S5c** (425 mg, 1.80 mmol, 1.20 equiv.). The vessel was evacuated under vacuum and refilled with nitrogen three times. Nitrogen sparged *n*-BuOH (8.4 mL) was added to the reaction vessel and the reaction was pre-stirred at room temperature for 15 min. $CsOH{\cdot}H_2O$ (0.25 mL, 1.68 equiv., 50 wt.% in water) was added to the reaction vessel. The Schlenk tube was sealed and stirred at 45 °C for 18 h. The reaction was diluted with water (10 mL) and extracted with DCM (2 x 10 mL). The combined organic layers were dried over $MgSO_4$, concentrated *in vacuo*, purified by column chromatography (1:19 EtOAc:Hexane) and triturated in Et_2O and hexane to give **3b** as a yellow solid in 80% yield (409 mg, 1.19 mmol).

^{1}H NMR (400 MHz, $CDCl_3$) δ 9.09 (d, J = 8.5 Hz, 1H), 8.49 (s, 1H), 7.98 (dd, J = 7.9, 1.4 Hz, 1H), 7.95 (dd, J = 8.4, 0.7 Hz, 1H), 7.84 – 7.78 (m, 2H), 7.79 (d, J = 8.5 Hz, 1H), 7.74 (ddd, J = 8.5, 6.9, 1.5 Hz, 1H), 7.65 (ddd, J = 8.0, 7.0, 1.1 Hz, 1H), 7.33 (d, J = 15.7 Hz, 1H), 7.26 (d, J = 5.0 Hz, 1H), 7.22 (d, J = 15.8 Hz, 1H), 7.14 (d, J = 3.5 Hz, 1H), 7.05 (dd, J = 5.1, 3.6 Hz, 1H). **^{13}C NMR (101 MHz, $CDCl_3$)** δ 142.7, 142.3, 139.5, 135.6, 133.2, 130.8, 130.6, 128.9, 128.0, 127.6, 126.9, 126.6, 126.5, 126.5, 126.4, 126.3, 126.3, 125.3, 125.2, 123.8, 122.3, 121.3.

HRMS (ASAP, QTOF) m/z: $[M+H]^+$ calcd. for $C_{22}H_{15}S_2$ 343.0615; Found 343.0608.

Melting point: 168-169 °C.

The procedure was adapted from a previous report.

[6]Dithienohelicene (**4b**)

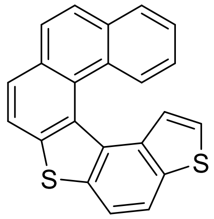

3b was subjected to general procedure B on a scale of 0.200 mmol and purified by column chromatography (1:19 EtOAc:Hexane) to give product **4b** as a yellow solid in 87% yield (59.4 mg, 0.174 mmol).

^{1}H NMR (400 MHz, $CDCl_3$) δ 8.28 (dd, J = 8.4, 0.4 Hz, 1H), 8.05 – 8.01 (m, 2H), 8.00 – 7.95 (m, 2H), 7.93 – 7.89 (m, 3H), 7.55 (ddd, J = 8.0, 7.0, 1.1 Hz, 1H), 7.23 – 7.16 (m, 2H), 6.72 (d, J = 5.5 Hz, 1H). **^{13}C NMR (101 MHz, $CDCl_3$)** δ 139.8, 137.3, 137.2, 135.1, 132.4, 132.1, 130.9, 129.5, 129.5, 128.9, 128.0, 127.5, 127.1, 126.9, 126.7, 126.7, 126.5, 125.0, 124.1, 121.2, 121.1, 119.2.

HRMS (ASAP, QTOF) m/z: $[M+H]^+$ calcd. for $C_{22}H_{13}S_2$ 341.0459; Found 341.0449.

Melting point: 201-205 °C.

References

(1) Still, W. C.; Kahn, M.; Mitra, A. Rapid Chromatographic Technique for Preparative Separations with Moderate Resolution. *J Org Chem* **1978**, *43* (14), 2923–2925. https://doi.org/10.1021/jo00408a041.

(2) Klimash, A.; Pander, P.; Klooster, W. T.; Coles, S. J.; Data, P.; Dias, F. B.; Skabara, P. J. Intermolecular Interactions in Molecular Crystals and Their Effect on Thermally Activated Delayed Fluorescence of Helicene-Based Emitters. *J Mater Chem C Mater* **2018**, *6* (39), 10557–10568. https://doi.org/10.1039/c8tc03390k.

(3) Talele, H. R.; Chaudhary, A. R.; Patel, P. R.; Bedekar, A. V. Expeditious Synthesis of Helicenes Using an Improved Protocol of Photocyclodehydrogenation of Stilbenes. *Arkivoc* **2011**, *2011* (9), 15–37. https://doi.org/10.3998/ark.5550190.0012.902.

(4) Maya, A. B. S.; Pérez-Melero, C.; Mateo, C.; Alonso, D.; Fernández, J. L.; Gajate, C.; Mollinedo, F.; Peláez, R.; Caballero, E.; Medarde, M. Further Naphthylcombretastatins. An Investigation on the Role of the Naphthalene Moiety. *J Med Chem* **2005**, *48* (2), 556–568. https://doi.org/10.1021/jm0310737.

(5) Ichikawa, J.; Yokota, M.; Kudo, T.; Umezaki, S. Efficient Helicene Synthesis: Friedel-Crafts-Type Cyclization of 1,1-Difluoro-1-Alkenes. *Angew Chem, Int Ed* **2008**, *47* (26), 4870–4873. https://doi.org/10.1002/anie.200801396.

(6) Li, Q. Q.; Shah, Z.; Qu, J. P.; Kang, Y. B. Direct Wittig Olefination of Alcohols. *J Org Chem* **2018**, *83* (1), 296–302. https://doi.org/10.1021/acs.joc.7b02720.

(7) Zhang, N.; Quan, Z. J.; Zhang, Z.; Da, Y. X.; Wang, X. C. Synthesis of Stilbene Derivatives via Visible-Light-Induced Cross-Coupling of Aryl Diazonium Salts with Nitroalkenes Using-NO2 as a Leaving Group. *Chem Commun* **2016**, *52* (99), 14234–14237. https://doi.org/10.1039/c6cc08182g.

(8) Das, M.; OShea, D. F. Z-Stereoselective Aza-Peterson Olefinations with Bis(Trimethylsilane) Reagents and Sulfinyl Imines. *Org Lett* **2016**, *18* (2), 336–339. https://doi.org/10.1021/acs.orglett.5b03519.

(9) De, P. K.; Neckers, D. C. Sulfur Containing Stable Unsubstituted Heptacene Analogs. *Org Lett* **2012**, *14* (1), 78–81. https://doi.org/10.1021/ol2028724.

(10) Bazzini, C.; Brovelli, S.; Caronna, T.; Gambarotti, C.; Giannone, M.; Macchi, P.; Meinardi, F.; Mele, A.; Panzeri, W.; Recupero, F.; Sironi, A.; Tubino, R. Synthesis and Characterization of Some Aza[5]Helicenes. *Eur J Org Chem* **2005**, No. 7, 1247–1257. https://doi.org/10.1002/ejoc.200400648.

(11) Biet, T.; Martin, K.; Hankache, J.; Hellou, N.; Hauser, A.; Bürgi, T.; Vanthuyne, N.; Aharon, T.; Caricato, M.; Crassous, J.; Avarvari Narcis. Triggering Emission with the Helical Turn in Thiadiazole-Helicenes. *Chem – Eur J* **2017**, *23* (1), 437–446. https://doi.org/10.1002/chem.201604602.

(12) Chen, C.; Wang, X.; Xu, D.; Zhang, H.; Chan, H. N.; Zhan, Z.; Jia, S.; Song, Q.; Song, G.; Li, H. W.; Wong, M. S. Multifunctional Theranostic Carbazole-Based Cyanine for Real-Time Imaging of Amyloid-β and Therapeutic Treatment of Multiple Pathologies in Alzheimer's Disease. *J Mater Chem B* **2023**, *11* (22), 4865–4873. https://doi.org/10.1039/d3tb00082f.

(13) Li, X.; Zhang, X.; Li, W.; Wang, Y.; Liu, T.; Zhang, B.; Yang, W. Synthesis and Enhanced Two-Photon Absorption Properties of Tetradonor-Containing Anthracene-Centered 2-D Cross-Conjugated Polymers. *J Mater Chem* **2011**, 21, 3916–3924. https://doi.org/ 10.1039/c0jm04067c.

(14) Feng, X. J.; Tian, P. Z.; Xu, Z.; Chen, S. F.; Wong, M. S. Fluorescence-Enhanced Chemosensor for Metal Cation Detection Based on Pyridine and Carbazole. *J Org Chem* **2013**, *78* (22), 11318–11325. https://doi.org/10.1021/jo401808c.

(15) Iwao, M.; Lee, M. L.; Castle, R. N. Synthesis of Phenanthro[b]Thiophenes. *J Heterocycl Chem* **1980**, *17* (6), 1259–1264. https://doi.org/10.1002/jhet.5570170623.

(16) Cook, J. W.; Thomson, W. H. S. Polycyclic Aromatic Hydrocarbons. Part XXXI. Some Nitrogenous Analogues of Chrysene, Pyrene and 3 : 4-Benxphenanthrene. *J Chem Soc* **1945**, 395–399. https://doi.org/ 10.1039/jr9450000395.

(17) Dopper, J. H.; Oudman, D.; Wynberg, H. Dehydrogenation of Heterohelicenes by a Scholl Type Reaction The Dehydrohelicenes. *J Org Chem* **1975**, *40* (23), 3398–3401.

(18) Cossu, S.; De Lucchi, O.; Fabbri, D.; Valle, G.; Painter, G. F.; Smith, R. A. J. Synthesis of Structurally Modified Atropisomeric Biaryl Dithiols. Observations on the Newman-Kwart Rearrangement. Tetrahedron **1997**, *53* (17), 6073–6084. https://doi.org/10.1016/S0040-4020(97)00268-8

(19) Bronovitskaya, V. P.; Berlin, A. Y.; Sheinker, Y. N.; Bakhmedova, A. A. Phenanthrothiazoles III. Synthesis of some phenanthro[3,4-d]thiazoles and determination of the structures of phenanthrothiazoles by means of PMR spectra. *Chem Heterocycl Compd* **1971**, 7, 1389–1392. https://doi.org/10.1007/BF00481102.

(20) Lieber, E.; Rao, C. N. R.; Ramachandran, J. The Infrared Spectra of Organic Thiocyanates and Isothiocyanates. *Spectrochim Acta* **1959**, *13* (4), 296–299. https://doi.org/10.1016/0371-1951(59)80030-8.

(21) Gao, T. T.; Jin, A. P.; Shao, L. X. N-Heterocyclic Carbene-Palladium(II)-1-Methylimidazole Complex Catalyzed Mizoroki-Heck Reaction of Aryl Chlorides with Styrenes. *Beilstein J Org Chem* **2012**, *8*, 1916–1919. https://doi.org/10.3762/bjoc.8.222.

(22) Fyfe, J. W. B.; Seath, C. P.; Watson, A. J. B. Chemoselective Boronic Ester Synthesis by Controlled Speciation. *Angew Chem, Int Ed* **2014**, *53* (45), 12077–12080. https://doi.org/10.1002/anie.201406714.

(23) Yang, J.; Liu, S.; Zheng, J. F.; Zhou, J. Room-Temperature Suzuki-Miyaura Coupling of Heteroaryl Chlorides and Tosylates. *Eur J Org Chem* **2012**, 31, 6248–6259. https://doi.org/10.1002/ejoc.201200918.

NMR spectra

*^{1}H-NMR of Bromo(naphthalen-2-ylmethyl)triphenyl-λ[5]-phosphane (400 MHz, $CDCl_3$) (**S5a**)*

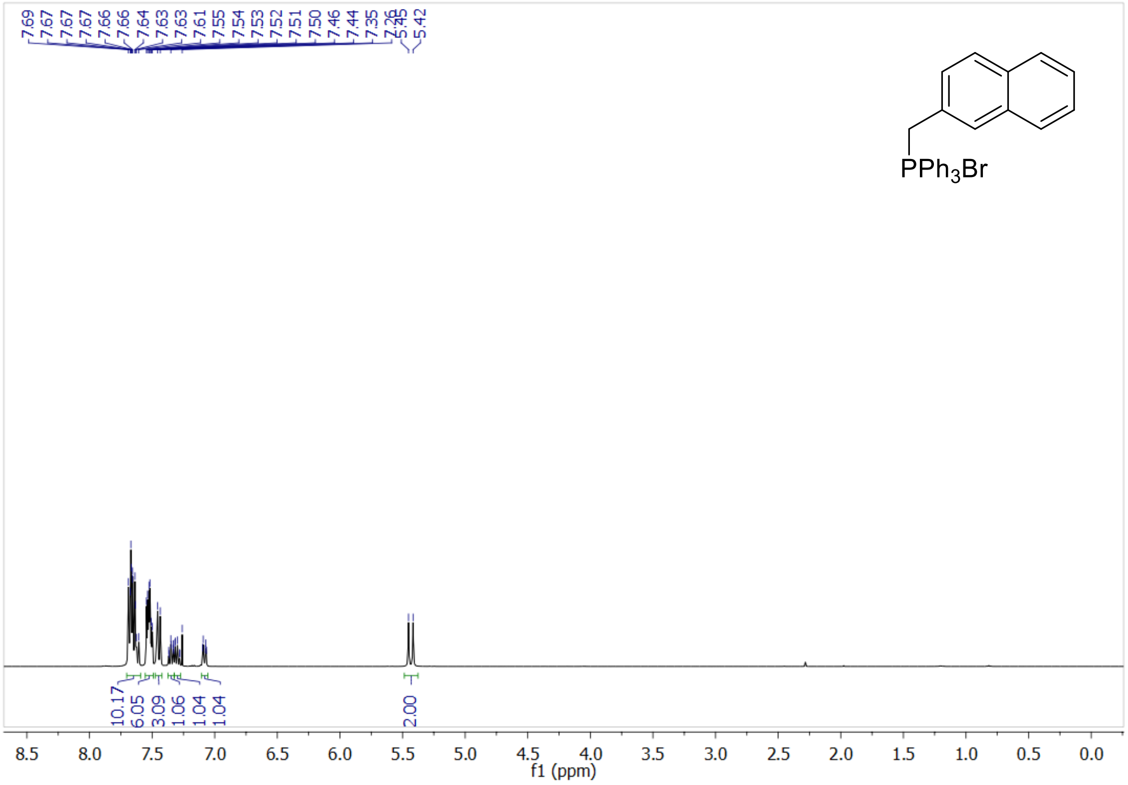

*^{13}C-NMR of Bromo(naphthalen-2-ylmethyl)triphenyl-λ[5]-phosphane (101 MHz, $CDCl_3$) (**S5a**)*

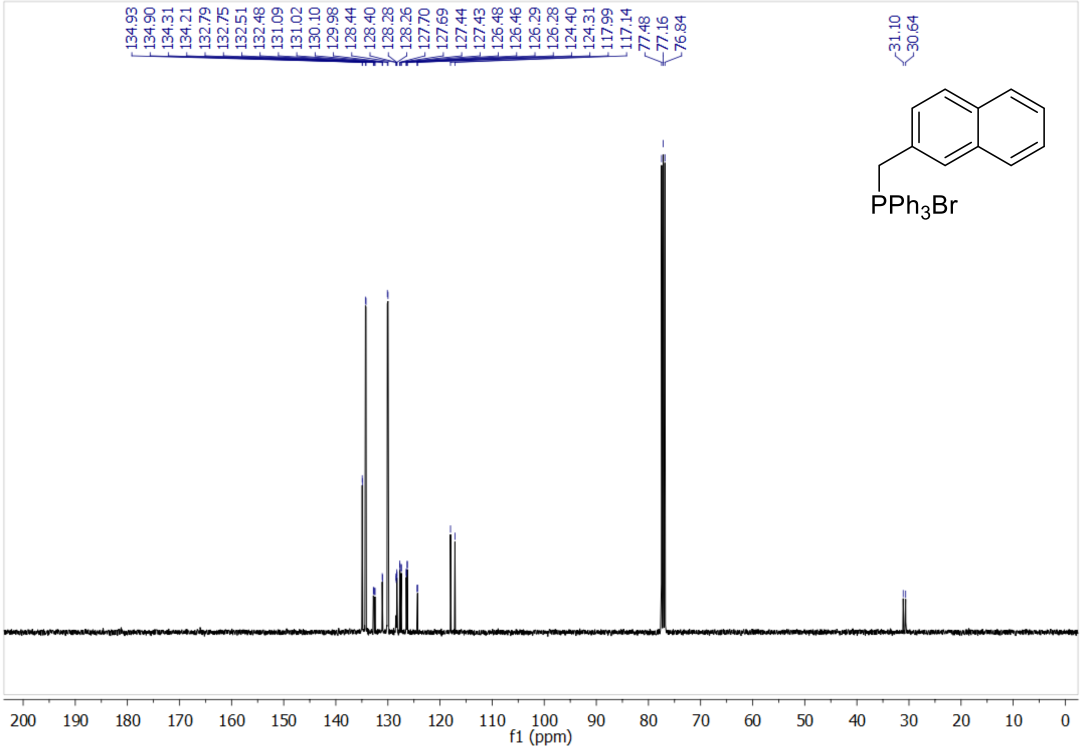

*^{31}P-NMR of Bromo(naphthalen-2-ylmethyl)triphenyl-λ[5]-phosphane (162 MHz, $CDCl_3$) (**S5a**)*

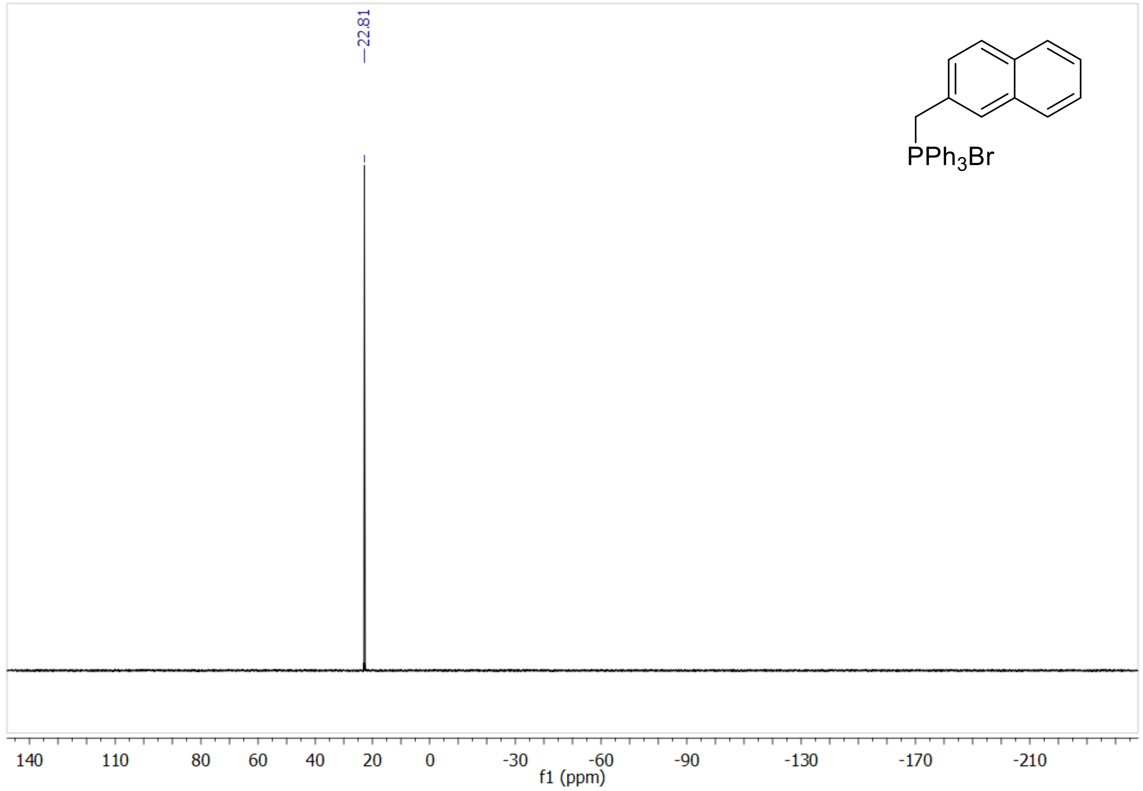

^{1}H-NMR of (Benzo[c]phenanthren-2-ylmethyl)bromotriphenyl- λ[5]-phosphane (400 MHz, $CDCl_3$) (**S5b**)

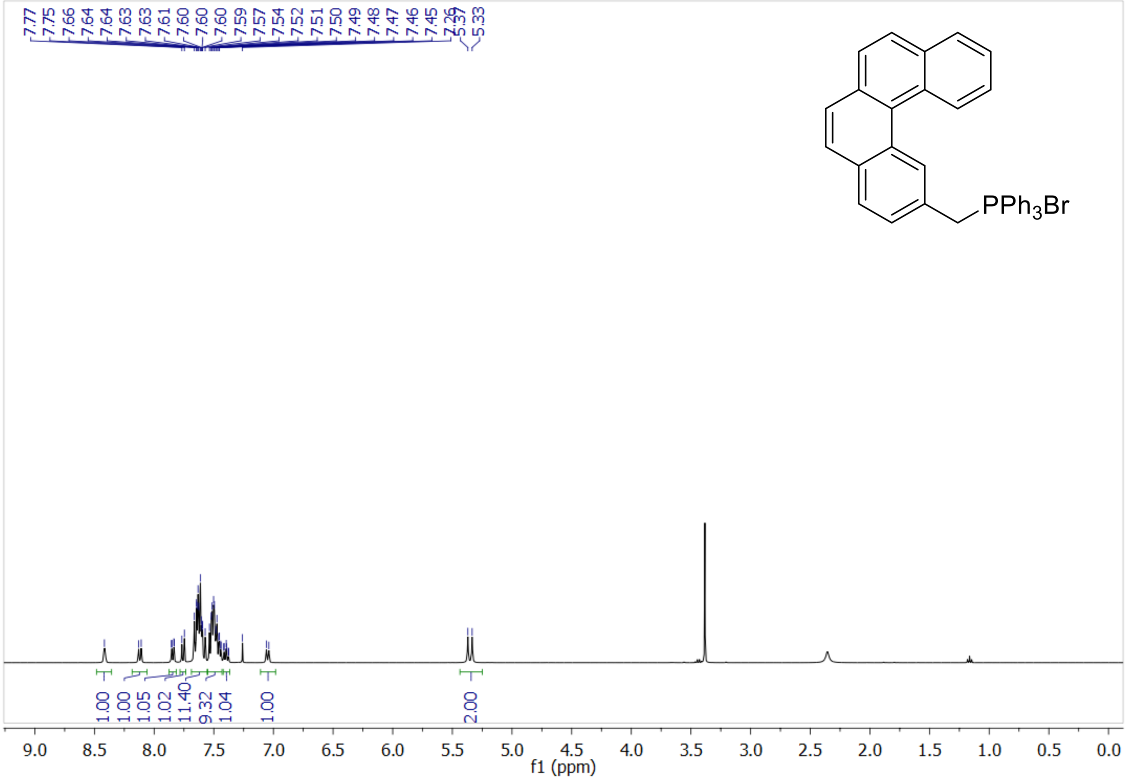

^{13}C-NMR of (Benzo[c]phenanthren-2-ylmethyl)bromotriphenyl- λ[5]-phosphane (101 MHz, $CDCl_3$) (**S5b**)

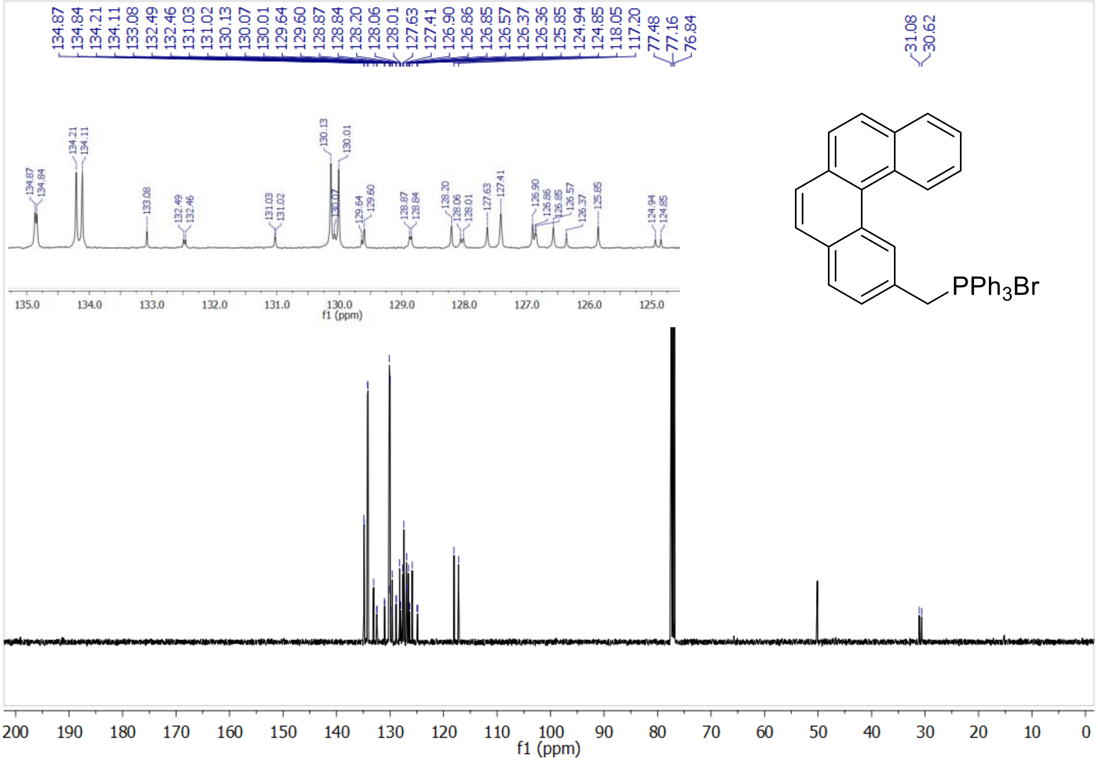

*^{31}P-NMR of (Benzo[c]phenanthren-2-ylmethyl)bromotriphenyl- λ^{5}-phosphane (162 MHz, $CDCl_3$) (**S5b**)*

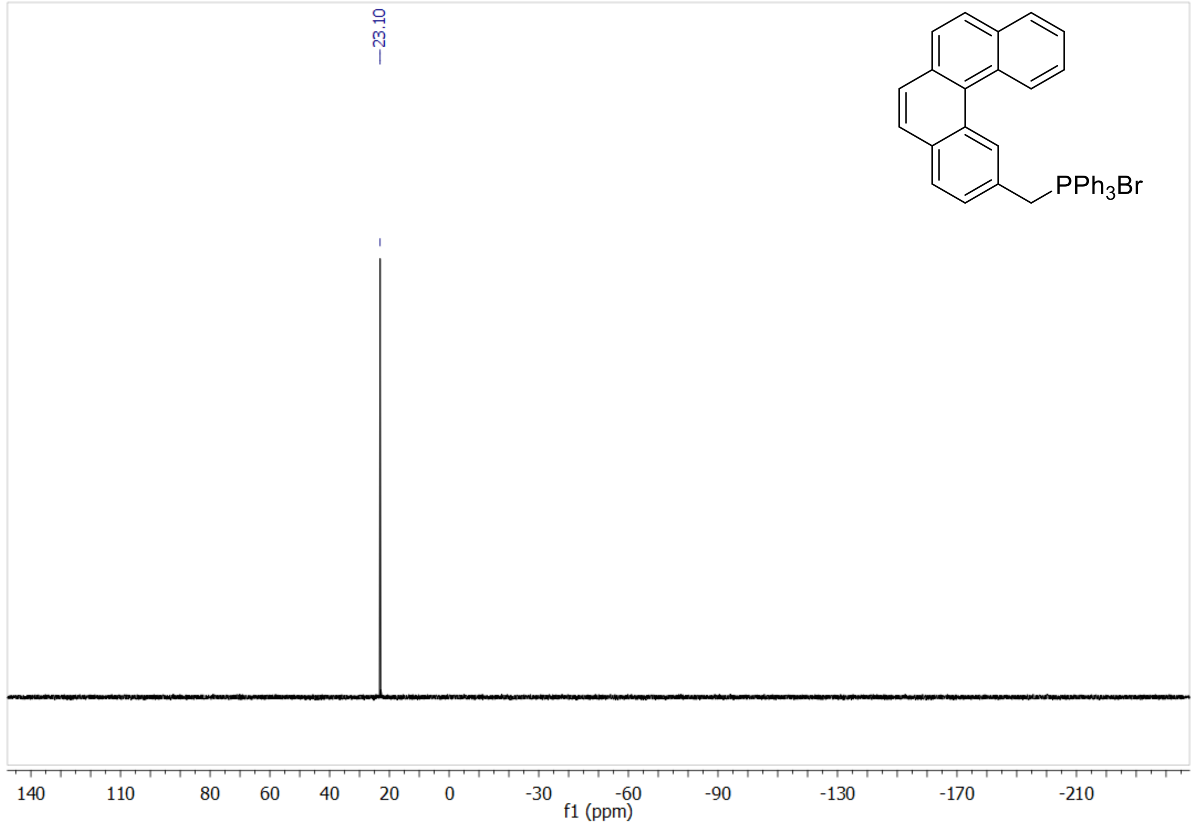

^{1}H-NMR of (E/Z)-2-(2-(naphthalen-2-yl)vinyl)thiophene (400 MHz, $CDCl_3$) (**1a**)

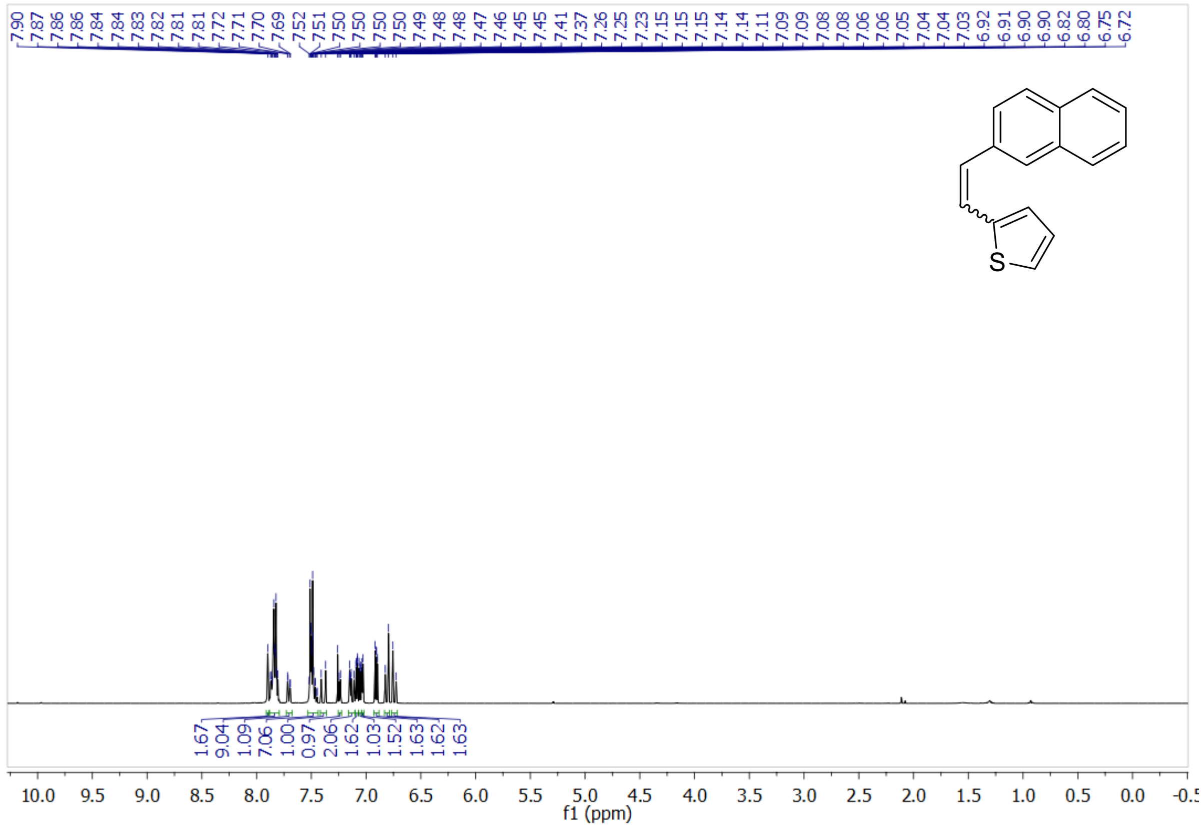

^{13}C-NMR of (E/Z)-2-(2-(naphthalen-2-yl)vinyl)thiophene (101 MHz, $CDCl_3$) (**1a**)

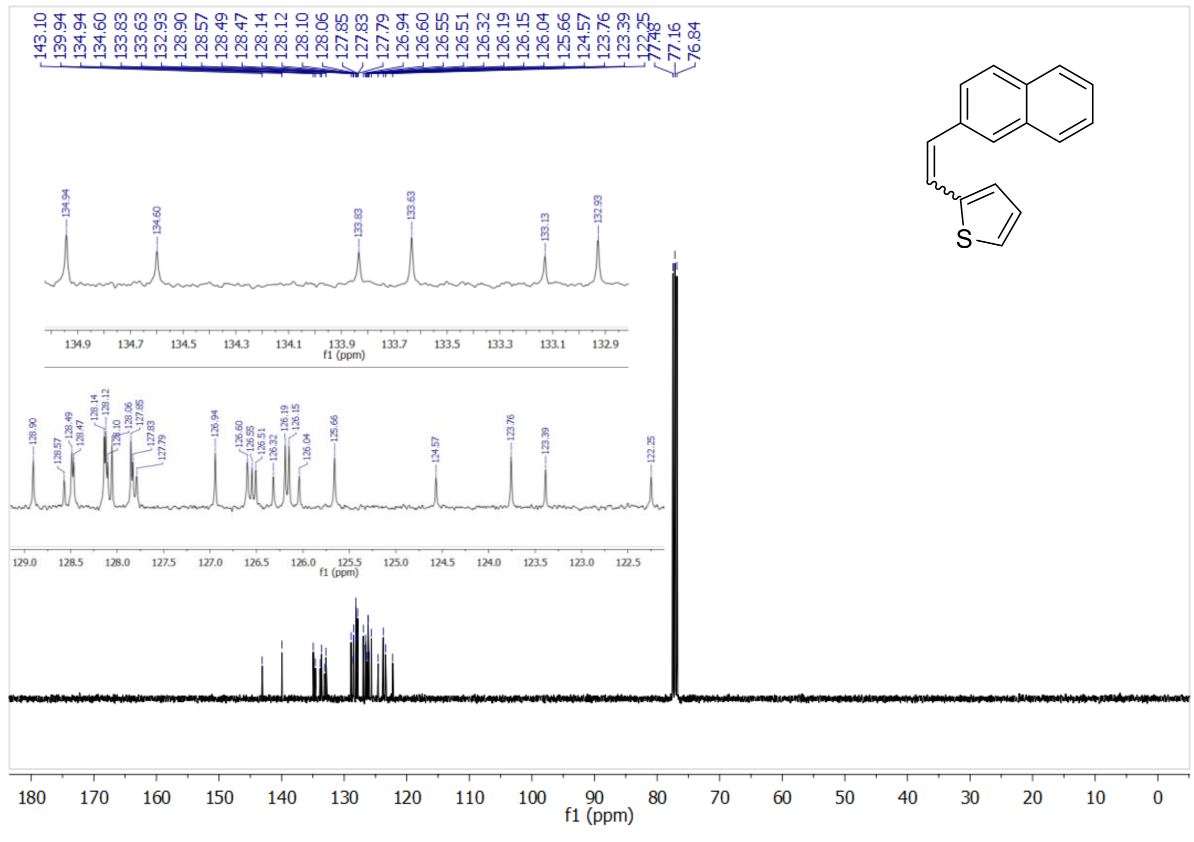

*^{1}H-NMR of (E)-2-chloro-5-(2-(naphthalen-2-yl)vinyl)thiophene (400 MHz, $CDCl_3$) (**1b**)*

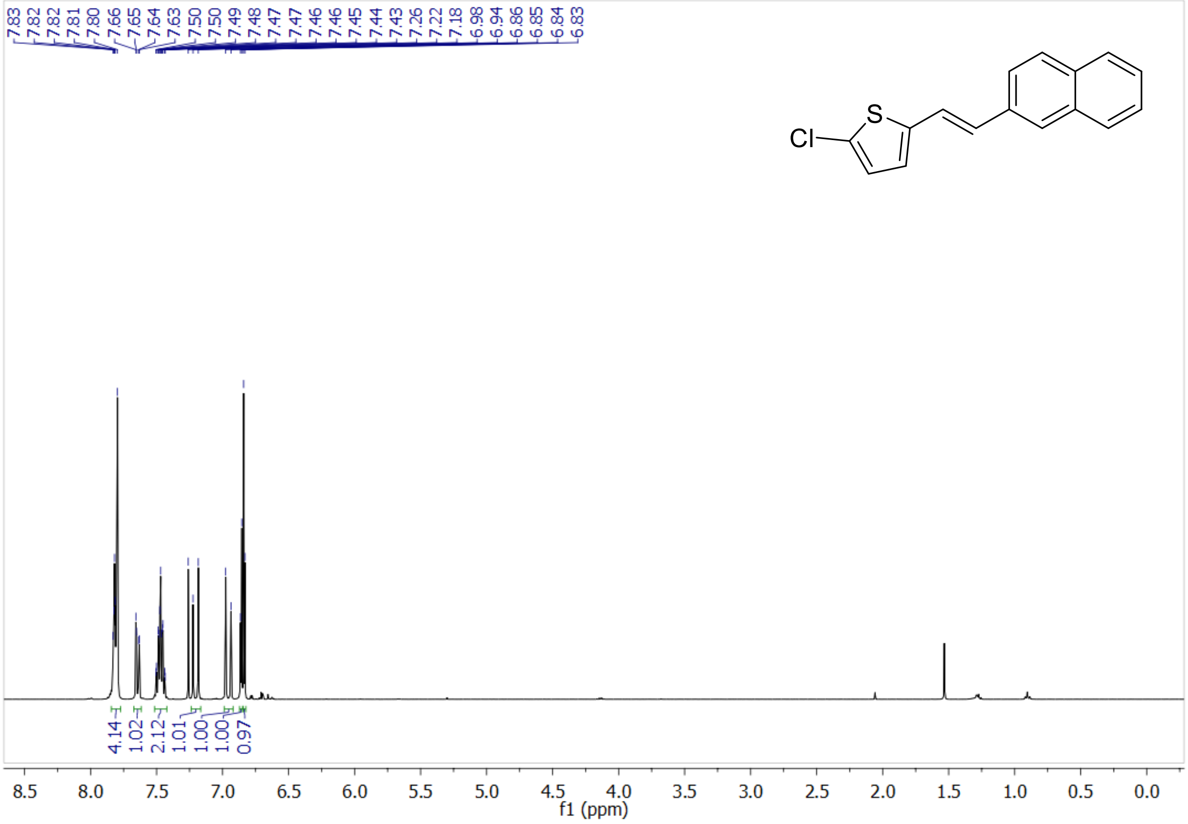

*^{13}C-NMR of (E)-2-chloro-5-(2-(naphthalen-2-yl)vinyl)thiophene (101 MHz, $CDCl_3$) (**1b**)*

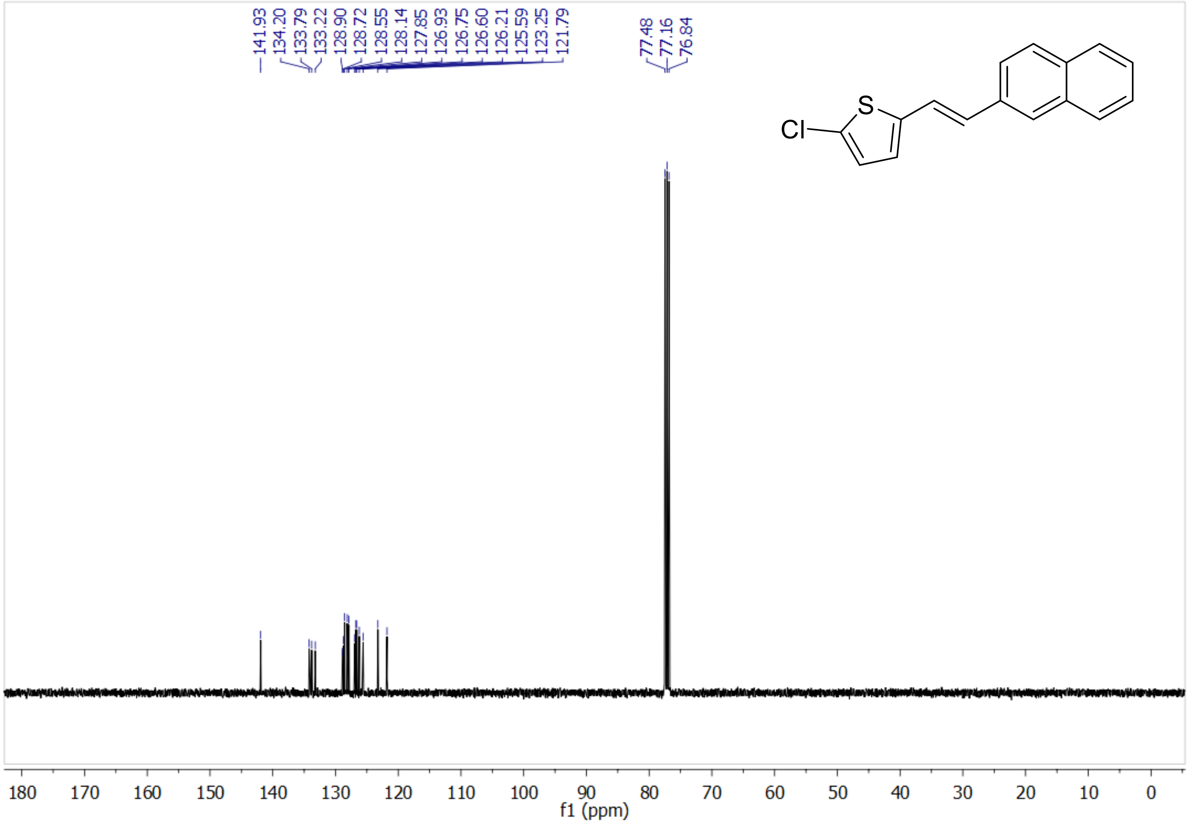

^{1}H-NMR of (E/Z)-2-chloro-5-(2-(naphthalen-2-yl)vinyl)thiophene (400 MHz, $CDCl_3$) (**1b**)

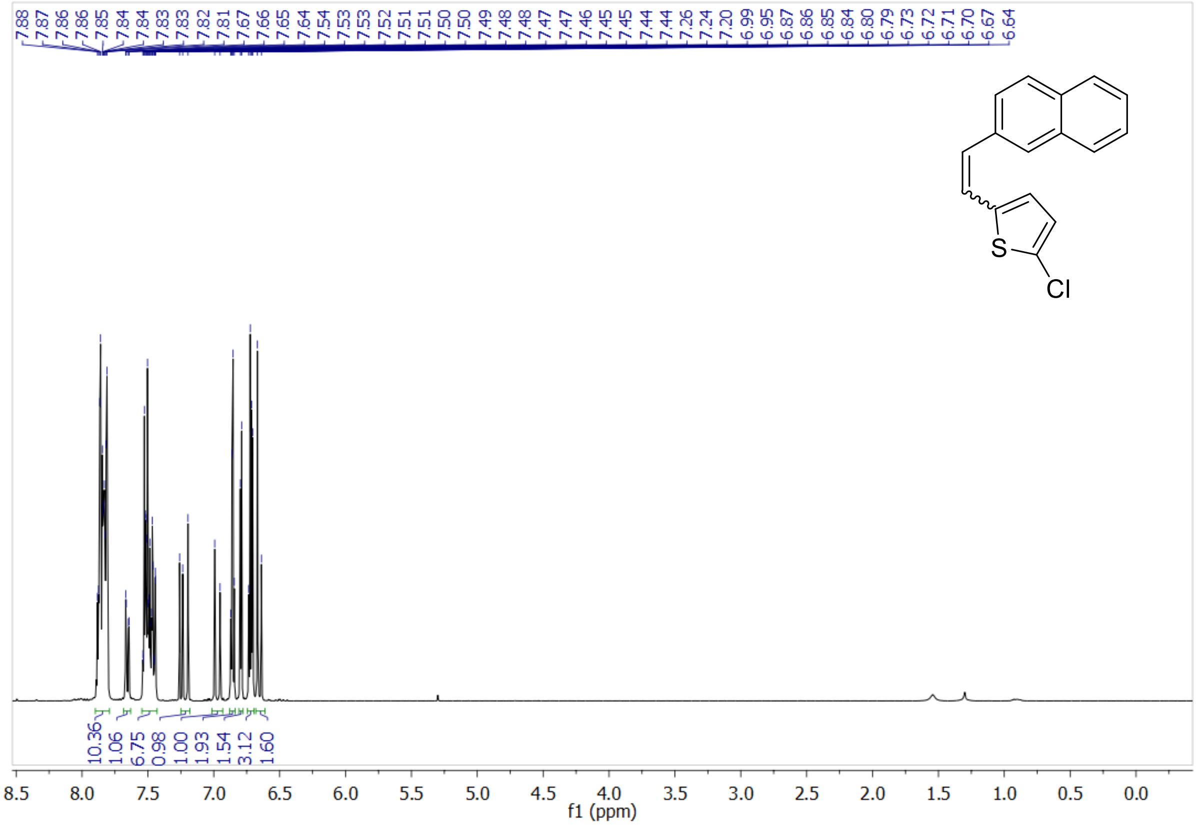

^{13}C-NMR of (E/Z)-2-chloro-5-(2-(naphthalen-2-yl)vinyl)thiophene (101 MHz, $CDCl_3$) (**1b**)

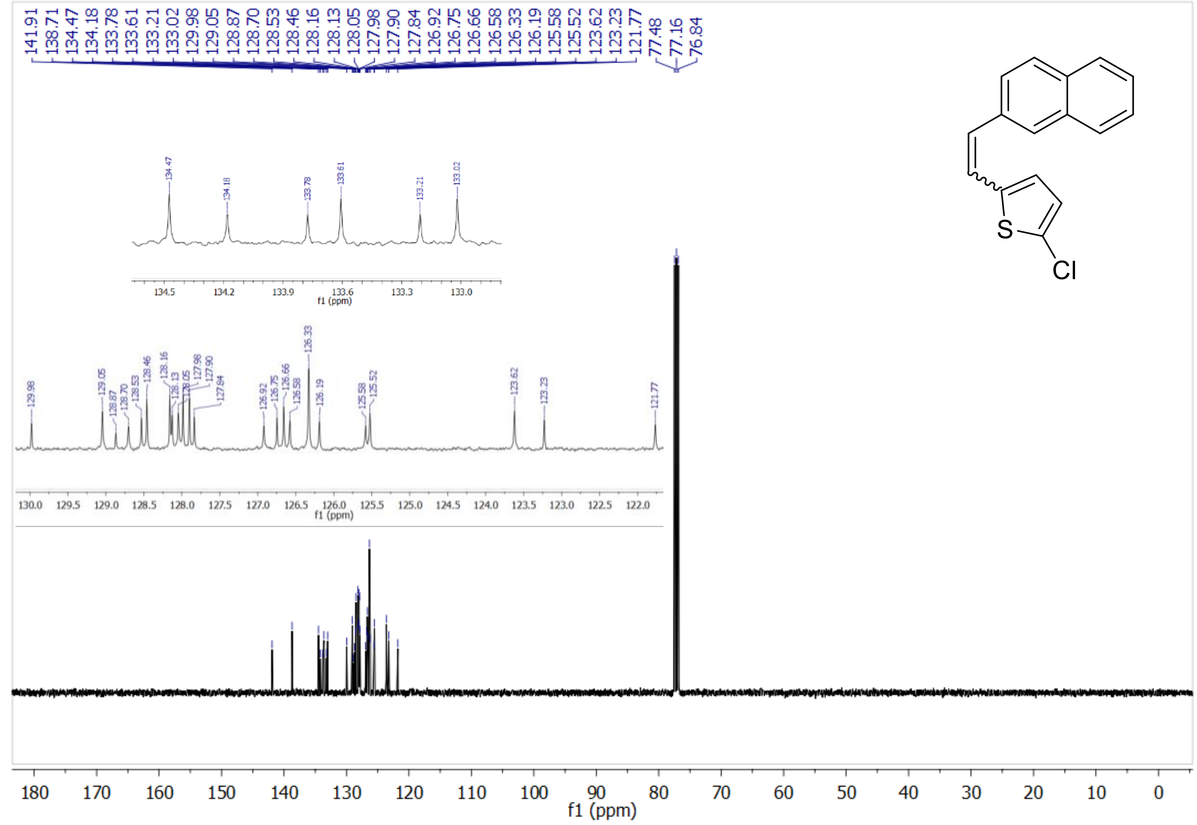

^{1}H-NMR of (E)-2-(2-(naphthalen-2-yl)vinyl)furan (400 MHz, $CDCl_3$) (**1c**)

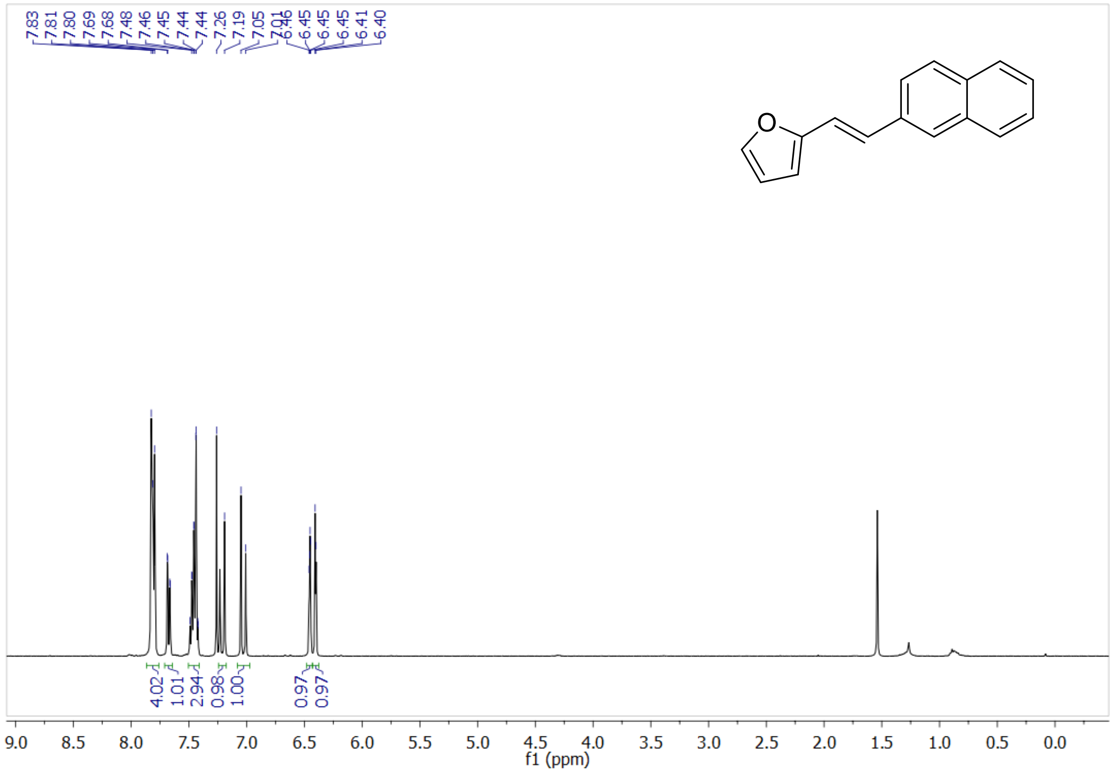

^{13}C-NMR of (E)-2-(2-(naphthalen-2-yl)vinyl)furan (101 MHz, $CDCl_3$) (**1c**)

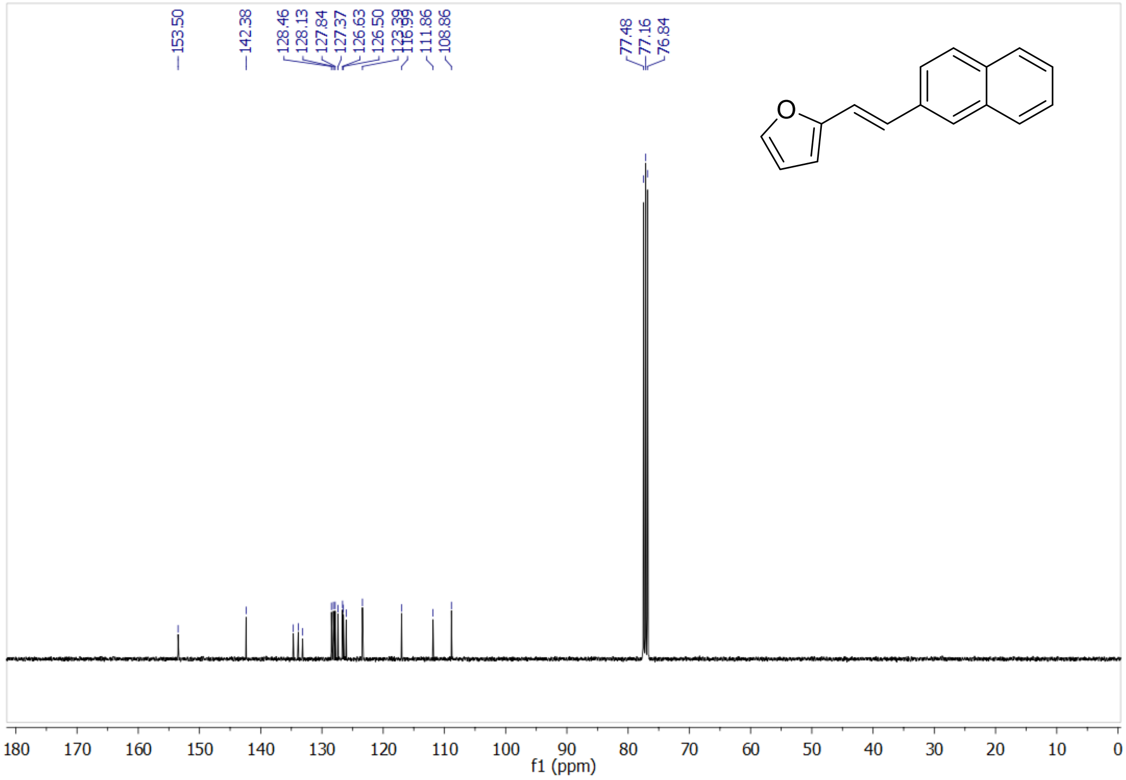

*^{1}H-NMR of (Z)-2-(2-(naphthalen-2-yl)vinyl)furan (400 MHz, $CDCl_3$) (**1c**)*

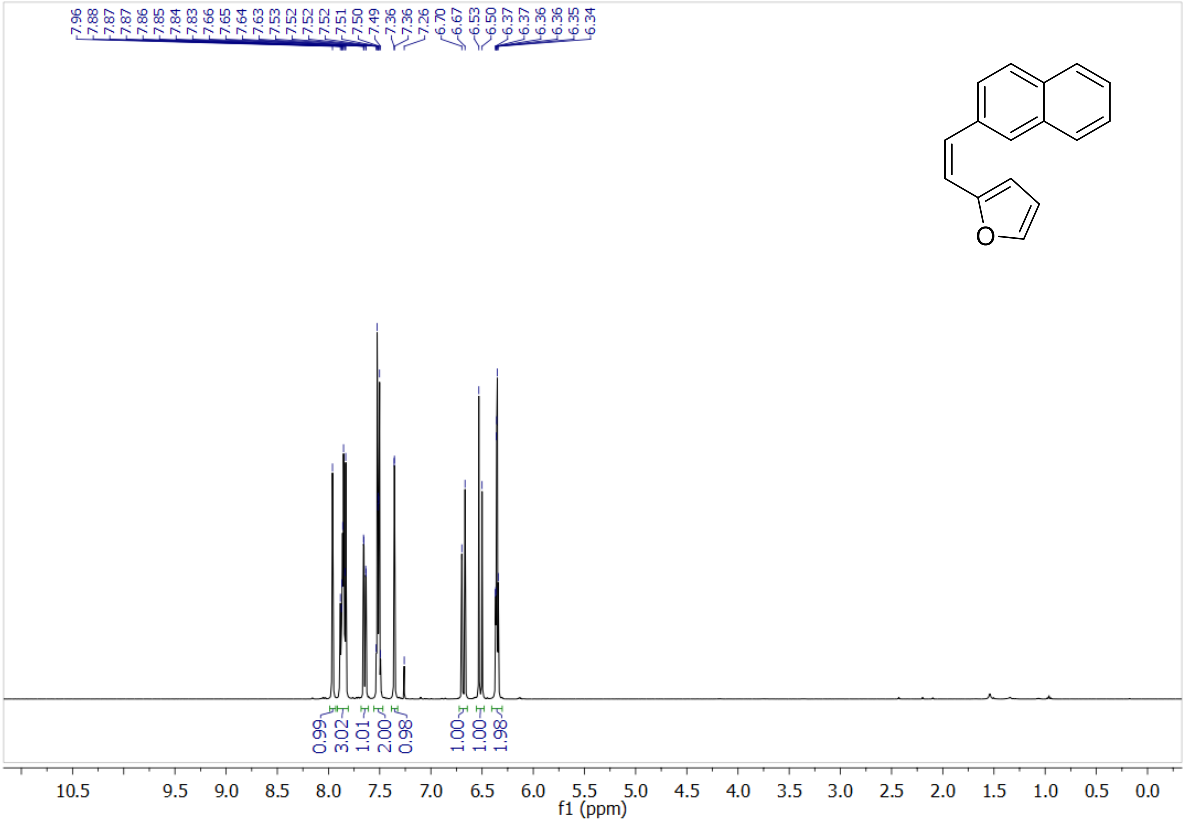

*^{13}C-NMR of (Z)-2-(2-(naphthalen-2-yl)vinyl)furan (101 MHz, $CDCl_3$) (**1c**)*

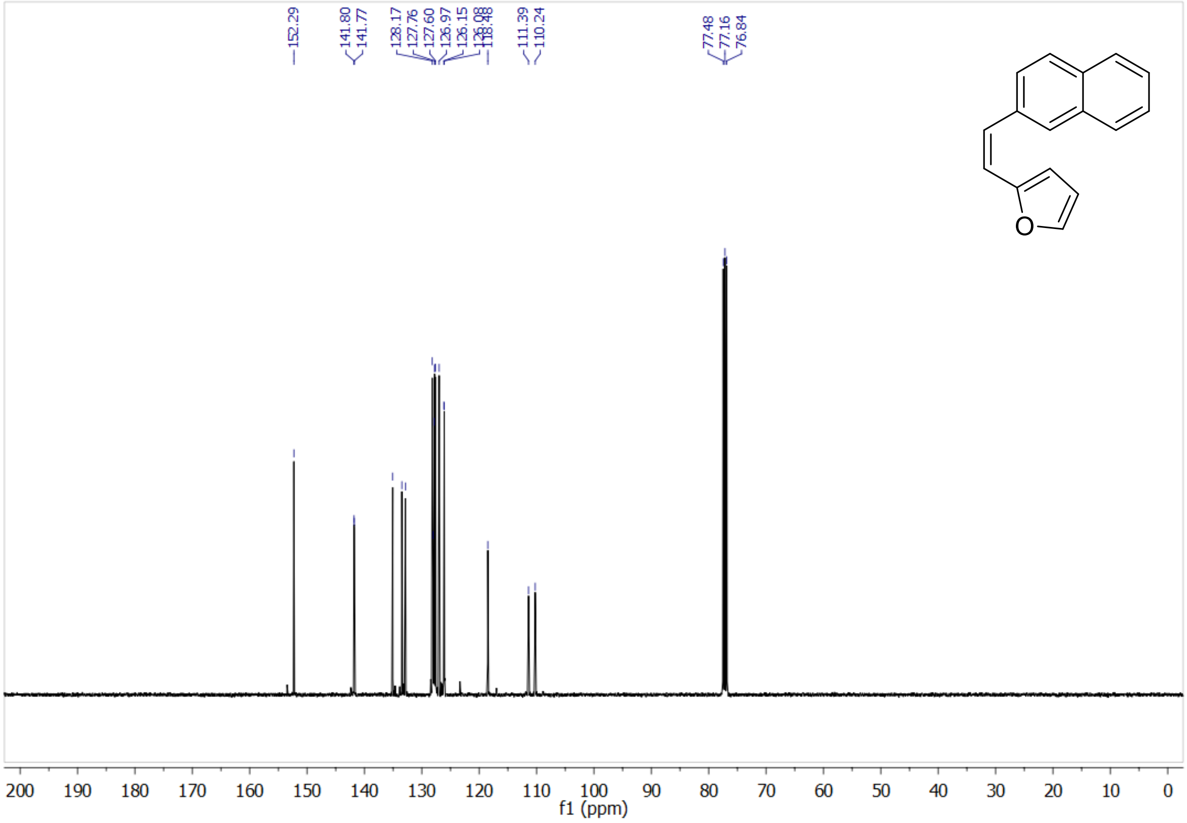

*^{1}H-NMR of (Z)-2-chloro-5-(2-(naphthalen-2-yl)vinyl)furan (400 MHz, CDCl₃) (**1d**)*

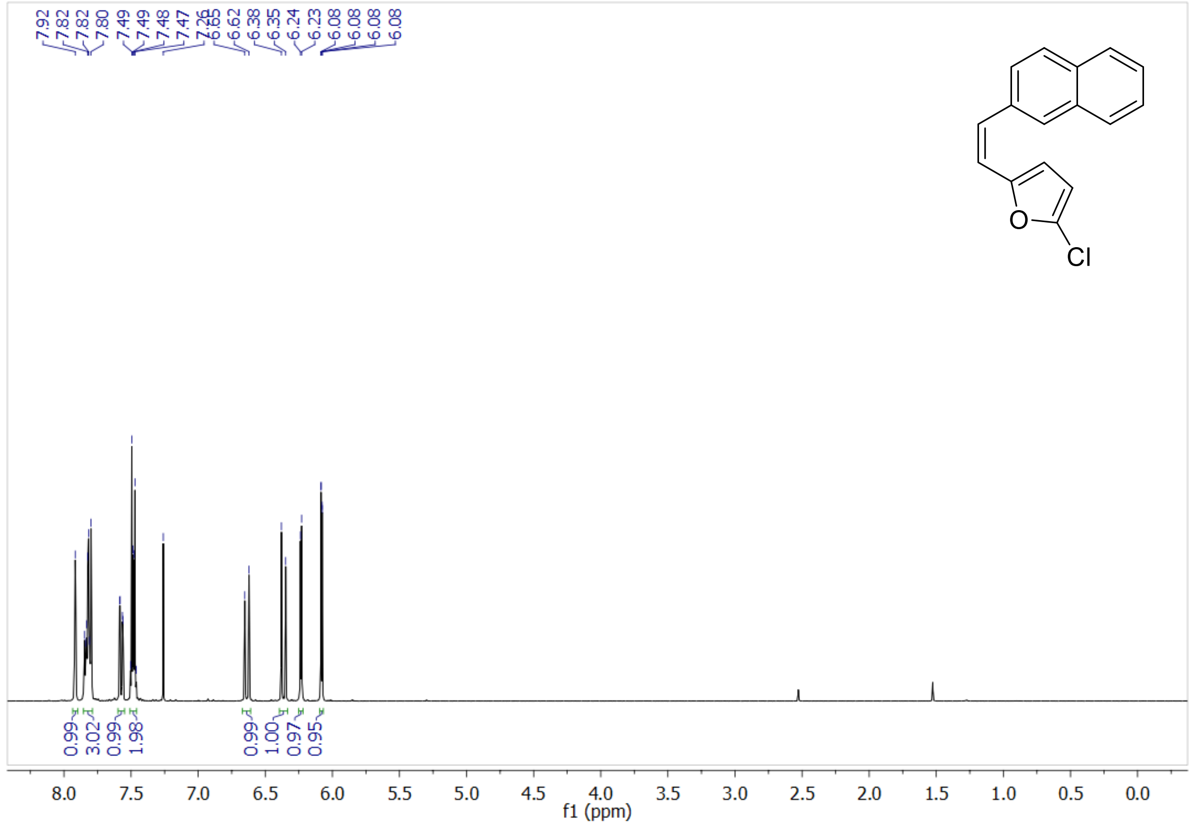

*^{13}C-NMR of (Z)-2-chloro-5-(2-(naphthalen-2-yl)vinyl)furan (101 MHz, CDCl₃) (**1d**)*

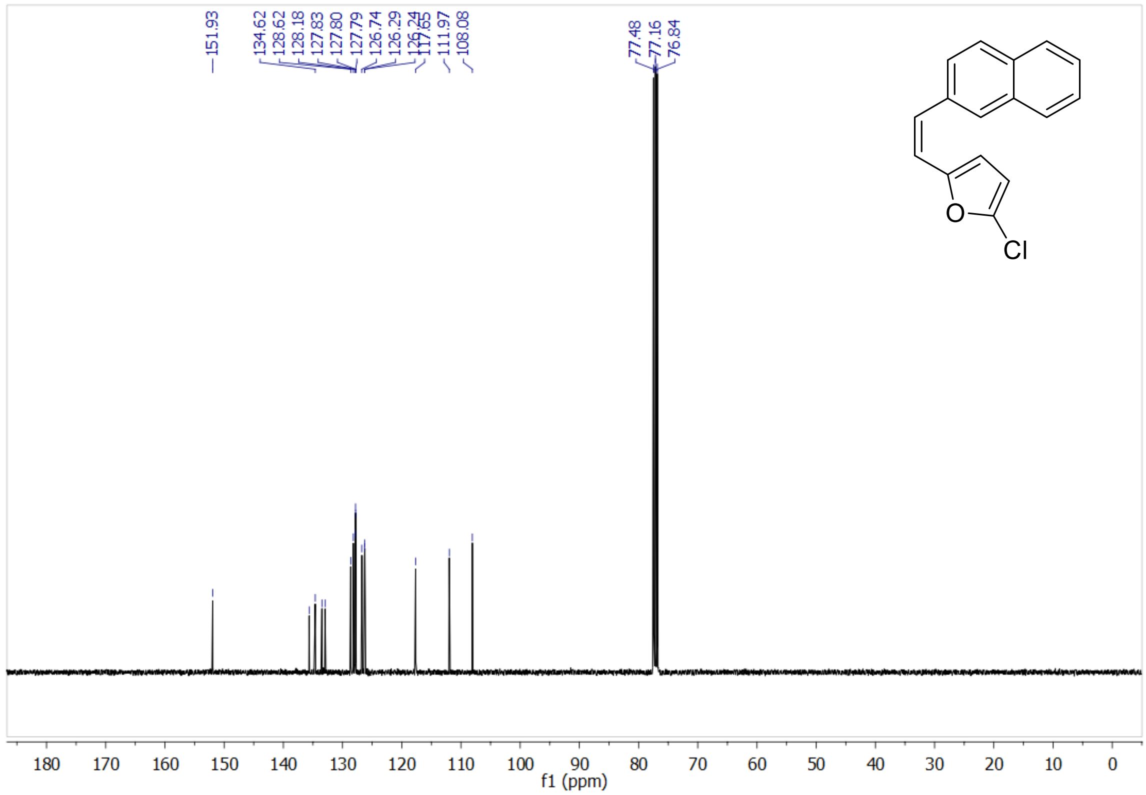

*¹H-NMR of (E/Z)-2-chloro-5-(2-(naphthalen-2-yl)vinyl)furan (400 MHz, $CDCl_3$) (**1d**)*

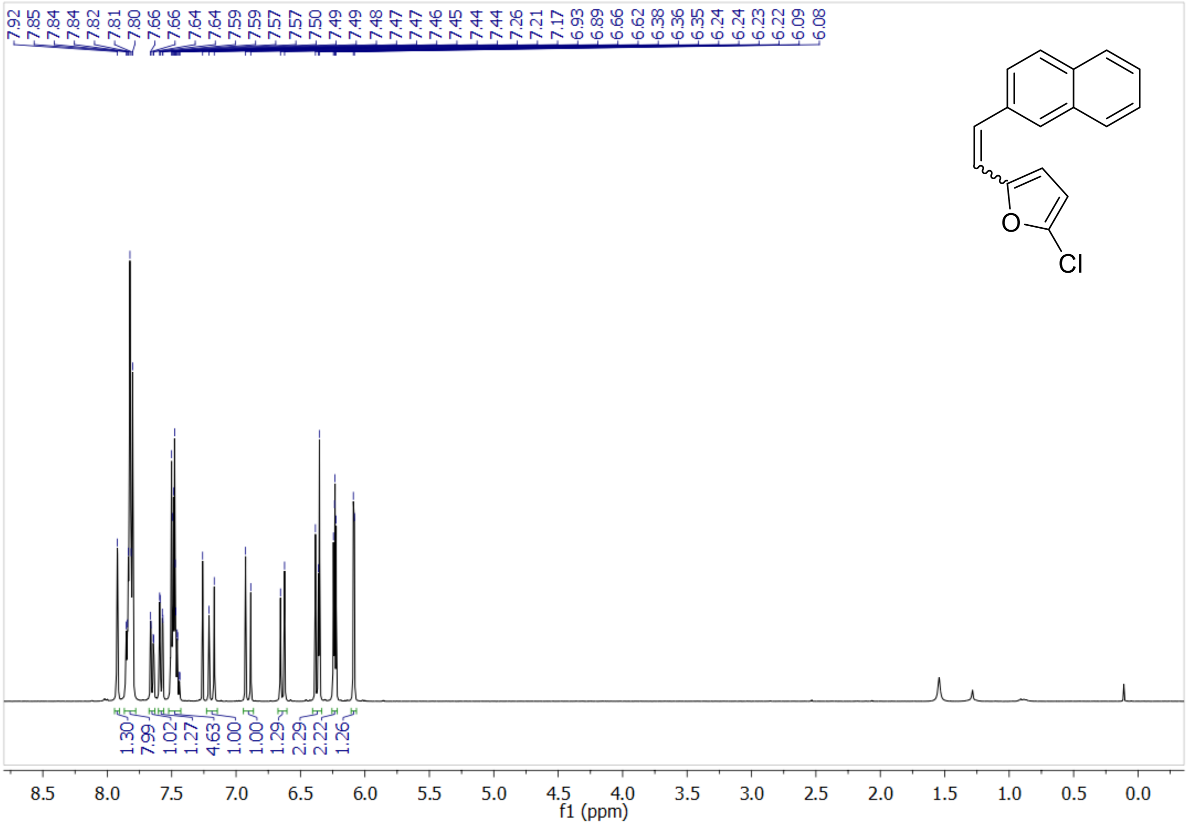

*¹³C-NMR of (E/Z)-2-chloro-5-(2-(naphthalen-2-yl)vinyl)furan (101 MHz, $CDCl_3$) (**1d**)*

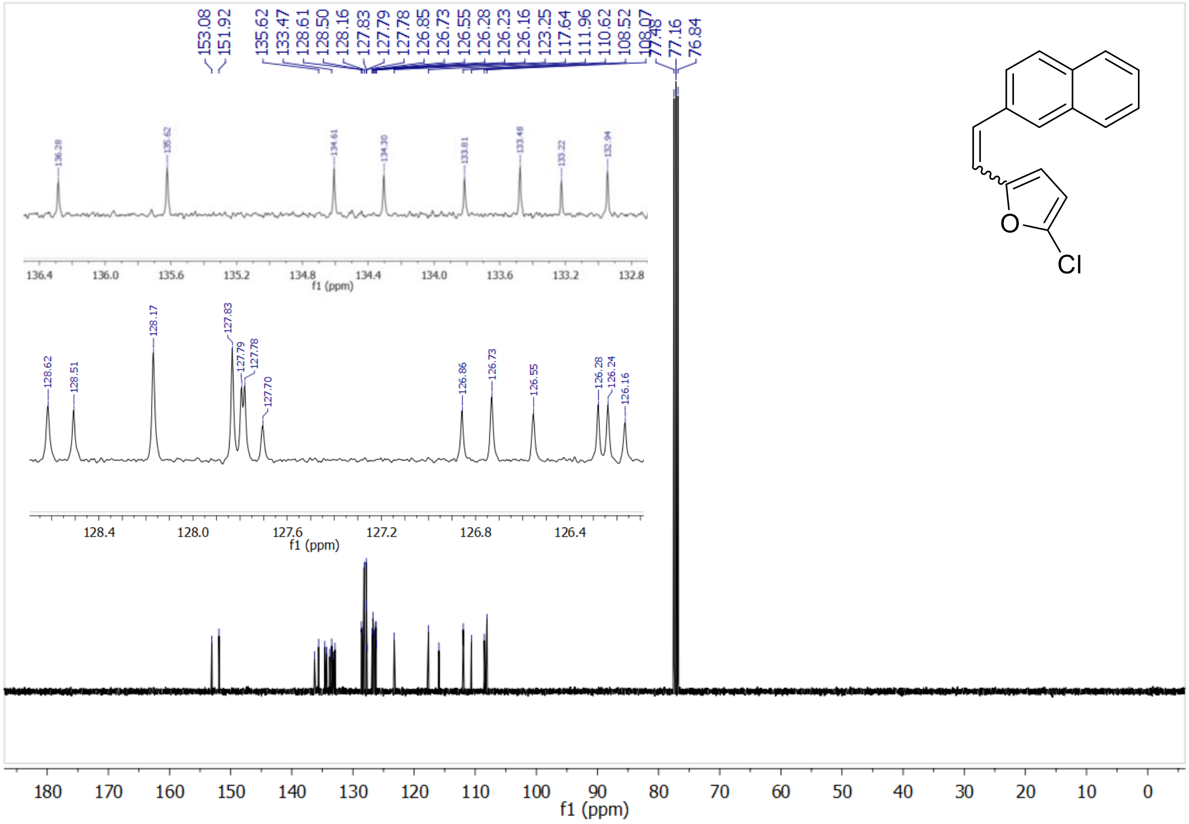

^{1}H-NMR of (E)-2-(2-(benzo[c]phenanthren-2-yl)vinyl)thiophene (400 MHz, CDCl$_3$) (**1e**)

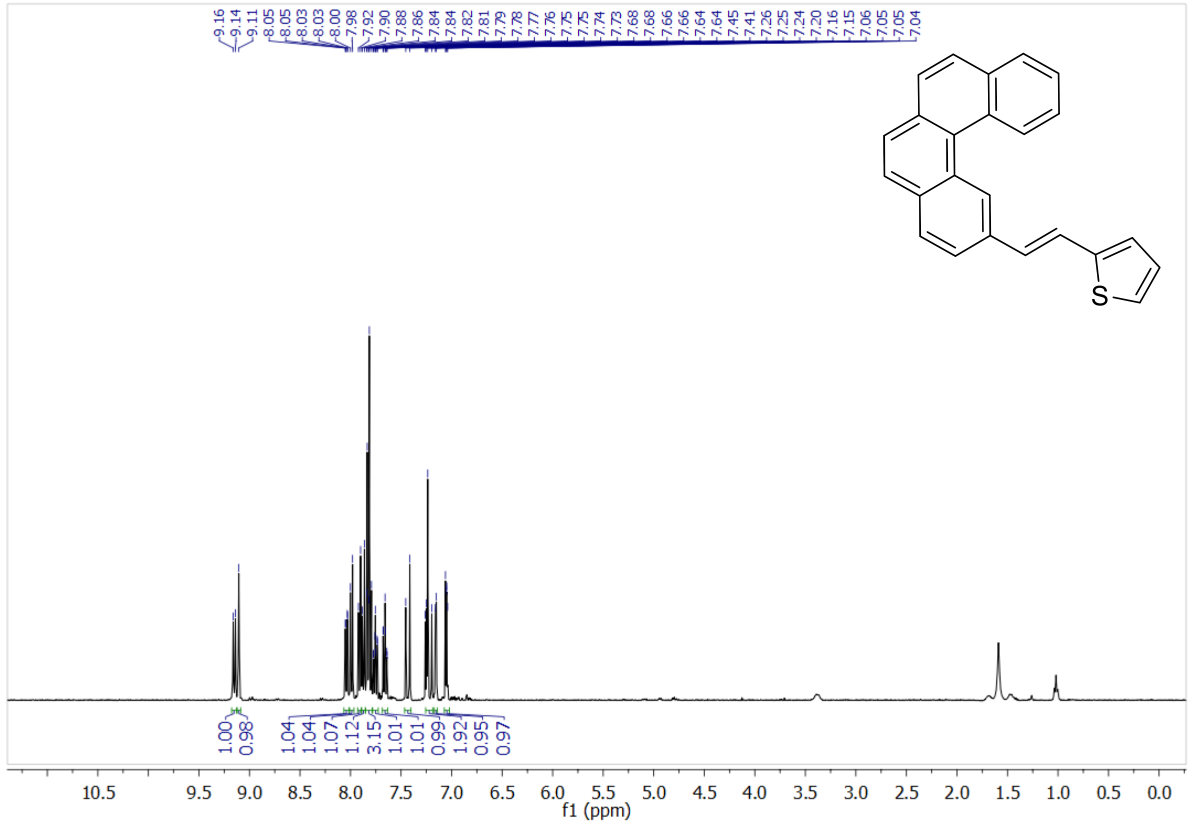

^{13}C-NMR of (E)-2-(2-(benzo[c]phenanthren-2-yl)vinyl)thiophene (101 MHz, CDCl$_3$) (**1e**)

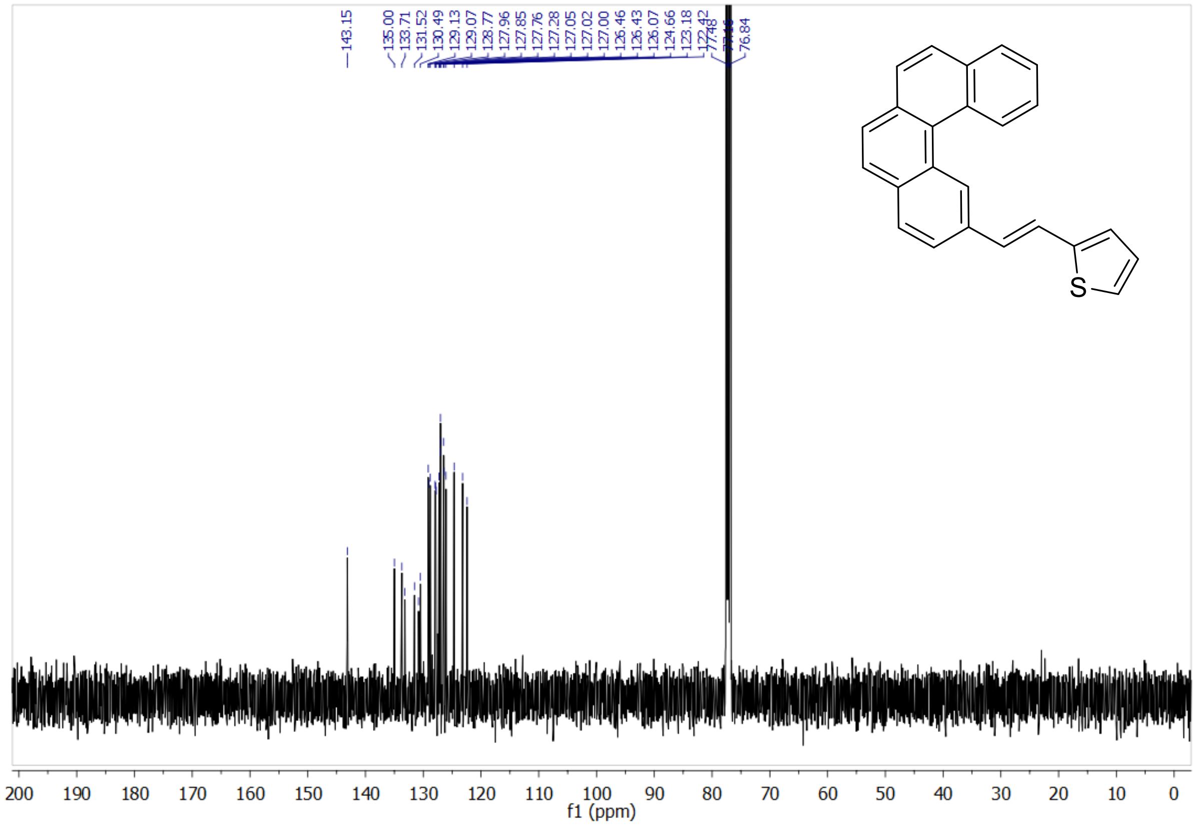

*^{1}H-NMR of (E/Z)-2-(2-(benzo[c]phenanthren-2-yl)vinyl)thiophene (400 MHz, CDCl$_3$) (**1e**)*

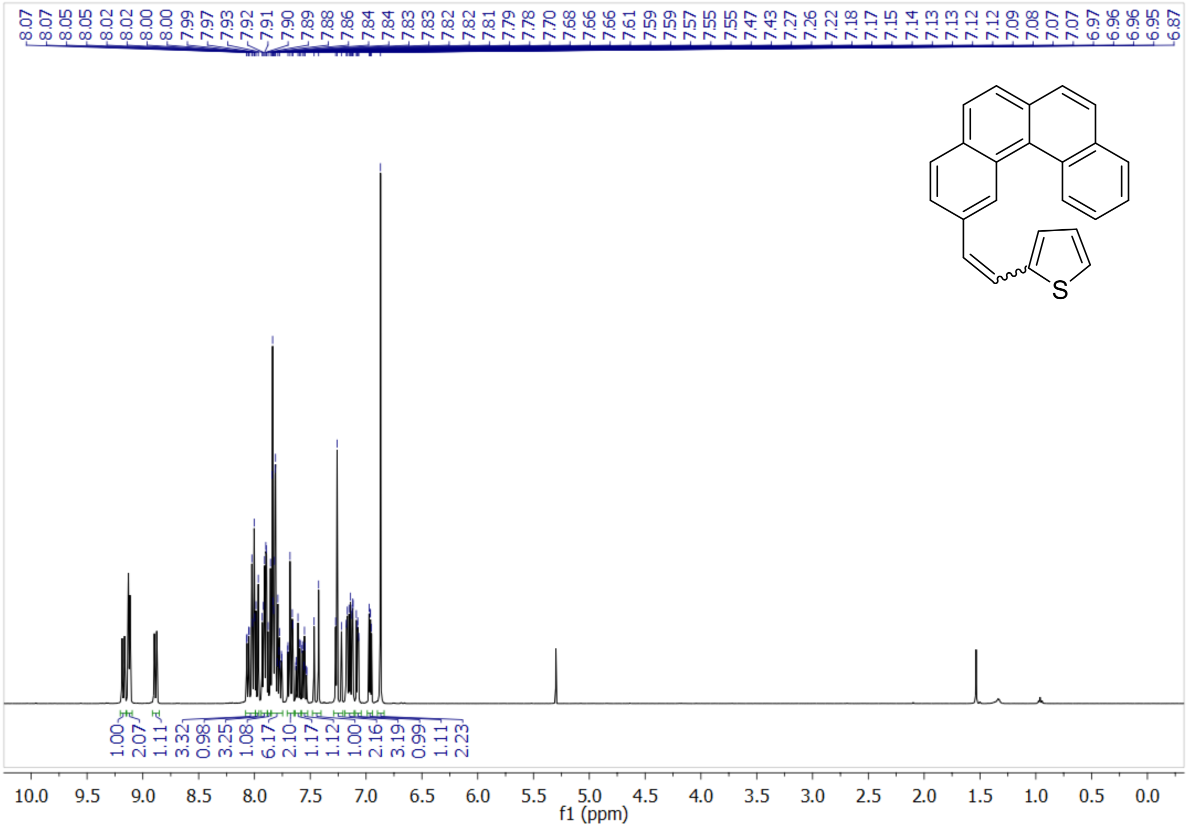

*^{13}C-NMR of (E/Z)-2-(2-(benzo[c]phenanthren-2-yl)vinyl)thiophene (101 MHz, CDCl$_3$) (**1e**)*

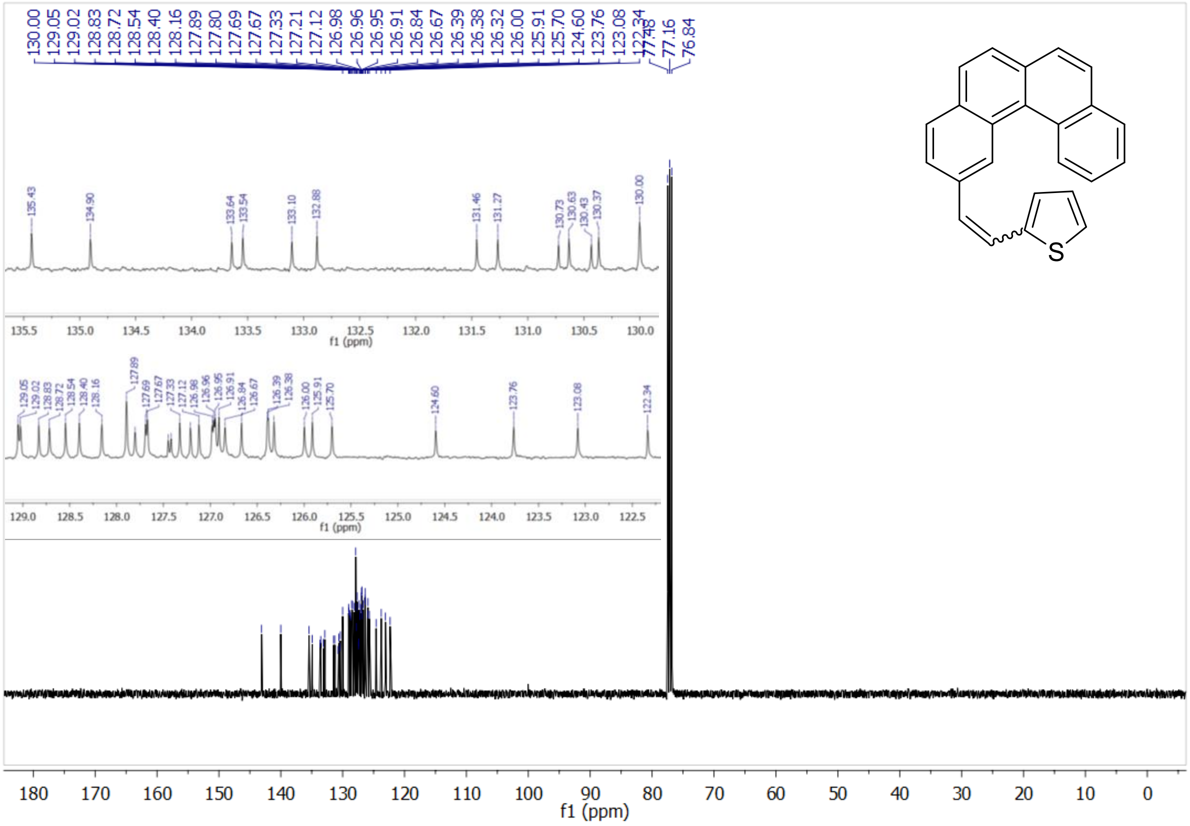

*^{1}H-NMR of (Z)-5-(2-(naphthalen-2-yl)vinyl)benzo[b]thiophene (400 MHz, CDCl$_3$) (**1f**)*

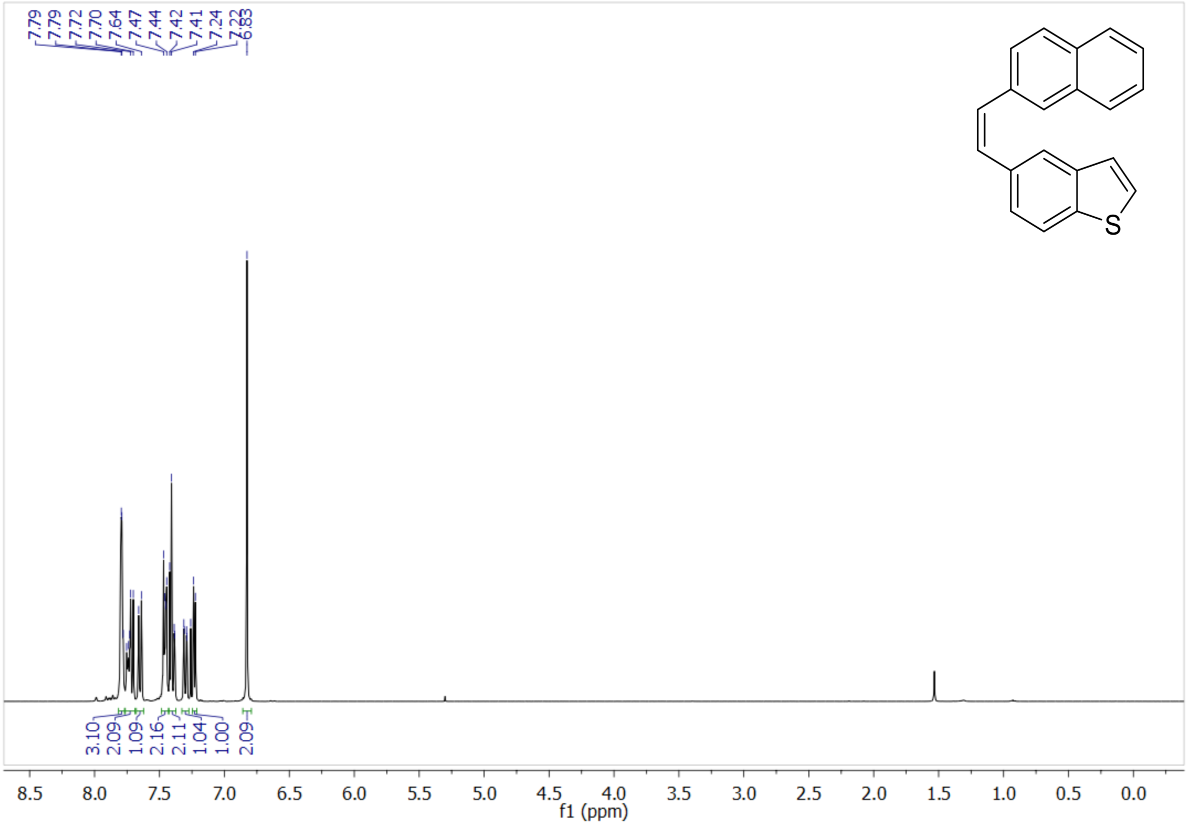

^{13}C-NMR of (Z)-5-(2-(naphthalen-2-yl)vinyl)benzo[b]thiophene (101 MHz, $CDCl_3$) (**1f**)

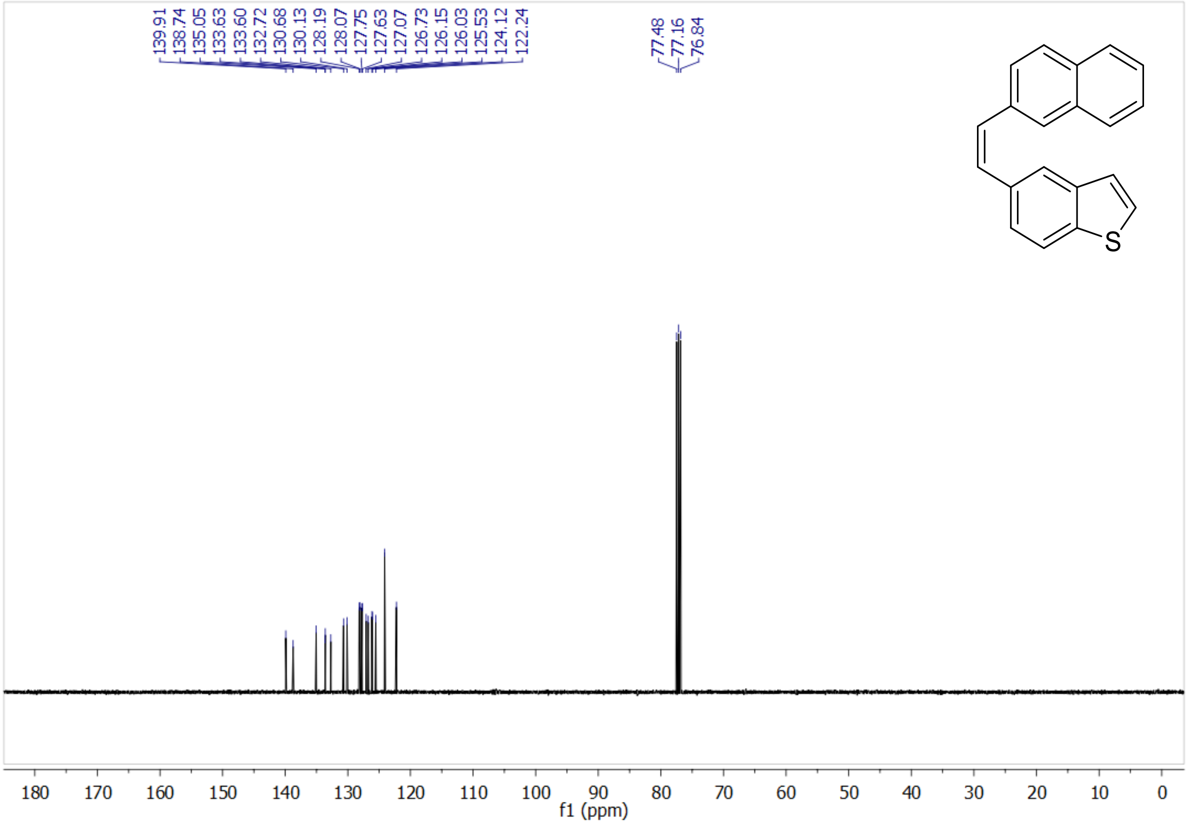

^{1}H-NMR of (E)-5-(2-(naphthalen-2-yl)vinyl)benzo[b]thiophene (400 MHz, THF-d8) (**1f**)

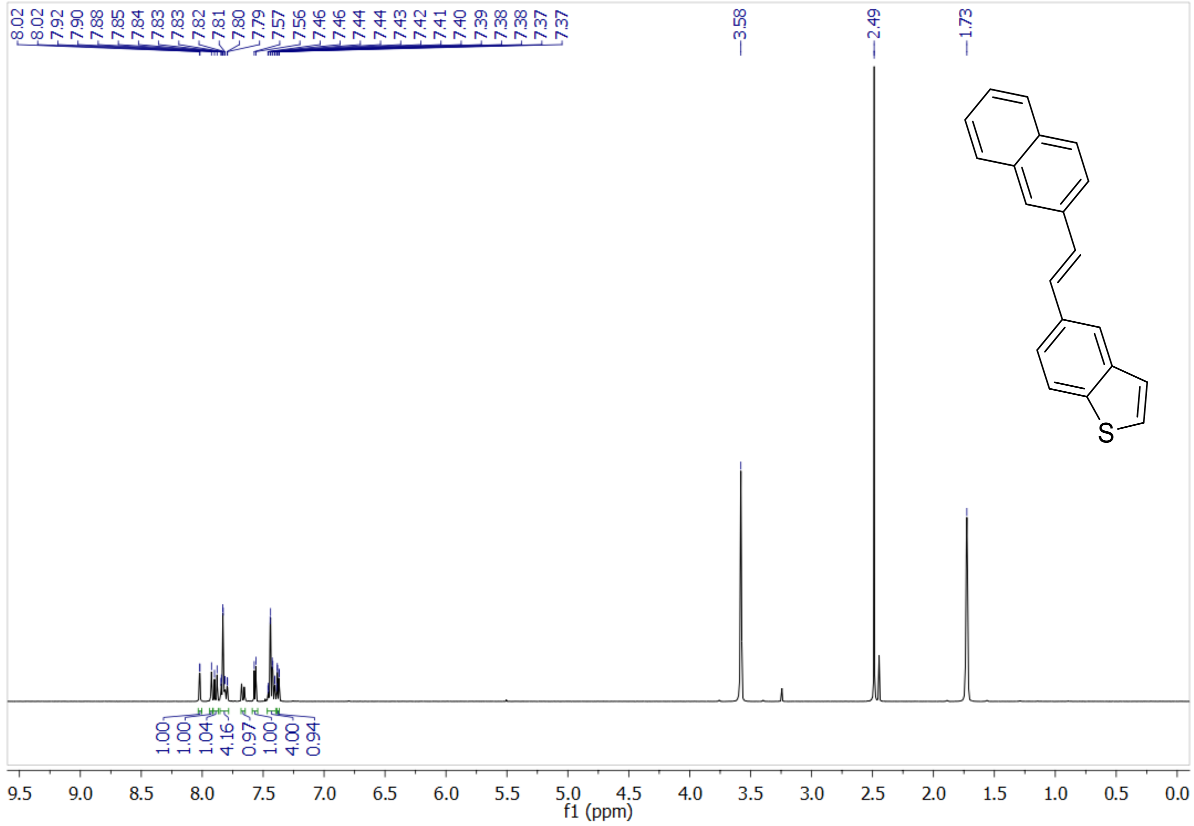

*^{1}H-NMR of 2,5-bis(2-(naphthalen-2-yl)vinyl)thiophene (400 MHz, THF-*d8*) (**1g**)*

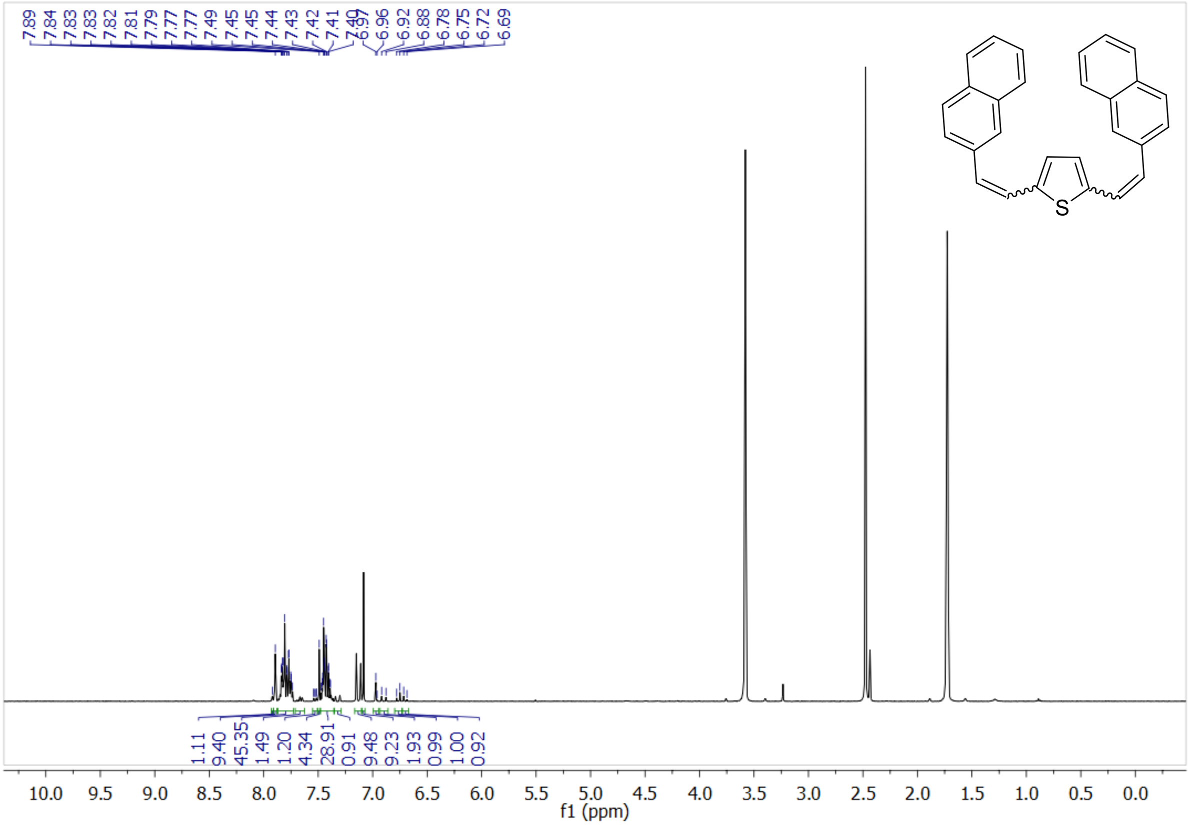

^{1}H-NMR of (E)-3-(2-(naphthalen-2-yl)vinyl)quinoline (400 MHz, $CDCl_3$) (**1h**)

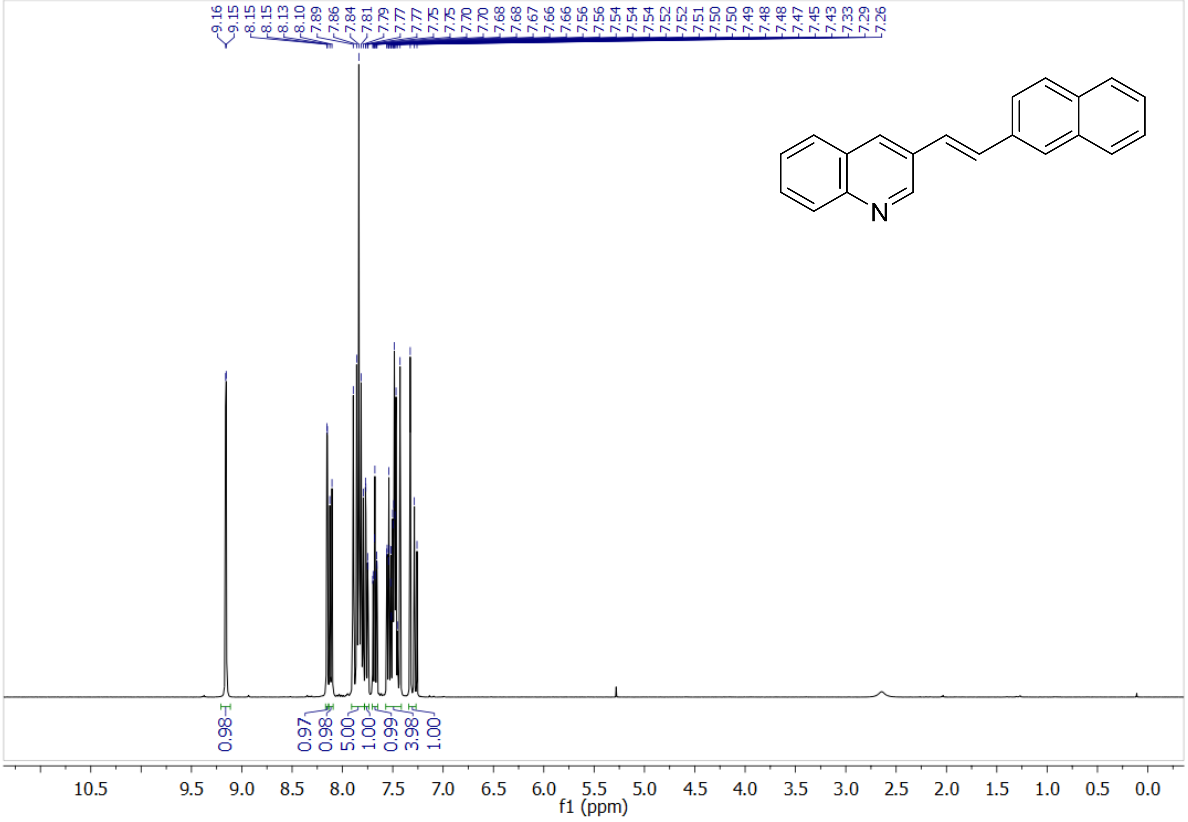

^{13}C-NMR of (E)-3-(2-(naphthalen-2-yl)vinyl)quinoline (101 MHz, $CDCl_3$) (**1h**)

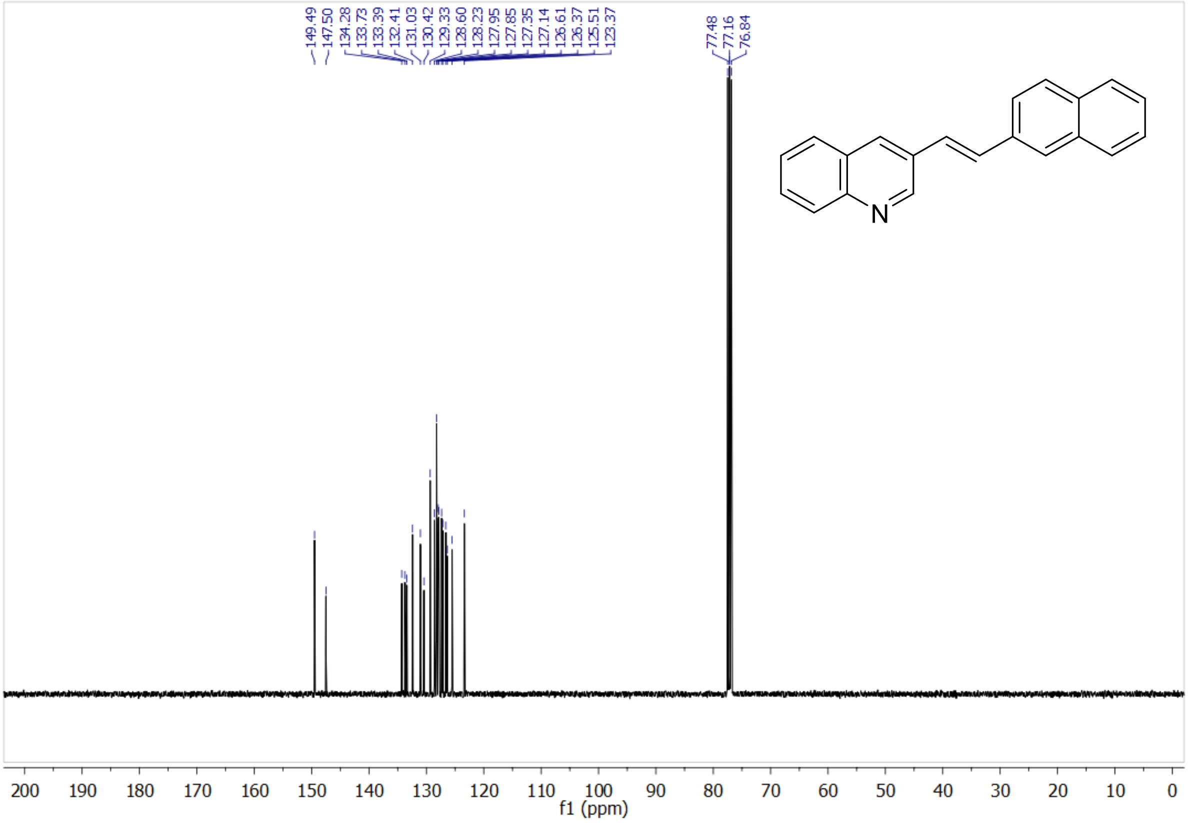

*^{1}H-NMR of (Z)-3-(2-(naphthalen-2-yl)vinyl)quinoline (400 MHz, $CDCl_3$) (**1h**)*

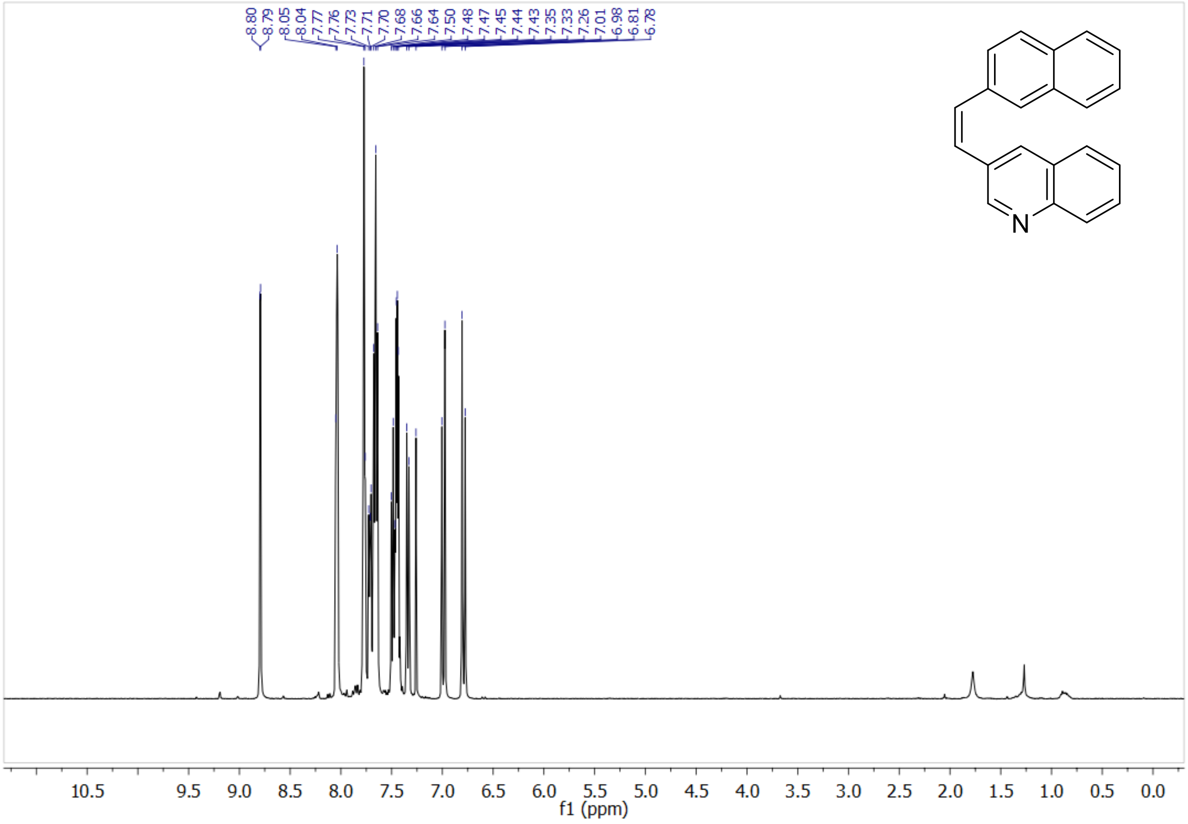

*^{13}C-NMR of (Z)-3-(2-(naphthalen-2-yl)vinyl)quinoline (101 MHz, $CDCl_3$) (**1h**)*

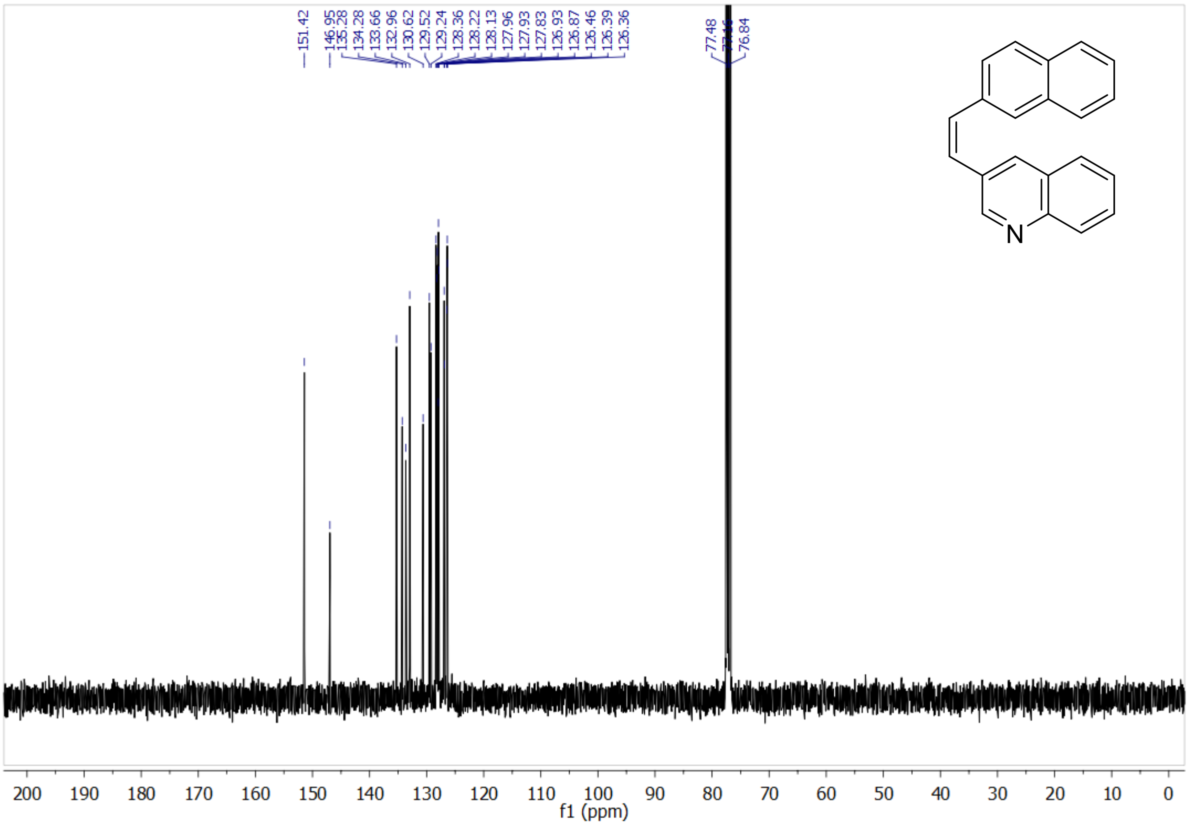

*^{1}H-NMR of (E/Z)-4-(2-(naphthalen-2-yl)vinyl)thiazole (400 MHz, CDCl$_3$) (**1i**)*

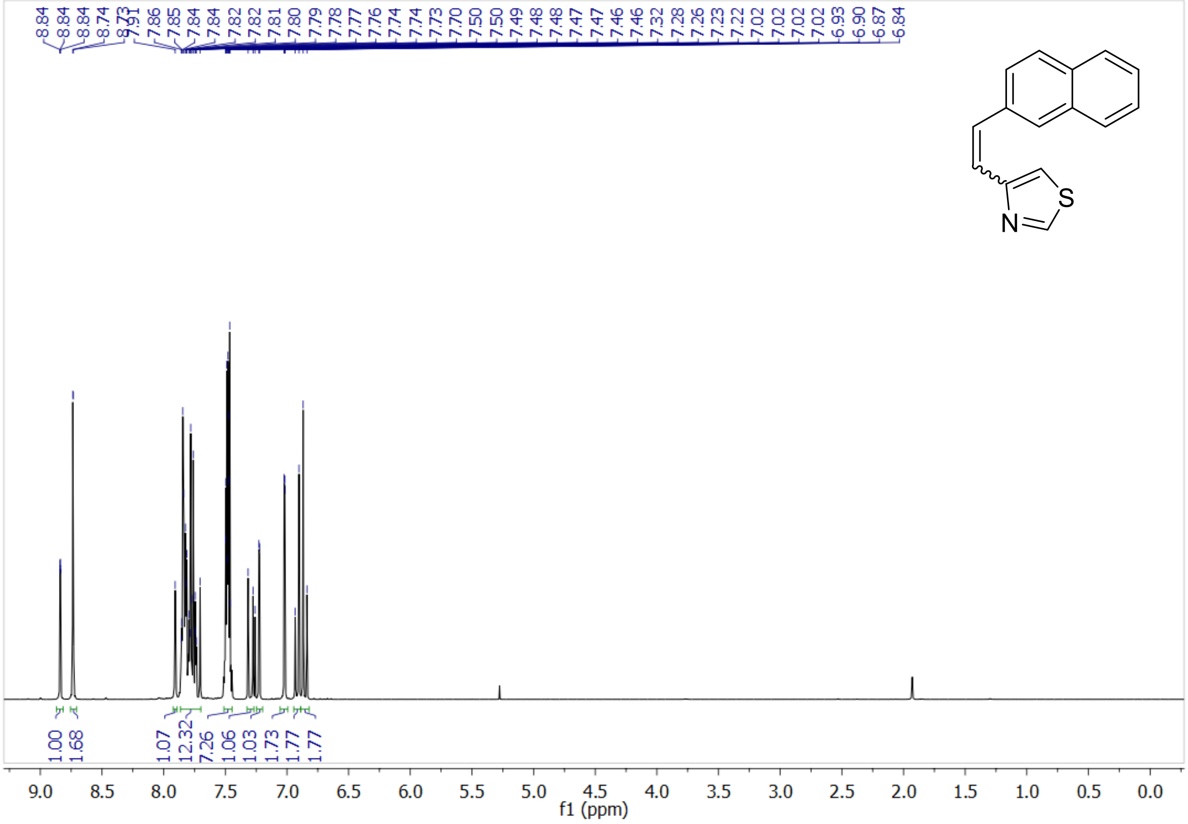

*^{13}C-NMR of (E/Z)-4-(2-(naphthalen-2-yl)vinyl)thiazole (101 MHz, CDCl$_3$) (**1i**)*

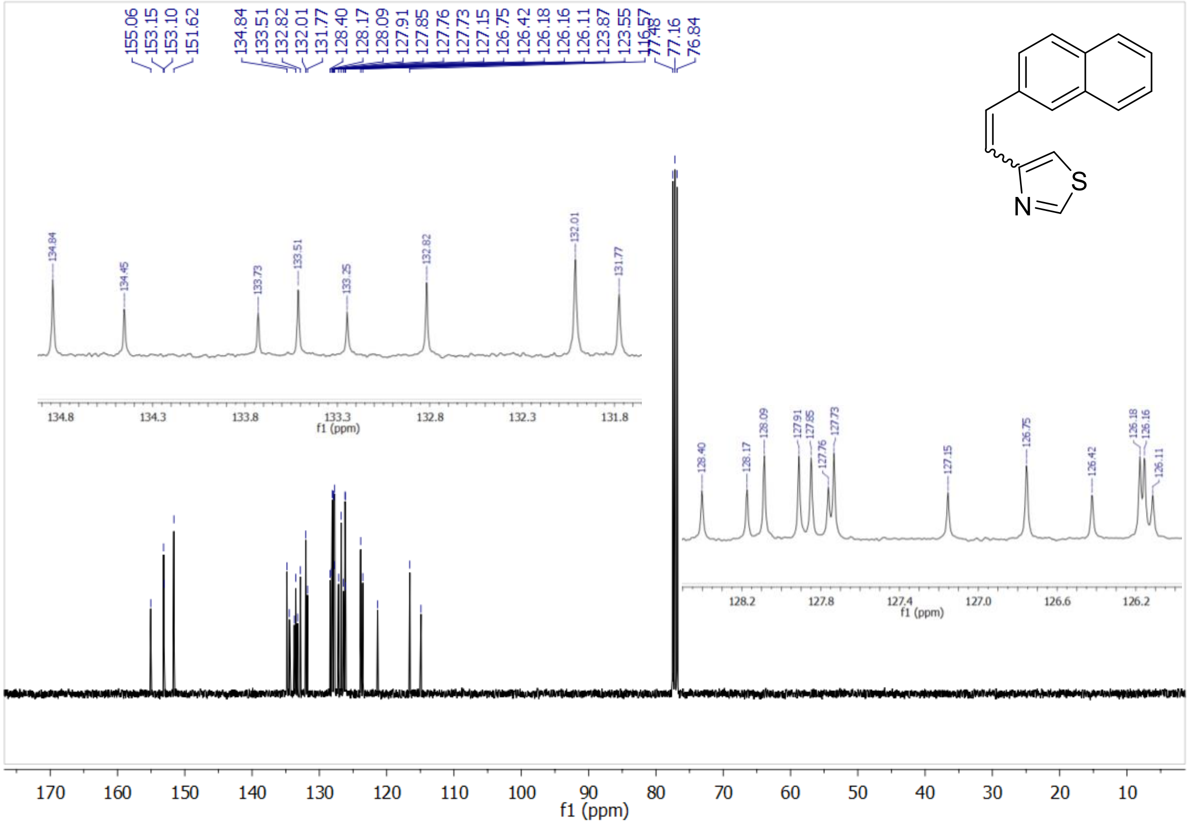

^{1}H-NMR of (E)-5-(2-(naphthalen-2-yl)vinyl)benzo[d]oxazole (400 MHz, $CDCl_3$) (**1k**)

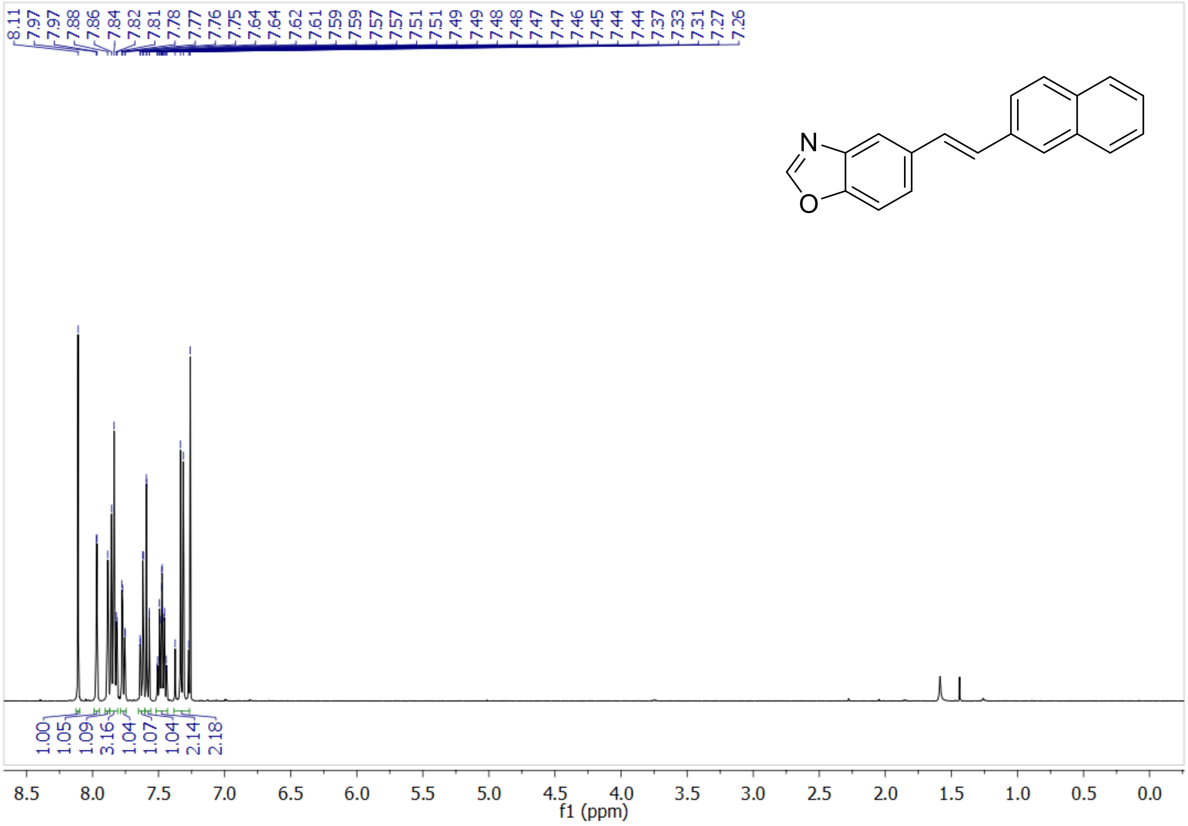

^{13}C-NMR of (E)-5-(2-(naphthalen-2-yl)vinyl)benzo[d]oxazole (101 MHz, $CDCl_3$) (**1k**)

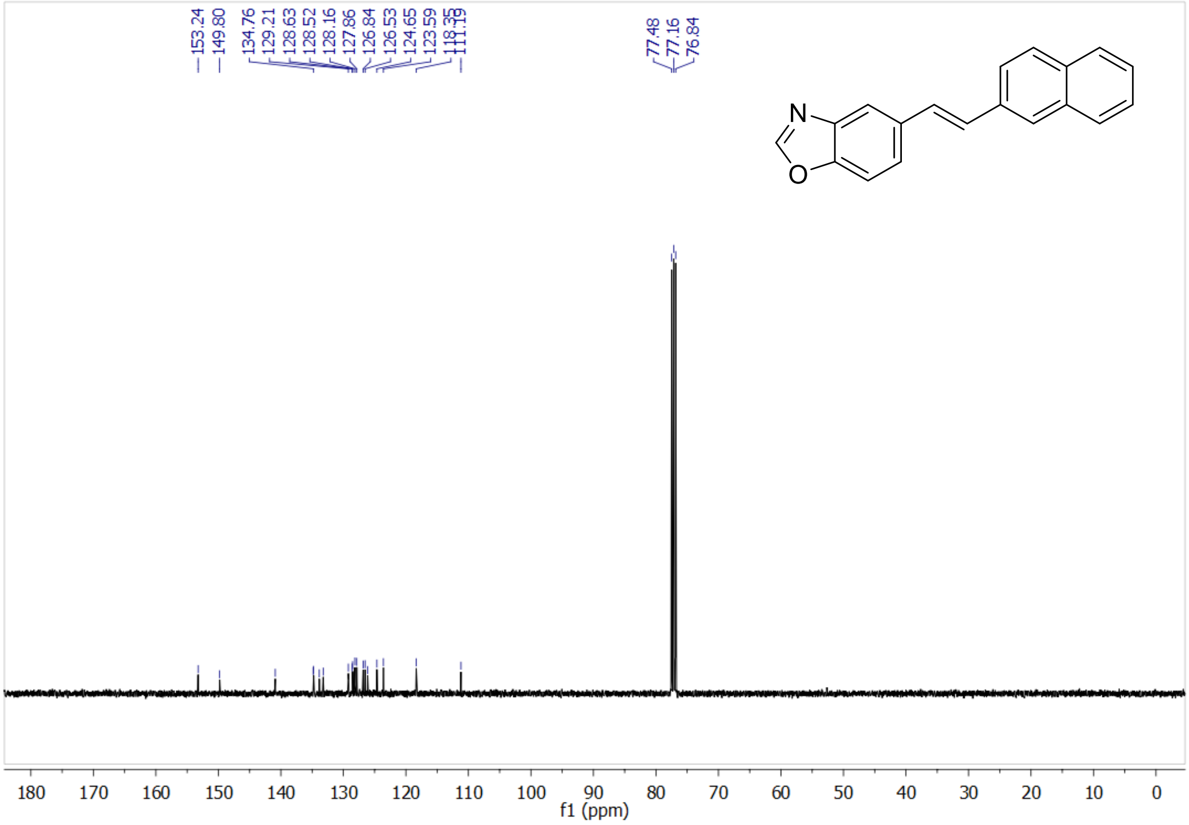

*^{1}H-NMR of (Z)-5-(2-(naphthalen-2-yl)vinyl)benzo[d]oxazole (400 MHz, $CDCl_3$) (**1k**)*

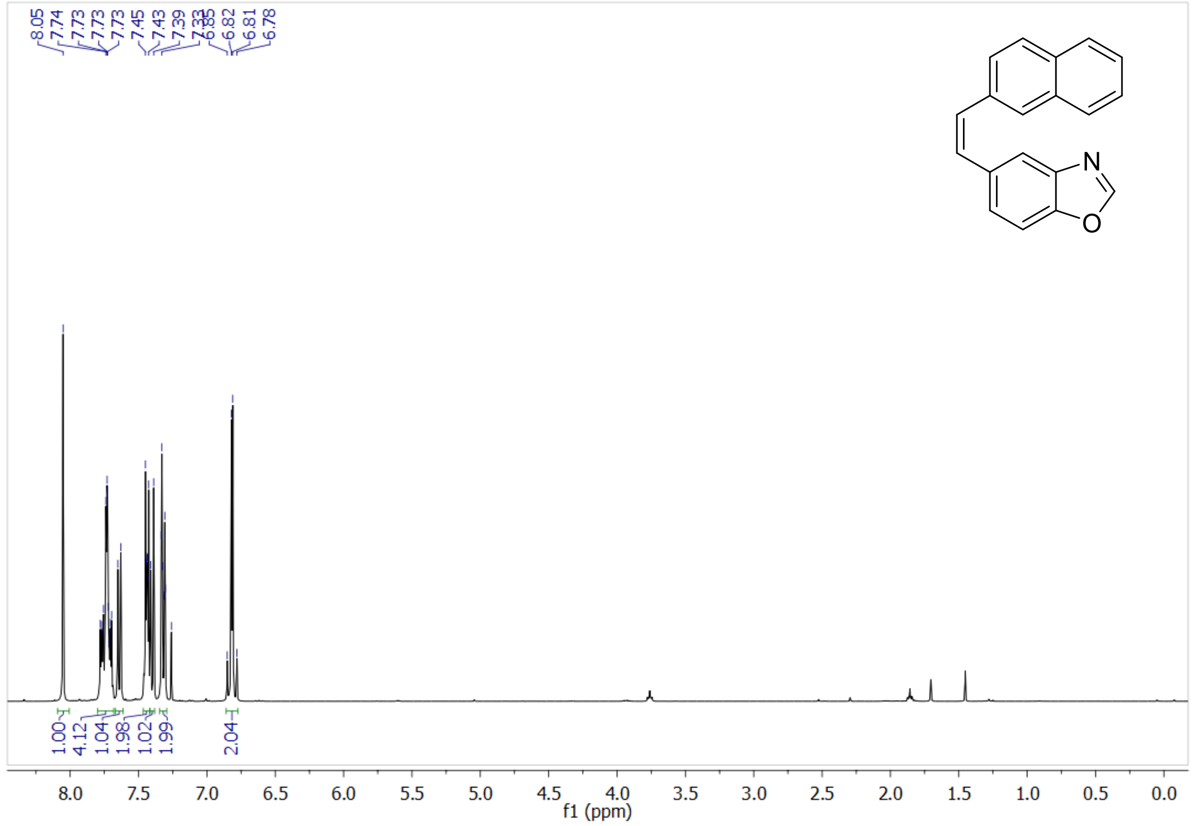

*^{13}C-NMR of (Z)-5-(2-(naphthalen-2-yl)vinyl)benzo[d]oxazole (101 MHz, $CDCl_3$) (**1k**)*

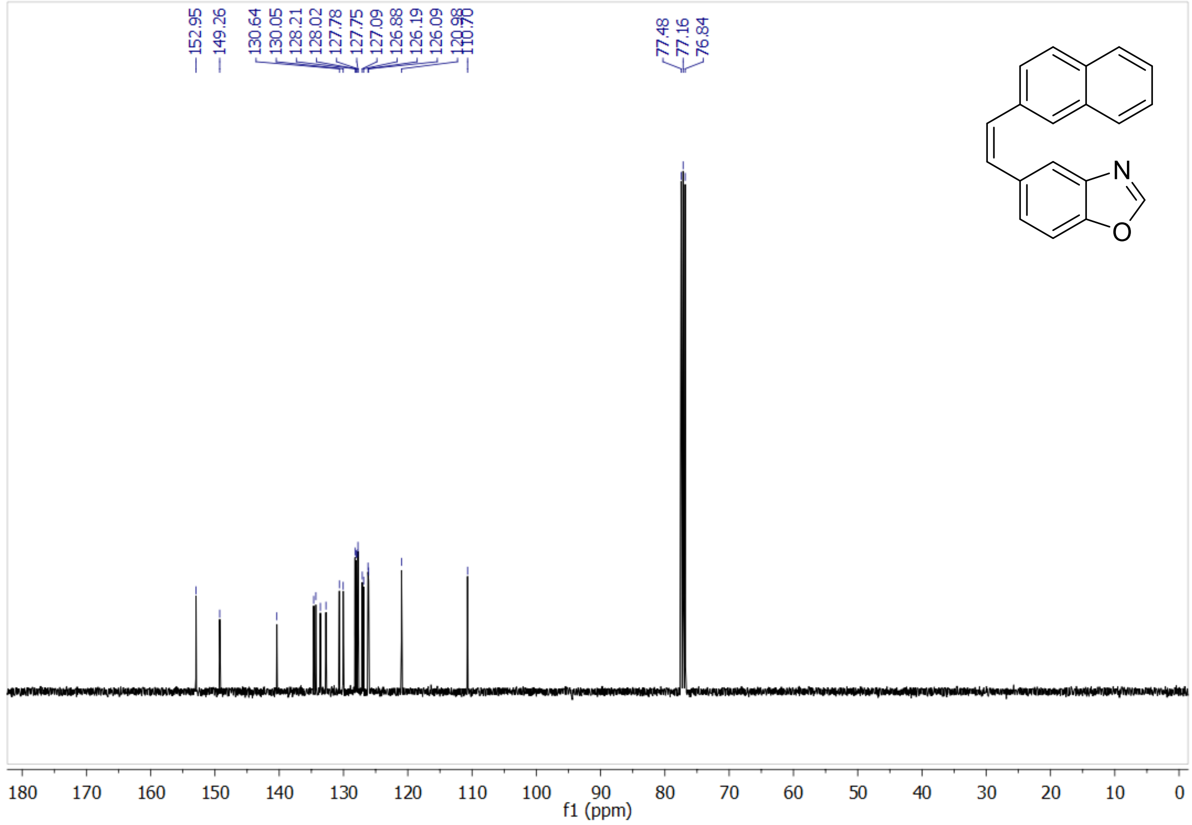

*^{1}H-NMR of (E)-3-chloro-5-(2-(naphthalen-2-yl)vinyl)pyridine (400 MHz, CDCl₃) (**1m**)*

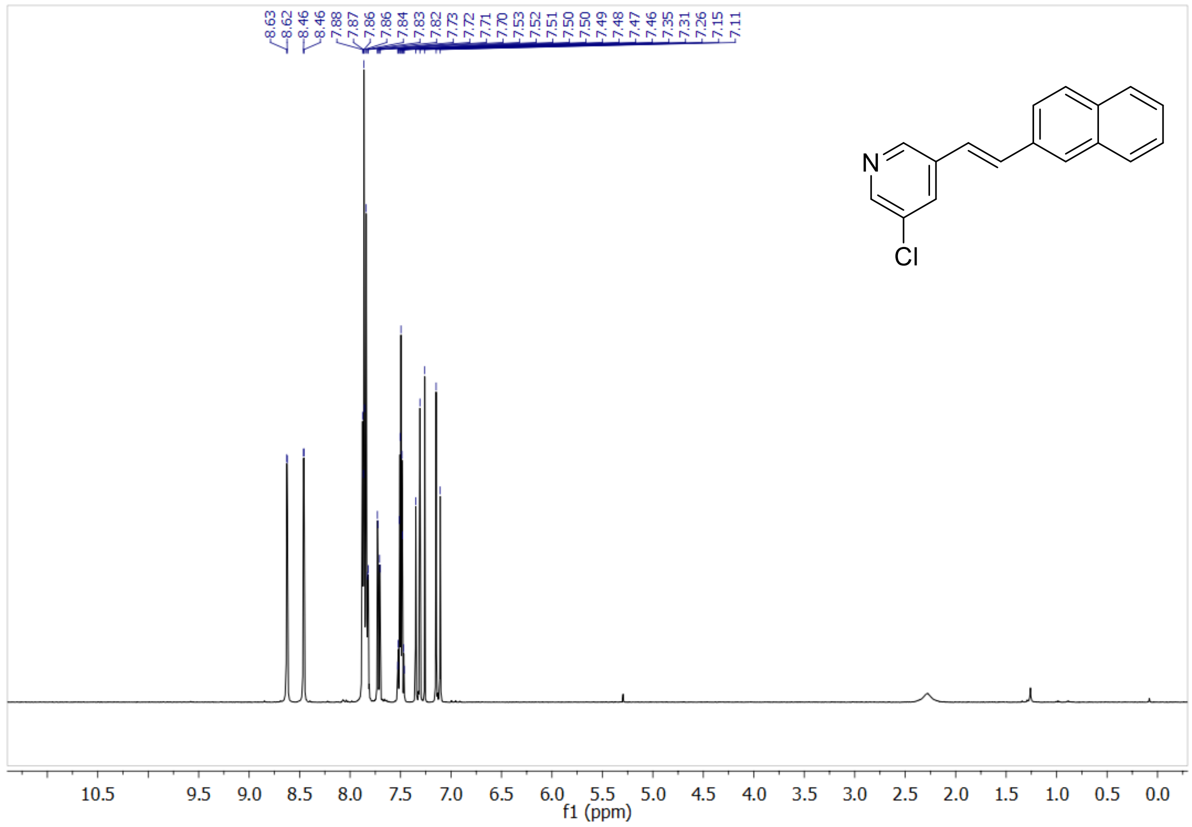

*^{13}C-NMR of (E)-3-chloro-5-(2-(naphthalen-2-yl)vinyl)pyridine (101 MHz, CDCl₃) (**1m**)*

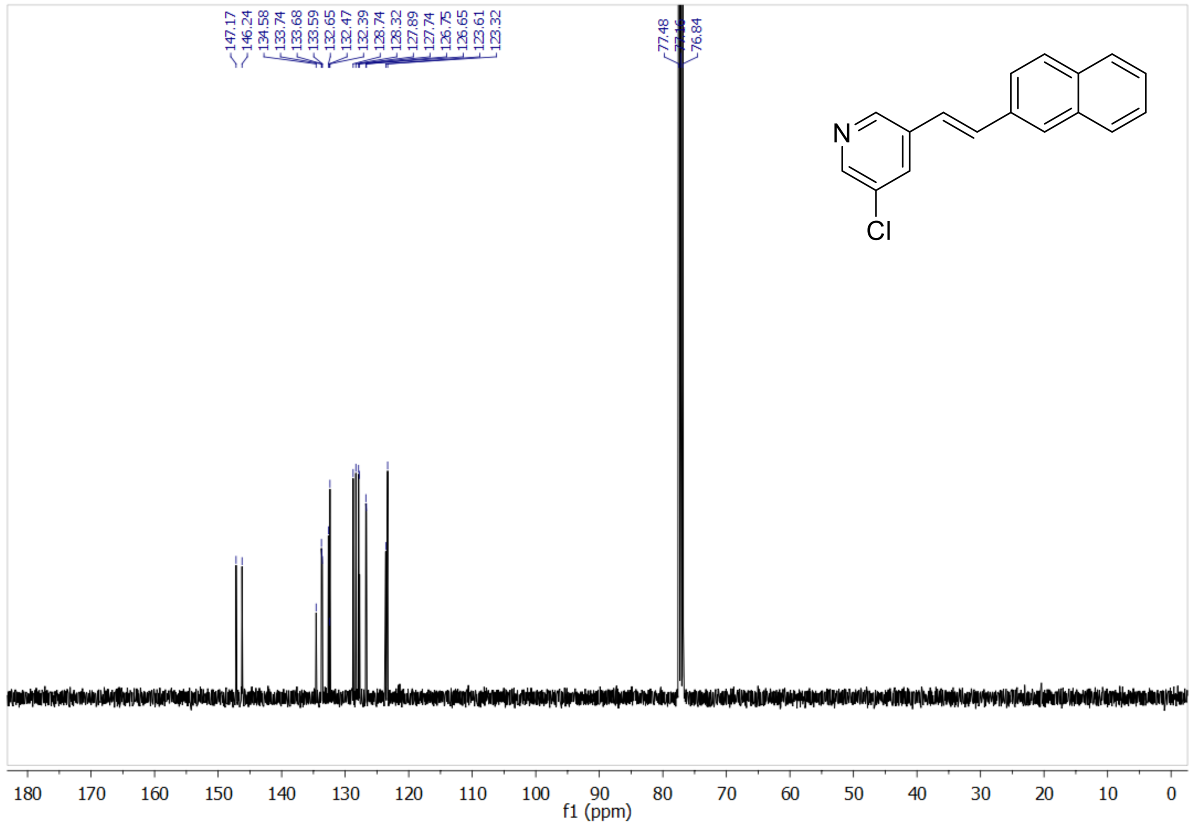

^{1}H-NMR of (Z)-3-chloro-5-(2-(naphthalen-2-yl)vinyl)pyridine (400 MHz, $CDCl_3$) (**1m**)

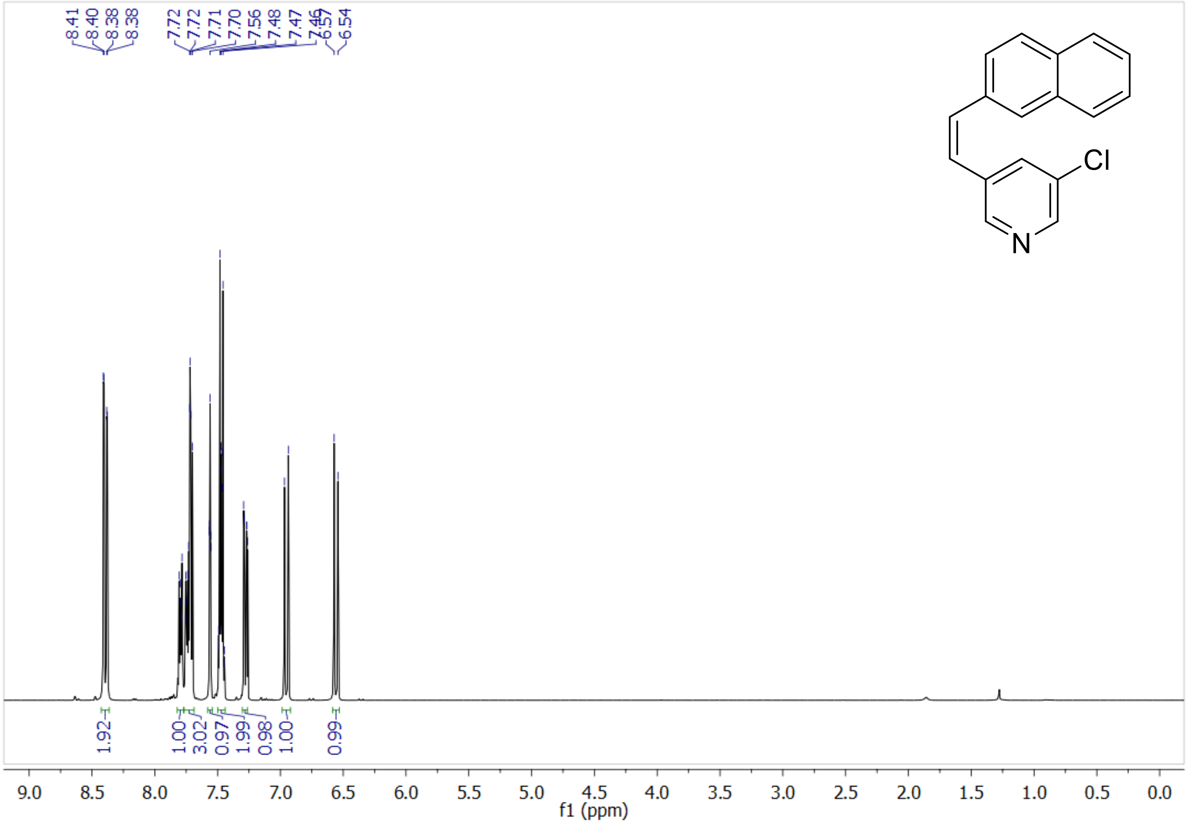

^{13}C-NMR of (Z)-3-chloro-5-(2-(naphthalen-2-yl)vinyl)pyridine (101 MHz, $CDCl_3$) (**1m**)

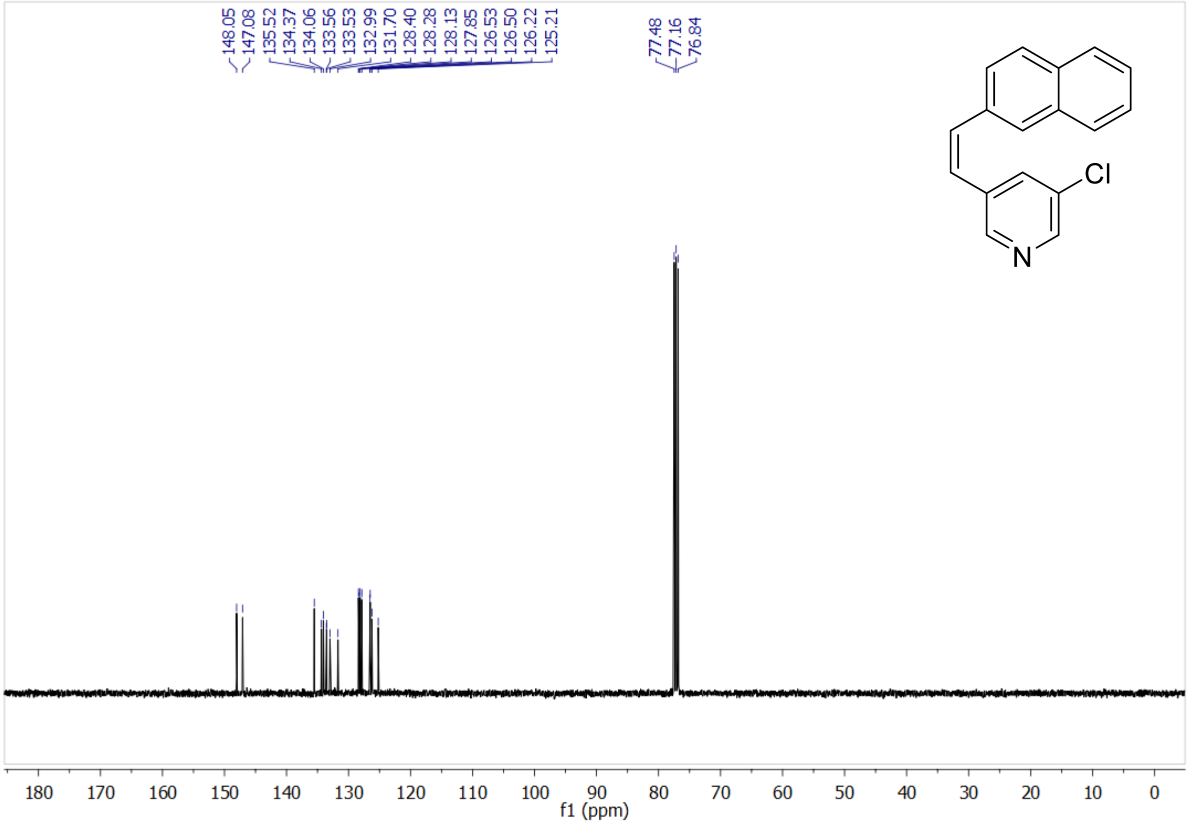

^{1}H-NMR of (E)-5-(2-(naphthalen-2-yl)vinyl)benzo[c][1,2,5]thiadiazole (400 MHz, $CDCl_3$) (**1n**)

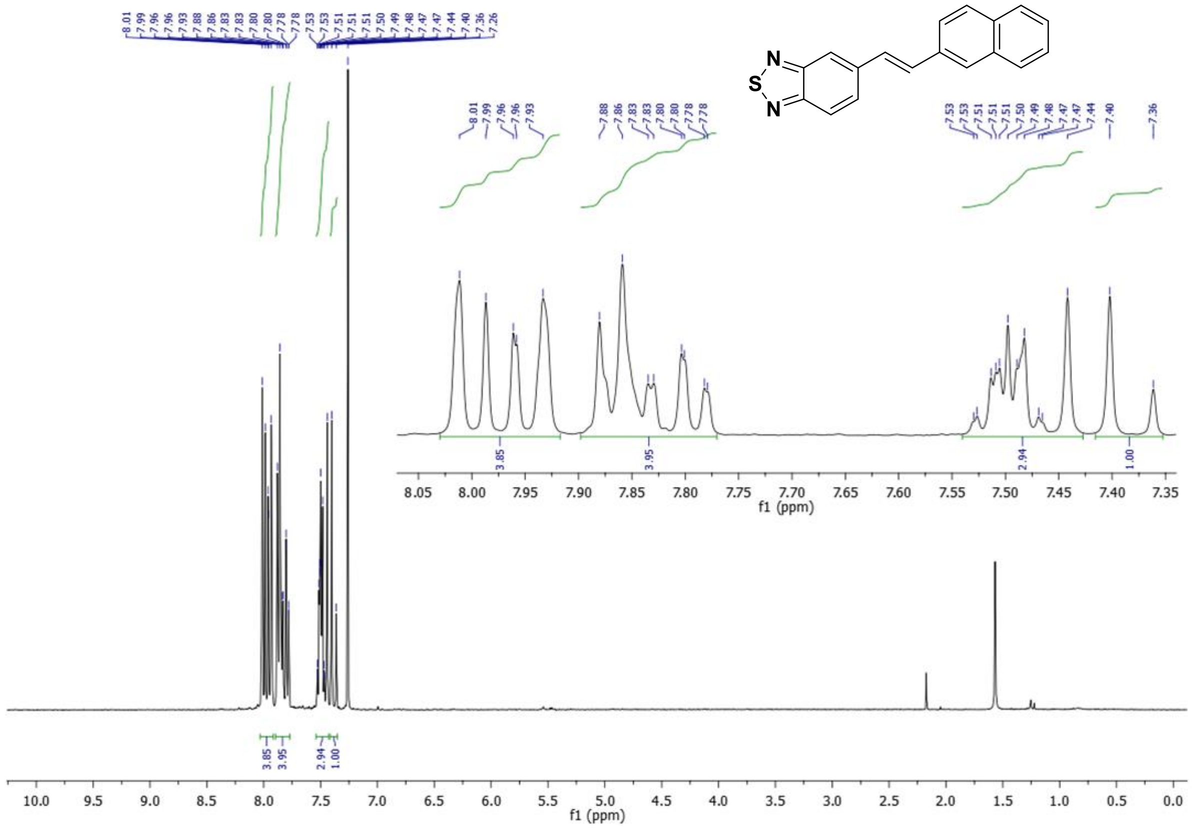

^{13}C-NMR of (E)-5-(2-(naphthalen-2-yl)vinyl)benzo[c][1,2,5]thiadiazole (101 MHz, $CDCl_3$) (**1n**)

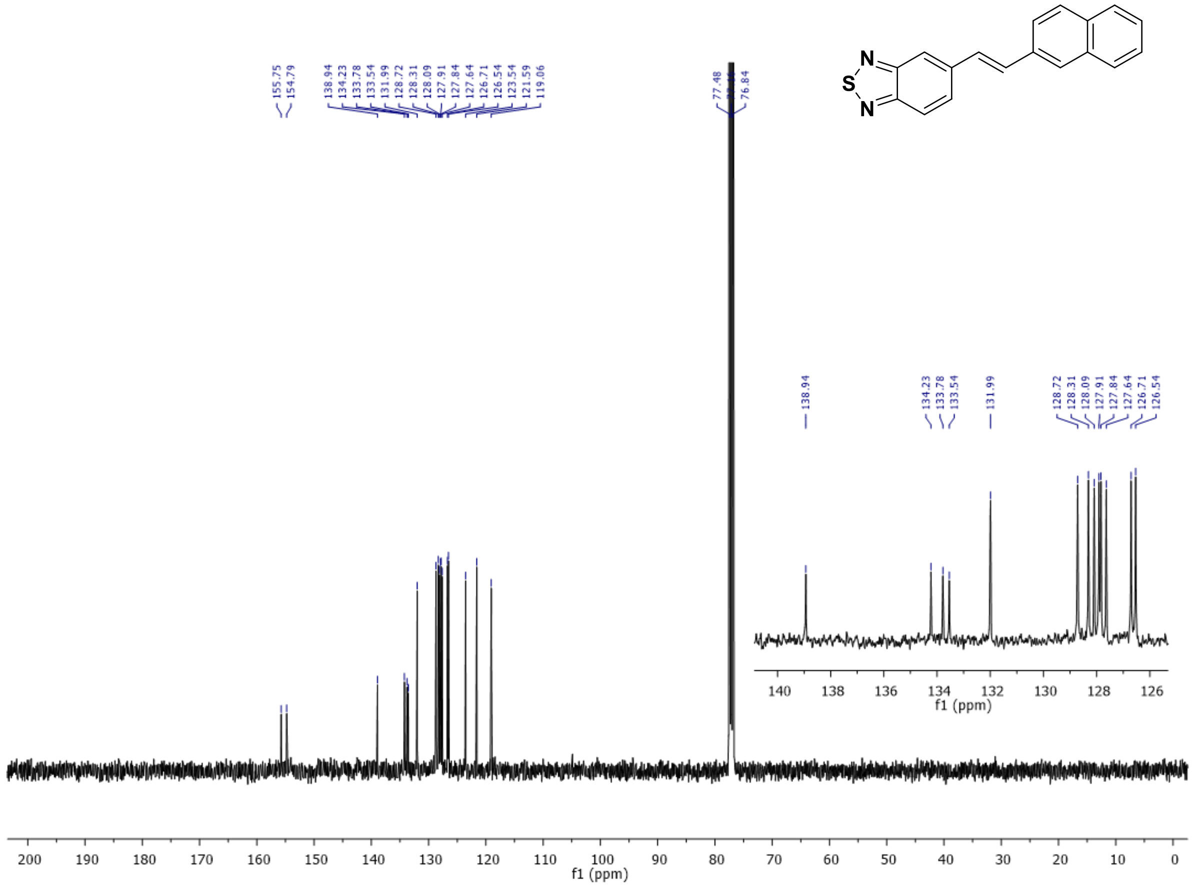

^{1}H-NMR of (E/Z)-5-(2-(naphthalen-2-yl)vinyl)benzo[c][1,2,5]thiadiazole (400 MHz, $CDCl_3$)
*(**1n**)*

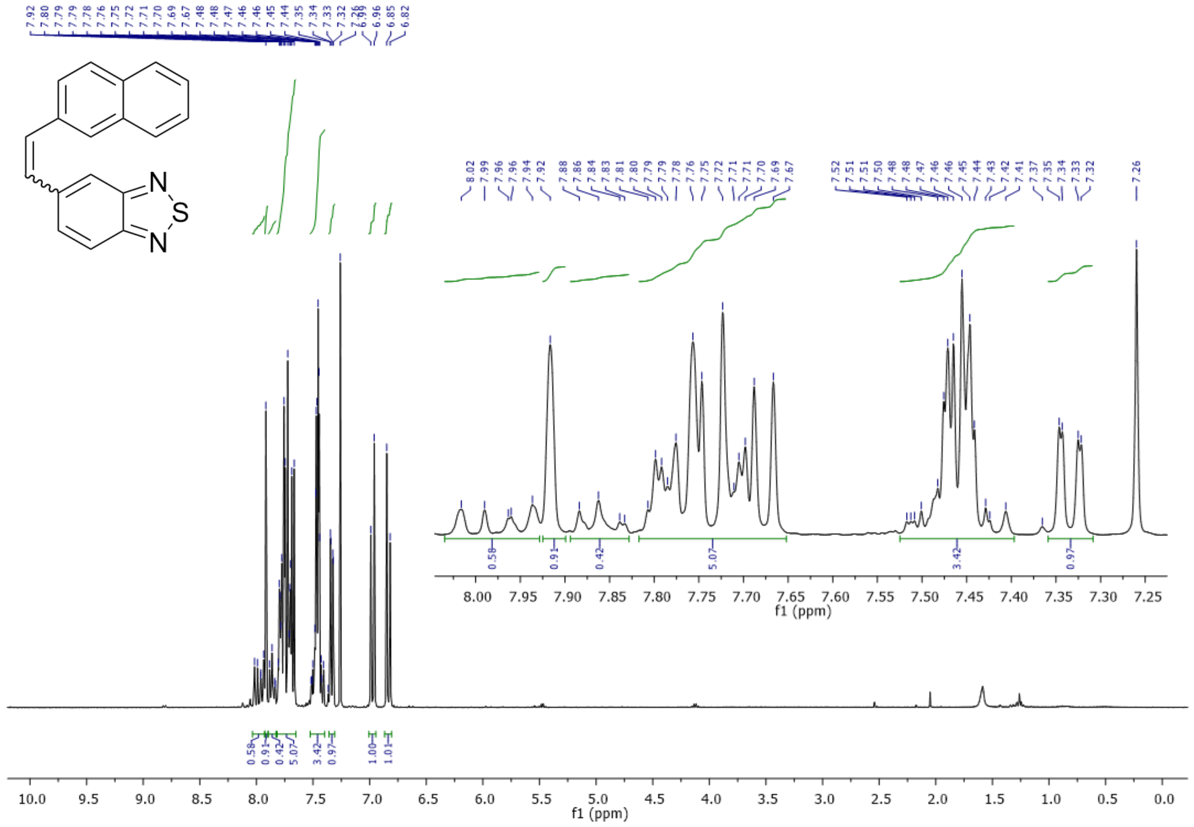

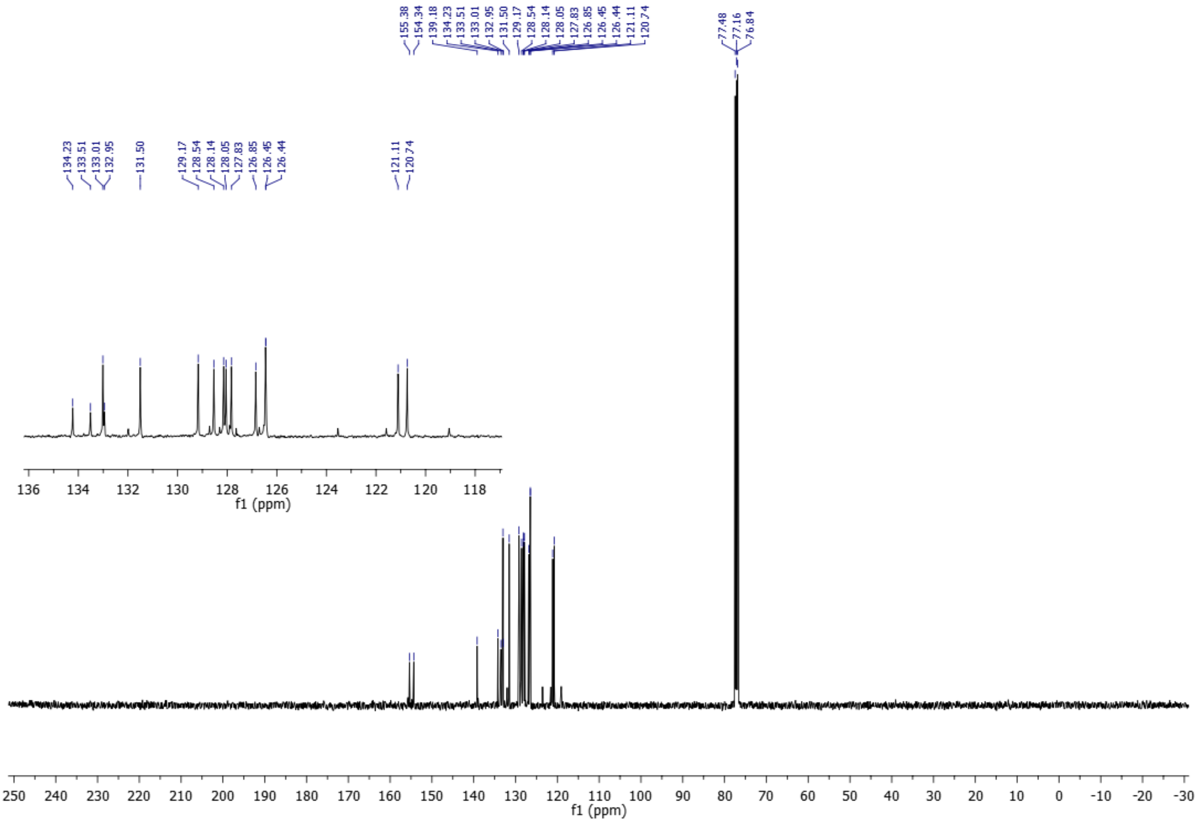

*^{13}C-NMR of (*E/Z*)-5-(2-(naphthalen-2-yl)vinyl)benzo[*c*][1,2,5]thiadiazole (101 MHz, $CDCl_3$) (**1n**)*

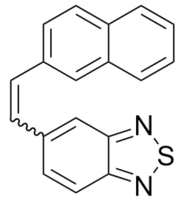

^{1}H-NMR of (Z)-2-chloro-3-(2-(naphthalen-2-yl)vinyl)pyridine (400 MHz, $CDCl_3$) (**1o**)

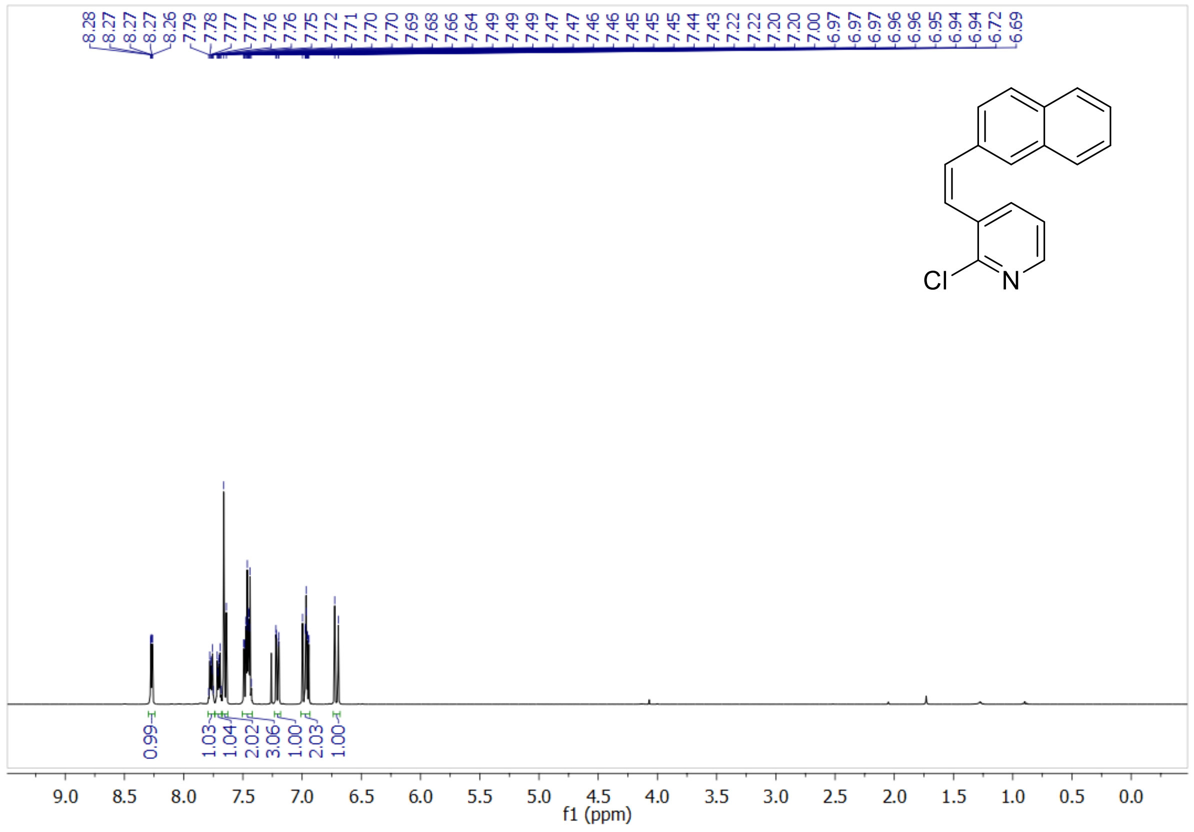

^{13}C-NMR of (Z)-2-chloro-3-(2-(naphthalen-2-yl)vinyl)pyridine (101 MHz, $CDCl_3$) (**1o**)

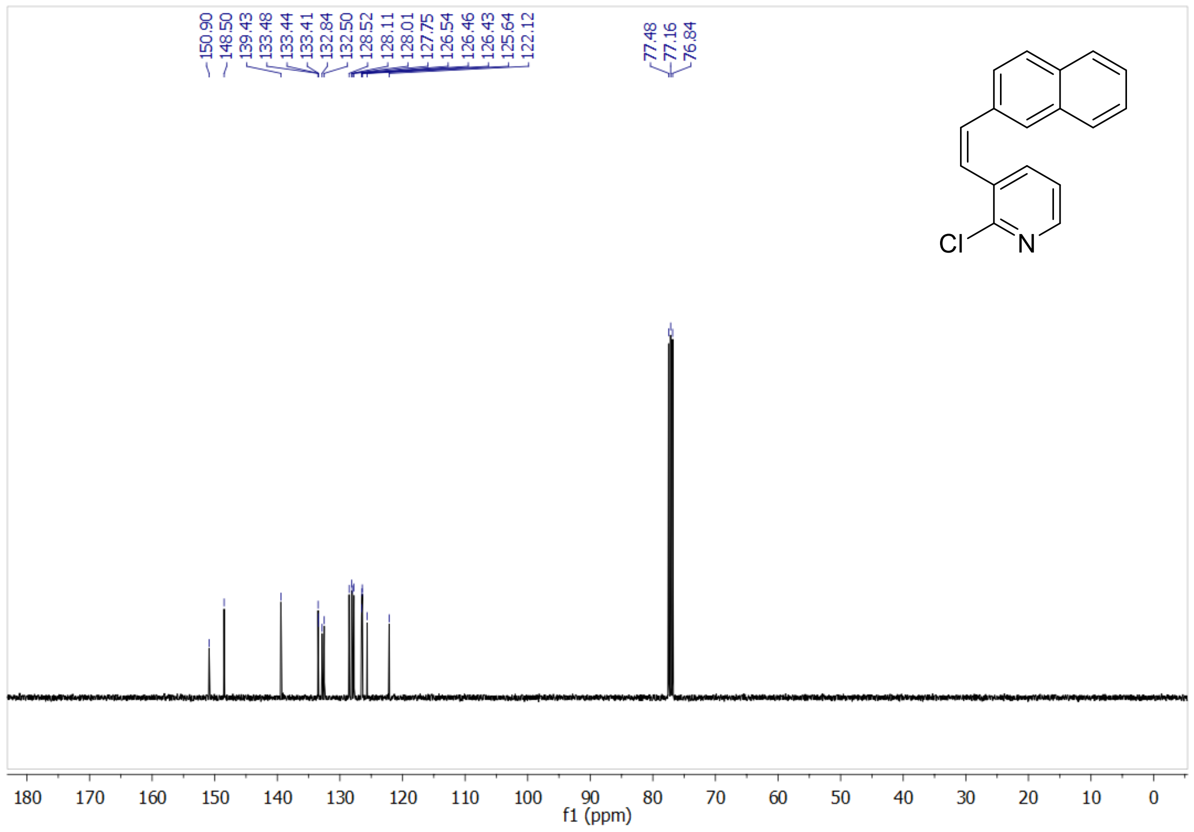

^{1}H-NMR of (E)-2-chloro-3-(2-(naphthalen-2-yl)vinyl)pyridine (400 MHz, $CDCl_3$) (**1o**)

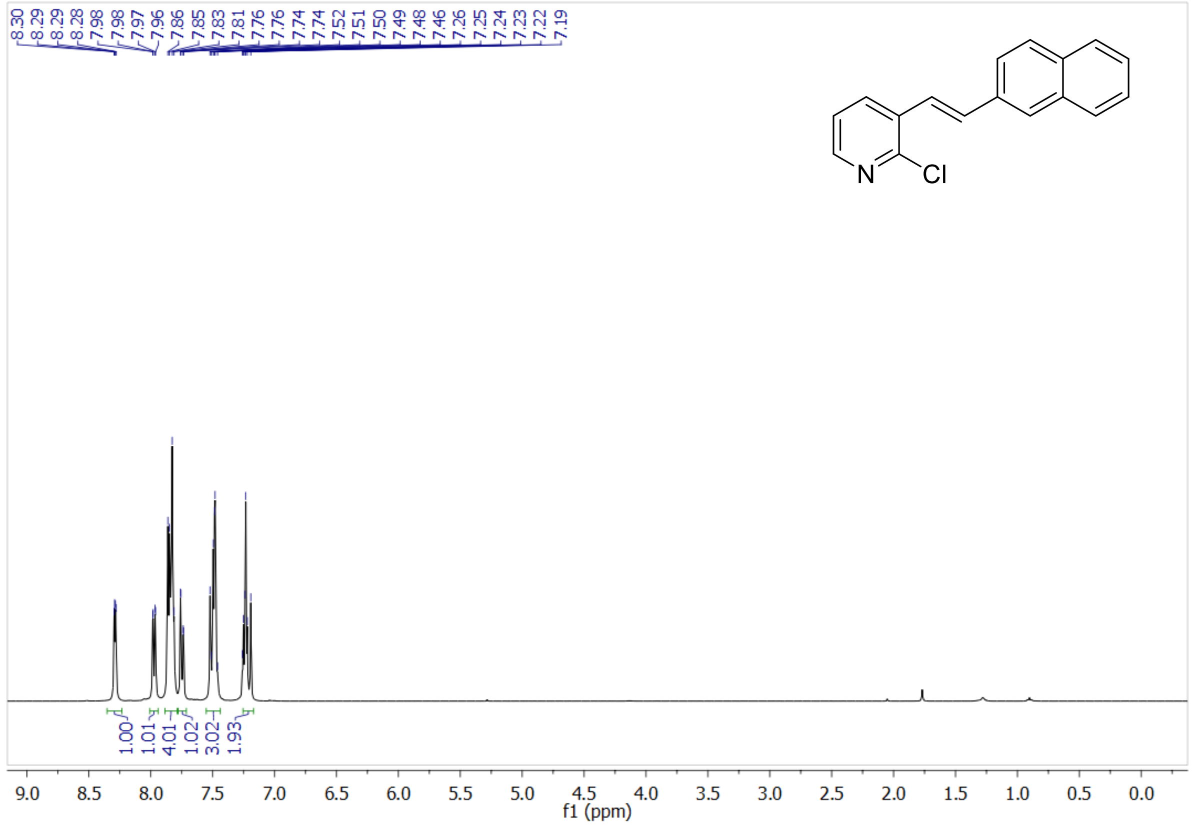

^{13}C-NMR of (E)-2-chloro-3-(2-(naphthalen-2-yl)vinyl)pyridine (101 MHz, $CDCl_3$) (**1o**)

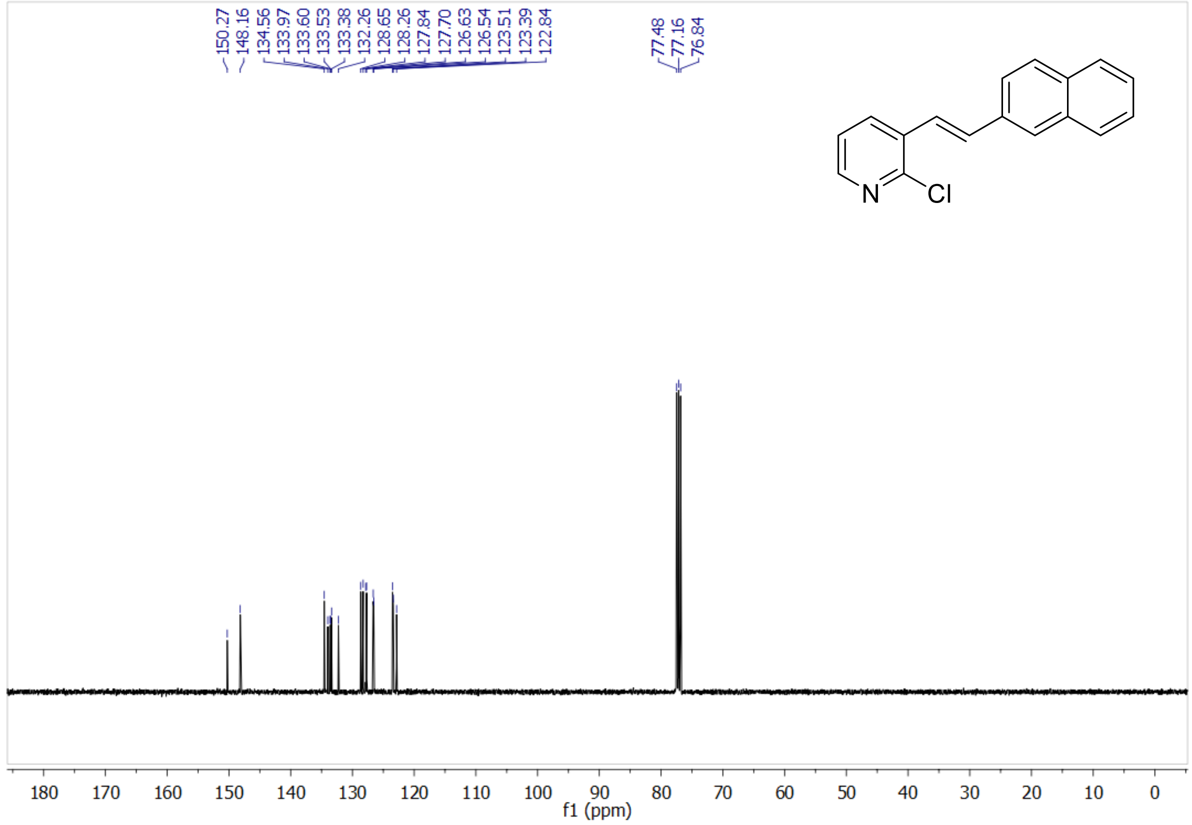

^{1}H-NMR of 5-(2-(benzo[c]phenanthren-2-yl)vinyl)benzo[b]thiophene (400 MHz, $CDCl_3$) (**1p**)

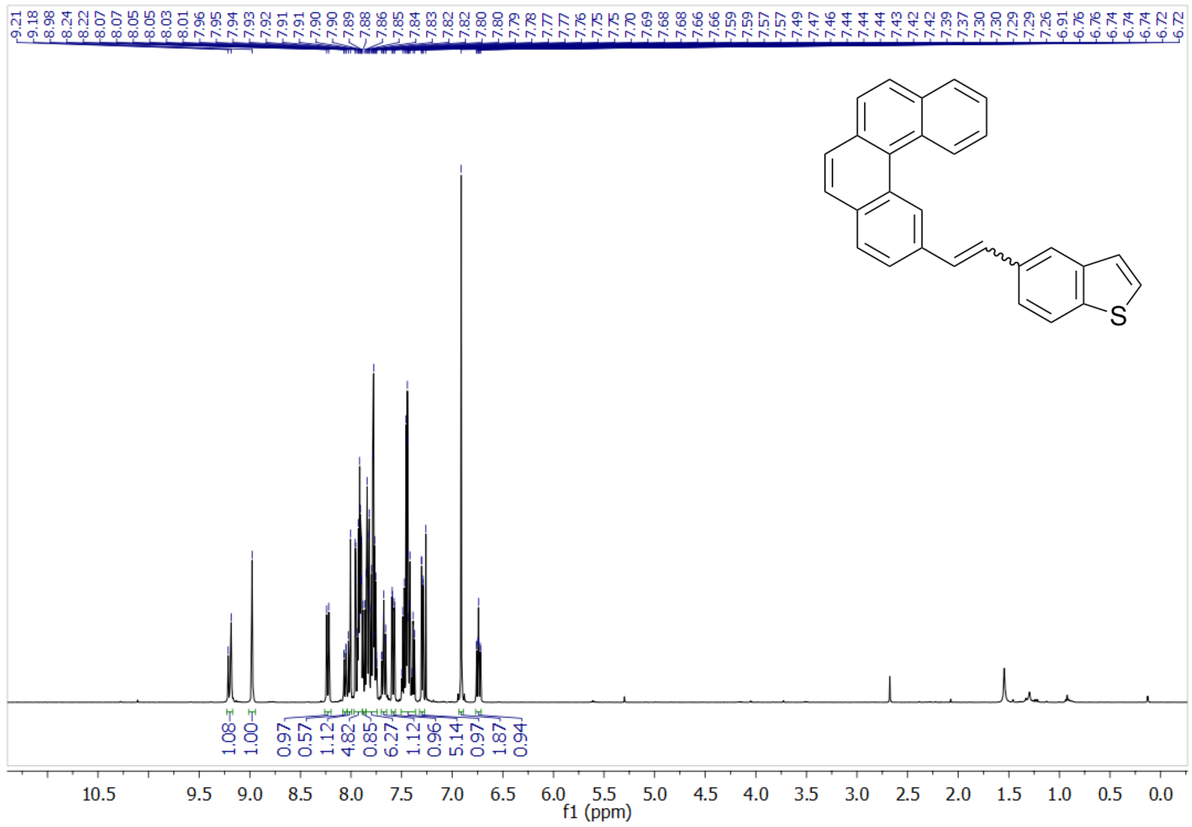

^{13}C-NMR of 5-(2-(benzo[c]phenanthren-2-yl)vinyl)benzo[b]thiophene (101 MHz, $CDCl_3$) (**1p**)

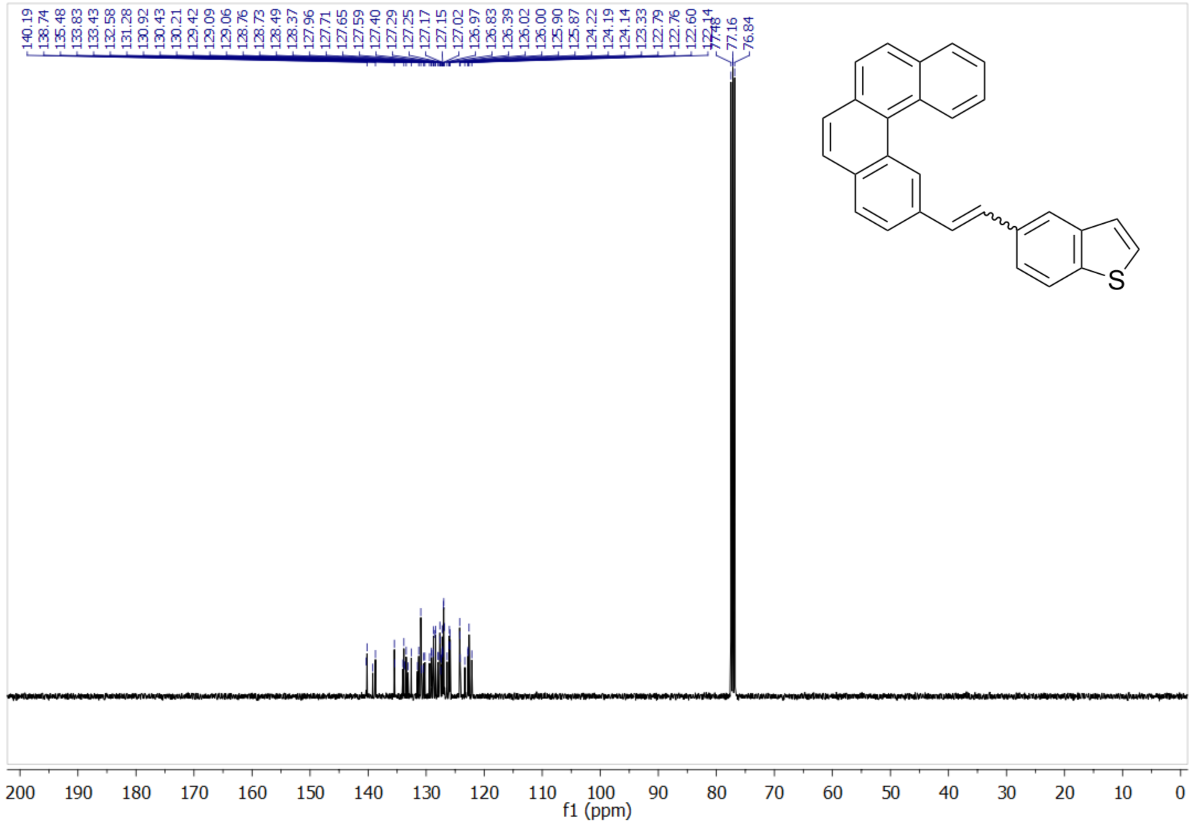

*^{1}H-NMR of 3,6-dibromo-9-((2-(trimethylsilyl)ethoxy)methyl)-9H-carbazole (400 MHz, $CDCl_3$) (**1qa**)*

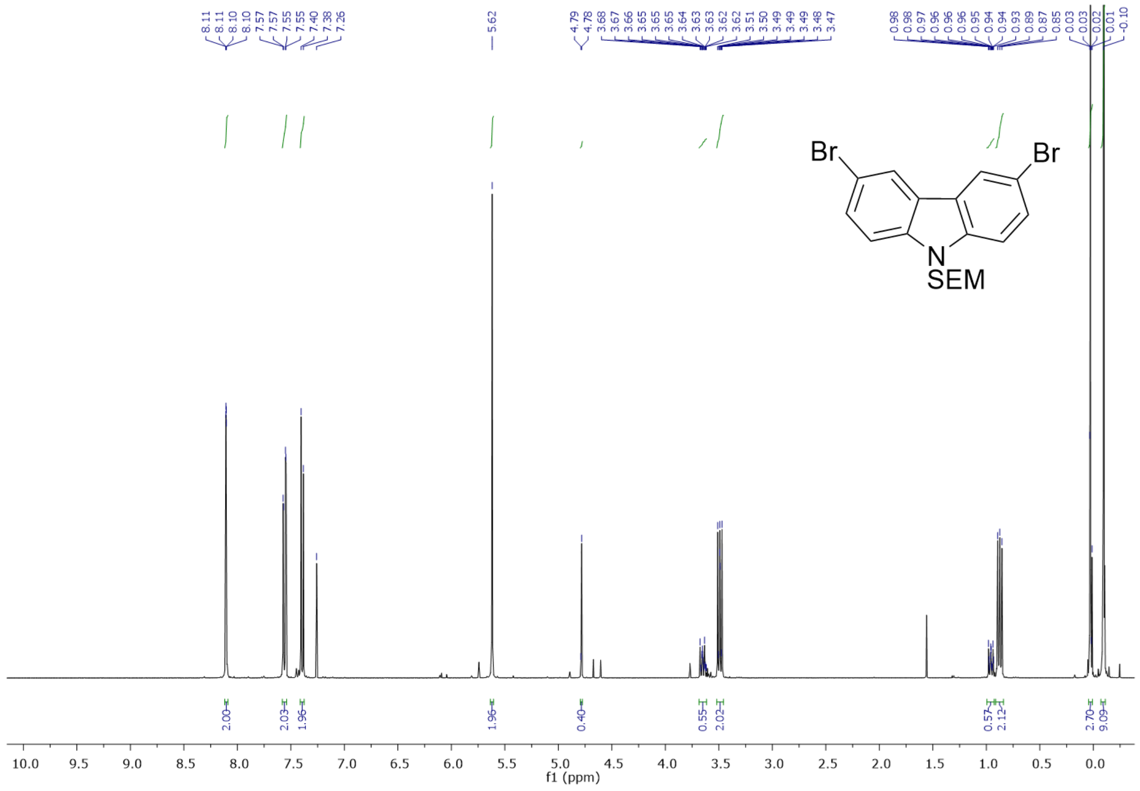

*^{13}C-NMR of 3,6-dibromo-9-((2-(trimethylsilyl)ethoxy)methyl)-9H-carbazole (101 MHz, $CDCl_3$) (**1qa**)*

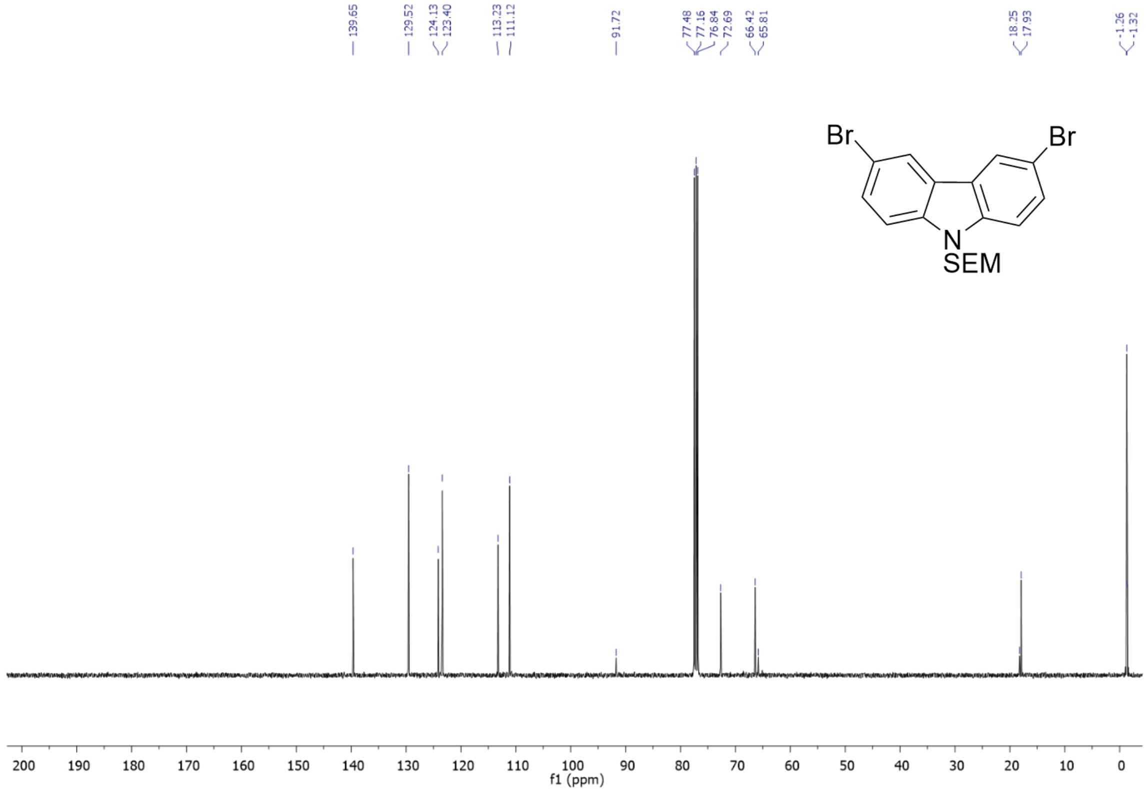

*^{1}H-NMR of 9-((2-(trimethylsilyl)ethoxy)methyl)-9*H*-carbazole-3,6-dicarbaldehyde (400 MHz, $CDCl_3$) (**1qb**)*

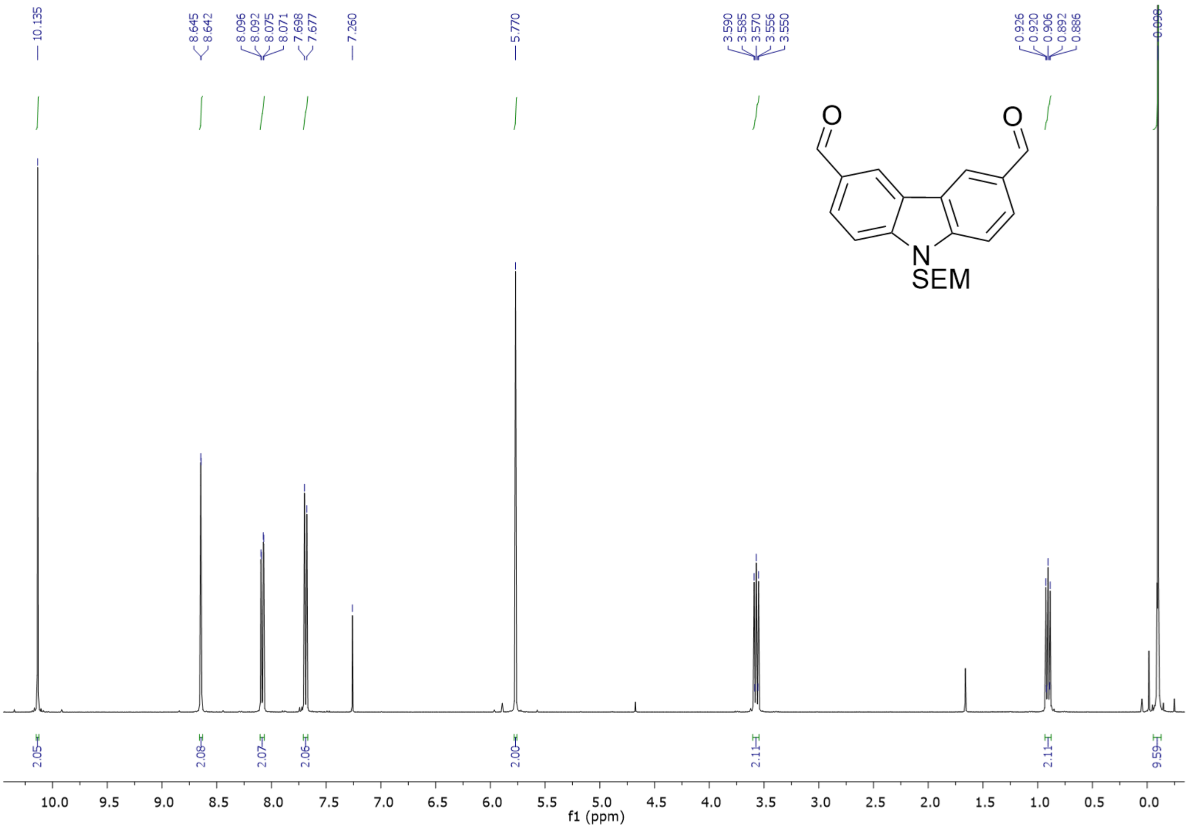

*^{13}C-NMR of 9-((2-(trimethylsilyl)ethoxy)methyl)-9*H*-carbazole-3,6-dicarbaldehyde (101 MHz, $CDCl_3$) (**1qb**)*

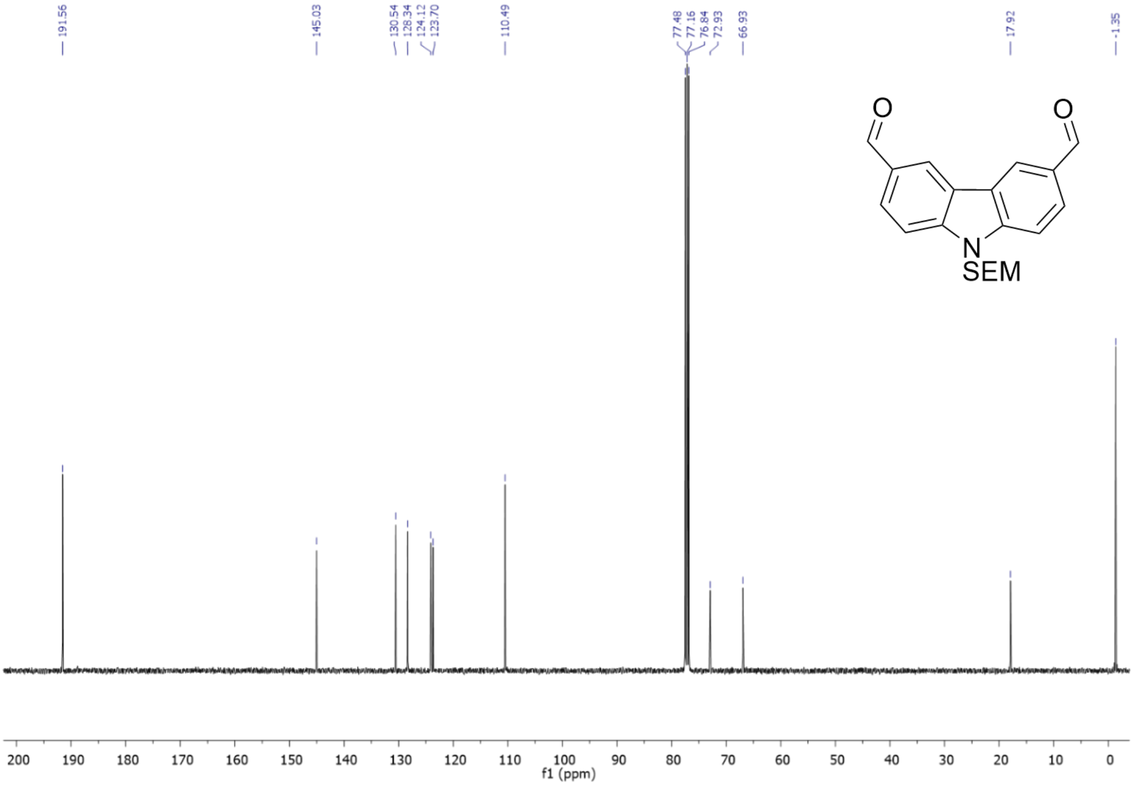

*^{1}H-NMR of 3,6-bis((E)-4-bromostyryl)-9-((2-(trimethylsilyl)ethoxy)methyl)-9H-carbazole (400 MHz, $CDCl_3$) (**1q**)*

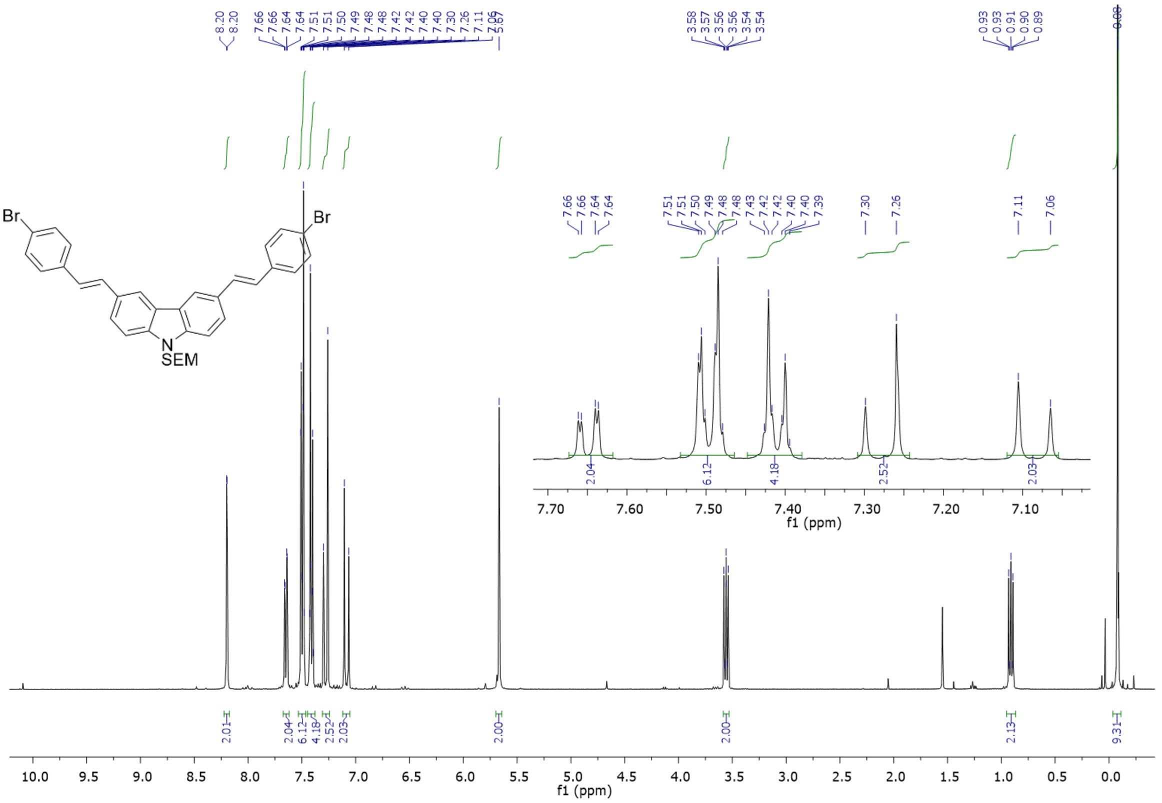

*^{13}C-NMR of 3,6-bis((E)-4-bromostyryl)-9-((2-(trimethylsilyl)ethoxy)methyl)-9H-carbazole (101 MHz, $CDCl_3$) (**1q**)*

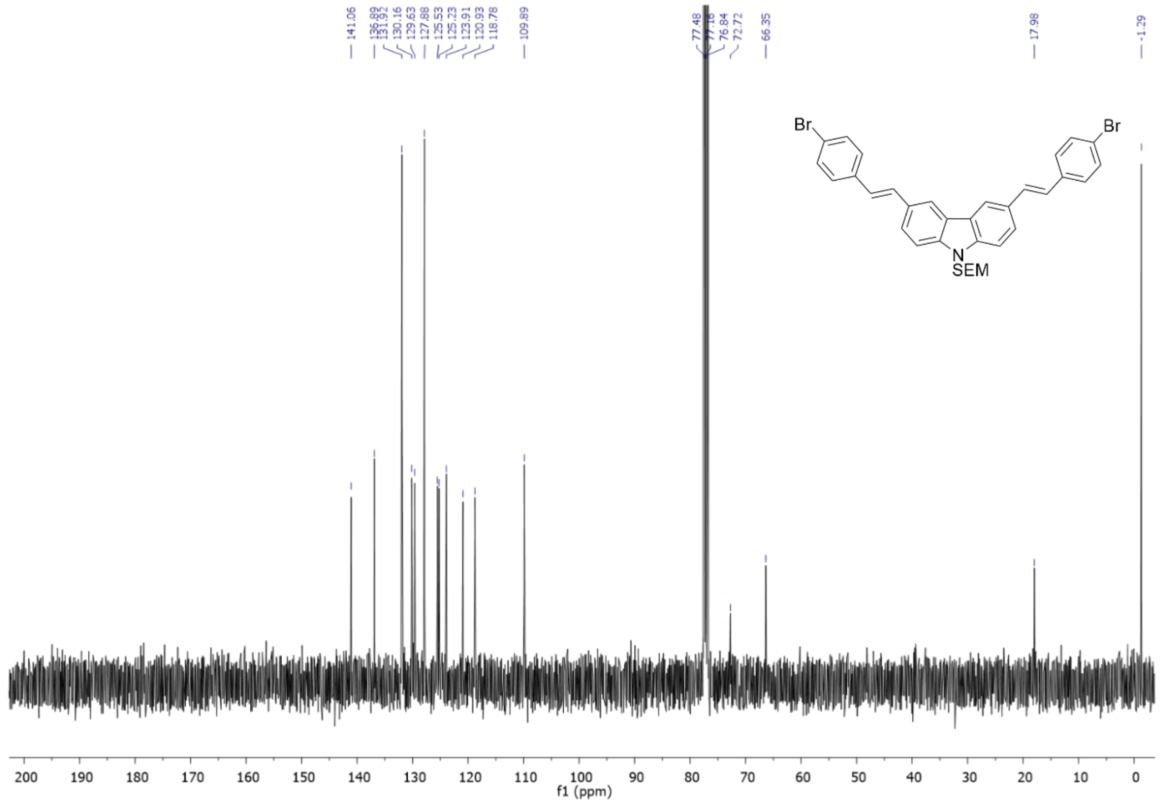

*^{1}H-NMR of [4]thienohelicene (400 MHz, CDCl₃) (**2a**)*

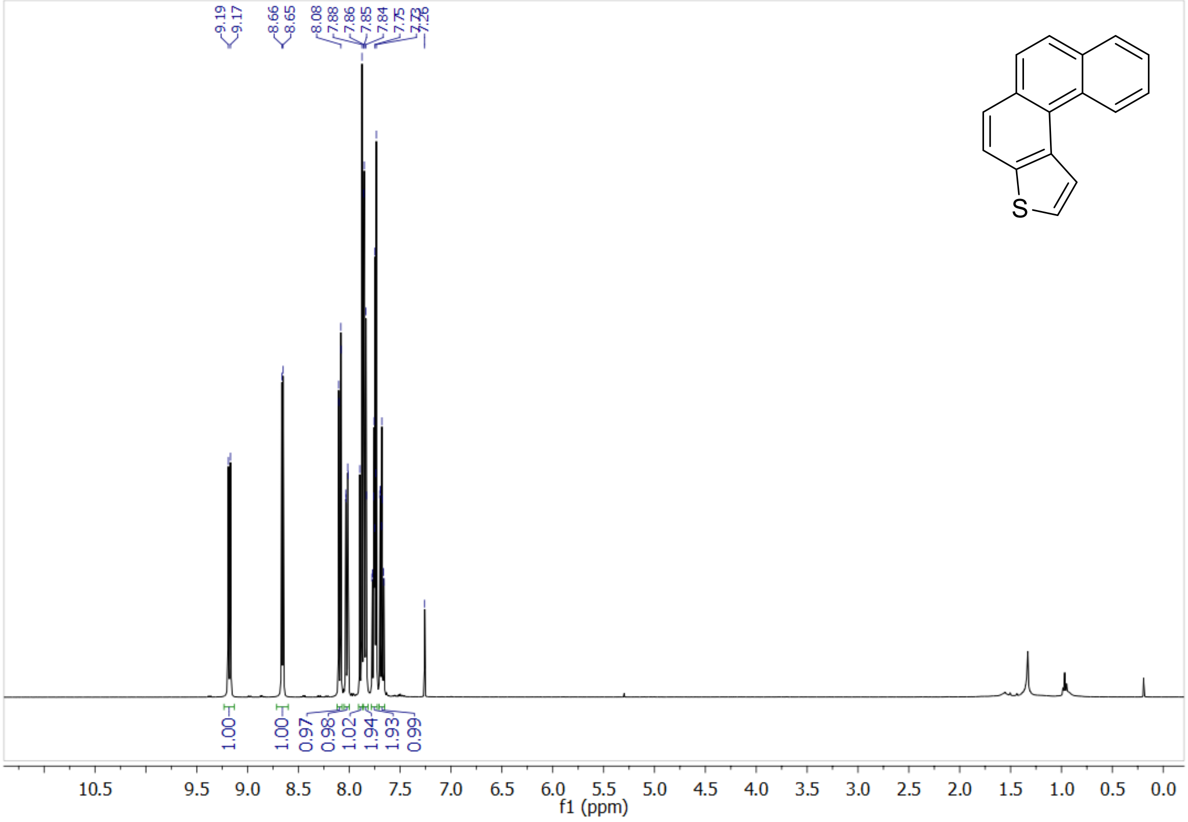

*^{13}C-NMR of [4]thienohelicene (101 MHz, CDCl₃) (**2a**)*

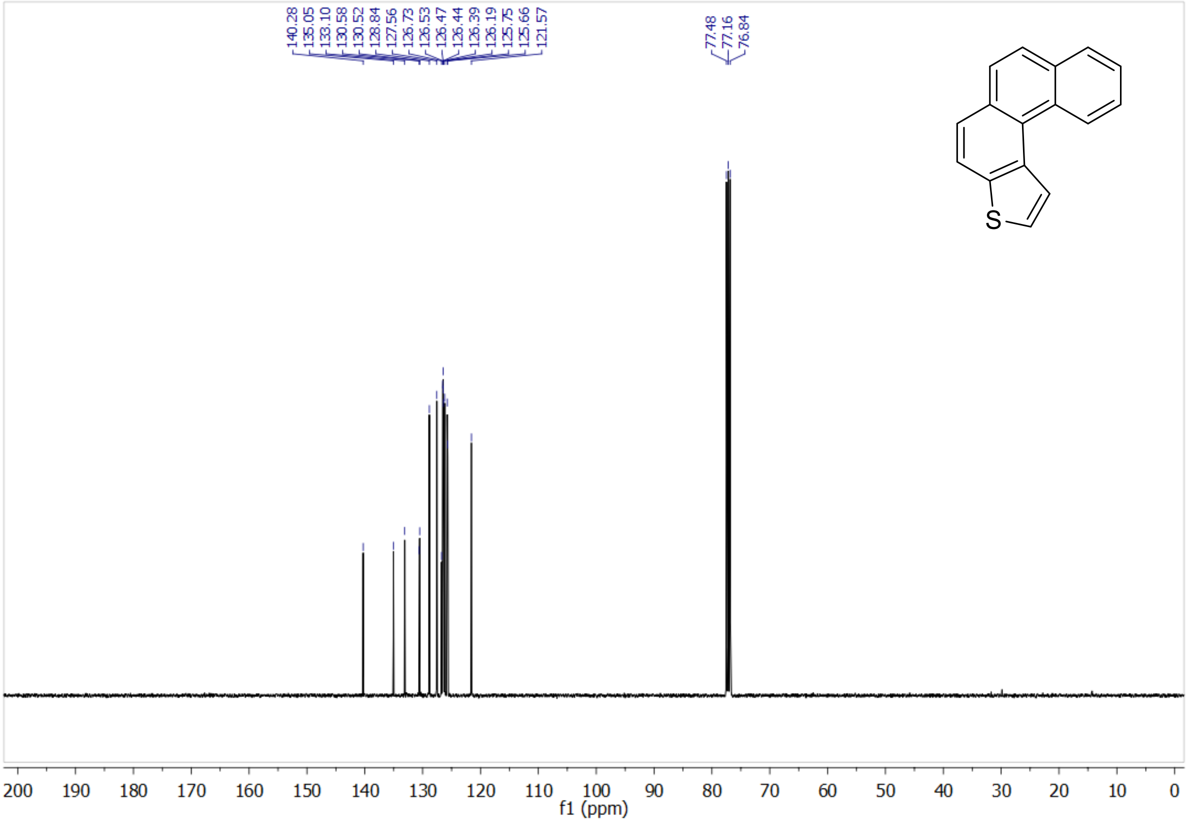

^{1}H-NMR of 2-chloro[4]thienohelicene (400 MHz, $CDCl_3$) (**2b**)

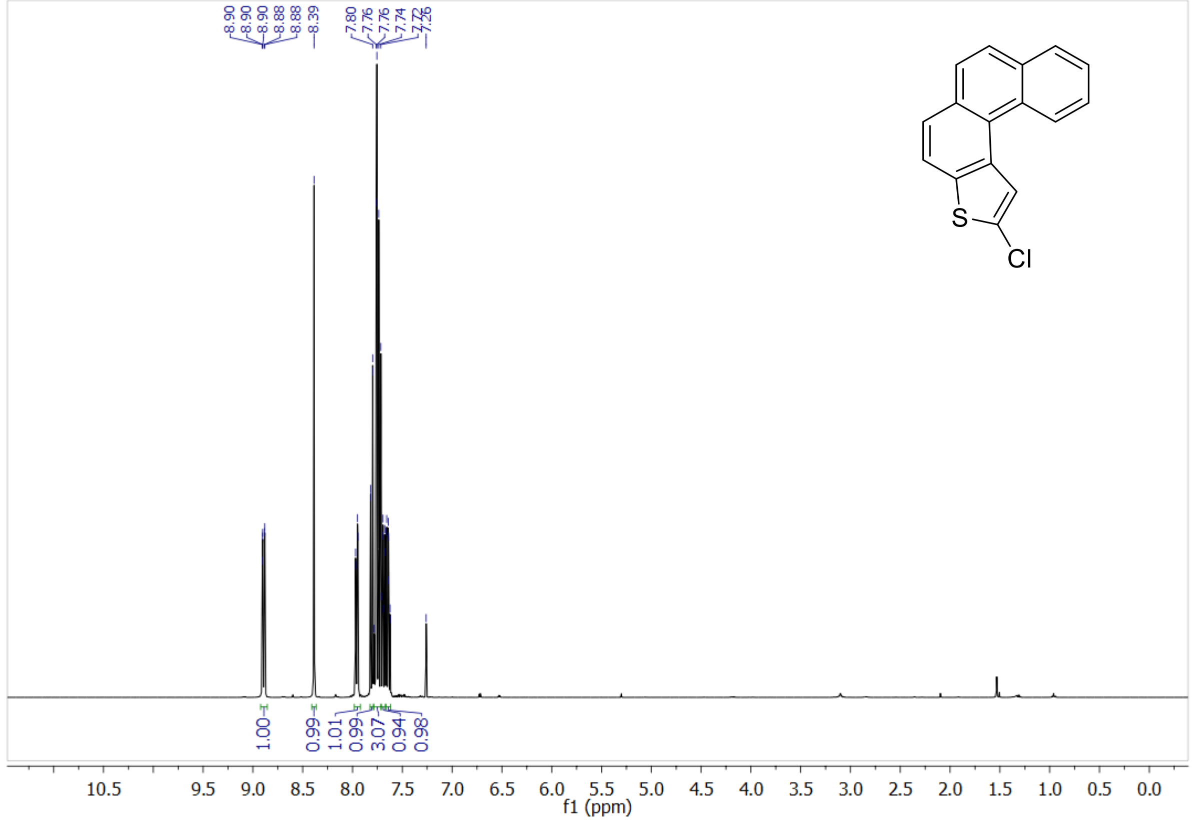

^{13}C-NMR of 2-chloro[4]thienohelicene (101 MHz, $CDCl_3$) (**2b**)

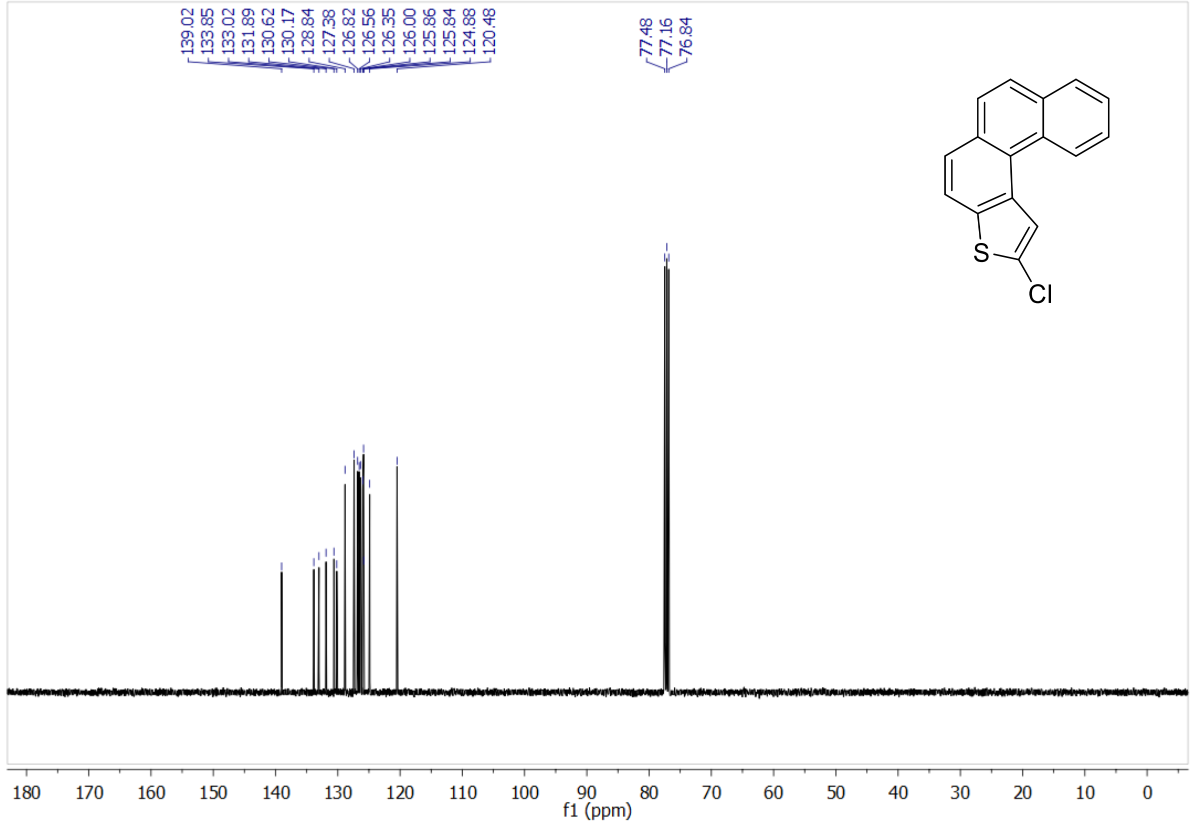

^{1}H-NMR of [4]furohelicene (400 MHz, $CDCl_3$) (**2c**)

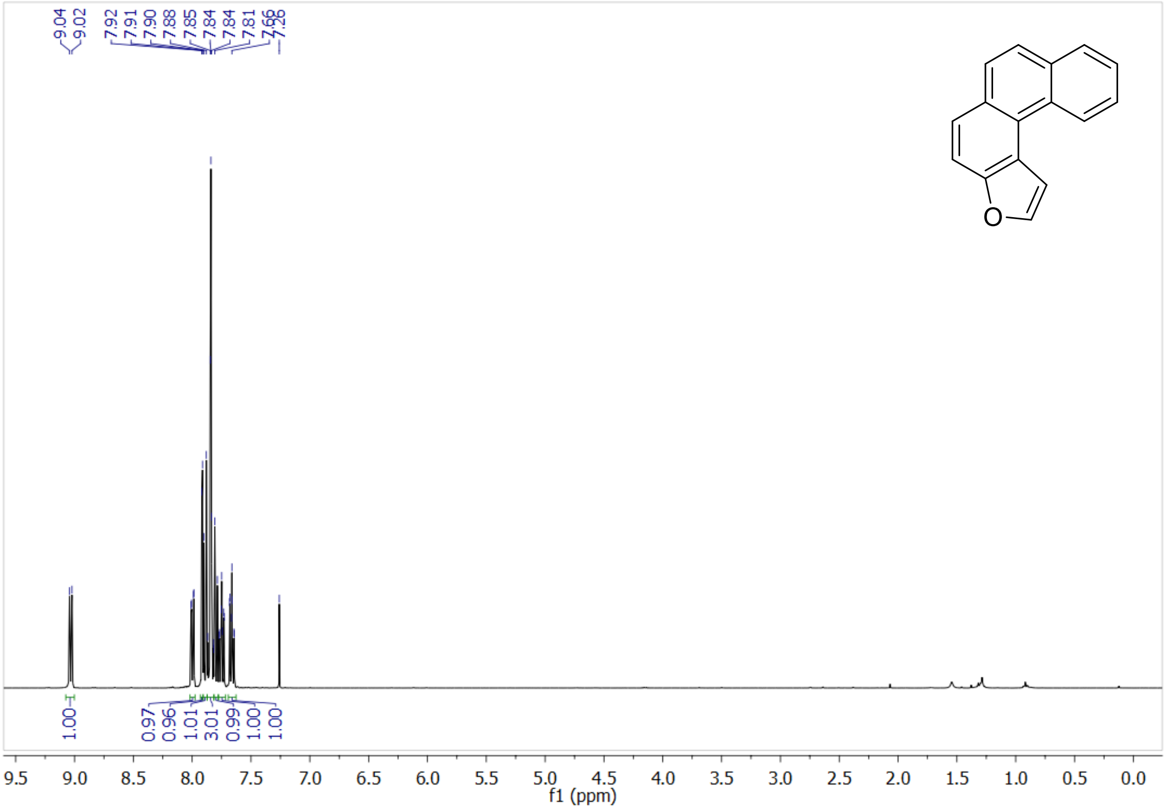

^{13}C-NMR of [4]furohelicene (101 MHz, $CDCl_3$) (**2c**)

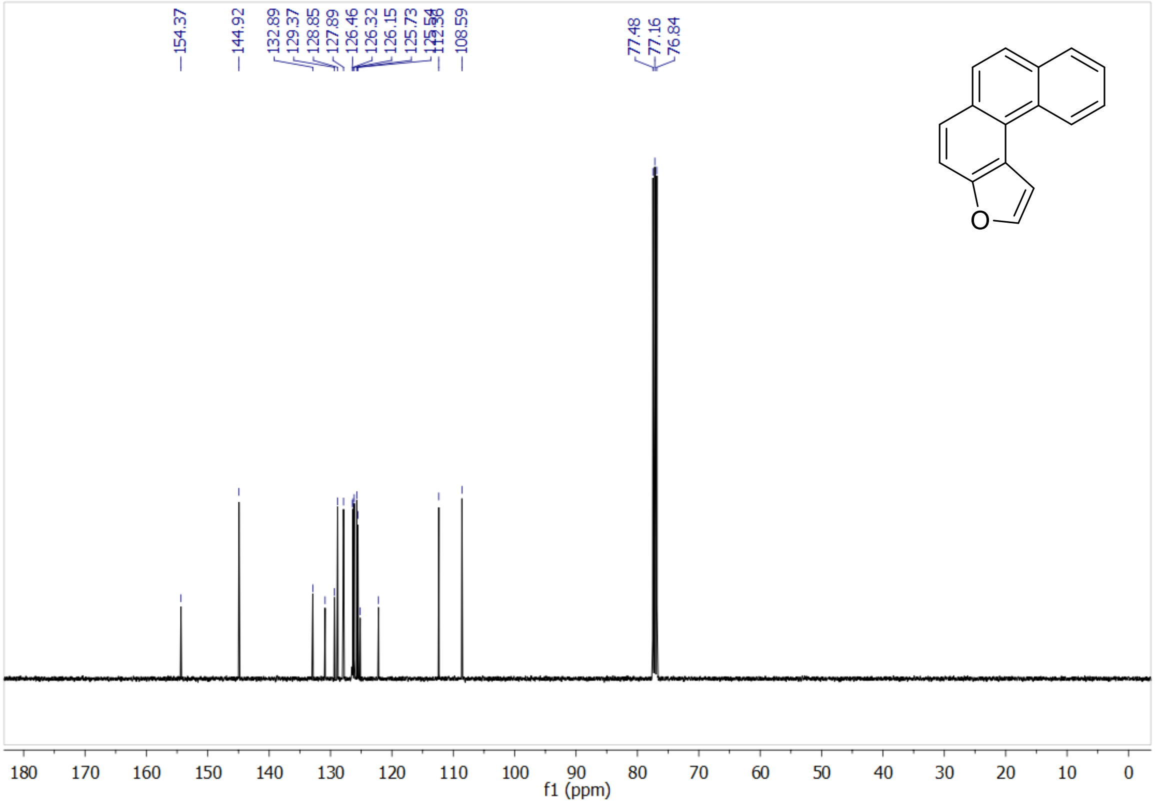

*^{1}H-NMR of 2-chloro[4]furohelicene (400 MHz, $CDCl_3$) (**2d**)*

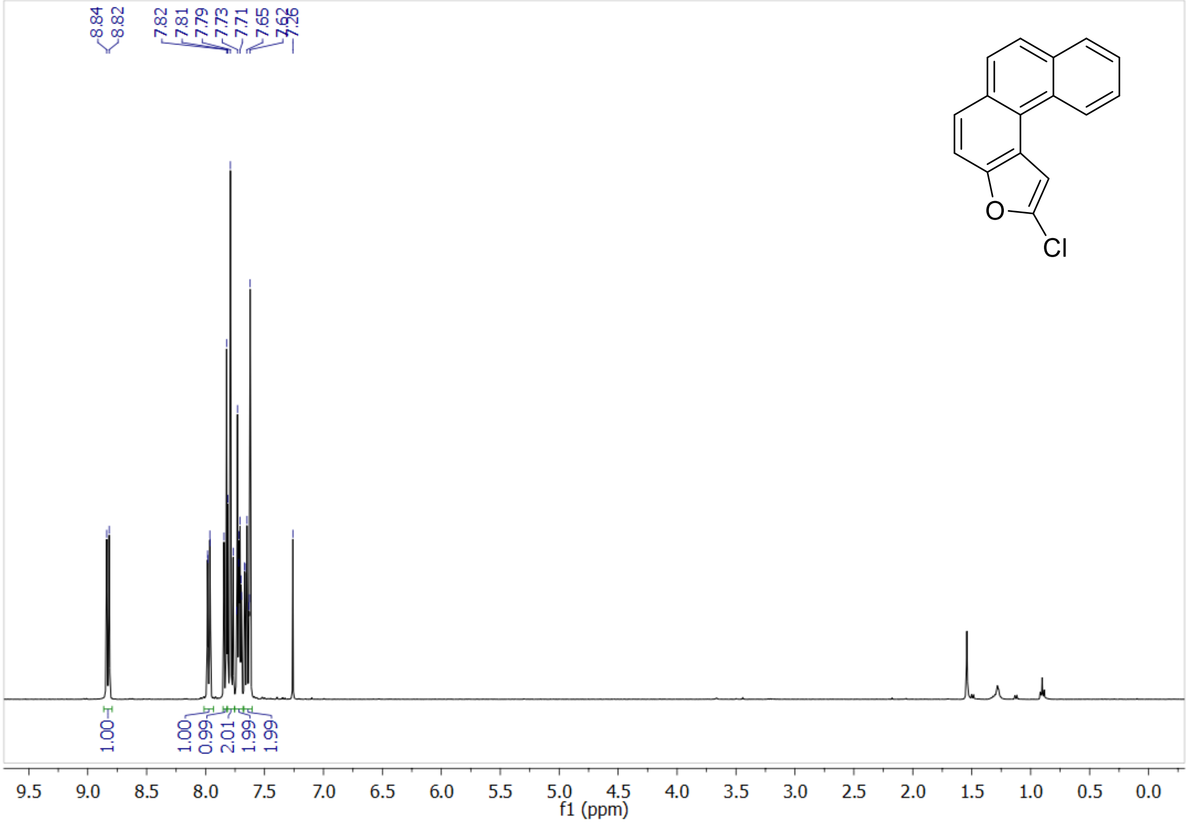

*^{13}C-NMR of 2-chloro[4]furohelicene (101 MHz, $CDCl_3$) (**2d**)*

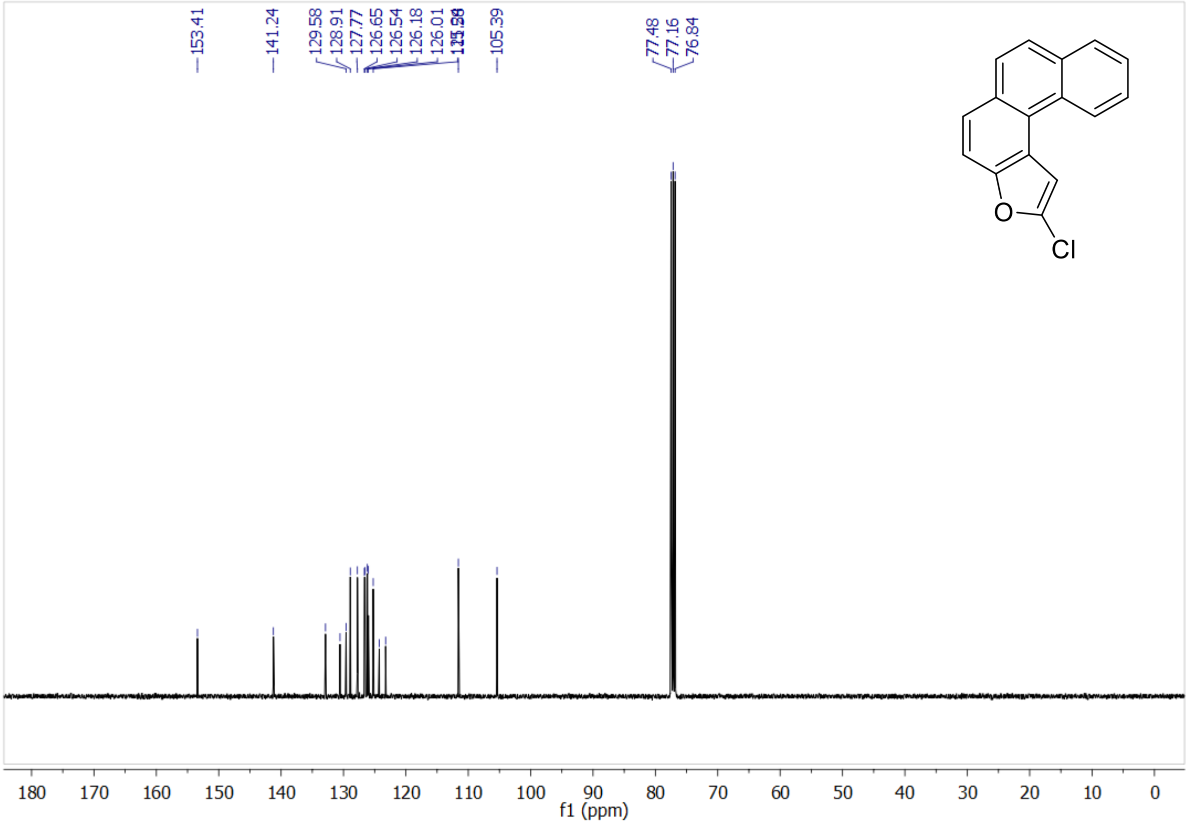

*^{1}H-NMR of [6]thienohelicene (400 MHz, $CDCl_3$) (**2e**)*

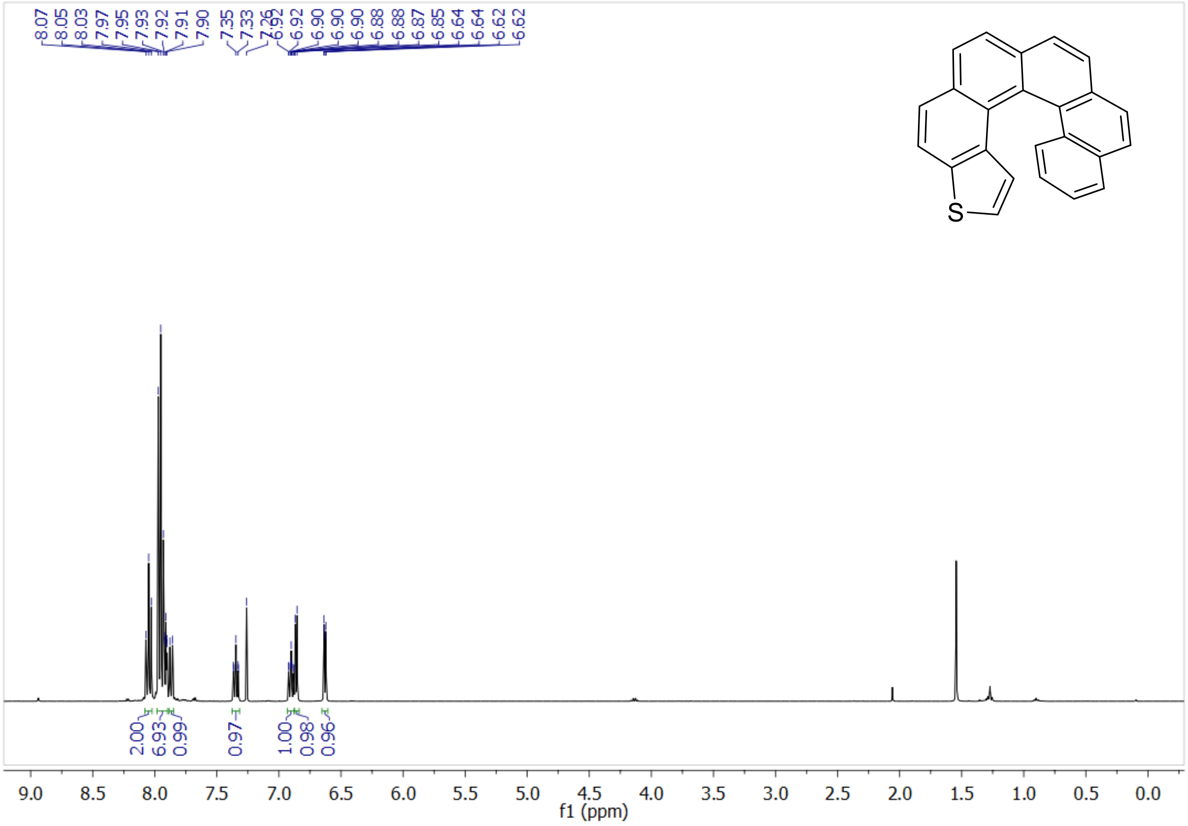

*^{13}C-NMR of [6]thienohelicene (101 MHz, $CDCl_3$) (**2e**)*

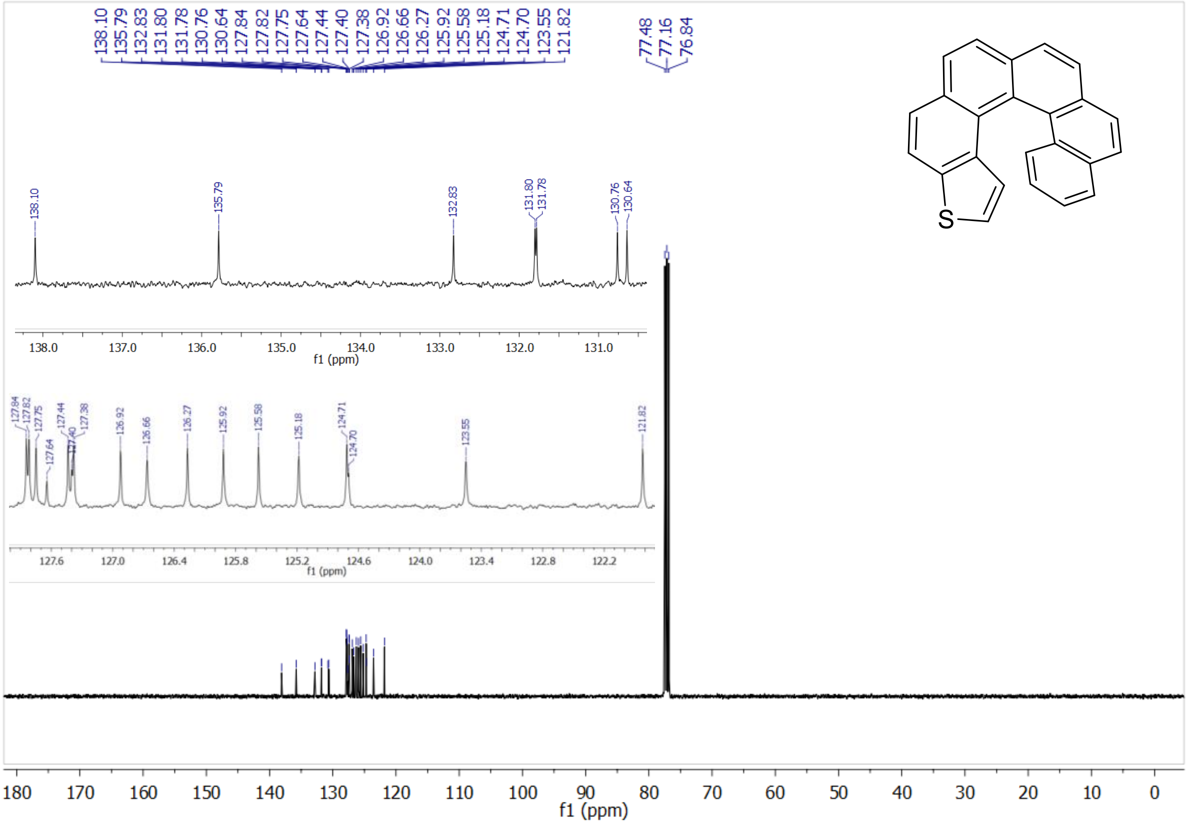

*^{1}H-NMR of [5]thienohelicene (400 MHz, CDCl3) (**2f**)*

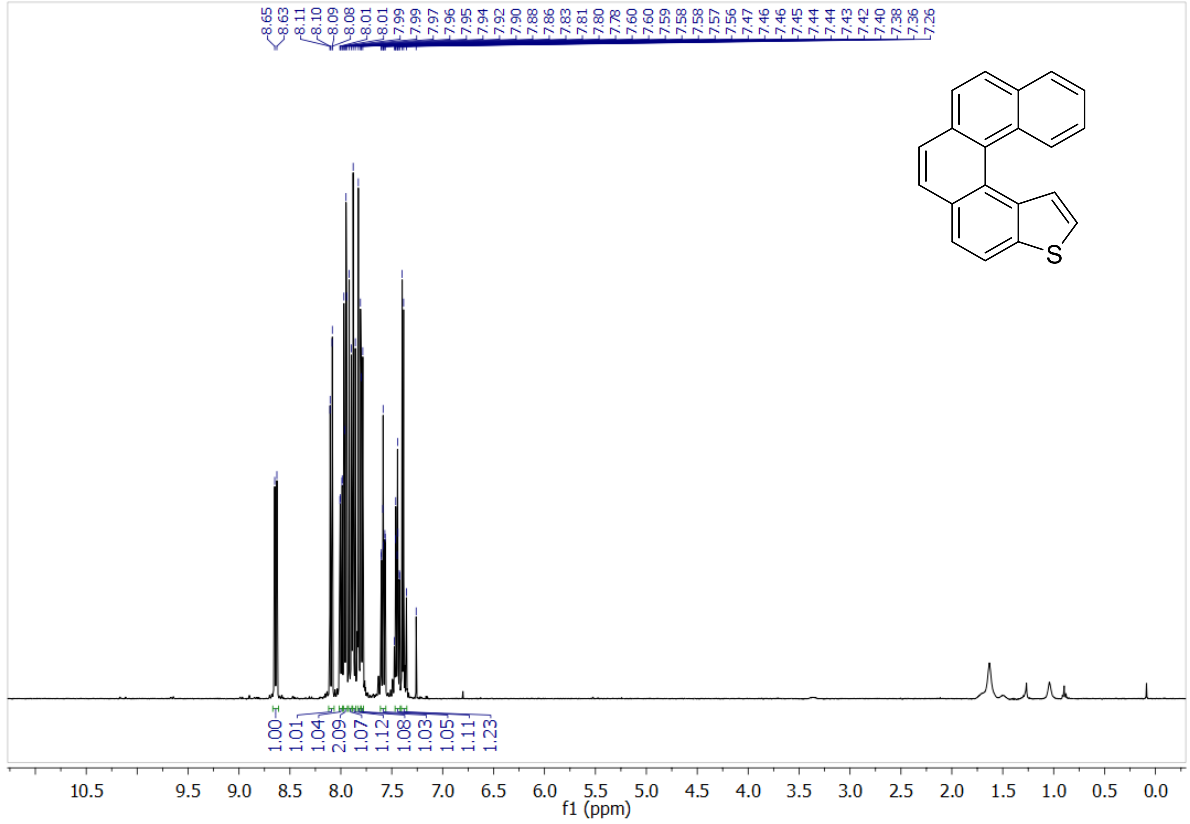

*^{13}C-NMR of [5]thienohelicene (101 MHz, CDCl3) (**2f**)*

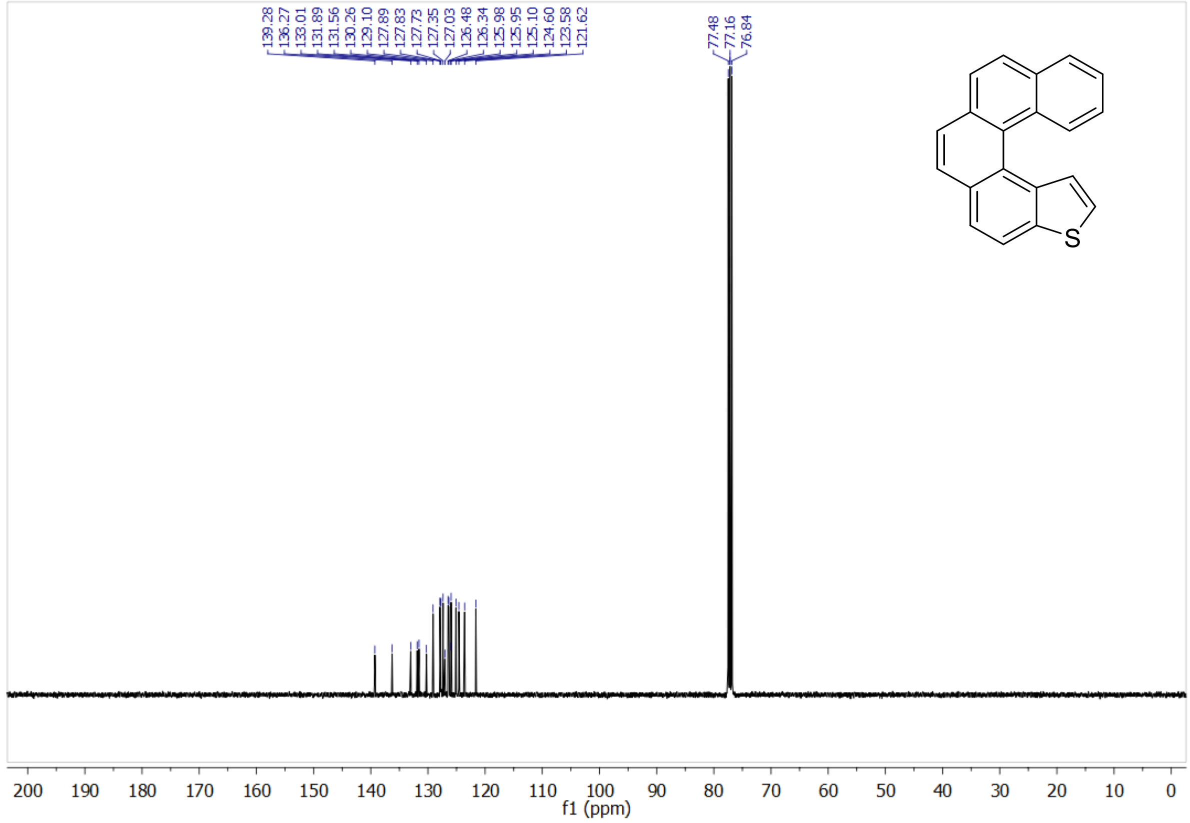

^{1}H-NMR of [7]thienohelicene (400 MHz, $CDCl_3$) (**2g**)

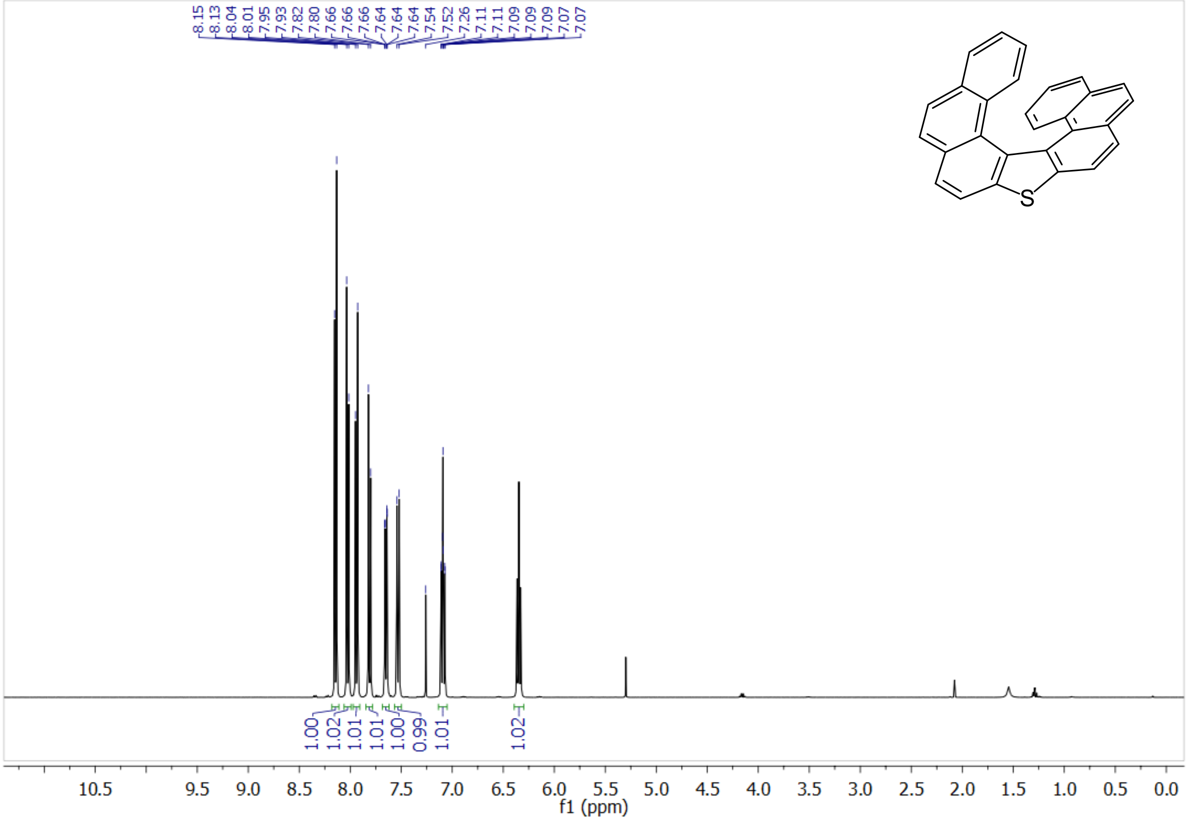

^{13}C-NMR of [7]thienohelicene (101 MHz, $CDCl_3$) (**2g**)

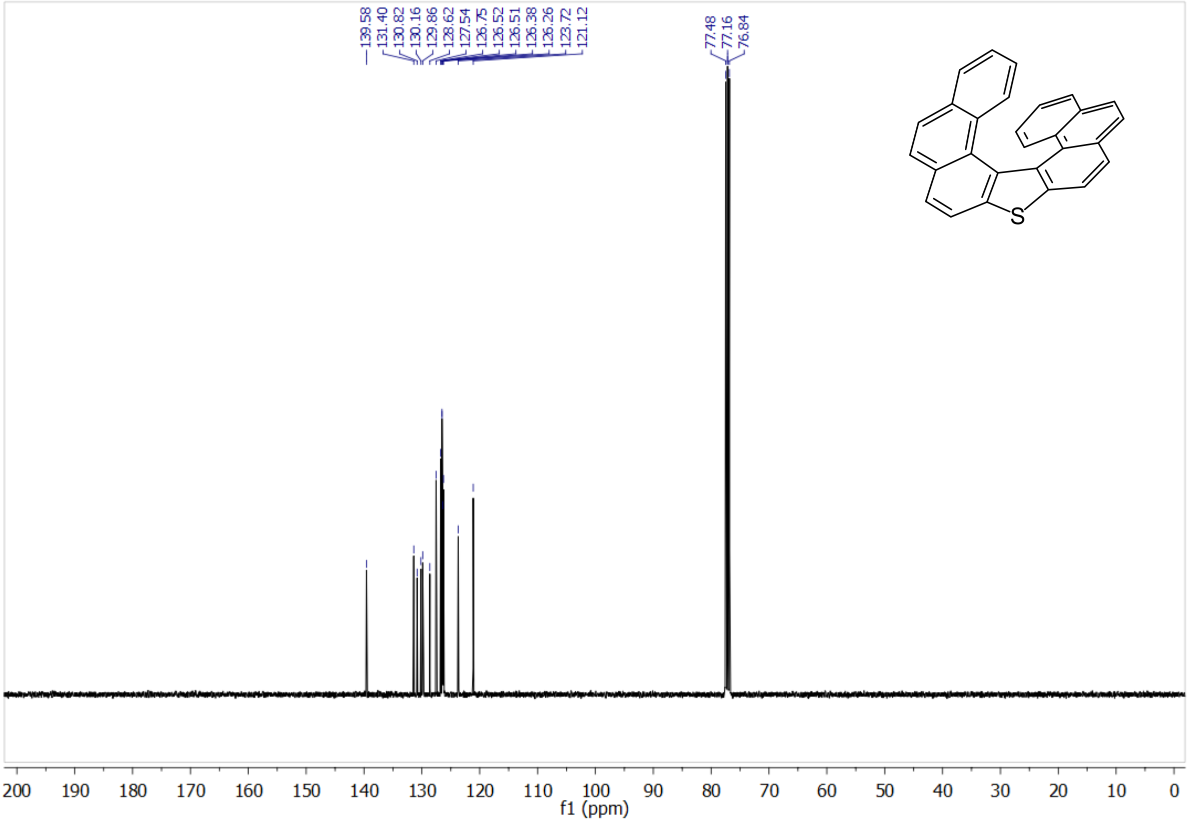

*^{1}H-NMR of [5]pyridohelicene (400 MHz, $CDCl_3$) (**2h**)*

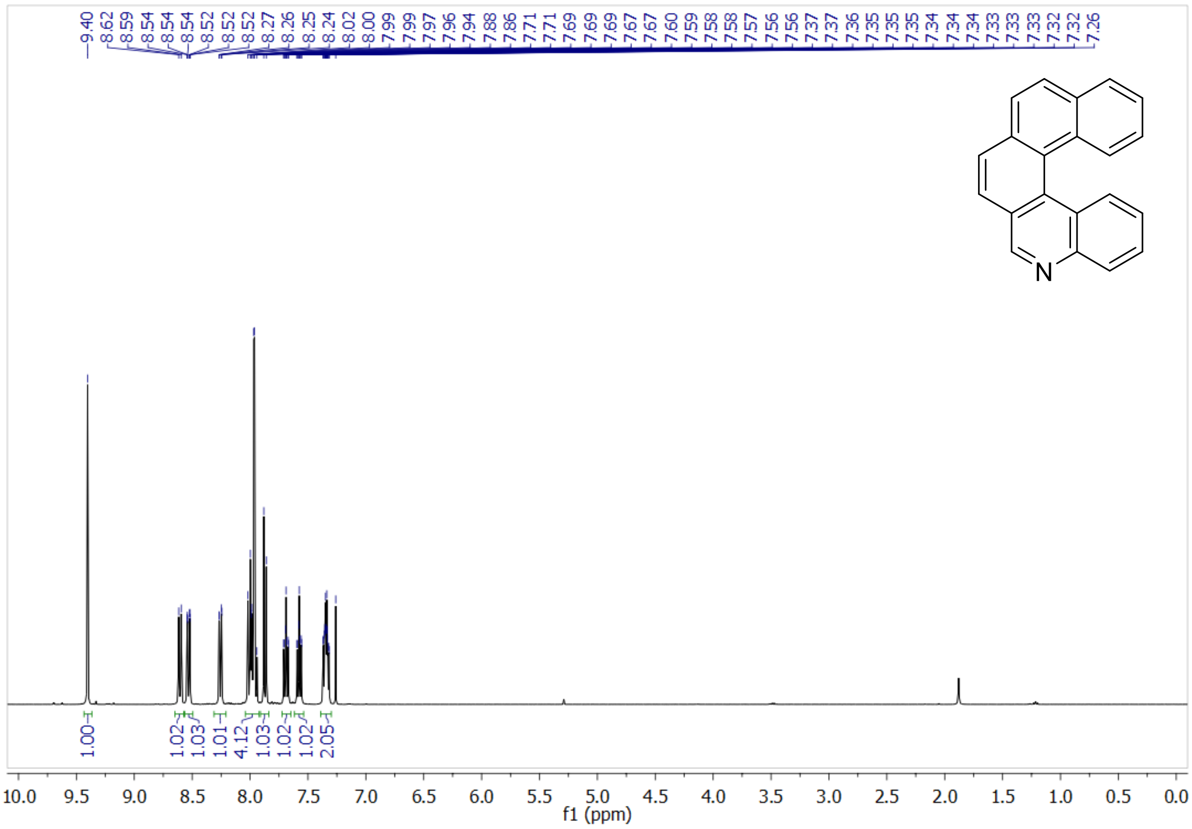

*^{13}C-NMR of [5]pyridohelicene (101 MHz, $CDCl_3$) (**2h**)*

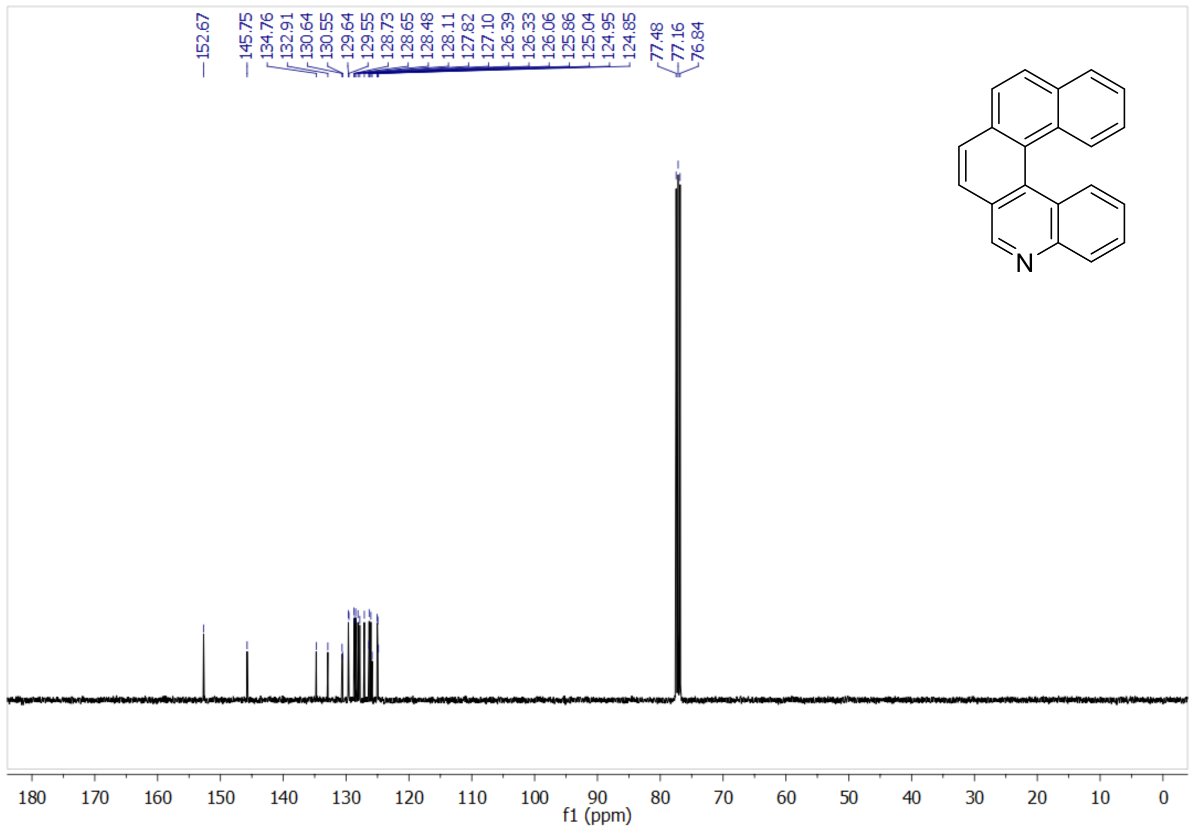

*^{1}H-NMR of [4]thiazolohelicene (400 MHz, CDCl3) (**2i**)*

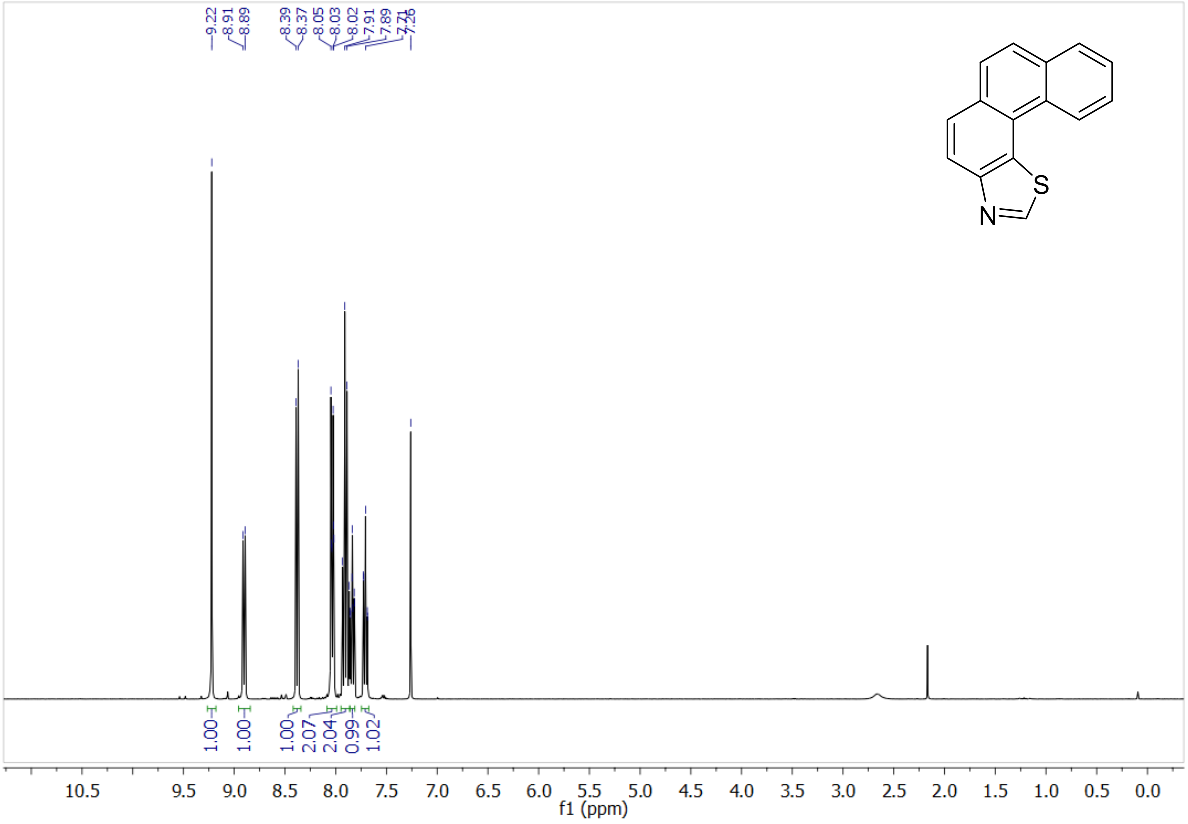

*^{13}C-NMR of [4]thiazolohelicene (101 MHz, CDCl3) (**2i**)*

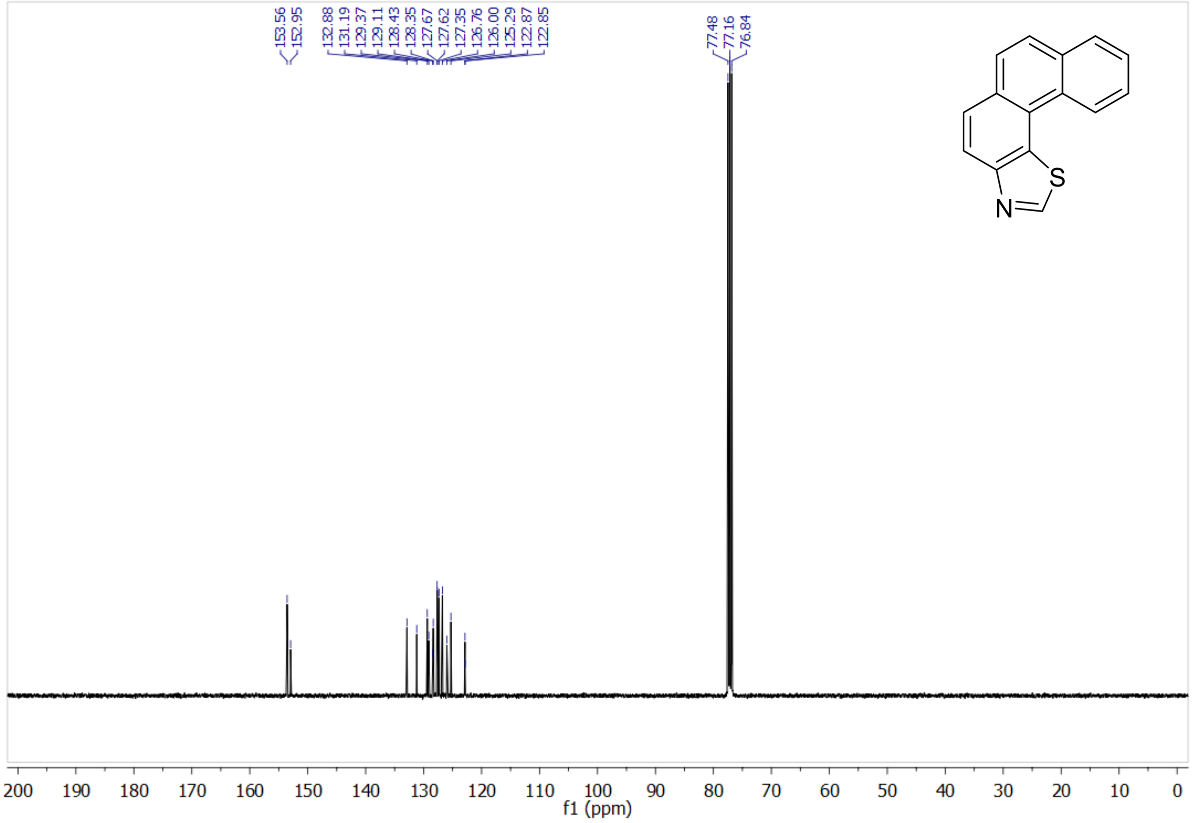

*^{1}H-NMR of 3-isothiocyanatophenanthrene (400 MHz, CDCl3) (**S2i'**)*

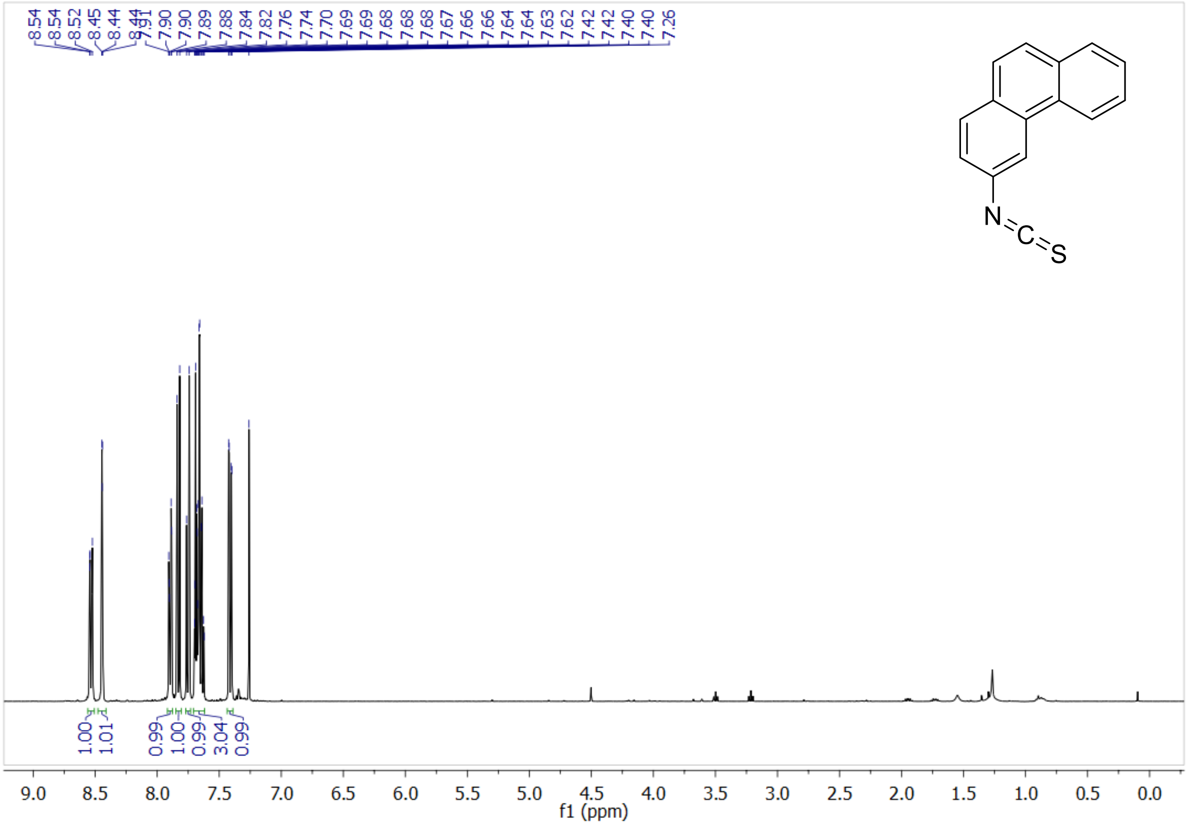

*^{13}C-NMR of 3-isothiocyanatophenanthrene (101 MHz, CDCl3) (**S2i'**)*

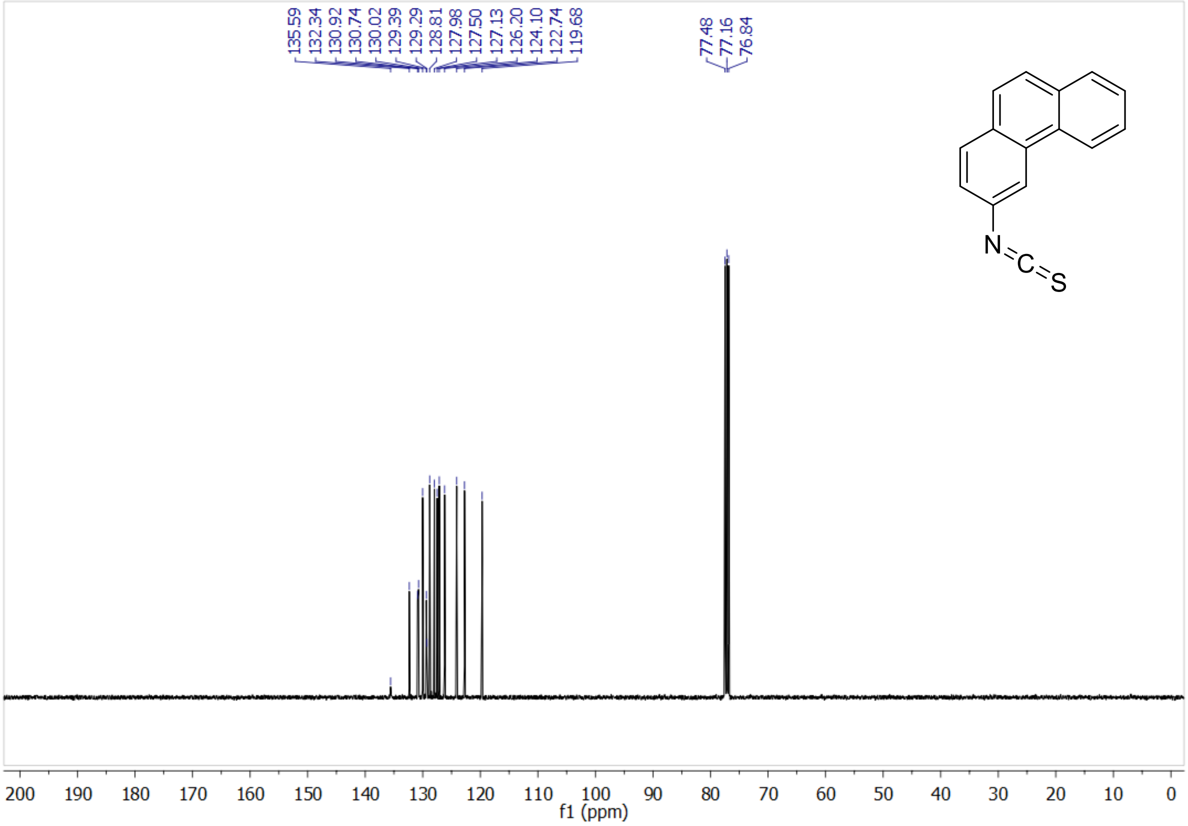

*^{1}H-NMR of [5]oxazolohelicene (400 MHz, $CDCl_3$) (**2k**)*

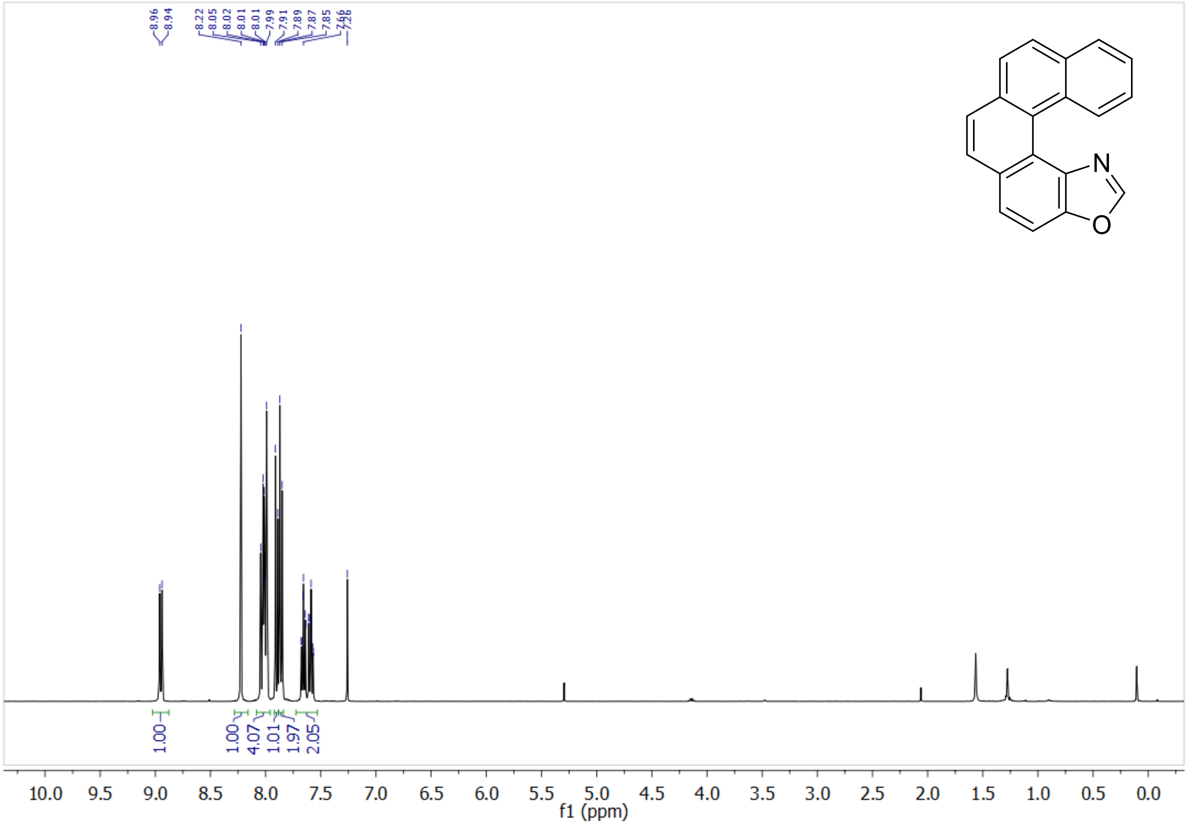

*^{13}C-NMR of [5]oxazolohelicene (101 MHz, $CDCl_3$) (**2k**)*

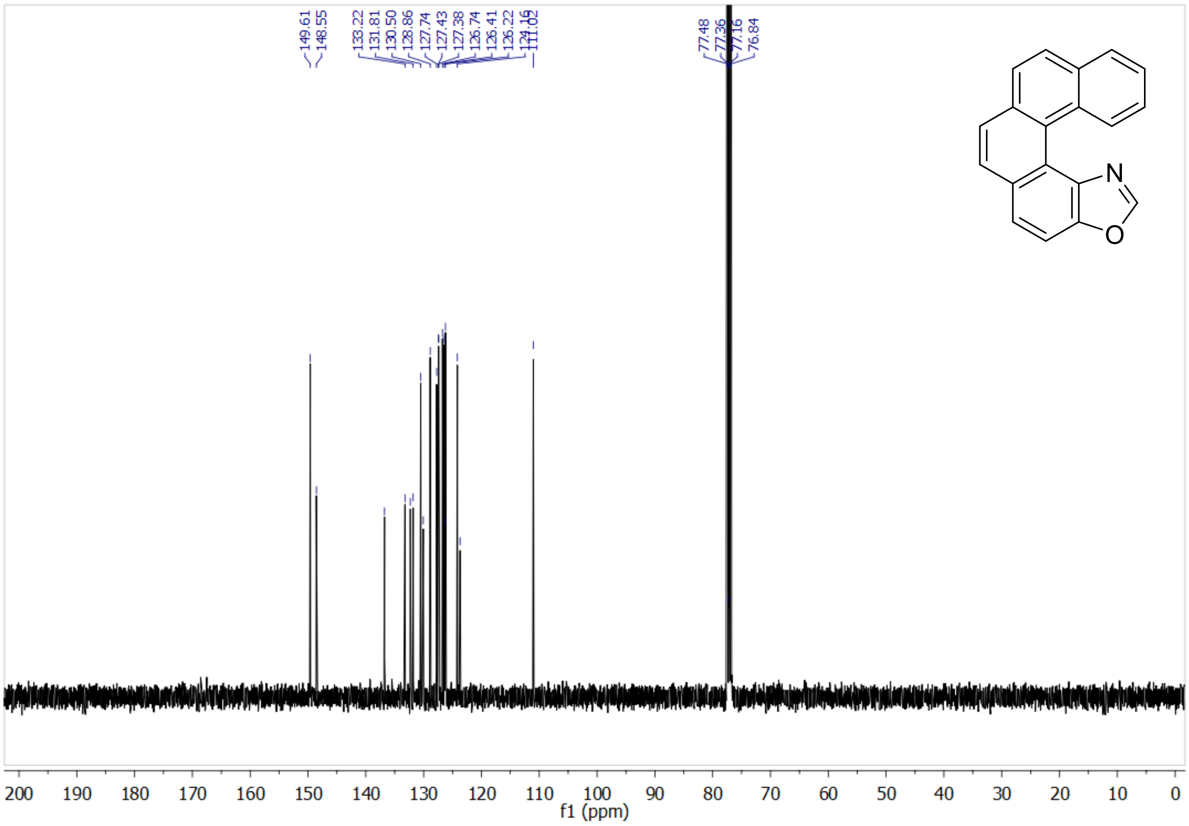

*^{1}H-NMR of 1-chloro[4]pyridohelicene (400 MHz, $CDCl_3$) (**2m**)*

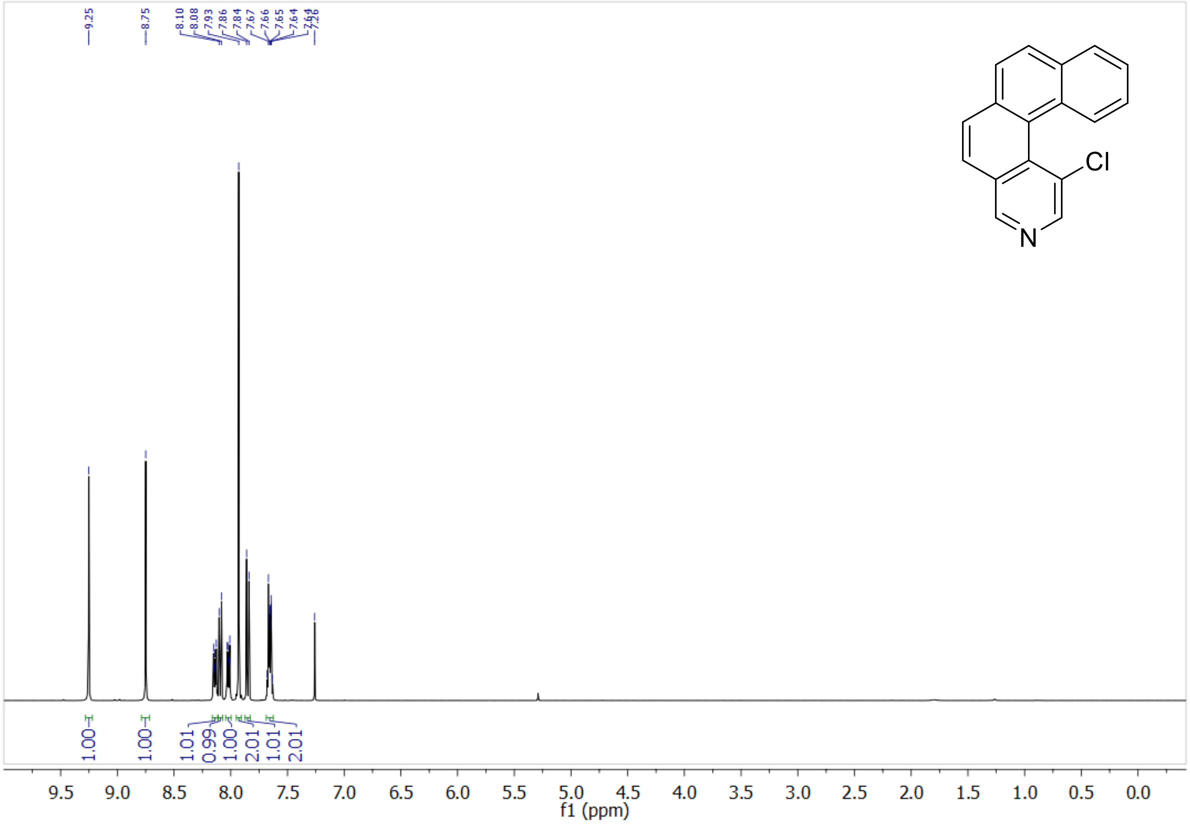

*^{13}C-NMR of 1-chloro[4]pyridohelicene (101 MHz, $CDCl_3$) (**2m**)*

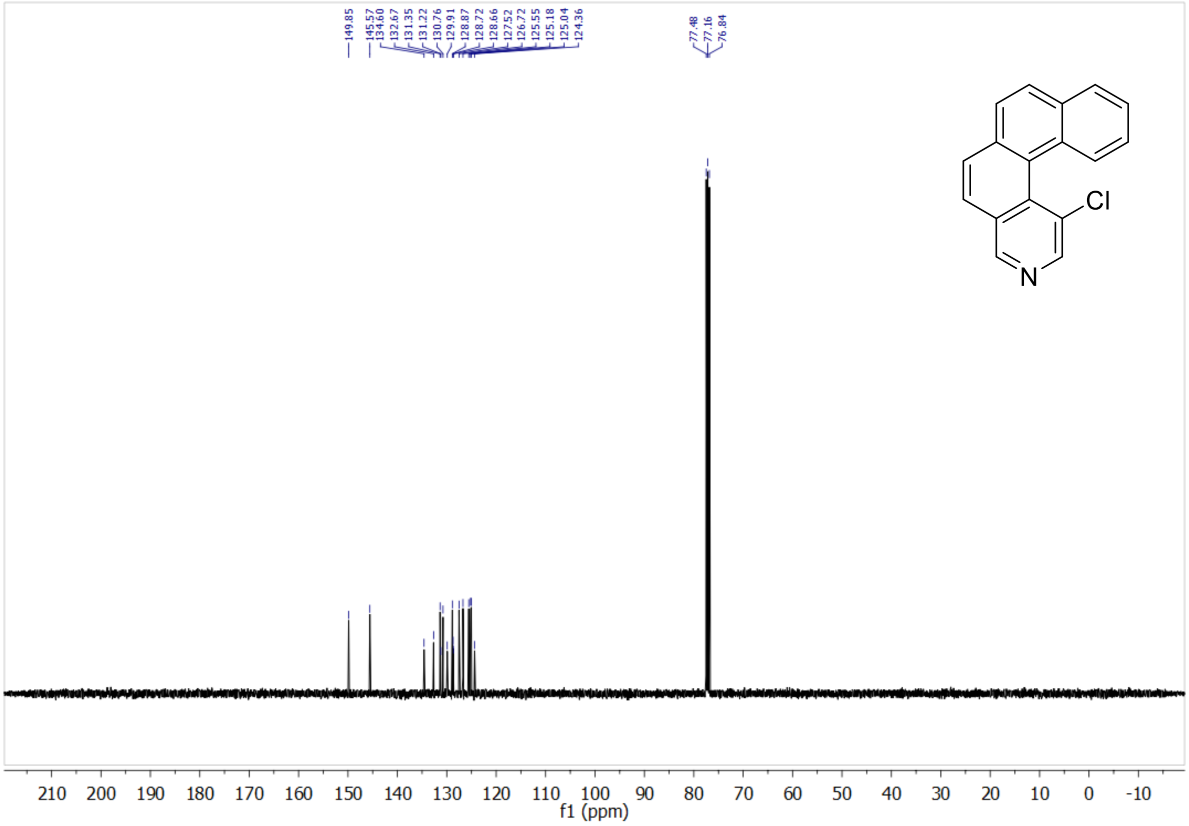

*^{1}H-NMR of 3-chloro[4]pyridohelicene (400 MHz, $CDCl_3$) (**2m'**)*

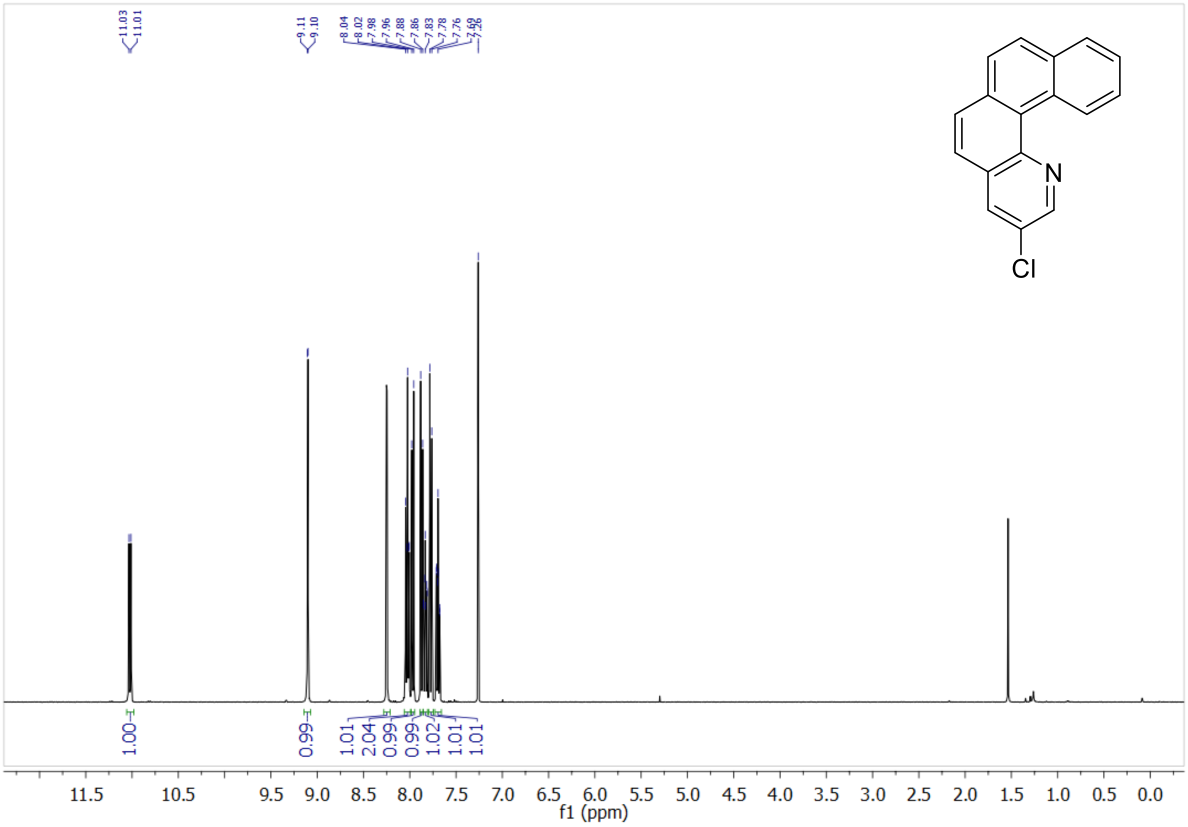

*^{13}C-NMR of 3-chloro[4]pyridohelicene (101 MHz, $CDCl_3$) (**2m'**)*

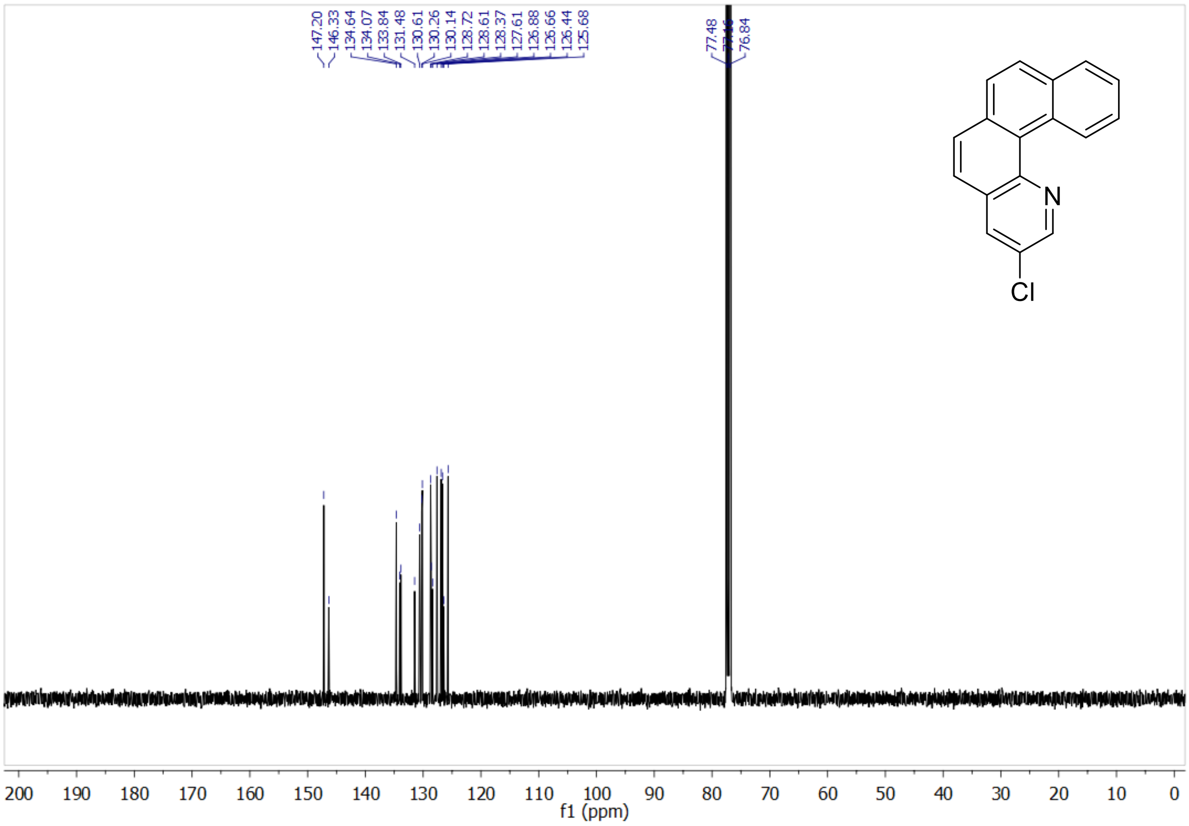

*^{1}H-NMR of [5]thiadiazolohelicene (400 MHz, $CDCl_3$) (**2n**)*

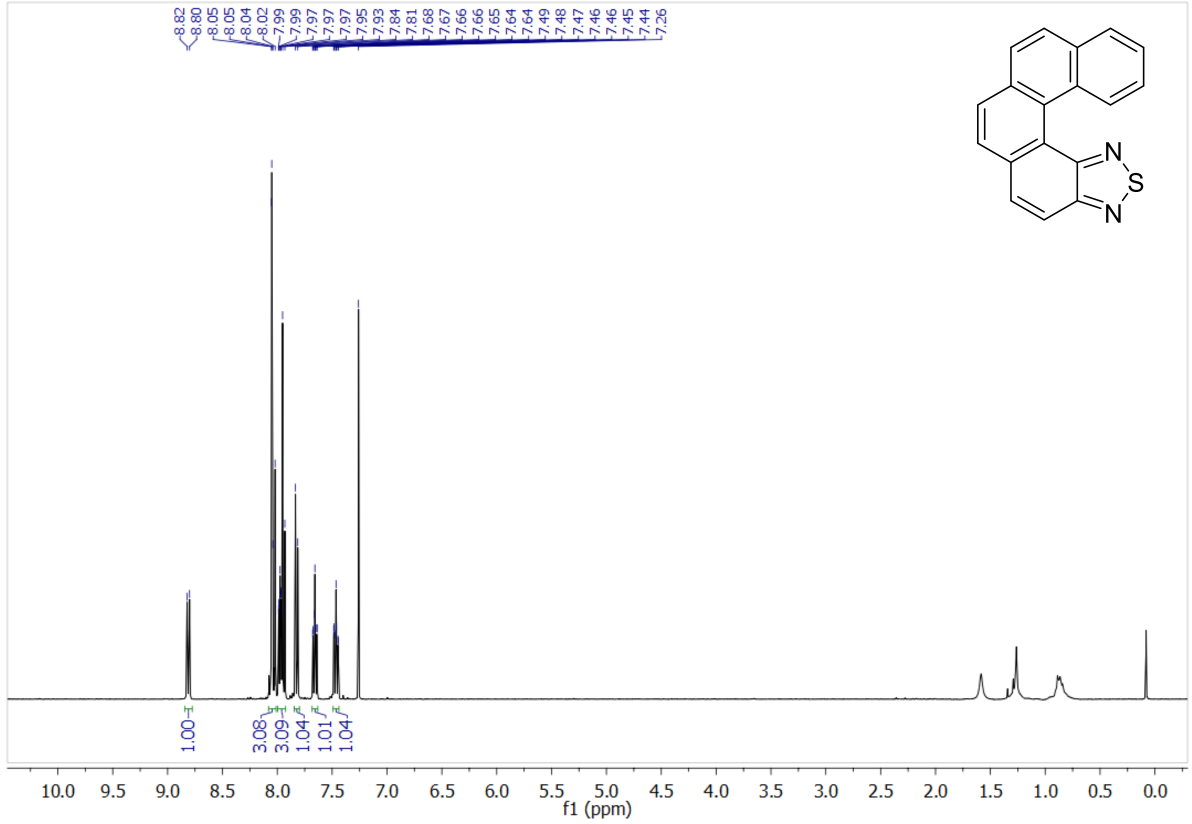

*^{13}C-NMR of [5]thiadiazolohelicene (101 MHz, $CDCl_3$) (**2n**)*

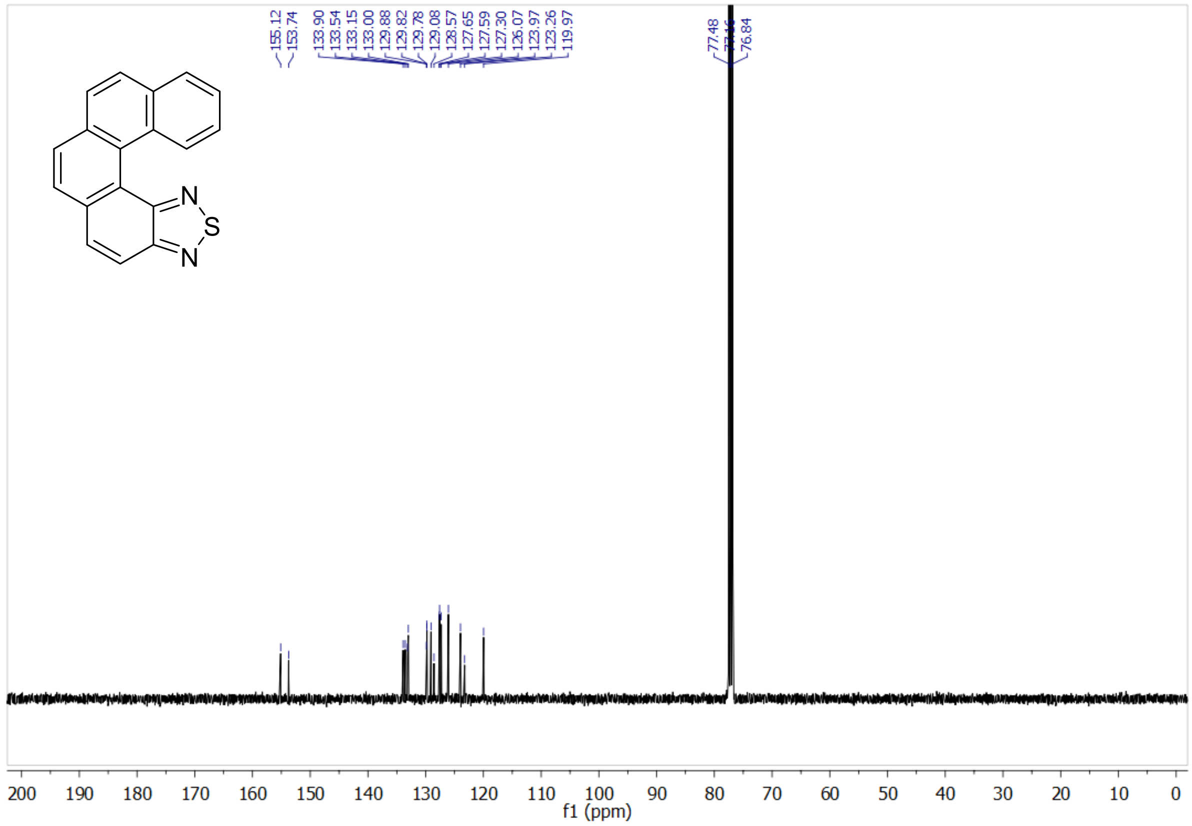

*^{1}H-NMR of tetrapheno[2,3-c][1,2,5]thiadiazole (400 MHz, $CDCl_3$) (**2n'**)*

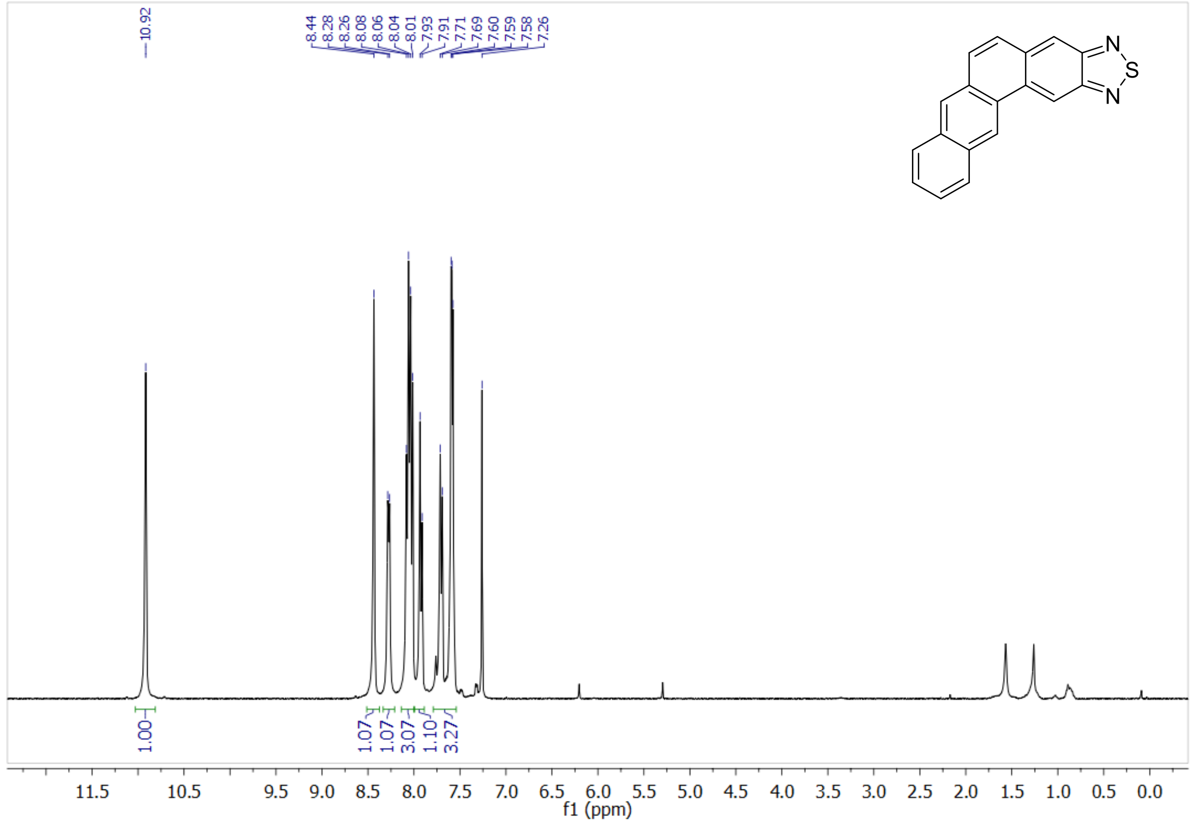

*^{13}C-NMR of tetrapheno[2,3-c][1,2,5]thiadiazole (101 MHz, $CDCl_3$) (**2n'**)*

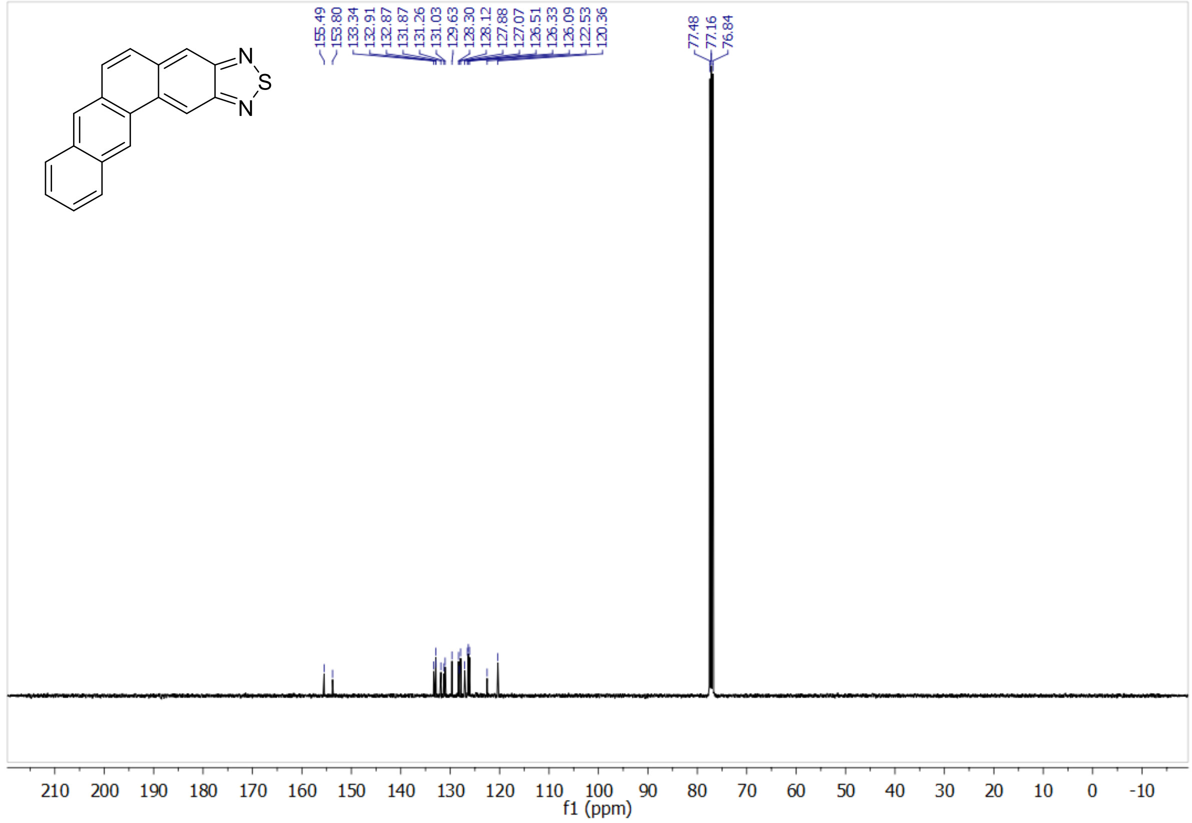

*^{1}H-NMR of 4-chloro[4]pyridohelicene (400 MHz, $CDCl_3$) (**2o**)*

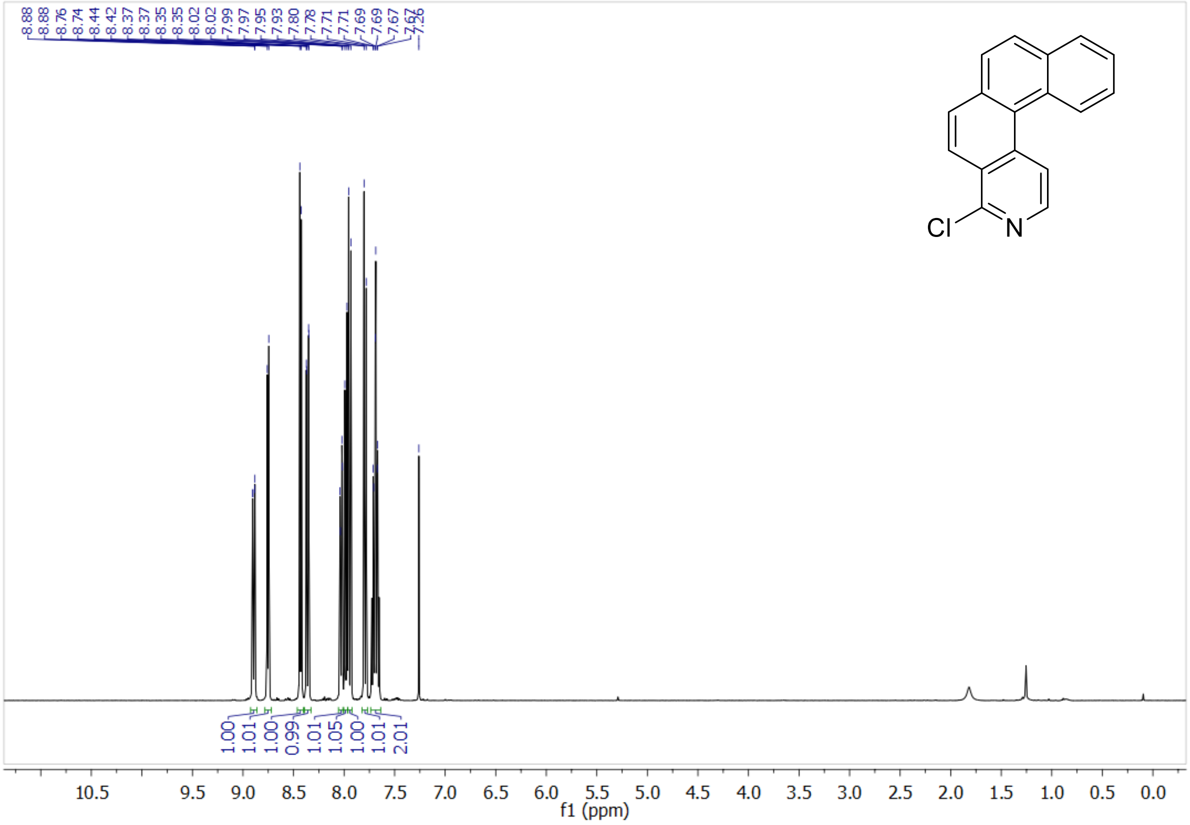

*^{13}C-NMR of 4-chloro[4]pyridohelicene (400 MHz, $CDCl_3$) (**2o**)*

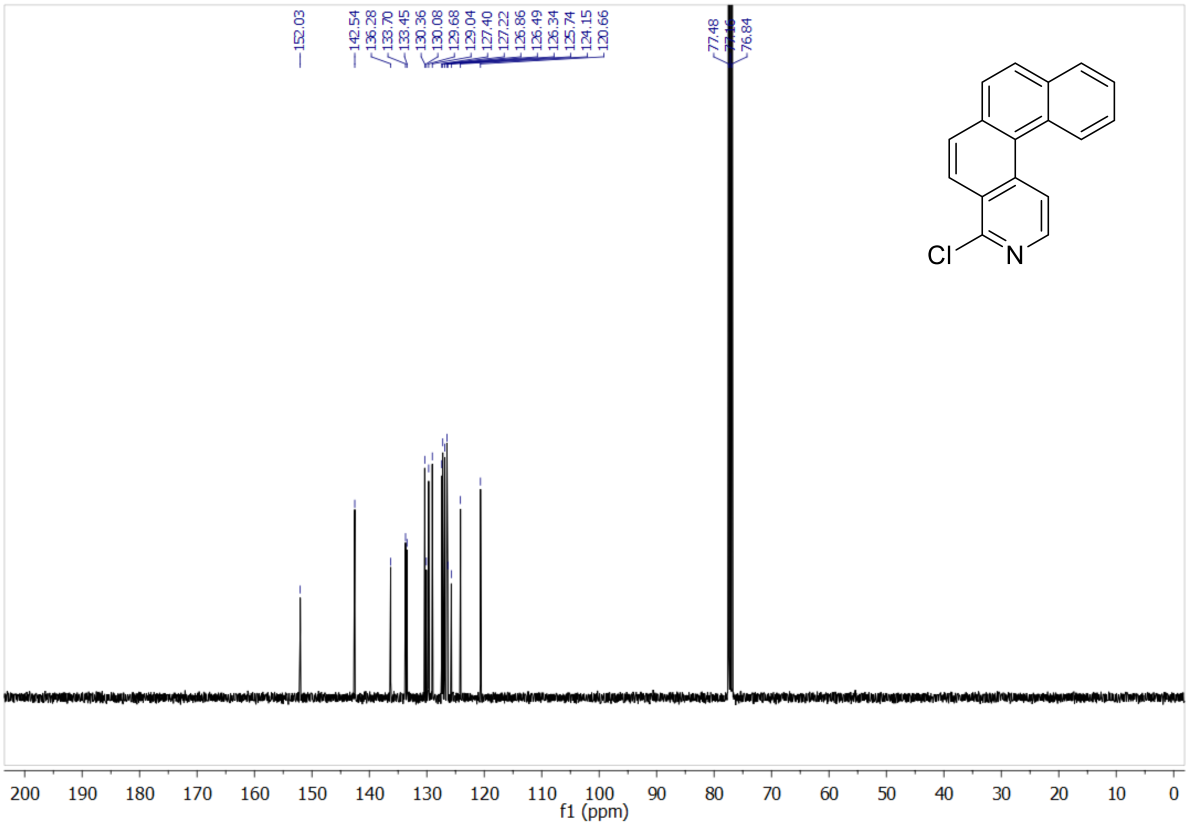

^{1}H-NMR of [7]thienohelicene (400 MHz, $CDCl_3$) (**2p**)

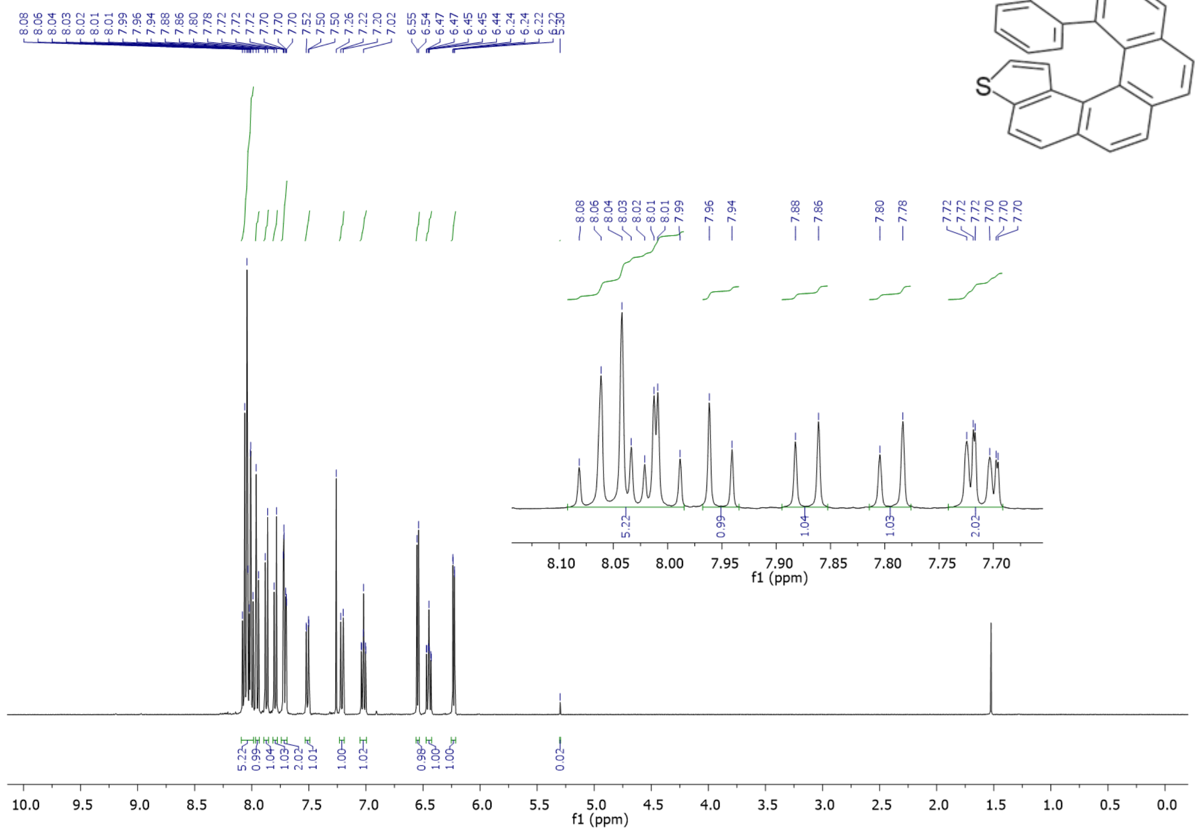

^{13}C-NMR of [7]thienohelicene (101 MHz, $CDCl_3$) (**2p**)

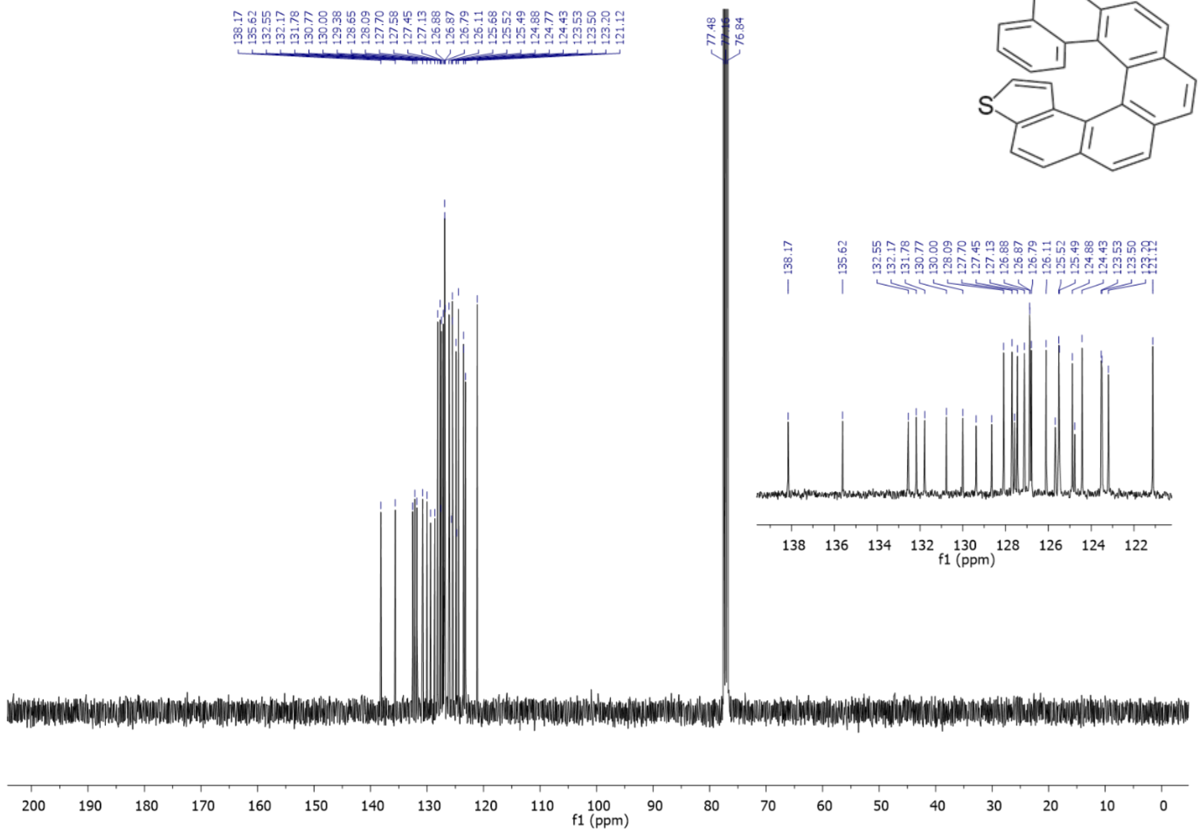

*^{1}H-NMR of naphtho[1',2':8,9]tetrapheno[2,1-b]thiophene (400 MHz, $CDCl_3$) (**S2p'**)*

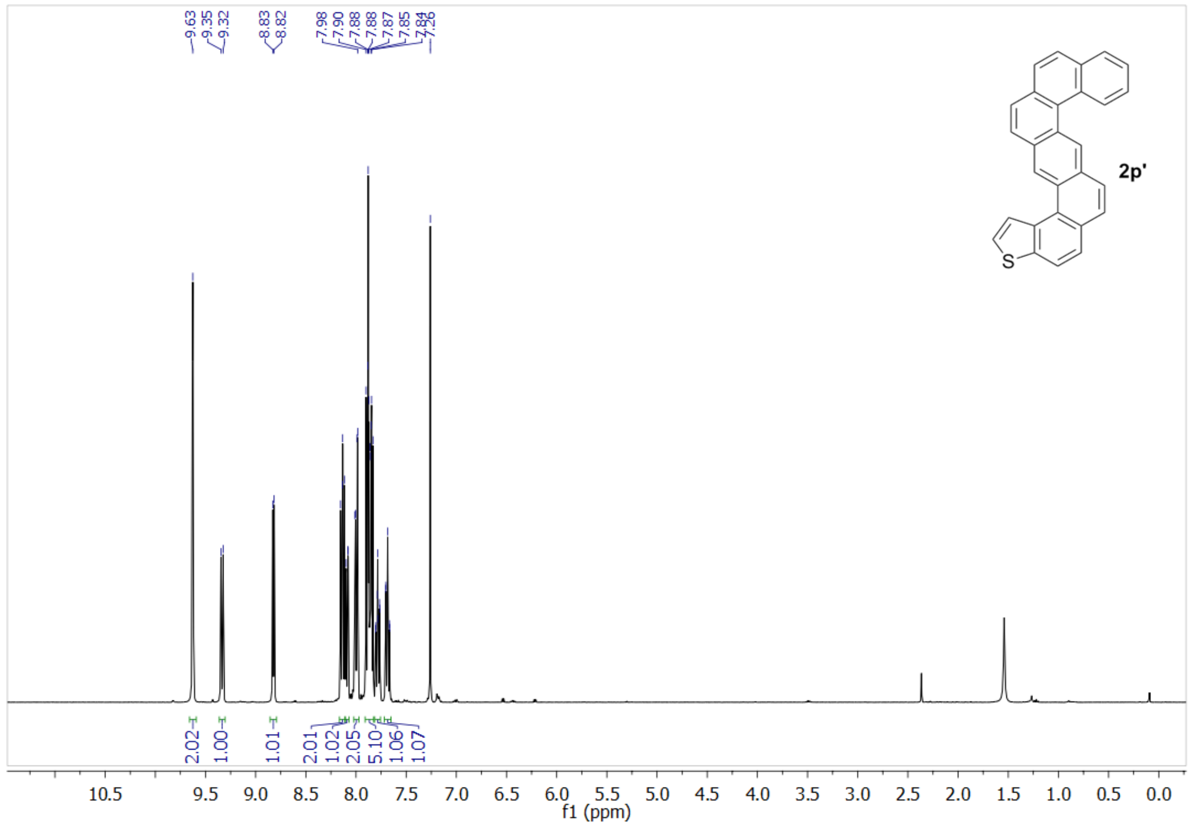

*^{13}C-NMR of naphtho[1',2':8,9]tetrapheno[2,1-b]thiophene (101 MHz, $CDCl_3$) (**S2p'**)*

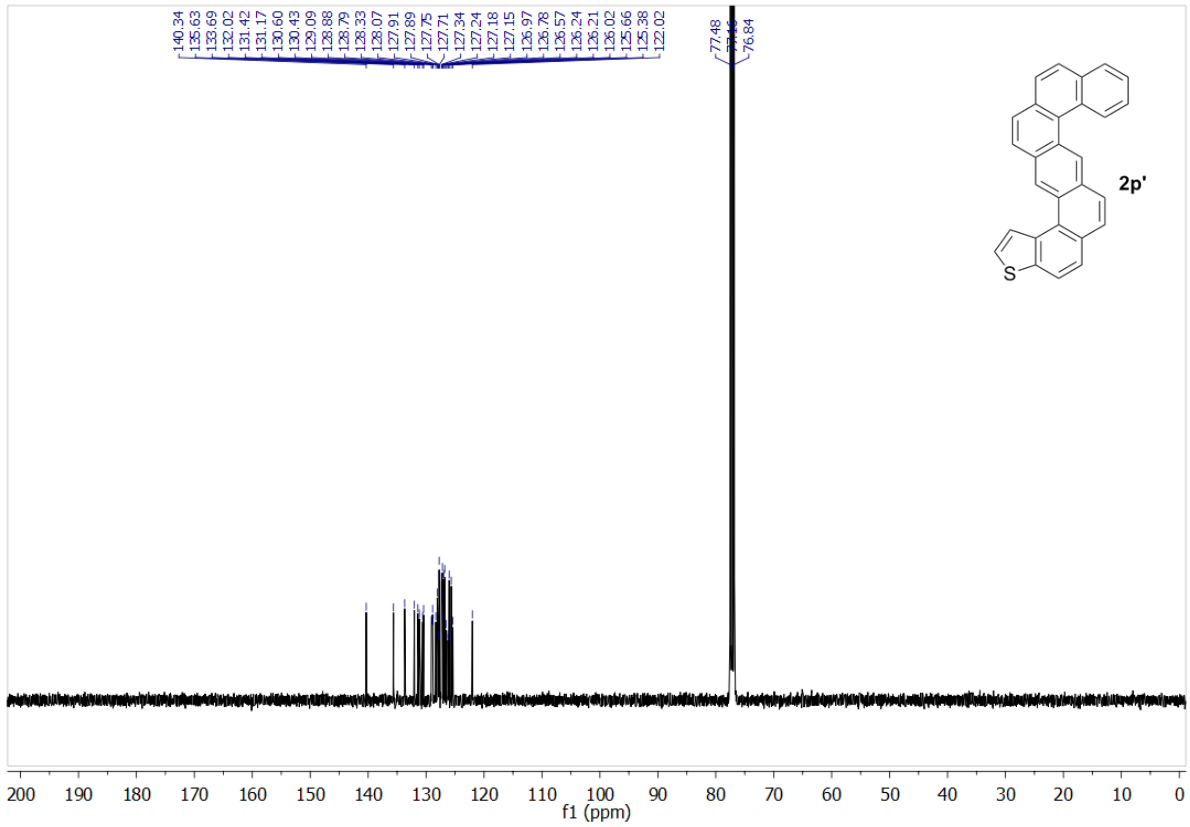

^{1}H-NMR of dibromo[7]pyrrolohelicene (400 MHz, $CDCl_3$) (**2q**)

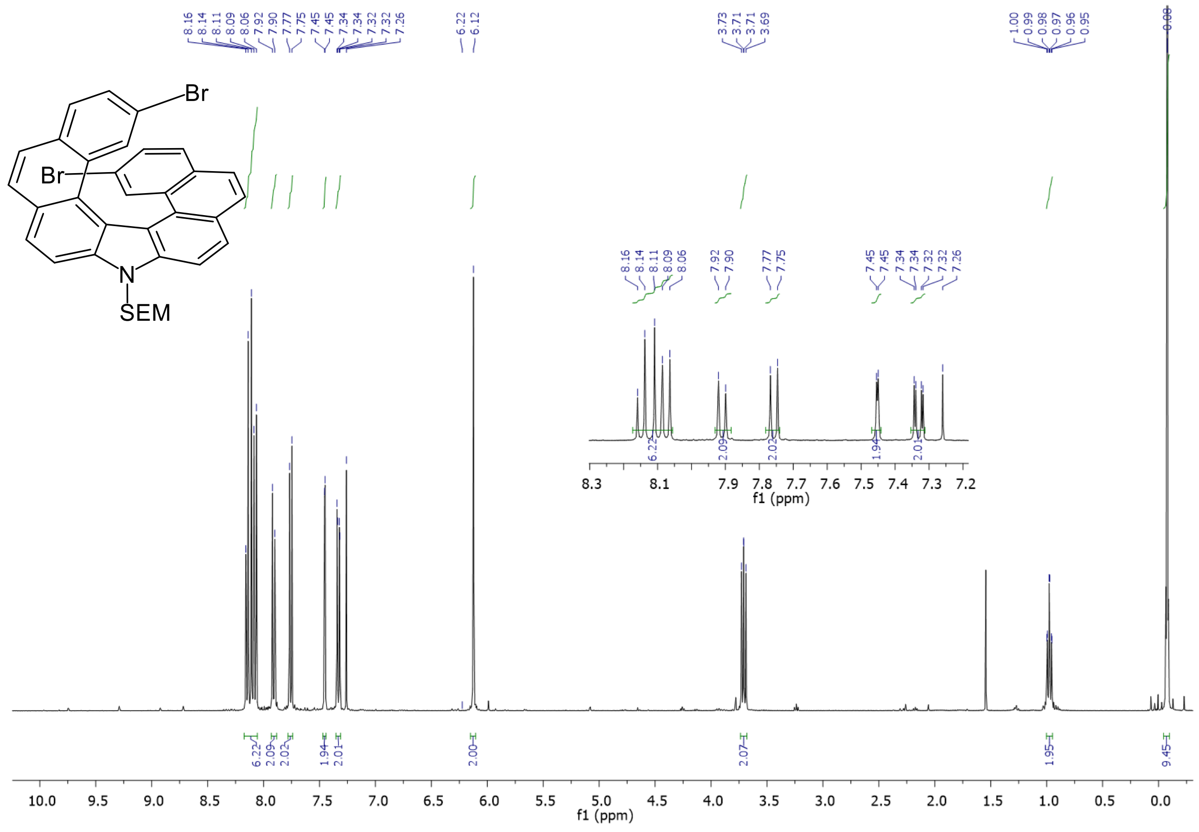

^{13}C-NMR of dibromo[7]pyrrolohelicene (101 MHz, $CDCl_3$) (**2q**)

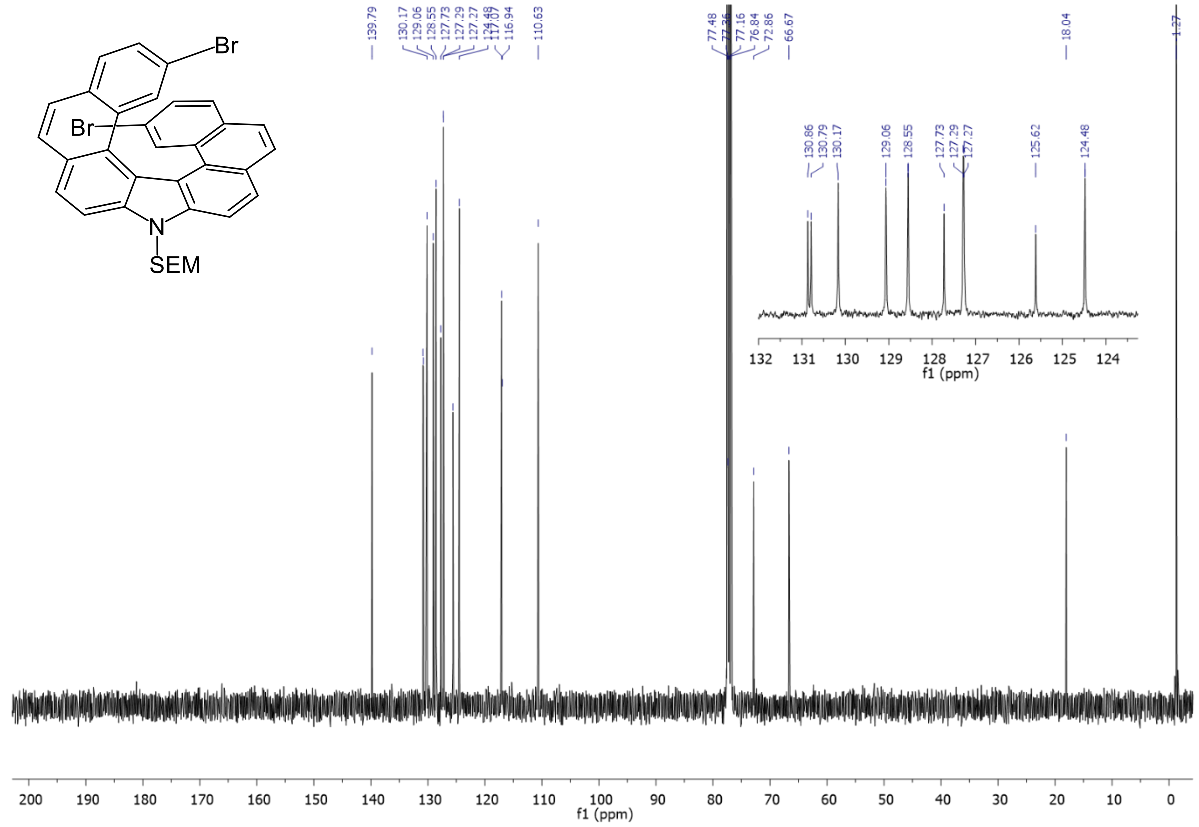

*^{1}H-NMR of (E)-2-styrylphenanthro[3,4-b]thiophene (400 MHz, $CDCl_3$) (**3a**)*

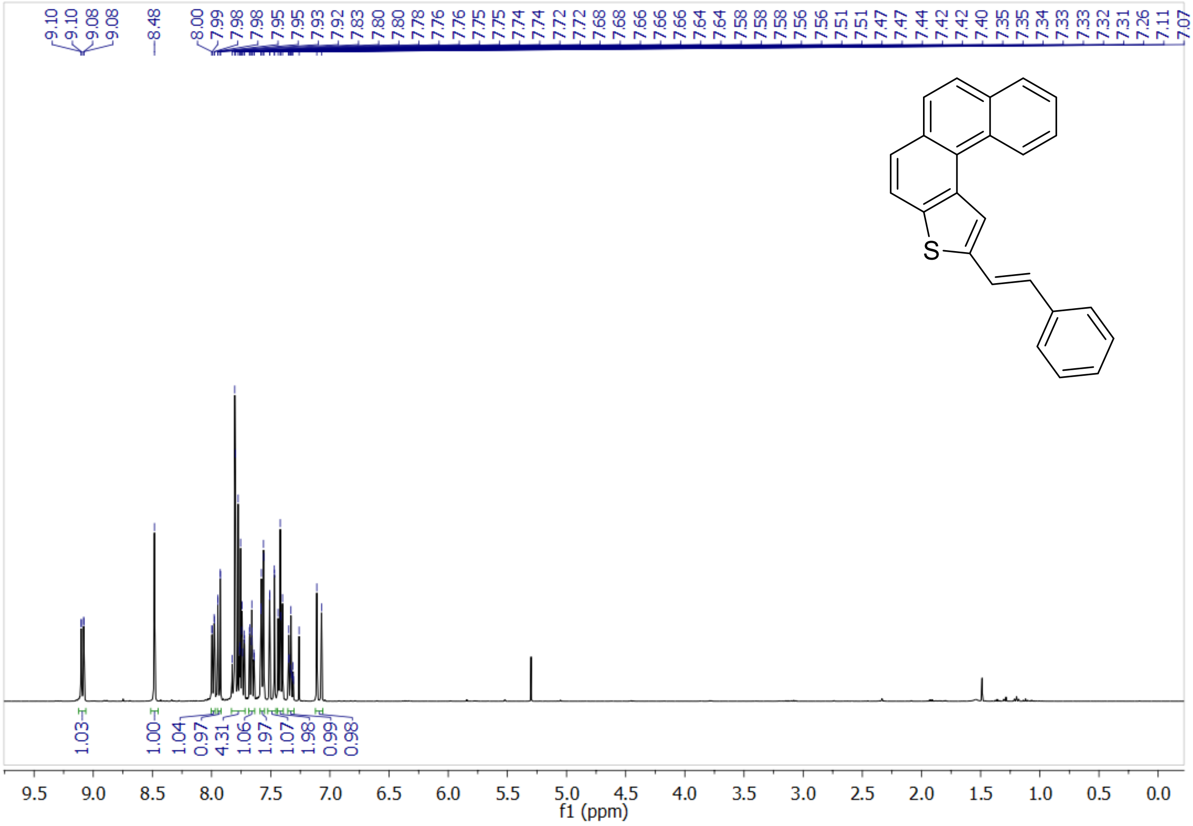

*^{13}C-NMR of (E)-2-styrylphenanthro[3,4-b]thiophene (101 MHz, $CDCl_3$) (**3a**)*

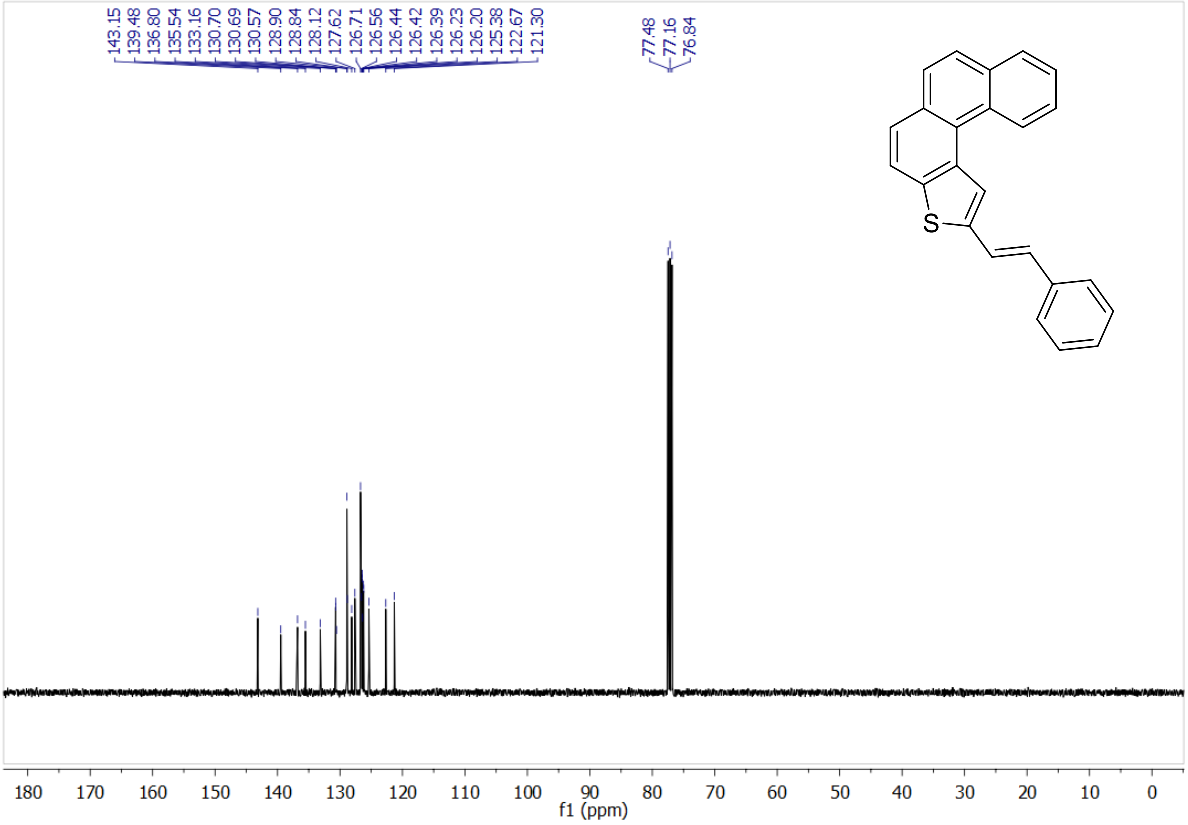

*^{1}H-NMR of [6]thienohelicene (400 MHz, $CDCl_3$) (**4a**)*

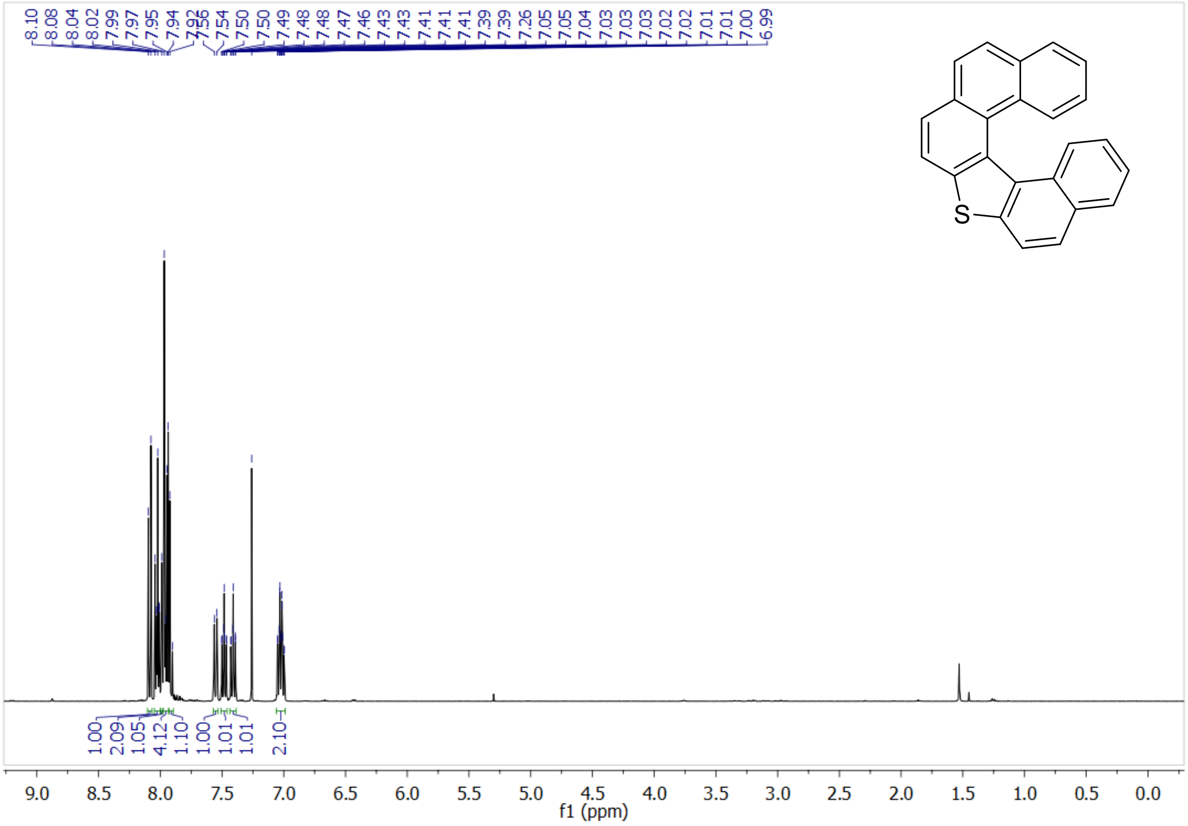

*^{13}C-NMR of [6]thienohelicene (101 MHz, $CDCl_3$) (**4a**)*

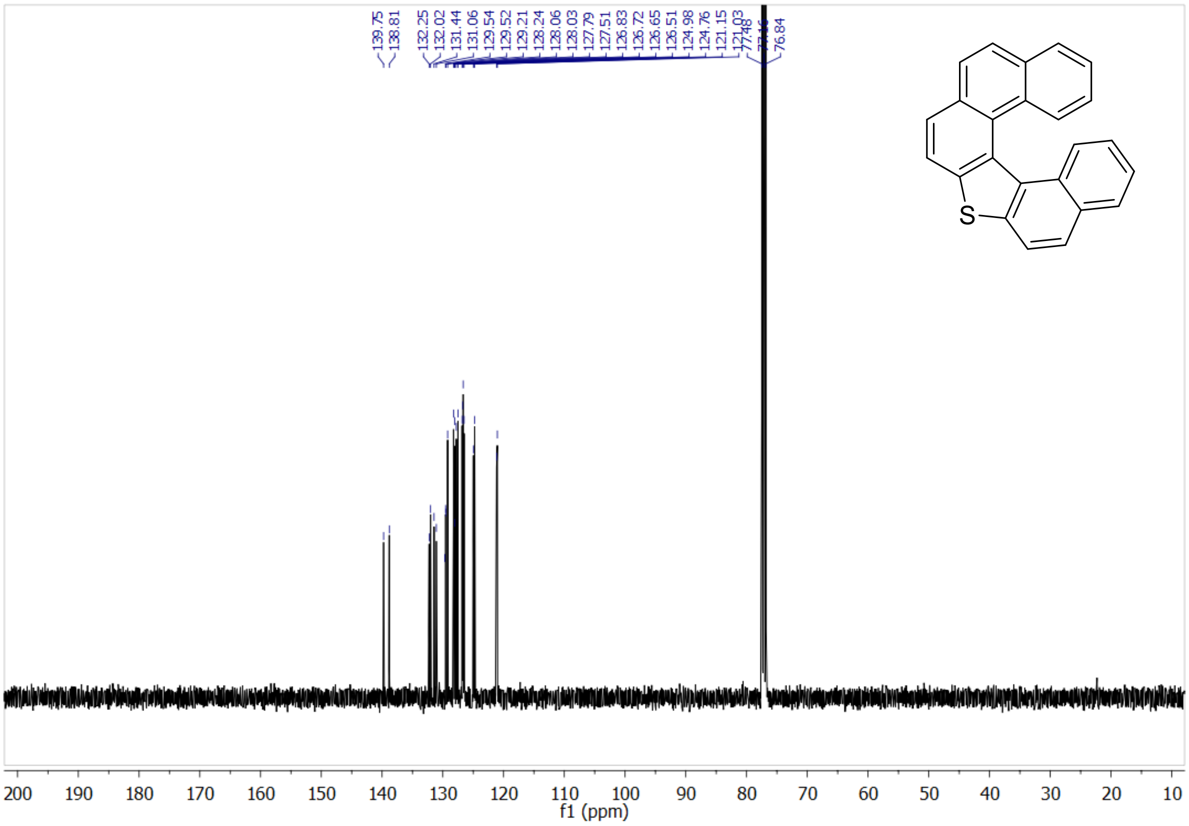

*^{1}H-NMR of (E)-4,4,5,5-tetramethyl-2-(2-(thiophen-2-yl)vinyl)-1,3,2-dioxaborolane (400 MHz, $CDCl_3$) (**S5c**)*

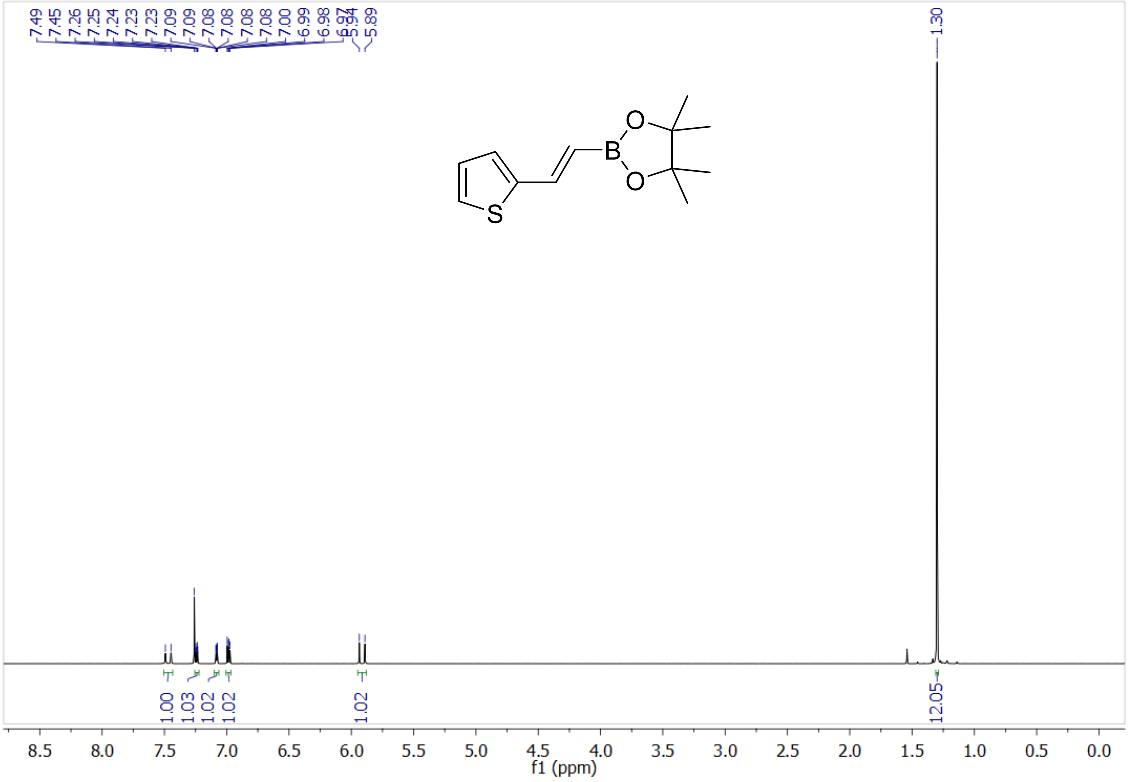

*^{13}C-NMR of (E)-4,4,5,5-tetramethyl-2-(2-(thiophen-2-yl)vinyl)-1,3,2-dioxaborolane (101 MHz, $CDCl_3$) (**S5c**)*

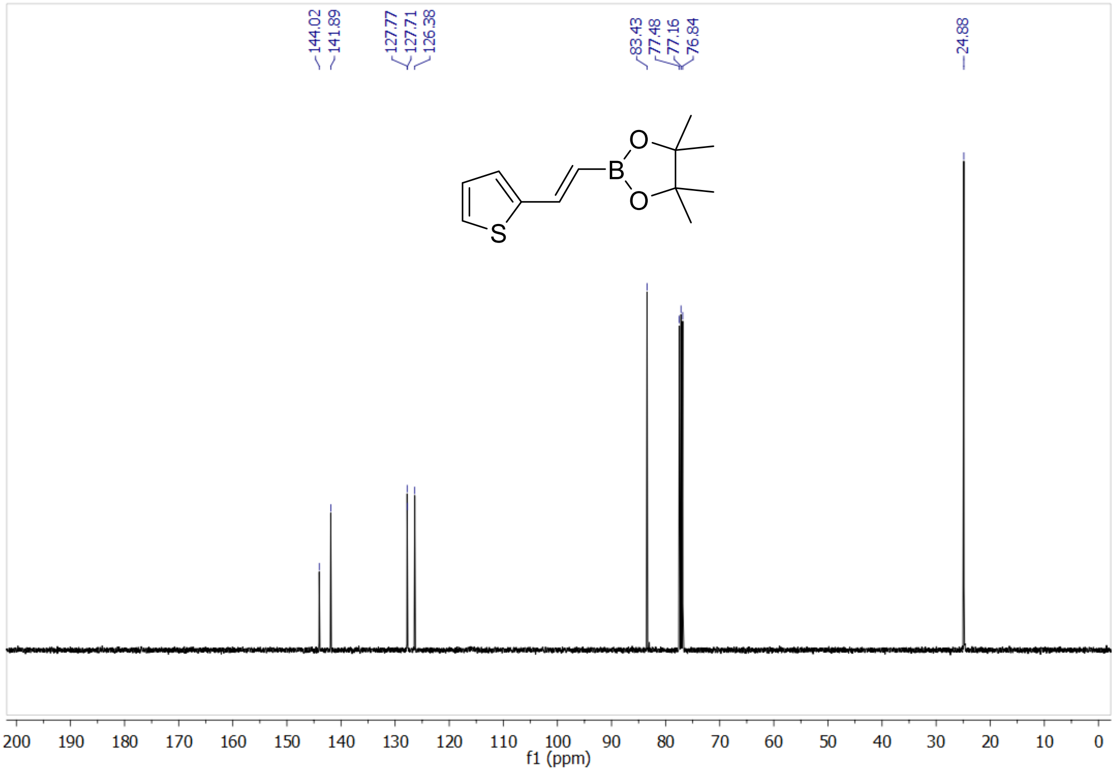

*^{1}H-NMR of (*E*)-2-(2-(thiophen-2-yl)vinyl)phenanthro[3,4-*b*]thiophene (400 MHz, $CDCl_3$) (**3b**)*

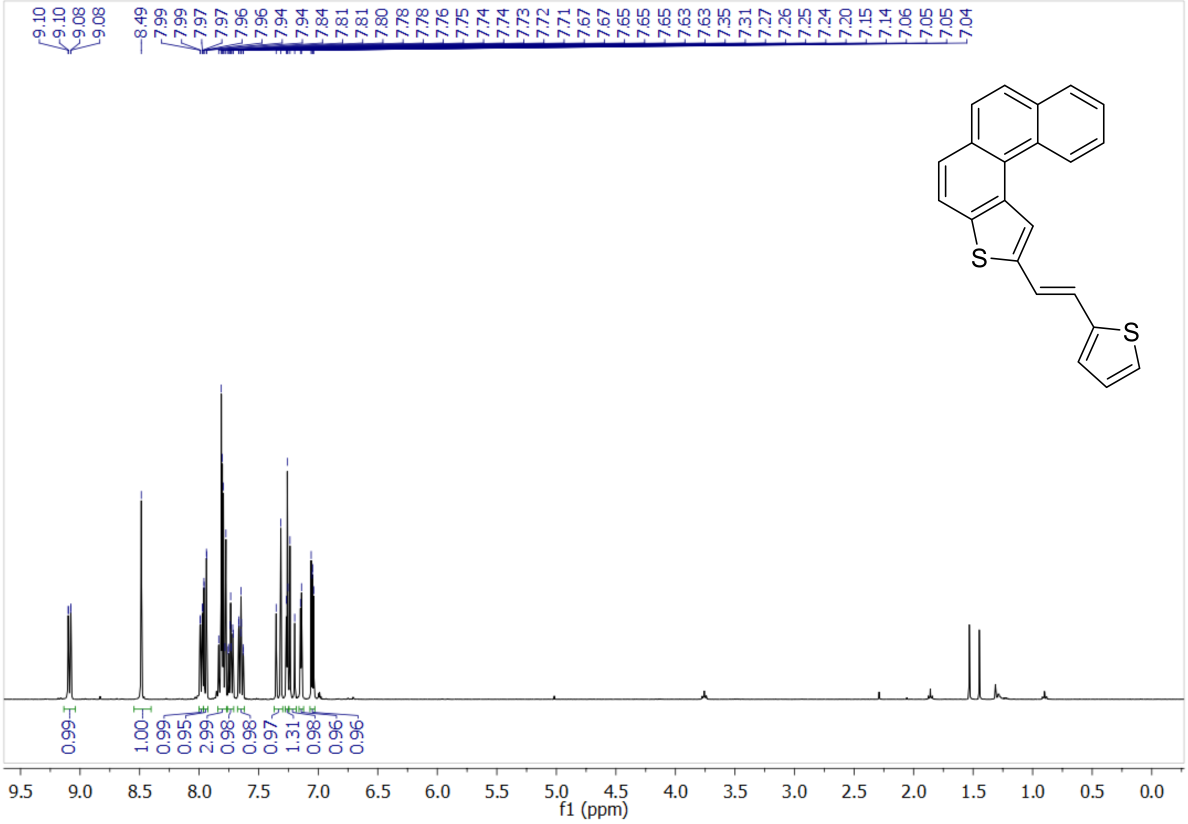

*^{13}C-NMR of (*E*)-2-(2-(thiophen-2-yl)vinyl)phenanthro[3,4-*b*]thiophene (101 MHz, $CDCl_3$) (**3b**)*

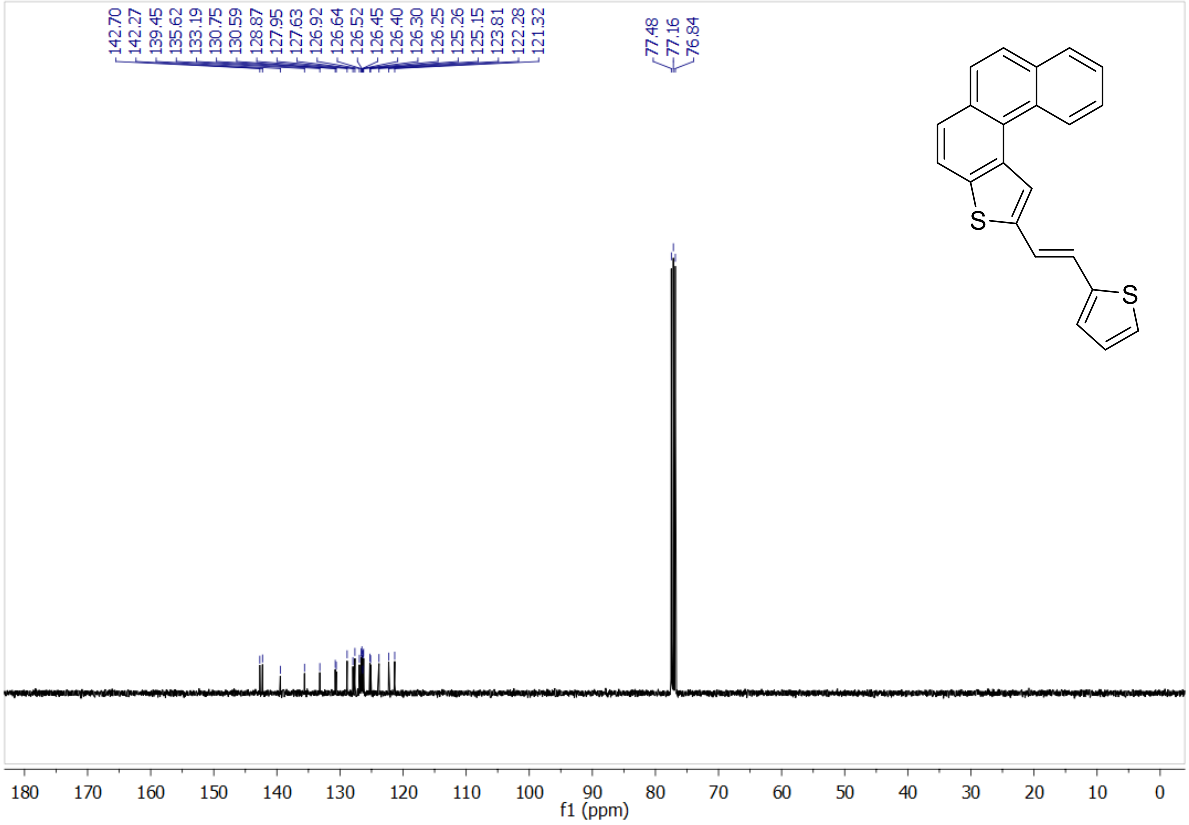

^{1}H-NMR of [6]Dithienohelicene (400 MHz, $CDCl_3$) (**4b**)

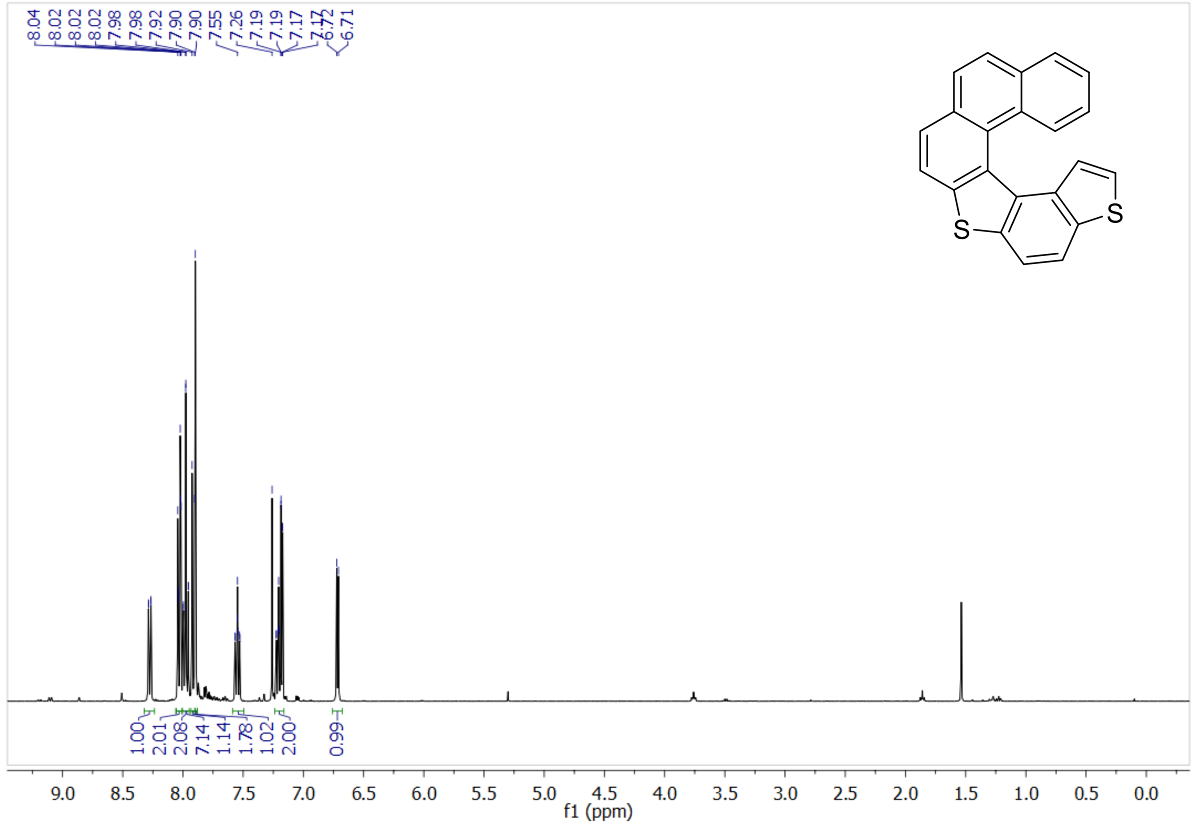

^{13}C-NMR of [6]Dithienohelicene (400 MHz, $CDCl_3$) (**4b**)

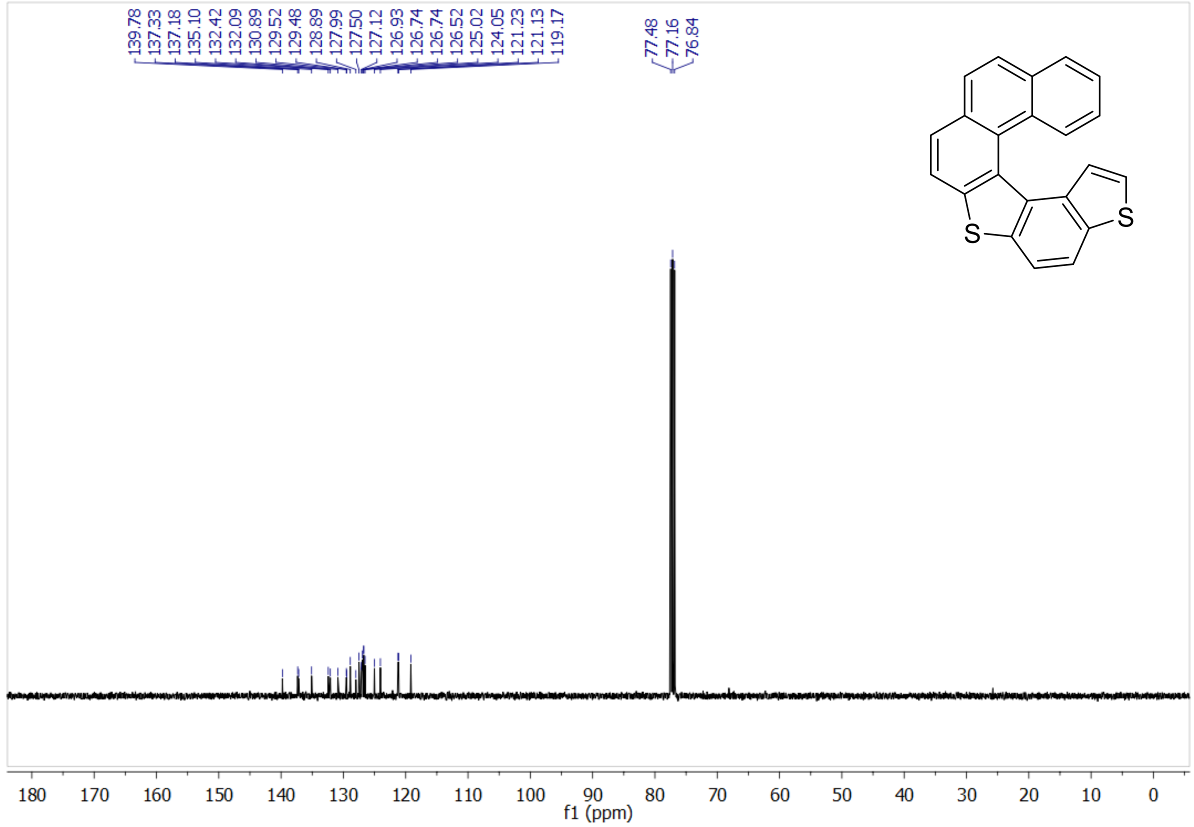

Supplement: Supplementary file 1 [file ol6c00559_si_001.pdf]
